# *GATA2* monoallelic expression underlies reduced penetrance in inherited *GATA2*-mutated MDS/AML

**DOI:** 10.1038/s41375-018-0134-9

**Published:** 2018-04-19

**Authors:** Ahad F. Al Seraihi, Ana Rio-Machin, Kiran Tawana, Csaba Bödör, Jun Wang, Ai Nagano, James A. Heward, Sameena Iqbal, Steven Best, Nicholas Lea, Donal McLornan, Emilia J. Kozyra, Marcin W. Wlodarski, Charlotte M. Niemeyer, Hamish Scott, Chris Hahn, Alicia Ellison, Hemanth Tummala, Shirleny Romualdo Cardoso, Tom Vulliamy, Inderjeet Dokal, Tom Butler, Matthew Smith, Jamie Cavenagh, Jude Fitzgibbon

**Affiliations:** 1 Centre for Haemato-Oncology, Barts Cancer Institute, Queen Mary University of London, London, UK; 2 MTA-SE Lendulet Molecular Oncohematology Research Group, 1st Department of Pathology and Experimental Cancer Research, Semmelweis University, Budapest, Hungary; 3 Centre for Molecular Oncology, Barts Cancer Institute, Queen Mary University of London, London, UK; 4 Laboratory for Molecular Haemato-Oncology, King’s College Hospital NHS Foundation Trust, London, UK; 5 Department of Haematological Medicine, King’s College Hospital, London, UK; 6 University of Freiburg, Faculty of Biology, Freiburg, Germany; 7 Pediatric Hematology and Oncology, University Children’s Hospital Freiburg, Freiburg, Germany; 8 Centre for Cancer Biology, SA Pathology, University of South Australia, Adelaide, SA Australia; 9 Centre for Genomics and Child Health, Blizard Institute, Queen Mary University of London, London, UK; 10 Department of Haemato-Oncology, St. Bartholomew’s Hospital, Barts Health NHS Trust, London, UK

While the majority of myelodysplasia and acute myeloid leukemia (MDS/AML) cases are sporadic, rare familial predisposition syndromes have been delineated and now represent a separate disease entity in the revised World Health Organization (WHO) classification of myeloid neoplasms [1]. Germline mutations in ~14 disease genes have been uncovered thus far, with *GATA2* representing one of the key transcriptional regulators commonly mutated in inherited MDS/AML [2]. Increasing evidence suggests that aberrations in *GATA2* impair its transcription and promoter activation, leading to a loss-of-function, supporting a mechanism of *GATA2* haploinsufficiency [3–5]. Reduced penetrance, the observation that family members carry an identical germline mutation yet display variable clinical manifestations, is common and poses a clinical challenge in the diagnosis and management of familial leukemia's, particularly when identifying “silent” mutation carriers for genetic screening and exclusion as potential stem cell transplant donors [6, 7]. Indeed, we have noted that reduced penetrance is a feature among certain *GATA2*-mutated MDS/AML families [8], especially those harboring missense germline mutations such as c.1061C>T (p.Thr354Met) (Table S1) although the precise molecular explanation of such occurrence has not been investigated.

Analysis of five MDS/AML families harboring p.Thr354Met *GATA2* mutations displayed significant intra- and interfamilial variations in disease latency, phenotype, and penetrance (Figure S1). These observations suggest that individuals require additional co-operating events for the development of overt malignancy within the context of a shared germline mutation. To investigate this hypothesis further, we examined an extensive five-generation pedigree [9] (Fig. 1a) where two first-degree cousins (IV.1 and IV.6) developed high-risk MDS/AML with monosomy 7, while a third cousin (IV.10) presented with recurrent minor infections and significant monocytopenia [0.1 × 10^9^/L] and neutropenia [0.8 × 10^9^/L] in year (yr.) 1–3 which subsequently stabilized (monocyte count, neutrophils [>1 × 10^9^/L]) 3 years after presentation (Fig. 1b). This contrasted with the parental generation (III.1, III.5, and III.7) where mutation carriers remain symptom-free with no evidence of hematopoietic abnormality over 60 years of age.

**Fig. 1:**
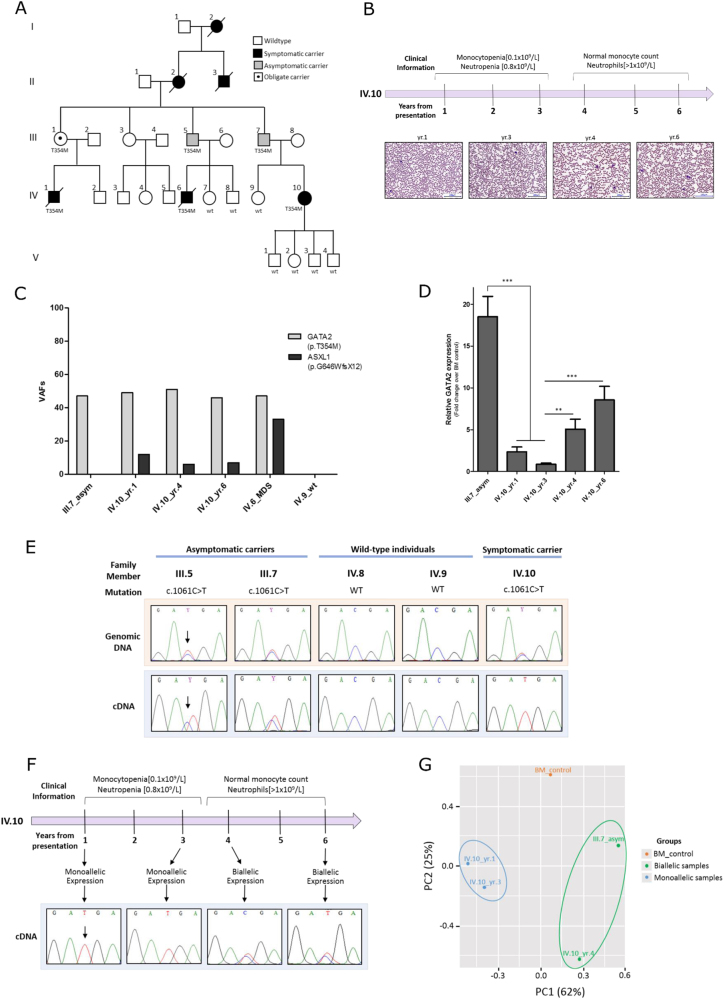
Investigating the molecular mechanisms underlying the reduced penetrance of germline p.Thr354Met mutations observed in a *GATA2-*mutated MDS/AML family. **a** Genogram of the *GATA2-*mutated pedigree. Squares denote males and circles denote females. This five-generation MDS/AML family presented to Barts Health hospital in London with identical germline *GATA2* mutations (p.Thr354Met; c.1061C>T) and variable clinical manifestations. Two first-degree cousins (IV.1 and IV.6) presented at 23 and 18 years of age, respectively, with high-grade MDS transforming to AML and monosomy 7. Both cousins died post allogeneic hematopoietic stem cell transplant (HSCT) due to transplant-related complications (IV.1 from graft vs. host disease (GvHD) and IV.6 from relapsed MDS/AML). Ten years later, their first cousin (IV.10) developed symptoms at 31 years, including recurrent minor infections and significant leukopenia (monocytopenia [0.1 × 10^9^/L] and neutropenia [0.8 × 10^9^/L]) with mild macrocytosis and normal hemoglobin and platelet counts. She remains under close surveillance where her blood counts are routinely monitored. All four of her children have inherited her WT *GATA2* allele. Similarly, members (IV.7, IV.8, and IV.9) were screened for the mutation and all have a WT *GATA2* configuration. The paternal grandmother (II.2) of IV.10 as well as her paternal great-uncle (II.3) and great-grandmother (I.2) all were reported to have died of AML (ages of disease onset were 53, 24, and 53-years old, respectively). Not only did *GATA2* mutations correlate with early age of disease onset in the fourth generation (IV.1/23 yr., IV.6/18 yr., and IV.10/31 yr.), but the parental third-generation carriers (III.1, III.5, and III.7) remain hematologically normal and symptom-free into their mid–late 60s. No material was available from other family members. **b** A clinical timeline of IV.10 showing the change in clinical parameters over the course of disease presentation. Photographs of peripheral blood smears from IV.10 (yr. 1, 3, 4, and 6) stained with May-Grünwald Giemsa staining. Magnification: ×20. **c** Secondary *ASXL1* mutations: variant allele frequencies of *GATA2* germline mutation and *ASXL1* acquired mutation. Samples from three individuals were sequenced: one asymptomatic parent (III.7), one deceased MDS/AML cousin (IV.6), and across three time-points (yr. 1, 4, and 6) from the symptomatic patient (IV.10) reflecting disease evolution. **d** *GATA2* global expression measured by qRT-PCR of bone marrow samples and normalized to healthy bone marrow control: downregulation in IV.10_yr.1 compared with III.7 and downregulation in IV.10_yr.1–3 *GATA2* expression compared with IV.10_yr.4–6. The average of five independent experiments is shown. Statistical significance was determined at *p < 0.05, **p < 0.01, and ***p < 0.001 using a *t*-test with Bonferroni correction. Error bars represent standard error of the mean (SEM). **e** *GATA2* monoallelic expression of the mutant allele in symptomatic (IV.10) vs. asymptomatic carriers (III.5 and III.7), as measured by cDNA sequencing of bone marrow samples. **f** Correlation of monoallelic *GATA2* expression with disease symptoms across the time-points studied in IV.10 with reactivation of the WT allele **“**C**”** expression noted 3 years after presentation, concurrent with improvements in hematological parameters. **g** RNA-seq analysis: principal component analysis (PCA) plot showing a good separation between *GATA2* biallelic (green) and monoallelic (blue) groups based on all transcriptomes

We therefore started with targeted deep sequencing of 33 genes frequently mutated in MDS/AML to define the landscape of secondary genetic mutations across mutation carriers. Notably, while no acquired mutations were detected in asymptomatic family members, all affected cousins analyzed shared an identical somatic *ASXL1* mutation (p.Gly646TrpfsTer12) (Fig. 1c). The variant allele frequency (VAF), however, was lower (12%) in IV.10 and remained stable (range 12–6%) over a 6-year monitoring period. While the co-occurrence of *ASXL1* and *GATA2* mutations has been proposed as one mechanism for driving the onset and severity of disease symptoms [9–11], the low VAF of *ASXL1* mutation and stable improvement in hematopoiesis at IV.10 later follow-up suggested that a combination of *GATA2*–*ASXL1* mutation alone is insufficient to promote clonal expansion and leukemic transformation, as this secondary somatic hit may not represent disease progression or identify when treatment is indicated. Intriguingly, apart from the *ASXL1* mutation, no other acquired mutations were detected in the 33-myeloid genes assessed in the affected individuals. Moreover, on the basis of our observations and in agreement with previous studies [12, 13], it seems that monosomy 7 in IV.1 and IV.6 is acquired following acquisition of *ASXL1* mutations, hence contributing to the malignancy but not initiating symptoms.

We next considered whether disease symptoms are modulated by endogenous levels of *GATA2*. Quantitative real-time PCR (qRT-PCR) of bone marrow material demonstrated total *GATA2* expression to be significantly lower in the symptomatic (IV.10-yr.1) compared with an asymptomatic carrier (III.7) (Fig. 1d). Significantly, Sanger sequencing of the cDNA template revealed striking allele-specific expression (ASE), favoring the mutant (T) allele with the absence of the wild-type (WT) (C) allele expression in the symptomatic patient (IV.10), contrasting with biallelic expression in asymptomatic members (III.5 and III.7) (Fig. 1e). This observation was validated by cDNA cloning of III.7 and IV.10 bone marrow samples and subsequent Sanger sequencing of individual clones (Figure S2). As this suggested that an allelic imbalance in WT:mutant *GATA2* expression ratio may account for the variable disease penetrance in this pedigree, we assessed *GATA2* expression in IV.10 over a 6-year disease period at four time-points (yr. 1, 3, 4, and 6), demonstrating increased *GATA2* expression at later time-points (yr. 4 and 6) (Fig. 1d) coinciding with reactivation of the WT (C) allele expression (Fig. 1f) and an improvement in hematological parameters, in the absence of any clinical intervention (Fig. 1b).

To test whether monoallelic *GATA2* expression has an impact on the transcriptome driving the onset of disease symptoms, we performed RNA-seq with a view of examining downstream biological features distinctive of *GATA2* monoallelic (IV.10-yr.1 and 3) vs. biallelic (IV.10-yr.4 and III.7) groups. Unsupervised analysis revealed a clear separation between *GATA2* monoallelic and biallelic samples (Fig. 1g, S3 and Table S2). It was noteworthy that certain canonical pathways and gene sets related to tumorigenesis (e.g., DNA replication and cell cycle) were enriched in *GATA2* monoallelic vs. biallelic groups (Figure S4), potentially reflecting the clinical and phenotypic switch between these two groups. We also noted a significant overexpression of genes with *GATA2* cofactor *PU.1* motifs in their regulatory regions (*p* value NES = 2.06) in *GATA2* biallelic vs. monoallelic samples, in support of a recent finding [14] that p.Thr354Met mutants bind and interact with PU.1 more tightly than WT, thus leading to sequestration of PU.1 from its normal cellular functions. Consequently, the transcriptional activation triggered by PU.1 will be diminished in our *GATA2* monoallelic samples.

The differences observed in these gene-expression profiles prompted us to explore the molecular mechanisms underlying monoallelic *GATA2* expression. We hypothesized that these allele-specific changes in *GATA2* expression are driven by transient epigenetic mechanisms that include changes in DNA methylation and chromatin mark deposition. A CpG single-nucleotide polymorphism (CpG-SNP) (rs1806462) [C/A] located within the promoter and 5′UTR of *GATA2* overlapping a CpG island offered a marker to distinguish between mutant and WT alleles where this SNP creates/abolishes a CpG dinucleotide within the *GATA2* promoter region (Fig. 2a). More specifically, cDNA sequencing of 5′UTR allowed us to define haplotypes, where the promoter SNP allele (A) resides on the germline mutant *GATA2* allele (T) (Fig. 2a(ii)). Apart from IV.10, no other family members and only 2/12 individuals from pedigrees presented in Figure S1 were heterozygous for this SNP (one of whom is an asymptomatic carrier). Therefore, we do not infer that this haplotype would contribute to the progression of symptoms. Instead, we used this SNP to determine whether allele-specific differences in DNA methylation could explain the silencing of WT *GATA2* allele expression observed in earlier time-points of IV.10. As illustrated in Fig. 2b and S5, bisulfite sequencing of a 200-bp region encompassing rs1806462 demonstrated a significant increase in promoter methylation in the WT allele of IV.10 in yr. 1 and yr. 3 following diagnosis, in contrast with the absence of allele-specific differences in methylation at a later time-point.

**Fig. 2:**
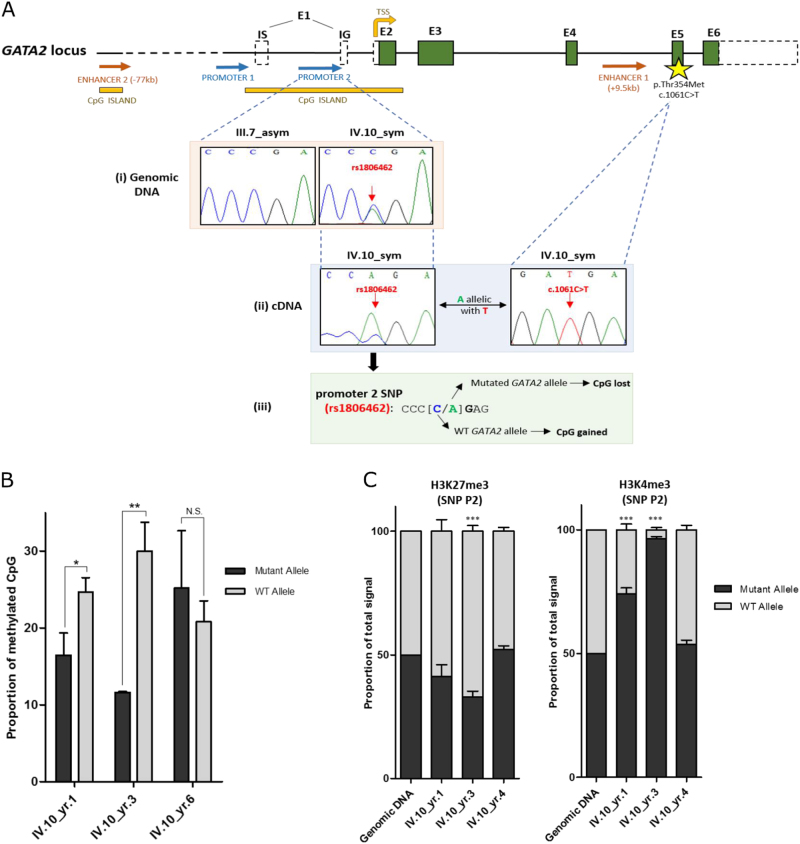
Elucidating the molecular mechanisms driving allele-specific changes in *GATA2* expression. **a(i)** A noncoding SNP (rs1806462 [C/A]) located within the second *GATA2* promoter region overlapping a CpG island was detected in the symptomatic (IV.10) but not in asymptomatic members (III.7). **a(ii)** Given the location of promoter 2 SNP within the 5’UTR, a haplotype between the SNP allele “A” and the germline mutant allele “T” was established, providing a means of distinguishing between mutant and WT alleles in subsequent experiments. **a(iii)** This promoter SNP [C/A] removes a CpG methylation site in the mutant allele “A” and generates a CpG methylation site in the WT allele “C”. **b** The proportion of methylated CpGs between mutant and WT alleles across the three time-points of IV.10. WT allele is significantly more methylated than the mutant allele in monoallelic samples (yr. 1 and yr. 3), whereas no significant allele-specific differences in methylation were observed in a biallelic-expressing sample (yr. 6). The average of three independent experiments is shown. **c** Quantification of mutant and WT allele ChIP sequence peak heights across the time-points of IV.10 based on Sanger sequencing. H3K4me3 activation mark favoring the mutant allele was enriched in monoallelic samples (yr. 1 and yr. 3) compared with the biallelic sample (yr. 4). The average of three independent experiments is shown. Statistical significance was determined at *p < 0.05, **p < 0.01, and ***p < 0.001 using a *t*-test with Bonferroni correction. NS corresponds to nonsignificant comparisons. Error bars represent SEM

We next sought to establish whether these allele-specific changes in *GATA2* methylation and expression are accompanied by changes in chromatin structure at the promoter. H3K4me3 and H3K27me3 define poised or closed chromatin, respectively, rendering them more or less accessible for transcription factors, thereby regulating gene expression [15]. The deposition of these bivalent marks was assessed in IV.10 by allele-specific chromatin immunoprecipitation (ChIP) followed by Sanger sequencing within *GATA2* promoter region encompassing the SNP rs1806462 [C/A]. While there were no apparent allele-specific differences in H3K27me3 deposition across the different time-points of IV.10, an enrichment in the deposition of H3K4me3 on the promoter of the mutant allele (A) relative to the WT allele (C) was noted in IV.10 monoallelic samples (yr. 1 and 3) (Fig. 2c, S6 and S7). In contrast, and consistent with the pattern observed with DNA methylation, there was no demonstrable difference in H3K4me3 deposition in the IV.10 biallelic sample (yr. 4), coinciding with reactivation of the WT allele expression and an overall improvement in clinical parameters. We believe that these observations are in keeping with the notion that H3K4me3 occupancy inhibits de novo DNA methylation [16] which was borne out by subsequent bisulfite sequencing of H3K4me3-enriched DNA from our ChIP experiments, demonstrating that DNA methylation and H3K4me3 deposition are mutually exclusive in our IV.10 samples (Figure S8).

Collectively, our findings provide a step forward in understanding the molecular mechanisms underlying reduced penetrance in *GATA2*-mutated MDS/AML pedigrees, which may be governed by the acquisition of additional co-operating mutations (e.g., *ASXL1*) combined with dynamic epigenetic reprogramming and subsequent allele-specific expression of *GATA2* mutant allele, adding another level of complexity to the (epi)genetic basis of familial MDS/AML.

## Electronic supplementary material

Supplementary Information

Table S2
