# Supplementary material for: GATA2 monoallelic expression underlies reduced penetrance in inherited GATA2-mutated MDS/AML
Source: Leukemia. 2018 Apr 19;32(11):2502–7. doi: 10.1038/s41375-018-0134-9 (PMC6224398; doi:10.1038/s41375-018-0134-9)
Supplement: Supplementary file 1 — Supplementary Information [file 41375_2018_134_MOESM1_ESM.pdf]

Supplementary Information for:

GATA2 Monoallelic Expression Underlies Reduced Penetrance in Inherited *GATA2*-mutated MDS/AML

Ahad F. Al Seraihi[1]*, Ana Rio-Machin[1]*, Kiran Tawana[1], Csaba Bödör[2], Jun Wang[3], Ai Nagano[3], James A. Heward[1], Sameena Iqbal[1], Steven Best[4], Nicholas Lea[4], Donal McLornan[5], Emilia J. Kozyra[6,7], Marcin W. Wlodarski[7], Charlotte M. Niemeyer[7], Hamish Scott[8], Chris Hahn[8], Alicia Ellison[9], Hemanth Tummala[9], Shirleny Romualdo Cardoso[9], Tom Vulliamy[9], Inderjeet Dokal[9], Tom Butler[10], Matthew Smith[10], Jamie Cavenagh[10] and Jude Fitzgibbon[1].

[1]Centre for Haemato-Oncology, Barts Cancer Institute, Queen Mary University of London, London, UK.

[2]MTA-SE Lendulet Molecular Oncohematology Research Group, 1st Department of Pathology and Experimental Cancer Research, Semmelweis University, Budapest, Hungary.

[3]Centre for Molecular Oncology, Barts Cancer Institute, Queen Mary University of London, London, UK.

[4]Laboratory for Molecular Haemato-Oncology, King's College Hospital NHS Foundation Trust, London, UK.

[5]Department of Haematological Medicine, King's College Hospital, London, UK.

[6]University of Freiburg, Faculty of Biology, Freiburg, Germany.

[7]Pediatric Hematology and Oncology, University Children´s Hospital Freiburg, Freiburg, Germany.

[8]Centre for Cancer Biology, SA Pathology, University of South Australia, Adelaide, South Australia, Australia.

[9]Centre for Genomics and Child Health, Blizard Institute, Queen Mary University of London, London, UK.

[10]Department of Haemato-Oncology, St Bartholomew's Hospital, Barts Health NHS Trust, London, UK.

* These authors contributed equally to this work.

Disclosure of Conflicts of Interest: The authors declare no conflicts of interest.

Running Title: Reduced Penetrance in *GATA2* Deficiency.

Correspondence:

Ahad F. Al Seraihi, Centre for Haemato-Oncology, Barts Cancer Institute, Queen Mary University of London, Charterhouse Square, London EC1M 6BQ, United Kingdom; E-mail: a.f.h.alseraihi@qmul.ac.uk

Tel: +44 (0)20 7882 8780

Jude Fitzgibbon, Centre for Haemato-Oncology, Barts Cancer Institute, Queen Mary University of London, Charterhouse Square, London EC1M 6BQ, United Kingdom; E-mail: j.fitzgibbon@qmul.ac.uk

Tel: +44 (0)20 7882 3814

Document Summary: Supplementary Methods, 3 Supplementary Tables and 8 Supplementary Figures.

Supplementary Methods

Familial MDS/AML patient samples and study approval

Bone marrow (BM) and/or peripheral blood (PB) diagnostic material were obtained from select members of the *GATA2*-mutated family as shown in **Figure 1A**: asymptomatic carriers (III.5 and III.7), wild-type individuals (IV.8 and IV.9) and multiple time-points from the symptomatic patient (IV.10: y.1, y.3, yr.4 and yr.6) obtained through regular follow-up. Only genomic DNA samples were available from the two MDS/AML first cousins (IV.1 and IV.6). Samples were collected via referrals from clinicians managing these patients at Barts Health hospital, London. Informed, written consent was obtained for use of samples for research purposes with ethical approval (06/Q0401/31) received in accordance with the Declaration of Helsinki.

Targeted deep sequencing

A targeted myeloid panel of 33 genes (*ASXL1* (exon 12), *BCOR* (all), *CALR* (exon 9), *CBL* (exons 7 + 8 + 9), *CEBPA* (all), *CSF3R* (exons 14 – 17), *DNMT3A* (all), *ETV6* (all), *EZH2* (all), *FLT3* (exons 14 + 15 + 20), *GATA2* (all), *GNAS* (exons 8 + 9), *IDH1* (exon 4), *IDH2* (exon 4), *IKZF1* (all), *JAK2* (exons 12 + 14), *KIT* (exons 2, 8-11, 13 + 17), *KRAS* (exons 2 + 3), *MPL* (exon 10), *NPM1* (exon 12), *NRAS* (exons 2 + 3), *PDGFRA* (exons 12, 14, 18), *PHF6* (all), *PTPN11* (exons 3 + 13), *RUNX1* (all), *SETBP1* (exon 4), *SF3B1* (exons 12 – 16), *SRSF2* (exon 1), *TET2* (all), *TP53* (all), *U2AF1* (exons 2 + 6), *WT1* (exons 7 + 9) and *ZRSR2* (all)) frequently mutated in MDS/AML was employed to determine the secondary mutational profiles across the sequential samples from the symptomatic patient compared with asymptomatic and deceased family members. Target enrichment was achieved using an in-house True SeqCustom Amplicon (TSCA) design (Illumina, San Diego, California, USA). The targeted region consisted of a total of 71Kb represented by 295 amplicons. Pooled library targets were sequenced in batches of 24 samples on the MiSeq sequencing platform, using version 3.0 MiSeq sequencing reagents (Illumina). Minimum read depth threshold was 150 reads; lower limit of sensitivity was 5-10% variant allele frequency (VAF). All variants of unknown significance were eliminated[1].

cDNA synthesis and quantitative real-time PCR (qRT-PCR)

Total RNA was extracted from patient BM aspirates using the RNeasy Kit with an on-column DNase digestion (Qiagen, Valencia, California, USA) as per the manufacturer's instructions and RNA concentrations were determined using Qubit® 3.0 Fluorometer. RNA was then reverse transcribed to cDNA using the High Capacity cDNA Reverse Transcription Kit (Applied Biosystems, Foster City, California, USA). qRT-PCR was performed on the synthesized cDNA using the Universal SYBR® Green assay (Bio-Rad, Hercules, California, USA) to measure *GATA2* expression levels (primers are listed in **Table S3**) relative to the expression of the housekeeping gene (*GAPDH*) on the CFX384 Touch Real-Time PCR Detection System (Bio-Rad). Auto-

threshold was used to yield values of threshold cycle (Ct) and the ΔΔCt method was applied to normalize *GATA2* expression relative to *GAPDH* and using a healthy BM as a positive control.

PCR and Sanger sequencing of genomic DNA and cDNA

Genomic DNA was extracted from patient PB or BM aspirates using the DNeasy Blood & Tissue Kit (Qiagen) following standard procedures. Nucleic acid quality and quantity were measured using the NanoDrop ND-1000 Spectrophotometer. Direct PCR amplification was performed using the 2x ReddyMix PCR Master Mix (Life Technologies Waltham, Massachusetts, USA). Primer sequences encompassing *GATA2* variants of interest (coding or non-coding) are listed in **Table S3**. PCR products were separated by 1.5% agarose gel electrophoresis and then purified using the QIAquick Gel Extraction Kit (Qiagen) according to the manufacturer's protocol. Purified PCR products were then sequenced by bidirectional capillary electrophoresis using the ABI PRISM 3730xl Genetic Analyzer (Applied Biosystems) from GATC Biotech. Sanger sequencing trace chromatograms were visualized using the BioEdit software and compared against a reference human genome sequence.

RNA-seq and data analysis

RNA-seq was performed to compare transcript profiles between *GATA2* monoallelic (IV.10-yr.1 and yr.3) vs. biallelic (IV.10-yr.4 and III.7) groups. Total RNA was quantified and integrity measured using the RNA 6000 nano assay kit on a 2100 BioAnalyzer (Agilent technologies, Santa Clara, California, USA), where RIN > 9.5 was considered high quality RNA. cDNA libraries were prepared using the TruSeq stranded mRNA library preparation kit (Illumina) according to the manufacturers' instructions and sequenced (300 cycles) on the Illumina HiSeq 4000 to generate 2x100bp paired-end sequencing reads by Oxford Genomics. After FASTQ data quality check, raw reads were aligned to the reference genome (GRCh37) using STAMPY[2]. The number of reads uniquely aligned ($q > 10$) to the exonic region of each gene was counted using HTSeq[3] based on the Ensembl annotation. Only genes that achieved at least one count per million (CPM) reads in at least 2 samples (the number of samples analyzed in each group) were included, leading to 17,555 filtered genes in total. Read counts were further normalized using the conditional quantile normalization (CQN) method[4], accounting for gene length and GC content. Differential expression (DE) analyses between *GATA2* biallelic vs. monoallelic groups were performed using the edgeR R package[5], employing the generalized linear model (GLM) approach. The significantly DE genes were selected based on the false discovery rate (FDR) q-value of < 0.05 and the absolute $\log_2$ fold change value of > 1 (**Table S2**). Gene set enrichment analysis (GSEA) was performed using the GSEA tool[6] to identify canonical pathways gene sets acquired from the Molecular Signatures Database (MSigDB-C2 v.5.0). Top significantly enriched gene sets were selected based on FDR q-value of <0.05. RNA-sequencing data have been deposited into Gene Expression Omnibus (GEO) under the accession number: GSE104570.

DNA methylation analysis: bisulfite sequencing

Genomic DNA of symptomatic patient (IV.10) was treated with sodium bisulfite using the Bisulfite Conversion Kit (Active Motif, Carlsbad, California), converting unmethylated cytosines into thymines. Bisulfite-treated DNA was then amplified using primers covering *GATA2* promoter region overlapping a CpG island (**Table S3**). The PCR products were cloned into pCR2.1 vectors using the TA-cloning Kit (Invitrogen, Waltham, Massachusetts, USA) and single colonies were purified and sequenced using vector M13 primers to measure allele-specific differences in *GATA2* promoter methylation.

Chromatin immunoprecipitation (ChIP) assay

ChIP was performed using the ChIP-IT High Sensitivity Kit (Active Motif) and following the manufacturer's instructions. Briefly, the symptomatic patient (IV.10) fresh BM samples (~6 million cells) across three different time-points (y.1, yr.3 and yr.4) were each subject to 5min of formaldehyde fixation to cross-link and maintain protein/DNA interactions. Fixed chromatin was then sonicated for ~15 cycles to an average length of 200bp using a Bio-ruptor. 2µg of H3K4me3 or 1µg of H3K27me3 antibodies (Diagenode, Liege, Belgium) were added separately to sheared chromatin and reactions were incubated on a rotator overnight at 4°C. Chromatin bound to either of these antibodies was then immunoprecipitated using Protein G Agarose beads (Active Motif). ChIP reactions were next subject to several washes by gravity filtration for reverse cross-linking and DNA purification. H3K4me3 or H3K27me3 ChIP-enriched DNA fragments were analyzed by direct PCR and Sanger sequencing within *GATA2* promoter regions to test for allele-specific variations in chromatin deposition (See **Table S3** for primer sequences).

Statistical analysis

Statistical significance for relative levels of gene expression in RT-qPCR, proportions of methylated CpGs in bisulfite sequencing experiments and sequencing peak heights in ChIP experiments was determined at $p<0.05$ (*), $p<0.01$(**) and $p<0.001$(***), calculated using *t*-test with Bonferroni correction. Data are expressed as mean ± standard error of the mean (SEM) and performed using the GraphPad Prsim version 5.0 software.

Supplementary Tables

Table S1. *GATA2*-mutated pedigrees with reduced penetrance.

Family	Germline *GATA2* Mutation	Asymptomatic Carriers	Hematological Malignancy	Reference
1	p.Thr354Met	2	MDS/AML	Hahn *et al*., 2011[7]
2	p.Thr354Met	1	MDS/AML	Hahn *et al*., 2011[7]
3	p.Thr354Met	1	MDS/AML	Hahn *et al*., 2011[7]
4	p.Thr354Met	3	MDS/AML	Bödör et al., 2012[8]
5	p.Arg398Trp	1	MDS/AML	Dickinson *et al*., 2014[9]
6	p.Arg398Gln	3	MDS/AML	Dickinson *et al*., 2014[9]
7	p. Ser447Arg	2	MDS/AML	Mir *et al*., 2015[10]
8	c.10171+572 (C>T), intronic	1	MDS/AML	Churpek *et al*., 2015[11]
9	p.Thr354Met	1	-	Unpublished (German family)
10	c.1017+582(G>T), intronic	3	-	Unpublished (German family)
11	p.Cys349Ser	1	-	Unpublished (German family)

Table S2. List of deferentially expressed (DE) genes between *GATA2* monoallelic and biallelic groups based on gene expression signature analysis from RNA-seq data (an external PDF file).

Table S3. Primer sequences used in this study.

Gene	Forward Primer	Reverse Primer
Intronic PCR primers for Genomic DNA amplification		
GATA2 Exon 5	TTAGCCCTCCTTGACTGAGC	AGCCAAGCTGGATATTGTGG
Exonic RT-qPCR primers for cDNA amplification		
GATA2 Exon 5	ACTCATCAAGCCCAAGCGAA	CTTCATGGTCAGTGGCCTGT
GAPDH	CCATCACCATCTTCCAGGAG	GAGATGATGACCCTTTTGGC
Overlapping PCR primer sets covering GATA2 promoter 2 region **chr3:128205645-128206997 (GRCh37/hg19)**		
Promoter 2 (1)	CGCCAGATACACATACTGATCTC	GCTGGCTTGGGCTTCTTA
Promoter 2 (2)	GACTCCTGCACAGACATGAA	GGGCAGTTGGTGGTTAGTTA
Promoter 2 (3)	TCCGCAATTCCCGAACC	GGGCAGTTGGTGGTTAGTTA
Promoter 2 (4)	GGCCTCCCTAGCAGTAACTAA	AGGTGACTTAGAAGACGGAGAC
Allele-specific methylation-specific PCR (MSP) GATA2 Primers		
Promoter 2 (Methylated)	GGTTTTTGAGAGTGAAGGAGTTTC	CTATACAAAAATCGACAACTAACGC
Promoter 2 (Unmethylated)	GGTTTTTGAGAGTGAAGGAGTTTT	TATACAAAAATCAACAACTAACACC
TA Vector M13	GTAAAACGACGGCCAGT	CAGGAAACAGCTATGAC
Allele-specific ChIP GATA2 primers		
Promoter 2 (141bp)	AACCCCAAACTTACACACGC	CAGCTCCTACCCTGTAAGCC
Promoter 2 (95bp)	GAGAGTGAAGGAGTTCCGGC	CCCCAGCTCCTACCCTGTAA

Supplementary Figures

Figure S1.

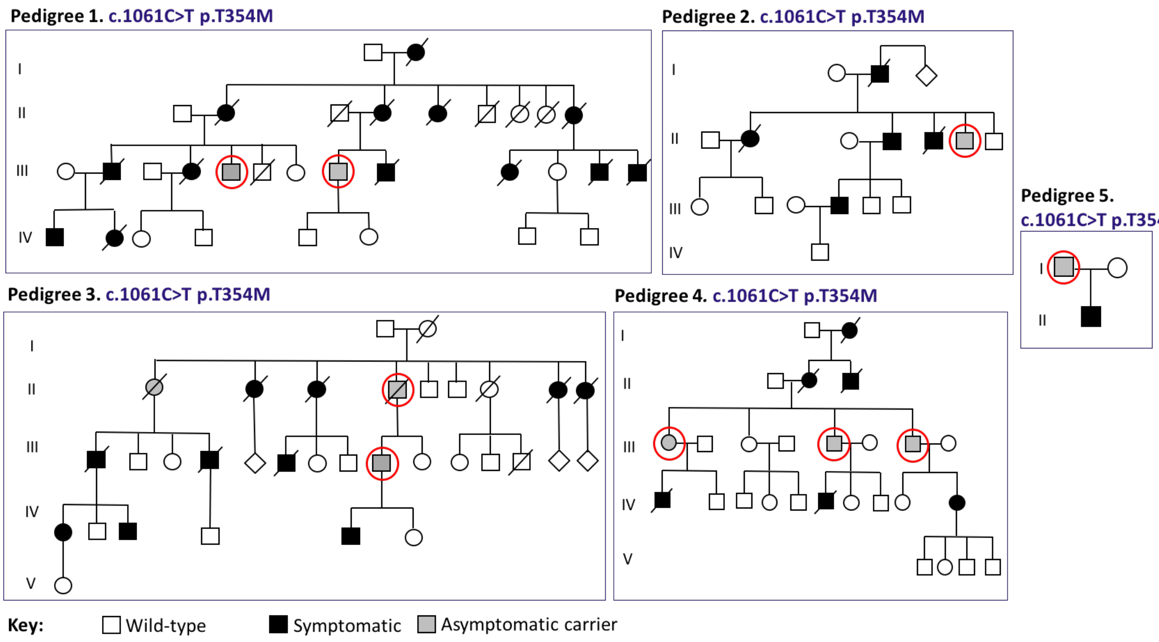

Figure S2.

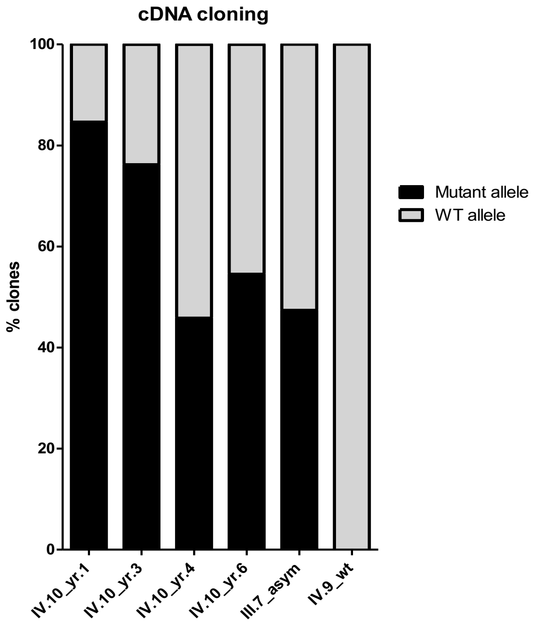

Figure S3.

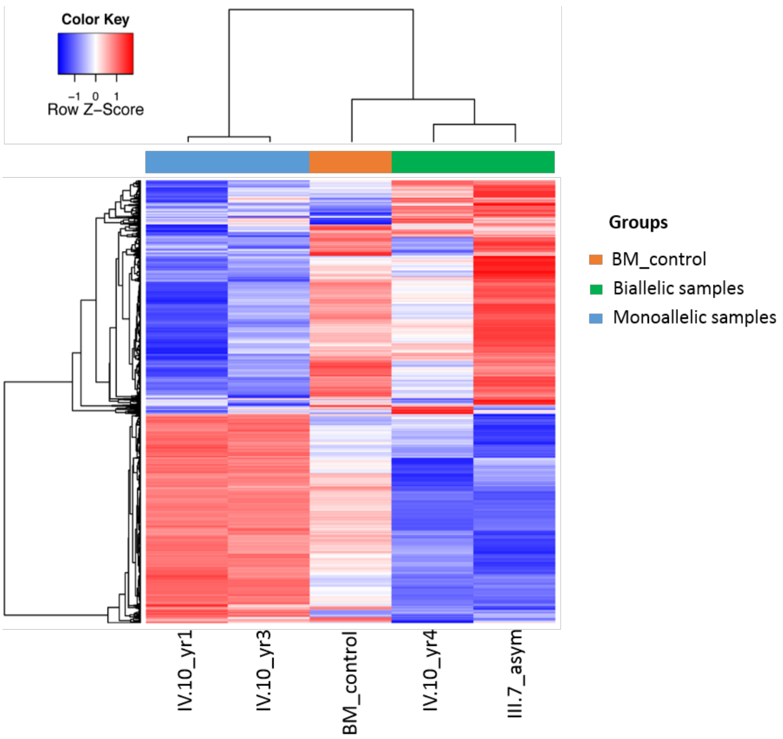

Figure S4.

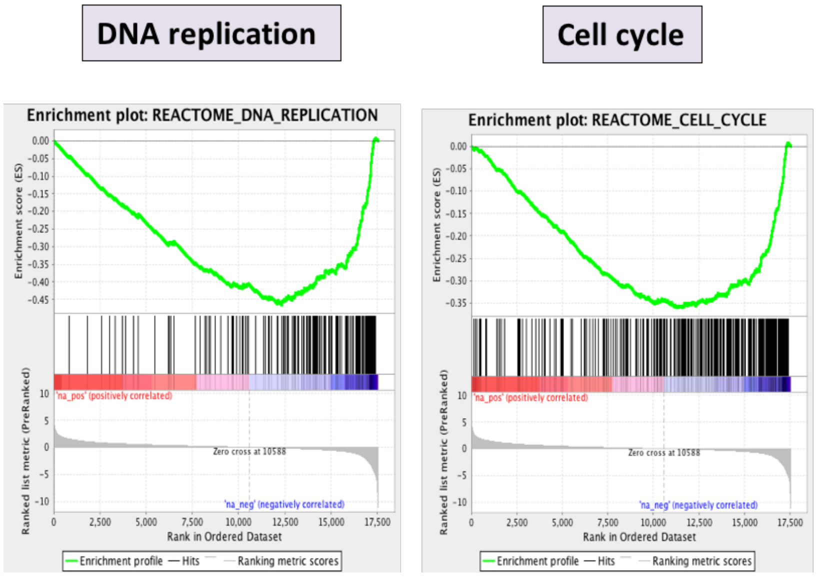

Figure S5.

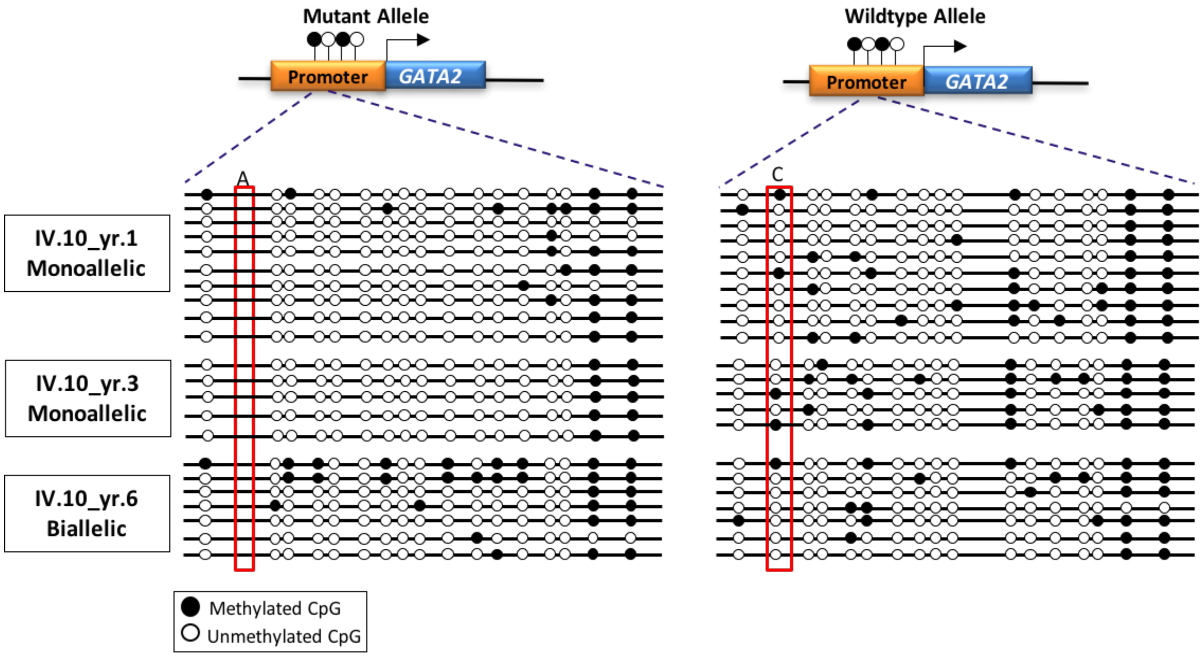

Figure S6.

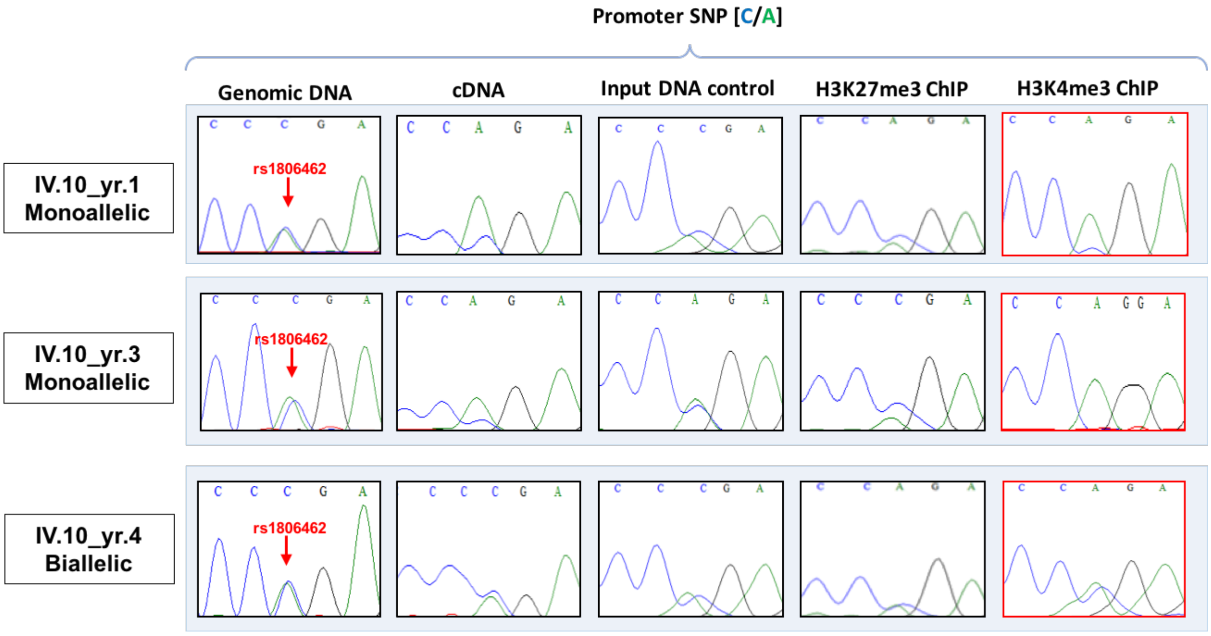

Figure S7.

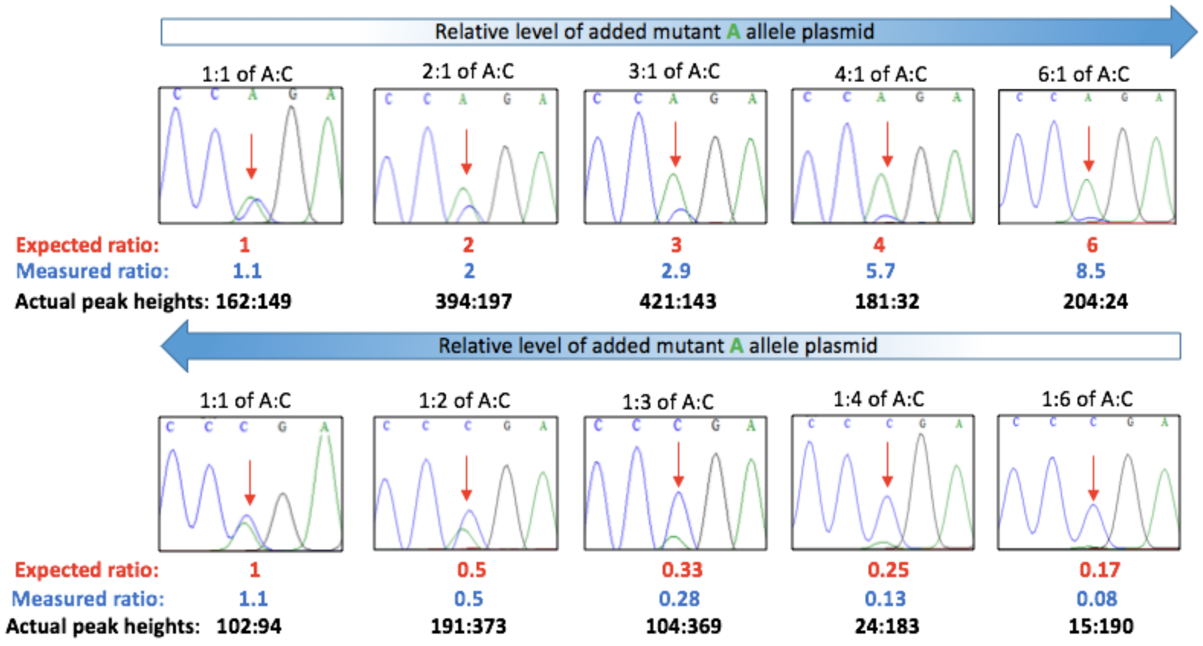

Figure S8.

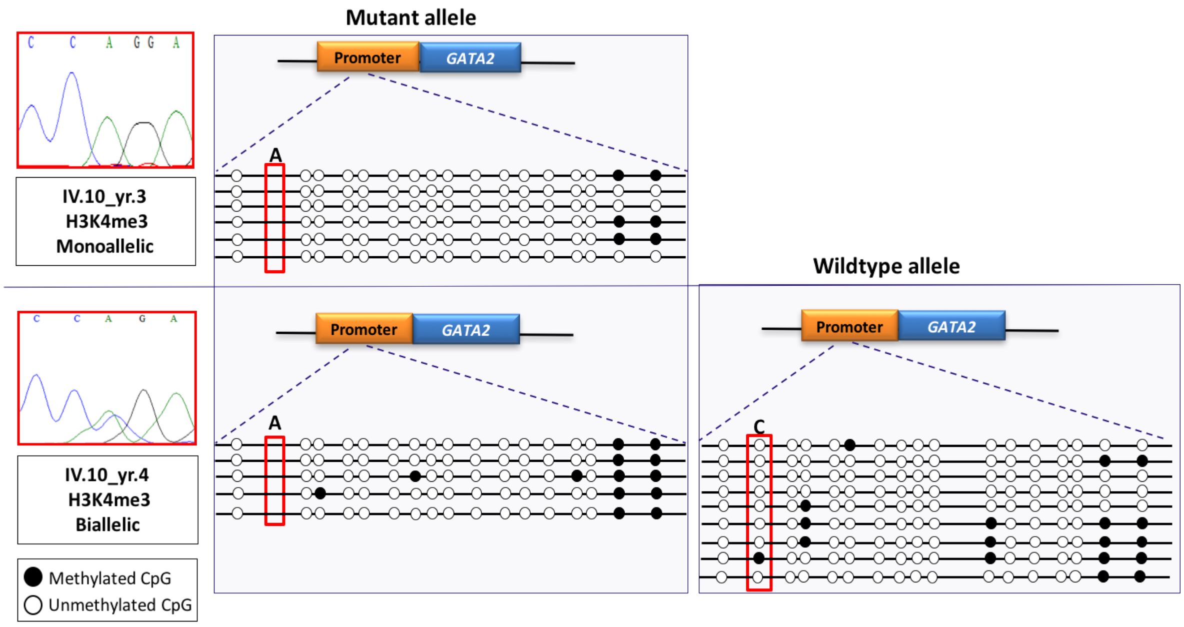

Supplementary Figure Legends

Figure S1. *GATA2* p.Thr354Met families with reduced penetrance. Three pedigrees published by Hahn et al[7] in Australia (pedigrees 1-3), a pedigree investigated in our study[8] (pedigree 4) and an unpublished pedigree by Wlodarski's group in Germany (pedigree 5). Notably, while the germline *GATA2* mutation c.1061C>T (p.Thr354Met) segregates with disease in these pedigrees, not all family members with the mutation developed symptoms of hematological malignancy (asymptomatic carriers are circled in red).

Figure S2. cDNA cloning. The percentage of mutant and WT allele clones across the time-points of IV.10 (yr.1,3,4, and 6) and in III.7 (asym) and IV.9 (WT) based on cDNA cloning and Sanger sequencing of individual clones (approximately 25 clones per sample were analyzed).

Figure S3. RNA-seq. Gene expression signature heat-map analysis revealing 2,432 differentially expressed genes, where 1,148 genes were downregulated and 1,284 were upregulated in *GATA2* biallelic (green) compared with the monoallelic group (blue).

Figure S4. Gene set enrichment analysis (GSEA). Selected canonical pathways and gene sets (DNA replication and cell cycle) were downregulated in *GATA2* biallelic compared with the monoallelic group.

Figure S5. Allele-specific DNA methylation profiles. Bisulfite sequencing of a 200bp region encompassing *GATA2* second promoter SNP [**C**/**A**] overlapping a CpG island (18 CpGs) was performed to compare DNA methylation patterns between mutant (**A**) and WT (**C**) alleles across 3 time-points of the symptomatic patient IV.10 (yr.1 and yr.3 with monoallelic *GATA2* expression) and (yr.6 with biallelic *GATA2* expression). Each row represents a separate clone. Black circles denote methylated CpGs while white circles denote unmethylated CpGs.

Figure S6. Allele-specific enrichment of H3K4me3 and H3K27me3 chromatin marks. The first 2 columns from the left (genomic DNA and cDNA) represent Sanger sequencing trace chromatograms obtained **before** ChIP and following PCR for a region spanning *GATA2* promoter SNP (rs1806462) [**C**/**A**] where (**A**) corresponds to the mutant allele and (**C**) corresponds to the WT allele. The remaining 3 columns represent Sanger sequencing traces obtained **after** ChIP and showing input DNA control, ChIP for H3K27me3 and H3K4me3, respectively, across three sequential time-points from the symptomatic carrier (IV.10_yr.1 and yr.3) with monoallelic *GATA2* expression and (IV.10_yr.4) with biallelic *GATA2* expression.

Figure S7. PCR quality control assay. This assay was performed to confirm that Sanger sequencing results were not due to bias introduced by PCR. Plasmids containing either mutant or WT promoter SNP alleles [**C**/**A**] were introduced together in PCR reactions at different ratios (1:1,2:1,3:1,4:1,6:1) of A:C and the reverse (1:1,1:2,1:3,1:4,1:6) of A:C which was then confirmed by Sanger sequencing chromatograms.

Figure S8. DNA methylation and H3K4me3 deposition are mutually exclusive. Bisulfite sequencing covering *GATA2* promoter SNP [**C**/**A**] region overlapping a CpG island was performed to assess DNA methylation status of H3K4me3 ChIP-enriched DNA across 2 time-points of our symptomatic patient IV.10 (yr.3 with monoallelic *GATA2* expression) and (yr.4 with biallelic *GATA2* expression). Each row represents a separate clone. Black circles denote methylated CpGs while white circles denote unmethylated CpGs.

References for Supplementary Information:

1. Tobiasson M, McLornan DP, Karimi M, et al. Mutations in histone modulators are associated with prolonged survival during azacitidine therapy. *Oncotarget.* 2016.
2. Lunter G, Goodson M. Stampy: a statistical algorithm for sensitive and fast mapping of Illumina sequence reads. *Genome Res.* 2011*;*21(6):936–945.
3. Anders S, Pyl PT, Huber W. HTSeq--a Python framework to work with high-throughput sequencing data. *Bioinformatics.* 2015;31(2):166–175.
4. Hansen KD, Irizarry RA, WU Z. Removing technical variability in RNA-seq data using conditional quantile normalization. *Biostatistics.* 2012;13(2)204–216.
5. Robinson MD, McCarthy DJ, Smyth GK. edgeR: a Bioconductor package for differential expression analysis of digital gene expression data. *Bioinformatics*. 2010;26: pp–1.
6. Subramanian A, Tamayo P, Mootha VK et al. Gene set enrichment analysis: a knowledge-based approach for interpreting genome-wide expression profiles. *Proc Natl Acad Sci USA*. 2005;102(43):15545-15550.
7. Hahn CN, Chong CE, Carmichael CL, et al. Heritable *GATA2* mutations associated with familial myelodysplastic syndrome and acute myeloid leukemia. *Nature Genetics.* 2011;43(10):1012–1019.
8. Bödör C, Renneville A, Smith M, et al. Germ-line *GATA2* p.THR354MET mutation in familial myelodysplastic syndrome with acquired monosomy 7 and *ASXL1* mutation demonstrating rapid onset and poor survival. *Haematologica.* 2012; 97:890–894.
9. Dickinson RE, Milne P, Jardine L, et al. The evolution of cellular deficiency in GATA2 mutation. *Blood.* 2014;123(6): 863–874.
10. Mir MA, Kochuparambil ST, Abraham RS, et al. Spectrum of myeloid neoplasms and immune deficiency associated with germline GATA2 mutations. *Cancer Med.* 2015;4(4):490-9.
11. Churpek JE, Pyrtel K, Kanchi KL, et al. Genomic analysis of germ line and somatic variants in familial myelodysplasia/acute myeloid leukemia. *Blood.* 2015;126(22):2484-90.
