# Supplementary material for: GATA2 monoallelic expression underlies reduced penetrance in inherited GATA2-mutated MDS/AML
Source: Leukemia. 2018 Apr 19;32(11):2502–7. doi: 10.1038/s41375-018-0134-9 (PMC6224398; doi:10.1038/s41375-018-0134-9)
Supplement: Supplementary file 2 — Table S2 [file 41375_2018_134_MOESM2_ESM.pdf]

Table S2. List of 2,432 differentially expressed (DE) genes between *GATA2* biallelic (green) and monoallelic (blue) groups

								GATA2 Monoallelic group		*GATA2* Biallelic group		
**ID**	**Name**	**Description**	**log2FC (Bi_ vs Mono_allelic)**	**logCPM**	**LR**	**PValue**	**FDR**	**IV.10_yr.1**	**IV.10_yr.3**	**IV.10_yr.4**	**III.7_asym**	**BM_control**
ENSG00000112077	RHAG	Rh-associated glycoprotein [Source:HGNC Symbol;Acc:10006]	-7.065450382	5.860144216	250.1161537	2.45E-56	4.30E-52	6.93698598	7.148898694	-0.82139248	-1.572251189	4.628306871
ENSG00000107562	CXCL12	chemokine (C-X-C motif) ligand 12 [Source:HGNC Symbol;Acc:10672]	-8.330878237	6.904575843	221.365128	4.56E-50	4.00E-46	8.463534146	7.896112965	-2.073364206	-0.956081001	2.751827722
ENSG00000169877	AHSP	alpha hemoglobin stabilizing protein [Source:HGNC Symbol;Acc:18075]	-7.680771874	6.64991784	218.0163748	2.45E-49	1.43E-45	7.868211149	7.83620084	0.505333341	-1.854339469	5.243726669
ENSG00000163554	SPTA1	spectrin, alpha, erythrocytic 1 (elliptocytosis 2) [Source:HGNC Symbol;Acc:11272]	-6.718271315	6.277401811	211.6474111	6.00E-48	2.64E-44	6.962770807	7.687236572	-0.589913612	0.154058352	5.777640584
ENSG00000223609	HBD	hemoglobin, delta [Source:HGNC Symbol;Acc:4829]	-6.948481557	7.476636825	209.3272706	1.93E-47	6.76E-44	8.65012583	8.713064225	1.855590689	0.984547783	5.982087099
ENSG00000196415	PRTN3	proteinase 3 [Source:HGNC Symbol;Acc:9495]	-6.967317693	6.019308576	198.8586812	3.71E-45	1.08E-41	6.122897312	6.891813193	-2.073364206	-1.133744865	7.075716419
ENSG00000197993	KEL	Kell blood group, metallo-endopeptidase [Source:HGNC Symbol;Acc:6308]	-6.10505537	5.103502598	194.5611546	3.21E-44	8.06E-41	6.075729559	6.416350763	-0.907612816	-0.874832399	4.138027348
ENSG00000143627	PKLR	pyruvate kinase, liver and RBC [Source:HGNC Symbol;Acc:9020]	-7.880567753	4.966265406	188.39622	7.12E-43	1.56E-39	6.184539705	6.111783523	-2.845623393	-7.002070102	3.799873749
ENSG00000170180	GYPA	glycophorin A (MNS blood group) [Source:HGNC Symbol;Acc:4702]	-7.67602921	5.312758052	187.8619216	9.31E-43	1.82E-39	6.067486521	6.812344661	-3.766908349	-2.97427061	4.224973246
ENSG00000197561	ELANE	elastase, neutrophil expressed [Source:HGNC Symbol;Acc:3309]	-6.023520652	5.626150861	183.3194363	9.13E-42	1.60E-38	5.682950487	6.155375067	-1.57307192	-0.406691783	6.942114908
ENSG00000055118	KCNH2	potassium voltage-gated channel, subfamily H (eag-related), member 2 [Source:HGNC Symbol;Acc:6251]	-5.924295794	6.923850092	181.6691407	2.09E-41	3.34E-38	8.16572184	8.025555626	2.330744516	1.508271876	5.67656232
ENSG00000131747	TOP2A	topoisomerase (DNA) II alpha 170kDa [Source:HGNC Symbol;Acc:11989]	-5.305849613	6.626103217	178.9653596	8.15E-41	1.19E-37	7.516881607	7.660910295	2.1289755	1.926103707	6.641917567
ENSG00000100448	CTSG	cathepsin G [Source:HGNC Symbol;Acc:2532]	-6.007947553	4.87429023	164.0906188	1.45E-37	1.95E-34	5.426778226	5.465512461	-2.845623393	-0.874832399	5.870464764
ENSG00000166947	EPB42	erythrocyte membrane protein band 4.2 [Source:HGNC Symbol;Acc:3381]	-5.874982473	5.431230207	160.912567	7.15E-37	8.96E-34	6.346024108	6.732915862	0.832340008	-1.449512048	4.673975502
ENSG00000211951	IGHV2-26	immunoglobulin heavy variable 2-26 [Source:HGNC Symbol;Acc:5575]	-5.975286828	4.456068793	156.1900471	7.69E-36	9.00E-33	5.80861299	5.379702666	-0.740035668	-1.449512048	3.430160334
ENSG00000211950	IGHV1-24	immunoglobulin heavy variable 1-24 [Source:HGNC Symbol;Acc:5551]	-6.106323229	5.519851109	154.179973	2.12E-35	2.32E-32	7.02191272	6.079121061	0.401591237	-0.589083246	4.886100463
ENSG00000126787	DLGAP5	discs, large (Drosophila) homolog-associated protein 5 [Source:HGNC Symbol;Acc:16864]	-5.030396722	4.916172395	151.7018599	7.36E-35	7.60E-32	6.028383742	5.949930118	0.365283811	0.114810932	4.405023371
ENSG00000039068	CDH1	cadherin 1, type 1, E-cadherin (epithelial) [Source:HGNC Symbol;Acc:1748]	-5.211476345	5.007048086	151.170497	9.62E-35	9.38E-32	6.306294485	6.123695506	0.036330944	0.642123966	3.168221511
ENSG00000243290	IGKV1-12	immunoglobulin kappa variable 1-12 [Source:HGNC Symbol;Acc:5730]	-6.1006739	5.961036193	149.0565118	2.79E-34	2.58E-31	7.514104155	6.586960424	1.098750223	-0.099242291	4.866565467
ENSG00000105610	KLF1	Kruppel-like factor 1 (erythroid) [Source:HGNC Symbol;Acc:6345]	-5.893335408	5.483139827	148.7154157	3.31E-34	2.90E-31	6.696714221	6.563538635	-0.66302287	0.940331404	4.488333455
ENSG00000168754	FAM178B	family with sequence similarity 178, member B [Source:HGNC Symbol;Acc:28036]	-5.635000879	4.527501921	146.0502752	1.27E-33	1.06E-30	5.687439991	5.669431044	-0.99931534	-1.854339469	3.557451401
ENSG00000088325	TPX2	TPX2, microtubule-associated [Source:HGNC Symbol;Acc:1249]	-4.530458654	5.615112454	144.8830773	2.28E-33	1.82E-30	6.672460965	6.601088773	1.89432737	1.820287937	5.263777731
ENSG00000231007	CDC20P1	cell division cycle 20 pseudogene 1 [Source:HGNC Symbol;Acc:29487]	-4.914837736	4.76963257	143.8082952	3.91E-33	2.99E-30	5.992551784	5.715811808	0.663204908	0.466186565	4.077008772
ENSG00000100336	APOL4	apolipoprotein L, 4 [Source:HGNC Symbol;Acc:14867]	-5.404204325	4.551292878	141.9318706	1.01E-32	7.36E-30	5.907365734	5.659645999	-0.059127673	-0.956081001	2.276968566
ENSG00000196188	CTSE	cathepsin E [Source:HGNC Symbol;Acc:2530]	-7.240018267	4.396196948	141.7465507	1.10E-32	7.76E-30	5.275162165	5.882824592	-4.621297774	-3.360122781	2.814341322
ENSG00000206177	HBM	hemoglobin, mu [Source:HGNC Symbol;Acc:4826]	-6.018993929	4.307945359	139.7225011	3.06E-32	2.07E-29	5.402953745	5.522410173	-1.886652327	-3.360122781	3.32911422
ENSG00000075340	ADD2	adducin 2 (beta) [Source:HGNC Symbol;Acc:244]	-4.937267494	6.496103826	138.9965122	4.41E-32	2.87E-29	7.586318744	7.695839822	3.149202463	2.116960417	5.257541658
ENSG00000152078	TMEM56	transmembrane protein 56 [Source:HGNC Symbol;Acc:26477]	-5.101636196	4.71092014	138.7745449	4.93E-32	3.09E-29	5.604410474	5.996020807	-0.907612816	0.192266291	4.039700715
ENSG00000117724	CENPF	centromere protein F, 350/400kDa [Source:HGNC Symbol;Acc:1857]	-4.399528673	5.736076703	137.1273625	1.13E-31	6.85E-29	6.447442448	6.927587689	2.439516168	1.65228493	5.60272092
ENSG00000211937	IGHV2-5	immunoglobulin heavy variable 2-5 [Source:HGNC Symbol;Acc:5576]	-5.788759437	5.939255855	134.1804102	4.99E-31	2.92E-28	7.429800935	6.439885712	1.32354911	-0.053815412	5.453583221

ENSG00000106327	TFR2	transferrin receptor 2 [Source:HGNC Symbol;Acc:11762]	-4.481451745	5.885401724	133.6098056	6.65E-31	3.77E-28	7.090637976	6.929964021	2.213098418	2.444318394	4.750573979
ENSG00000211976	IGHV3-73	immunoglobulin heavy variable 3-73 [Source:HGNC Symbol;Acc:5623]	-5.977982213	4.370701467	133.277693	7.86E-31	4.31E-28	5.707913644	4.960776417	-0.907612816	-2.0191864	4.245284812
ENSG00000070182	SPTB	spectrin, beta, erythrocytic [Source:HGNC Symbol;Acc:11274]	-5.06884676	6.533225014	131.0570654	2.41E-30	1.28E-27	7.358959289	7.907182372	1.992811462	2.913229148	5.554701189
ENSG00000089685	BIRC5	baculoviral IAP repeat containing 5 [Source:HGNC Symbol;Acc:593]	-4.706392021	4.794704565	130.3419074	3.45E-30	1.78E-27	5.988191525	5.74232305	0.632982468	0.871357159	4.175555295
ENSG00000171848	RRM2	ribonucleotide reductase M2 [Source:HGNC Symbol;Acc:10452]	-4.716374845	4.868285637	130.0210308	4.05E-30	2.03E-27	5.74111579	5.914026378	0.437007322	1.0274492	4.835083695
ENSG00000224650	IGHV3-74	immunoglobulin heavy variable 3-74 [Source:HGNC Symbol;Acc:5624]	-6.031477531	6.188290177	127.690626	1.31E-29	6.40E-27	7.652107473	6.825167815	1.582674799	-0.244792505	5.521776442
ENSG00000211955	IGHV3-33	immunoglobulin heavy variable 3-33 [Source:HGNC Symbol;Acc:5596]	-5.498740846	6.76309439	127.524157	1.43E-29	6.77E-27	8.195182702	7.439582655	2.556847448	1.224680533	6.084985712
ENSG00000029534	ANK1	ankyrin 1, erythrocytic [Source:HGNC Symbol;Acc:492]	-4.600610294	7.190314099	125.9028035	3.23E-29	1.49E-26	8.135482519	8.422533838	4.002557365	3.60780319	6.273554241
ENSG00000145386	CCNA2	cyclin A2 [Source:HGNC Symbol;Acc:1578]	-4.223280231	5.109739121	125.4877299	3.98E-29	1.79E-26	6.14264935	6.075445827	1.718398126	1.679439471	4.794309386
ENSG00000087085	ACHE	acetylcholinesterase (Yt blood group) [Source:HGNC Symbol;Acc:108]	-7.198916317	3.948520864	125.3590074	4.25E-29	1.86E-26	5.209254361	5.069467156	-3.233942493	-7.002070102	2.625803327
ENSG00000211962	IGHV1-46	immunoglobulin heavy variable 1-46 [Source:HGNC Symbol;Acc:5554]	-5.149202982	5.577620398	124.6197427	6.16E-29	2.64E-26	6.847949113	6.07973269	1.517996407	0.265774258	5.689591224
ENSG00000148773	MKI67	marker of proliferation Ki-67 [Source:HGNC Symbol;Acc:7107]	-4.443278622	6.563786166	124.4026338	6.88E-29	2.87E-26	7.167208233	7.688039042	2.694908712	2.952717131	6.761762231
ENSG00000211943	IGHV3-15	immunoglobulin heavy variable 3-15 [Source:HGNC Symbol;Acc:5582]	-5.790594543	6.582280331	124.1677086	7.74E-29	3.16E-26	8.142366367	7.174606224	2.139763053	0.74852395	5.514472885
ENSG00000251546	IGKV1D-39	immunoglobulin kappa variable 1D-39 [Source:HGNC Symbol;Acc:5756]	-5.685442172	4.381892865	124.0653229	8.15E-29	3.25E-26	5.793699085	5.039598485	-0.329705368	-1.572251189	3.853782901
ENSG00000211935	IGHV1-3	immunoglobulin heavy variable 1-3 [Source:HGNC Symbol;Acc:5552]	-5.403444604	4.774582821	122.9818688	1.41E-28	5.49E-26	6.118249329	5.236752175	0.168628767	-0.7248947	4.790858932
ENSG00000256269	HMBS	hydroxymethylbilane synthase [Source:HGNC Symbol;Acc:4982]	-3.933682296	6.261220124	119.4150459	8.50E-28	3.24E-25	7.182237296	7.42906119	3.506736406	3.310628593	5.469857628
ENSG00000211625	IGKV3D-20	immunoglobulin kappa variable 3D-20 [Source:HGNC Symbol;Acc:5825]	-5.894485247	4.736654206	119.0692967	1.01E-27	3.78E-25	6.209726581	5.267142457	0.036330944	-1.854339469	4.262825839
ENSG00000134057	CCNB1	cyclin B1 [Source:HGNC Symbol;Acc:1579]	-4.254122795	5.314783612	118.6254601	1.26E-27	4.63E-25	6.504021016	6.211707004	1.919585805	1.992567844	4.703637394
ENSG00000244437	IGKV3-15	immunoglobulin kappa variable 3-15 [Source:HGNC Symbol;Acc:5816]	-4.922634747	6.635197635	118.4795024	1.36E-27	4.88E-25	8.09752888	7.127465328	2.5886897	2.240618578	6.175088092
ENSG00000225698	IGHV3-72	immunoglobulin heavy variable 3-72 [Source:HGNC Symbol;Acc:5622]	-6.038438007	4.730664592	118.4069875	1.41E-27	4.96E-25	6.280970415	5.36170963	0.036330944	-2.205328685	3.62489131
ENSG00000072571	HMMR	hyaluronan-mediated motility receptor (RHAMM) [Source:HGNC Symbol;Acc:5012]	-4.342408058	4.65940497	118.1394811	1.62E-27	5.56E-25	5.735923879	5.629886249	0.75024962	0.722650538	4.314204472
ENSG00000112984	KIF20A	kinesin family member 20A [Source:HGNC Symbol;Acc:9787]	-5.054223802	4.054835521	117.2710672	2.50E-27	8.45E-25	5.180241648	4.951449524	-0.99931534	-0.655391447	3.853782901
ENSG00000133742	CA1	carbonic anhydrase I [Source:HGNC Symbol;Acc:1368]	-7.161992347	7.94844847	116.7036839	3.33E-27	1.10E-24	8.838217754	9.291234283	2.572856422	-0.099242291	7.166048797
ENSG00000169255	B3GALNT1	beta-1,3-N-acetylgalactosaminyltransferase 1 (globoside blood group) [Source:HGNC Symbol;Acc:918]	-4.938772537	4.228220086	115.6528437	5.66E-27	1.84E-24	5.605359776	5.187663739	-0.329705368	-0.525689211	2.758908956
ENSG00000243238	IGKV2-30	immunoglobulin kappa variable 2-30 [Source:HGNC Symbol;Acc:5785]	-5.968750798	5.54245143	114.9868559	7.92E-27	2.53E-24	7.120162256	6.13079577	1.053829154	-1.042179808	4.391417975
ENSG00000211892	IGHG4	immunoglobulin heavy constant gamma 4 (G4m marker) [Source:HGNC Symbol;Acc:5528]	-6.335098493	8.015924055	114.6080527	9.59E-27	3.01E-24	8.590010923	7.895418299	2.448220886	0.74852395	9.373775019
ENSG00000167513	CDT1	chromatin licensing and DNA replication factor 1 [Source:HGNC Symbol;Acc:24576]	-4.326091261	4.500947616	114.367747	1.08E-26	3.33E-24	5.48982274	5.45329231	0.884581662	0.984547783	4.345117047
ENSG00000176890	TYMS	thymidylate synthetase [Source:HGNC Symbol;Acc:12441]	-4.478929501	5.511401825	113.9626567	1.33E-26	4.02E-24	6.566773155	6.388974993	1.360908506	2.045707388	5.40813974
ENSG00000211642	IGLV10-54	immunoglobulin lambda variable 10-54 [Source:HGNC Symbol;Acc:5884]	-5.595282681	4.340854576	113.4216512	1.74E-26	5.19E-24	5.842018808	5.035821043	-0.10933503	-1.854339469	3.27086176
ENSG00000241294	IGKV2-24	immunoglobulin kappa variable 2-24 [Source:HGNC Symbol;Acc:5781]	-5.632456226	5.296048969	113.2711708	1.88E-26	5.51E-24	6.84030886	5.808760266	0.983710868	-0.589083246	4.515764535
ENSG00000211637	IGLV4-69	immunoglobulin lambda variable 4-69 [Source:HGNC Symbol;Acc:5921]	-5.109397867	4.668263214	112.7112819	2.50E-26	7.18E-24	6.125546594	5.196714041	0.210162819	-0.053815412	4.188725022
ENSG00000211938	IGHV3-7	immunoglobulin heavy variable 3-7 [Source:HGNC Symbol;Acc:5620]	-4.985715306	6.82526429	111.6943157	4.17E-26	1.18E-23	8.332940658	7.306914068	2.321298905	2.657338701	6.221321101
ENSG00000240864	IGKV1-16	immunoglobulin kappa variable 1-16 [Source:HGNC Symbol;Acc:5732]	-4.430079843	5.604219858	111.5629661	4.45E-26	1.24E-23	6.770508941	6.106390873	1.501363361	1.770646721	5.873730193
ENSG00000133026	MYH10	myosin, heavy chain 10, non-muscle [Source:HGNC Symbol;Acc:7568]	-4.724774205	4.993304343	111.50872	4.58E-26	1.26E-23	6.059196115	6.190343949	0.036330944	1.43048915	3.95282564

ENSG00000111206	FOXM1	forkhead box M1 [Source:HGNC Symbol;Acc:3818]	-4.327759315	4.822568413	110.3879561	8.06E-26	2.18E-23	5.744566712	5.776678697	0.538319988	1.187332556	4.858347165
ENSG00000172232	AZU1	azurocidin 1 [Source:HGNC Symbol;Acc:913]	-4.650179313	5.657235786	109.4484516	1.29E-25	3.44E-23	5.679348798	6.103987655	0.036330944	1.39816257	7.0045158
ENSG00000011426	ANLN	anillin, actin binding protein [Source:HGNC Symbol;Acc:14082]	-4.322713049	4.52673905	108.8120064	1.78E-25	4.68E-23	5.425703787	5.454236005	0.081791624	0.556835562	4.677716762
ENSG00000198901	PRC1	protein regulator of cytokinesis 1 [Source:HGNC Symbol;Acc:9341]	-3.914980629	5.627851691	108.5096876	2.08E-25	5.37E-23	6.607141062	6.603217413	2.34945221	2.650493526	5.351882062
ENSG00000211953	IGHV3-30	immunoglobulin heavy variable 3-30 [Source:HGNC Symbol;Acc:5591]	-5.532758757	7.596790763	108.4179538	2.18E-25	5.46E-23	9.009978251	8.239367937	3.334328805	1.992567844	7.085254968
ENSG00000101057	MYBL2	v-myb avian myeloblastosis viral oncogene homolog-like 2 [Source:HGNC Symbol;Acc:7548]	-3.906729655	5.461929768	108.4356499	2.16E-25	5.46E-23	6.336908399	6.394885118	2.085000104	2.303059008	5.520735338
ENSG00000211663	IGLV3-19	immunoglobulin lambda variable 3-19 [Source:HGNC Symbol;Acc:5903]	-5.117502932	6.133292464	108.2516323	2.37E-25	5.85E-23	7.606135168	6.790467091	2.321298905	0.894716898	5.25879103
ENSG00000135451	TROAP	trophinin associated protein [Source:HGNC Symbol;Acc:12327]	-4.537649542	4.213556524	107.9444781	2.76E-25	6.71E-23	5.295302869	4.958117738	0.081791624	-0.146146183	4.33330585
ENSG00000137804	NUSAP1	nucleolar and spindle associated protein 1 [Source:HGNC Symbol;Acc:18538]	-3.705922629	5.570012491	107.9265844	2.79E-25	6.71E-23	6.39460723	6.516878082	2.532495892	2.60872607	5.632905242
ENSG00000211660	IGLV2-23	immunoglobulin lambda variable 2-23 [Source:HGNC Symbol;Acc:5890]	-5.671420804	6.767590067	107.5640282	3.35E-25	7.95E-23	8.336661069	7.481149088	2.635172888	0.871357159	5.107514212
ENSG00000085840	ORC1	origin recognition complex, subunit 1 [Source:HGNC Symbol;Acc:8487]	-4.412896472	4.27203214	107.3869896	3.66E-25	8.57E-23	5.456542148	5.26176236	0.036330944	0.265774258	3.557451401
ENSG00000211974	IGHV2-70	immunoglobulin heavy variable 2-70 [Source:HGNC Symbol;Acc:5577]	-4.65657955	4.656702488	107.3272701	3.77E-25	8.72E-23	5.99545132	5.224635763	0.602113338	0.497040107	4.44068937
ENSG00000211897	IGHG3	immunoglobulin heavy constant gamma 3 (G3m marker) [Source:HGNC Symbol;Acc:5527]	-6.469923038	9.546131895	107.2073696	4.01E-25	9.14E-23	11.17050615	10.16257116	4.241637747	3.796351586	7.987804566
ENSG00000211964	IGHV3-48	immunoglobulin heavy variable 3-48 [Source:HGNC Symbol;Acc:5606]	-5.266659167	6.07354345	107.0255812	4.39E-25	9.89E-23	7.500646421	6.636855326	2.107155349	0.527247606	5.646332579
ENSG00000169679	BUB1	BUB1 mitotic checkpoint serine/threonine kinase [Source:HGNC Symbol;Acc:1148]	-3.966307573	4.806368348	106.5472332	5.59E-25	1.24E-22	5.812728526	5.667804805	1.566775005	0.984547783	4.787400207
ENSG00000211947	IGHV3-21	immunoglobulin heavy variable 3-21 [Source:HGNC Symbol;Acc:5586]	-4.967362918	6.504649009	106.3872541	6.06E-25	1.33E-22	7.918153567	7.064279521	2.74522259	1.43048915	6.100399002
ENSG00000253755	IGHGP	immunoglobulin heavy constant gamma P (non-functional) [Source:HGNC Symbol;Acc:5529]	-7.178894447	8.289107689	106.2201767	6.60E-25	1.43E-22	9.899470893	8.994731875	3.049757403	0.940331404	6.464325741
ENSG00000102145	GATA1	GATA binding protein 1 (globin transcription factor 1) [Source:HGNC Symbol;Acc:4170]	-4.264354241	5.111793543	103.8394368	2.19E-24	4.70E-22	6.319081102	6.11715609	1.379231566	2.156150191	4.022150723
ENSG00000066279	ASPM	asp (abnormal spindle) homolog, microcephaly associated (Drosophila) [Source:HGNC Symbol;Acc:19048]	-4.059651384	4.638717839	103.3077864	2.87E-24	6.07E-22	5.132309049	5.827089554	1.120696904	0.871357159	4.826683557
ENSG00000211669	IGLV3-10	immunoglobulin lambda variable 3-10 [Source:HGNC Symbol;Acc:5897]	-5.448457565	4.032401038	102.2505475	4.89E-24	1.02E-21	5.005729219	4.272793714	-1.721358966	-1.854339469	4.884482612
ENSG00000224373	IGHV4-59	immunoglobulin heavy variable 4-59 [Source:HGNC Symbol;Acc:5654]	-5.609929798	6.022710873	102.045395	5.43E-24	1.12E-21	7.372962766	6.457876597	1.907011864	-0.099242291	6.054375789
ENSG00000240382	IGKV1-17	immunoglobulin kappa variable 1-17 [Source:HGNC Symbol;Acc:5733]	-5.678343629	5.503302438	100.6304079	1.11E-23	2.25E-21	6.974574342	6.114173801	1.304500245	-1.133744865	4.835083695
ENSG00000186185	KIF18B	kinesin family member 18B [Source:HGNC Symbol;Acc:27102]	-4.427998276	4.005928759	100.6225931	1.11E-23	2.25E-21	4.957476183	4.998786624	-0.390491059	-0.46496394	3.95282564
ENSG00000115884	SDC1	syndecan 1 [Source:HGNC Symbol;Acc:10658]	-6.131662508	4.083717721	100.3256659	1.29E-23	2.58E-21	5.520341097	4.890006675	-3.766908349	-2.97427061	3.125029543
ENSG00000241351	IGKV3-11	immunoglobulin kappa variable 3-11 [Source:HGNC Symbol;Acc:5815]	-4.50343075	7.436027675	99.19027379	2.29E-23	4.52E-21	8.872136343	8.067016258	3.978722326	3.439318024	6.675049225
ENSG00000211956	IGHV4-34	immunoglobulin heavy variable 4-34 [Source:HGNC Symbol;Acc:5650]	-5.138009485	5.806963886	98.6128289	3.07E-23	5.99E-21	7.203394601	6.18580587	1.855590689	0.265774258	5.772401512
ENSG00000123485	HJURP	Holliday junction recognition protein [Source:HGNC Symbol;Acc:25444]	-4.197440126	4.181720215	98.13396311	3.91E-23	7.54E-21	5.142808642	5.071928522	0.125863448	0.114810932	4.270278622
ENSG00000169607	CKAP2L	cytoskeleton associated protein 2-like [Source:HGNC Symbol;Acc:26877]	-4.392454924	3.937260433	97.98308732	4.22E-23	8.05E-21	4.913697322	4.938019915	-0.390491059	-0.525689211	3.810136354
ENSG00000211638	IGLV8-61	immunoglobulin lambda variable 8-61 [Source:HGNC Symbol;Acc:5931]	-5.028984992	4.536231442	97.86274899	4.48E-23	8.46E-21	5.724610229	4.781402895	0.210162819	-0.956081001	5.075257746
ENSG00000211640	IGLV6-57	immunoglobulin lambda variable 6-57 [Source:HGNC Symbol;Acc:5927]	-5.340751891	5.677114329	97.54002426	5.28E-23	9.86E-21	7.173622725	6.345895385	1.868618824	-0.244792505	4.662693157
ENSG00000242371	IGKV1-39	immunoglobulin kappa variable 1-39 (gene/pseudogene) [Source:HGNC Symbol;Acc:5740]	-5.583737944	4.440653251	95.39693392	1.56E-22	2.88E-20	5.881737769	5.028236357	0.210162819	-2.419096061	3.940421563

ENSG00000225485	ARHGAP23	Rho GTPase activating protein 23 [Source:HGNC Symbol;Acc:29293]	-4.427442671	3.928989317	95.15849956	1.76E-22	3.21E-20	4.815392102	5.162478625	-0.907612816	-0.589083246	3.412306925
ENSG00000211967	IGHV3-53	immunoglobulin heavy variable 3-53 [Source:HGNC Symbol;Acc:5610]	-4.511088934	4.826024946	94.93740198	1.97E-22	3.56E-20	6.062656243	5.133315125	0.983710868	0.434658742	5.181845156
ENSG00000211662	IGLV3-21	immunoglobulin lambda variable 3-21 [Source:HGNC Symbol;Acc:5905]	-6.058839494	7.41620713	94.54567244	2.40E-22	4.29E-20	8.957787089	8.008792486	3.026695712	0.917704421	6.47404004
ENSG00000237649	KIFC1	kinesin family member C1 [Source:HGNC Symbol;Acc:6389]	-3.872038329	4.515417222	94.34744114	2.65E-22	4.69E-20	5.533367836	5.354651283	1.142314722	1.048430793	4.494710155
ENSG00000138160	KIF11	kinesin family member 11 [Source:HGNC Symbol;Acc:6388]	-3.414219242	5.188821107	90.69509655	1.68E-21	2.94E-19	5.94834458	6.106991053	2.6198443	2.003353492	5.365863633
ENSG00000073111	MCM2	minichromosome maintenance complex component 2 [Source:HGNC Symbol;Acc:6944]	-3.347054602	6.16069115	90.58127865	1.78E-21	3.09E-19	7.059111719	7.049680904	3.843391443	3.443284076	6.051495058
ENSG00000143476	DTL	denticleless E3 ubiquitin protein ligase homolog (Drosophila) [Source:HGNC Symbol;Acc:30288]	-3.814706661	4.264745289	89.0364815	3.88E-21	6.67E-19	5.117746192	5.310518437	0.632982468	0.847612951	4.172906862
ENSG00000211959	IGHV4-39	immunoglobulin heavy variable 4-39 [Source:HGNC Symbol;Acc:5651]	-5.177367804	6.78090876	88.72234099	4.54E-21	7.74E-19	8.185852252	7.298531266	2.961306749	1.12943269	6.507008424
ENSG00000129244	ATP1B2	ATPase, Na+/K+ transporting, beta 2 polypeptide [Source:HGNC Symbol;Acc:805]	-5.229161472	3.324857268	88.58242519	4.88E-21	8.23E-19	4.321238306	4.654130775	-2.845623393	-2.419096061	2.087470534
ENSG00000231475	IGHV4-31	immunoglobulin heavy variable 4-31 [Source:HGNC Symbol;Acc:5649]	-5.243021949	6.634106689	88.2558667	5.75E-21	9.62E-19	7.986237702	7.099991828	2.687575605	0.722650538	6.60751686
ENSG00000167900	TK1	thymidine kinase 1, soluble [Source:HGNC Symbol;Acc:11830]	-3.861011849	4.470624011	87.89978744	6.89E-21	1.14E-18	5.366462687	5.490566773	0.778135316	1.331251704	4.328554163
ENSG00000232216	IGHV3-43	immunoglobulin heavy variable 3-43 [Source:HGNC Symbol;Acc:5604]	-5.032944607	4.426273435	87.44777244	8.65E-21	1.42E-18	5.721986739	4.962103922	0.602113338	-1.572251189	4.420735849
ENSG00000178752	FAM132B	family with sequence similarity 132, member B [Source:HGNC Symbol;Acc:26727]	-4.462465838	3.59865254	87.40499743	8.84E-21	1.44E-18	4.838182845	4.773863491	-1.438615935	-0.146146183	1.580441325
ENSG00000167994	RAB3IL1	RAB3A interacting protein (rabin3)-like 1 [Source:HGNC Symbol;Acc:9780]	-6.199340122	3.409869185	87.08642531	1.04E-20	1.66E-18	4.391160537	4.76780347	-4.621297774	-3.888428289	2.153441144
ENSG00000240583	AQP1	aquaporin 1 (Colton blood group) [Source:HGNC Symbol;Acc:633]	-4.368078632	4.024929694	87.08487469	1.04E-20	1.66E-18	4.692133273	5.513383552	-0.520331194	-0.053815412	2.968901188
ENSG00000242076	IGKV1-33	immunoglobulin kappa variable 1-33 [Source:HGNC Symbol;Acc:5737]	-6.367157478	3.505828492	86.93180856	1.12E-20	1.78E-18	5.161001044	3.928913775	-3.233942493	-2.670141381	2.390748373
ENSG00000211933	IGHV6-1	immunoglobulin heavy variable 6-1 [Source:HGNC Symbol;Acc:5662]	-4.687534718	4.35859519	86.82186159	1.19E-20	1.84E-18	5.885650178	4.867514017	0.75024962	-0.350682213	3.524590078
ENSG00000138778	CENPE	centromere protein E, 312kDa [Source:HGNC Symbol;Acc:1856]	-3.60756482	4.419097959	86.83270368	1.18E-20	1.84E-18	5.066276063	5.485036629	1.450281721	1.148991974	4.548825207
ENSG00000104738	MCM4	minichromosome maintenance complex component 4 [Source:HGNC Symbol;Acc:6947]	-3.271902459	6.582582049	86.14810875	1.67E-20	2.57E-18	7.339978958	7.532092247	4.221468244	4.048876701	6.5937216
ENSG00000211893	IGHG2	immunoglobulin heavy constant gamma 2 (G2m marker) [Source:HGNC Symbol;Acc:5526]	-5.387177062	8.966710129	85.71257651	2.08E-20	3.18E-18	10.00992253	8.947091741	4.169775227	3.833089756	9.883404875
ENSG00000211655	IGLV1-36	immunoglobulin lambda variable 1-36 [Source:HGNC Symbol;Acc:5876]	-5.071840489	3.542227229	85.17917758	2.73E-20	4.12E-18	4.729184325	4.131277536	-0.82139248	-2.97427061	3.725915699
ENSG00000135476	ESPL1	extra spindle pole bodies homolog 1 (S. cerevisiae) [Source:HGNC Symbol;Acc:16856]	-3.852063805	4.137641432	84.62225261	3.61E-20	5.42E-18	5.105720809	4.997492463	0.365283811	0.527247606	4.217282073
ENSG00000128274	A4GALT	alpha 1,4-galactosyltransferase [Source:HGNC Symbol;Acc:18149]	-4.644975252	3.450200973	84.42422967	3.99E-20	5.94E-18	4.792235539	4.605689658	-1.097244829	-2.205328685	0.646616095
ENSG00000170312	CDK1	cyclin-dependent kinase 1 [Source:HGNC Symbol;Acc:1722]	-3.363928367	4.84178868	84.28597388	4.28E-20	6.32E-18	5.765100676	5.757693317	2.292585253	2.066427005	4.672101228
ENSG00000244116	IGKV2-28	immunoglobulin kappa variable 2-28 [Source:HGNC Symbol;Acc:5783]	-5.242475207	3.24814524	83.86494637	5.30E-20	7.75E-18	4.618813883	3.915271737	-1.202308962	-2.670141381	2.800681624
ENSG00000142945	KIF2C	kinesin family member 2C [Source:HGNC Symbol;Acc:6393]	-3.811737078	4.18819868	83.64393015	5.92E-20	8.59E-18	5.248673242	5.006527308	0.805492218	0.642123966	4.118890838
ENSG00000204613	TRIM10	tripartite motif containing 10 [Source:HGNC Symbol;Acc:10072]	-4.749550241	4.331449386	83.40172132	6.70E-20	9.64E-18	5.027140012	5.743095465	-1.438615935	0.696304622	3.601447293
ENSG00000121152	NCAPH	non-SMC condensin I complex, subunit H [Source:HGNC Symbol;Acc:1112]	-3.700465287	4.219689028	83.02989384	8.08E-20	1.15E-17	5.161001044	5.154372093	1.285196495	0.466186565	4.140740531
ENSG00000259261	IGHV4OR15-8	immunoglobulin heavy variable 4/OR15-8 (non-functional) [Source:HGNC Symbol;Acc:5658]	-5.40348623	4.427022239	82.49336204	1.06E-19	1.50E-17	5.658462756	4.576518077	-0.059127673	-2.97427061	4.993565856
ENSG00000128268	MGAT3	mannosyl (beta-1,4-)-glycoprotein beta-1,4-N-acetylglucosaminyltransferase [Source:HGNC Symbol;Acc:7046]	-3.547481038	4.464891548	82.27364705	1.18E-19	1.66E-17	5.649286698	5.470185095	1.829176068	1.242998101	3.27086176

ENSG00000127564	PKMYT1	protein kinase, membrane associated tyrosine/threonine 1 [Source:HGNC Symbol;Acc:29650]	-3.896735169	3.96573202	82.25261329	1.20E-19	1.67E-17	4.890520841	4.799338318	-0.059127673	0.402426505	4.172906862
ENSG00000241244	IGKV1D-16	immunoglobulin kappa variable 1D-16 [Source:HGNC Symbol;Acc:5748]	-5.331511831	3.782391755	82.07519469	1.31E-19	1.81E-17	5.197970942	4.227146839	-0.520331194	-2.670141381	3.613216922
ENSG00000183856	IQGAP3	IQ motif containing GTPase activating protein 3 [Source:HGNC Symbol;Acc:20669]	-4.111662435	3.910125814	81.99985683	1.36E-19	1.87E-17	4.590329104	4.59374918	-1.097244829	-0.589083246	4.673975502
ENSG00000223648	IGHV3-64	immunoglobulin heavy variable 3-64 [Source:HGNC Symbol;Acc:5617]	-4.841908393	3.538856045	81.63735092	1.63E-19	2.21E-17	4.650431026	4.08084297	-0.520331194	-2.419096061	3.899355549
ENSG00000086289	EPDR1	ependymin related 1 [Source:HGNC Symbol;Acc:17572]	-4.200677894	3.699258977	81.6341226	1.64E-19	2.21E-17	4.878006108	4.564332671	-0.520331194	-0.009775356	3.305127547
ENSG00000214018	RRM2P3	ribonucleotide reductase M2 polypeptide pseudogene 3 [Source:HGNC Symbol;Acc:10455]	-3.504025323	4.435978789	81.05320102	2.20E-19	2.94E-17	5.34275114	5.392559478	1.517996407	1.567613022	4.285069672
ENSG00000211979	IGHV7-81	immunoglobulin heavy variable 7-81 (non-functional) [Source:HGNC Symbol;Acc:5669]	-5.281401743	3.753317134	80.98602511	2.27E-19	3.02E-17	5.255945862	4.592035301	-0.215316613	-4.730697034	1.958084208
ENSG00000123191	ATP7B	ATPase, Cu++ transporting, beta polypeptide [Source:HGNC Symbol;Acc:870]	-3.691833211	4.089526406	80.81205794	2.48E-19	3.28E-17	5.202996693	5.148553697	0.959559782	0.962608982	3.102938765
ENSG00000122966	CIT	citron (rho-interacting, serine/threonine kinase 21) [Source:HGNC Symbol;Acc:1985]	-4.055432715	4.191627198	80.71838048	2.60E-19	3.41E-17	5.005729219	5.189931642	1.163613388	-0.406691783	4.280156152
ENSG00000160957	RECQL4	RecQ protein-like 4 [Source:HGNC Symbol;Acc:9949]	-3.233602256	4.649702372	80.51808686	2.88E-19	3.75E-17	5.479504787	5.539406699	2.358715867	2.086853261	4.626372277
ENSG00000065328	MCM10	minichromosome maintenance complex component 10 [Source:HGNC Symbol;Acc:18043]	-3.863686362	3.872517633	80.29378464	3.23E-19	4.17E-17	4.80386028	4.950112174	0.036330944	0.301169826	3.561506932
ENSG00000211648	IGLV1-47	immunoglobulin lambda variable 1-47 [Source:HGNC Symbol;Acc:5880]	-4.762512955	5.987112041	80.22297499	3.34E-19	4.25E-17	7.237471374	6.551683587	2.532495892	0.497040107	6.021631095
ENSG00000107789	MINPP1	multiple inositol-polyphosphate phosphatase 1 [Source:HGNC Symbol;Acc:7102]	-3.13464685	5.378091212	80.22806729	3.34E-19	4.25E-17	6.194667282	6.481212079	3.30587588	2.691085717	4.841768766
ENSG00000122952	ZWINT	ZW10 interacting kinetochore protein [Source:HGNC Symbol;Acc:13195]	-3.280830841	4.60710314	79.82007097	4.10E-19	5.18E-17	5.519334152	5.472049919	2.202847421	2.086853261	4.488333455
ENSG00000242534	IGKV2D-28	immunoglobulin kappa variable 2D-28 [Source:HGNC Symbol;Acc:5799]	-5.047527937	3.365020595	79.55819	4.68E-19	5.87E-17	4.820306233	3.99265773	-0.740035668	-2.205328685	2.641213969
ENSG00000243264	IGKV2D-29	immunoglobulin kappa variable 2D-29 [Source:HGNC Symbol;Acc:5800]	-5.147711735	3.894398799	79.54314369	4.72E-19	5.87E-17	5.579506799	4.447875012	-0.010608983	-1.449512048	1.612068369
ENSG00000214998	AC006465.4		-4.362980181	3.289251808	79.09424788	5.92E-19	7.32E-17	4.478721339	4.207132934	-0.907612816	-0.589083246	2.723150012
ENSG00000133488	SEC14L4	SEC14-like 4 (S. cerevisiae) [Source:HGNC Symbol;Acc:20627]	-7.275622228	3.007903877	79.04463718	6.07E-19	7.45E-17	3.918660435	4.372005251	-4.621297774	-7.002070102	1.994537666
ENSG00000211685	IGLC7	immunoglobulin lambda constant 7 [Source:HGNC Symbol;Acc:5861]	-5.128345401	3.625916121	78.87372324	6.62E-19	8.07E-17	5.300001274	4.16394696	-0.99931534	-1.133744865	1.658245749
ENSG00000250361	GYPB	glycophorin B (MNS blood group) [Source:HGNC Symbol;Acc:4703]	-5.478663777	3.436025336	78.81545105	6.82E-19	8.26E-17	4.160023991	4.952785634	-2.287883461	-4.730697034	2.216526545
ENSG00000211965	IGHV3-49	immunoglobulin heavy variable 3-49 [Source:HGNC Symbol;Acc:5607]	-5.406807826	5.279174037	78.74039907	7.08E-19	8.52E-17	6.862315351	5.784953762	1.432844522	-1.231517591	4.267798636
ENSG00000270999	CH17-132F21.1	Uncharacterized protein [Source:UniProtKB/TrEMBL;Acc:S4R394]	-5.219962285	3.449506303	78.474353	8.10E-19	9.68E-17	5.062133335	3.966439161	-0.907612816	-2.0191864	2.174777591
ENSG00000110693	SOX6	SRY (sex determining region Y)-box 6 [Source:HGNC Symbol;Acc:16421]	-5.176951181	2.562168712	78.32931812	8.72E-19	1.03E-16	3.420029142	3.847931966	-2.539967122	-2.670141381	1.908000984
ENSG00000115461	IGFBP5	insulin-like growth factor binding protein 5 [Source:HGNC Symbol;Acc:5474]	-11.02378861	2.349139128	78.27450522	8.97E-19	1.06E-16	3.753783962	3.480241792	-7.002070102	-7.002070102	-1.083770229
ENSG00000117399	CDC20	cell division cycle 20 [Source:HGNC Symbol;Acc:1723]	-4.307411958	3.131239647	77.67350751	1.22E-18	1.42E-16	4.30262994	4.088156756	-0.99931534	-0.797916568	2.504656975
ENSG00000226650	KIF4B	kinesin family member 4B [Source:HGNC Symbol;Acc:6322]	-4.236450677	3.17778869	77.44751912	1.36E-18	1.58E-16	4.055540716	4.306627984	-0.907612816	-0.655391447	2.874258264
ENSG00000183010	PYCR1	pyrroline-5-carboxylate reductase 1 [Source:HGNC Symbol;Acc:9721]	-3.987888141	3.709212595	77.3679767	1.42E-18	1.64E-16	4.970796966	4.578250478	0.125863448	0.114810932	2.938038295
ENSG00000239975	IGKV1D-33	immunoglobulin kappa variable 1D-33 [Source:HGNC Symbol;Acc:5753]	-5.838861924	3.452612906	77.29574011	1.47E-18	1.69E-16	5.120404941	3.82768418	-1.57307192	-3.360122781	2.334979851
ENSG00000211649	IGLV7-46	immunoglobulin lambda variable 7-46 (gene/pseudogene) [Source:HGNC Symbol;Acc:5930]	-4.637468367	4.004604459	77.01438853	1.70E-18	1.93E-16	5.345026248	4.409466019	0.28980717	-1.133744865	4.036790502
ENSG00000211671	IGLV2-8	immunoglobulin lambda variable 2-8 [Source:HGNC Symbol;Acc:5895]	-4.943398653	5.572086379	76.94544842	1.76E-18	1.98E-16	6.951243297	6.026161252	2.039642018	-0.244792505	5.450306193
ENSG00000112742	TTK	TTK protein kinase [Source:HGNC Symbol;Acc:12401]	-3.777560742	3.760504143	76.95601946	1.75E-18	1.98E-16	4.697484895	4.751005954	0.471574783	0.114810932	3.597502649
ENSG00000243466	IGKV1-5	immunoglobulin kappa variable 1-5 [Source:HGNC Symbol;Acc:5741]	-5.715470218	8.08357503	76.74053002	1.95E-18	2.18E-16	9.57711032	8.704662516	3.836777169	1.65228493	7.299817524

ENSG00000211898	IGHD	immunoglobulin heavy constant delta [Source:HGNC Symbol;Acc:5480]	-4.119964063	8.054952954	76.67091816	2.02E-18	2.24E-16	9.422431618	8.812545546	5.400059163	4.795946491	6.949484052
ENSG00000004809	SLC22A16	solute carrier family 22 (organic cation/carnitine transporter), member 16 [Source:HGNC Symbol;Acc:20302]	-4.091244638	3.443735944	76.48178553	2.22E-18	2.45E-16	4.335038744	4.56782473	-0.520331194	-1.231517591	3.178820438
ENSG00000165480	SKA3	spindle and kinetochore associated complex subunit 3 [Source:HGNC Symbol;Acc:20262]	-4.108687942	3.413423813	75.87677567	3.02E-18	3.31E-16	4.537594837	4.327378419	-0.907612816	-0.7248947	3.091765221
ENSG00000109805	NCAPG	non-SMC condensin I complex, subunit G [Source:HGNC Symbol;Acc:24304]	-3.858946463	3.705209931	75.64528275	3.39E-18	3.70E-16	4.644901642	4.588601424	0.250534523	-0.350682213	3.79299123
ENSG00000166803	KIAA0101	KIAA0101 [Source:HGNC Symbol;Acc:28961]	-4.120255942	3.729358726	75.61519008	3.45E-18	3.74E-16	4.954499232	4.472303462	-0.390491059	-0.244792505	3.486703703
ENSG00000156970	BUB1B	BUB1 mitotic checkpoint serine/threonine kinase B [Source:HGNC Symbol;Acc:1149]	-3.73691492	3.900385844	75.56401733	3.54E-18	3.81E-16	4.921340809	4.755606525	0.28980717	0.301169826	3.866951213
ENSG00000211659	IGLV3-25	immunoglobulin lambda variable 3-25 [Source:HGNC Symbol;Acc:5908]	-5.710932147	6.051161682	75.12981436	4.41E-18	4.72E-16	7.571336458	6.612968907	1.944409617	-1.133744865	5.281096681
ENSG00000116678	LEPR	leptin receptor [Source:HGNC Symbol;Acc:6554]	-3.161435856	5.258968255	74.69046821	5.51E-18	5.86E-16	6.410444715	6.186373911	3.049757403	3.188355007	3.986396982
ENSG00000188672	RHCE	Rh blood group, CcEe antigens [Source:HGNC Symbol;Acc:10008]	-4.661535175	3.339463343	74.37374222	6.46E-18	6.84E-16	4.055540716	4.875989911	-1.886652327	-2.0191864	1.895203721
ENSG00000136929	HEMGN	hemogen [Source:HGNC Symbol;Acc:17509]	-3.692081655	5.878024375	74.21575578	7.00E-18	7.36E-16	6.370240951	7.286659928	2.723873993	3.20246409	5.286007005
ENSG00000211966	IGHV5-51	immunoglobulin heavy variable 5-51 [Source:HGNC Symbol;Acc:5659]	-4.818048969	6.999916879	73.98915414	7.85E-18	8.21E-16	8.472146307	7.330746799	3.455848046	1.75796486	6.77142346
ENSG00000183032	SLC25A21	solute carrier family 25 (mitochondrial oxoadipate carrier), member 21 [Source:HGNC Symbol;Acc:14411]	-7.199482952	2.710294773	73.84732851	8.44E-18	8.77E-16	4.030315369	3.703019536	-4.621297774	-7.002070102	1.580441325
ENSG00000211949	IGHV3-23	immunoglobulin heavy variable 3-23 [Source:HGNC Symbol;Acc:5588]	-5.04677718	8.134436961	73.80266673	8.63E-18	8.91E-16	9.564779385	8.62801567	4.331420098	2.684399171	7.815127716
ENSG00000130821	SLC6A8	solute carrier family 6 (neurotransmitter transporter), member 8 [Source:HGNC Symbol;Acc:11055]	-3.775823845	4.428707972	73.56214572	9.75E-18	1.00E-15	5.427851864	5.557084059	0.9100098	1.832435869	3.557451401
ENSG00000241755	IGKV1-9	immunoglobulin kappa variable 1-9 [Source:HGNC Symbol;Acc:5744]	-6.272412205	6.439612109	73.41129347	1.05E-17	1.07E-15	8.034082604	6.938081855	1.855590689	-1.572251189	5.492337715
ENSG00000211673	IGLV3-1	immunoglobulin lambda variable 3-1 [Source:HGNC Symbol;Acc:5896]	-5.078496572	6.719504029	73.27367447	1.13E-17	1.15E-15	8.179332659	7.233508249	3.138485523	0.940331404	6.239154116
ENSG00000129173	E2F8	E2F transcription factor 8 [Source:HGNC Symbol;Acc:24727]	-3.930605927	3.480680248	73.22498634	1.16E-17	1.17E-15	4.443058246	4.314964006	-0.453950945	-0.589083246	3.640311645
ENSG00000041982	TNC	tenascin C [Source:HGNC Symbol;Acc:5318]	-6.398975631	2.883639109	72.46298003	1.70E-17	1.71E-15	4.344166237	3.942428021	-4.621297774	-4.730697034	-0.316136847
ENSG00000211639	IGLV4-60	immunoglobulin lambda variable 4-60 [Source:HGNC Symbol;Acc:5920]	-5.365231655	3.591154184	72.41880469	1.74E-17	1.74E-15	5.3897990814	3.955816848	-1.438615935	-1.336401299	0.819133578
ENSG00000211973	IGHV1-69	immunoglobulin heavy variable 1-69 [Source:HGNC Symbol;Acc:5558]	-5.376154356	7.390931289	72.33349479	1.82E-17	1.80E-15	8.734980269	7.848276278	3.425312536	1.109604583	7.405122753
ENSG00000143776	CDC42BPA	CDC42 binding protein kinase alpha (DMPK-like) [Source:HGNC Symbol;Acc:1737]	-3.694841187	4.355587798	71.92078622	2.24E-17	2.21E-15	5.445982527	5.266068042	0.858697288	1.808036846	3.873490568
ENSG00000211611	IGKV6-21	immunoglobulin kappa variable 6-21 (non-functional) [Source:HGNC Symbol;Acc:5836]	-5.658463285	3.183574707	71.88320432	2.28E-17	2.24E-15	4.758496361	3.480241792	-2.287883461	-2.670141381	2.71589062
ENSG00000137807	KIF23	kinesin family member 23 [Source:HGNC Symbol;Acc:6392]	-3.487381444	4.4309585	71.63764911	2.59E-17	2.52E-15	5.382056609	5.299008392	1.098750223	1.493047208	4.427417733
ENSG00000239855	IGKV1-6	immunoglobulin kappa variable 1-6 [Source:HGNC Symbol;Acc:5742]	-4.939961212	5.320731552	71.39431041	2.93E-17	2.84E-15	6.675175993	5.522410173	1.644577544	-0.589083246	5.573900552
ENSG00000140534	TICRR	TOPBP1-interacting checkpoint and replication regulator [Source:HGNC Symbol;Acc:28704]	-4.174700185	3.299704255	71.210797	3.21E-17	3.10E-15	4.185638714	4.262055685	-1.721358966	-1.336401299	3.439004866
ENSG00000211941	IGHV3-11	immunoglobulin heavy variable 3-11 (gene/pseudogene) [Source:HGNC Symbol;Acc:5580]	-5.192848201	5.915674801	71.17597499	3.27E-17	3.13E-15	7.416881779	6.607041069	2.330744516	-0.406691783	4.775228946
ENSG00000157456	CCNB2	cyclin B2 [Source:HGNC Symbol;Acc:1580]	-4.092838951	3.607129055	71.09885422	3.40E-17	3.24E-15	4.800548473	4.537869269	-1.202308962	0.074465887	3.005085735
ENSG00000163808	KIF15	kinesin family member 15 [Source:HGNC Symbol;Acc:17273]	-3.51762656	3.888333422	70.82549503	3.90E-17	3.70E-15	4.70812895	4.821448519	0.471574783	0.696304622	4.028024474
ENSG00000223350	IGLV9-49	immunoglobulin lambda variable 9-49 [Source:HGNC Symbol;Acc:5933]	-4.660117087	3.671580682	70.1434988	5.51E-17	5.20E-15	5.276354728	4.374001309	0.250534523	-1.449512048	1.239342106
ENSG00000211942	IGHV3-13	immunoglobulin heavy variable 3-13 [Source:HGNC Symbol;Acc:5581]	-4.877779059	3.508409608	70.01954902	5.87E-17	5.51E-15	4.970796966	4.182288334	-0.10933503	-2.670141381	2.586540774
ENSG00000250722	SEPP1	selenoprotein P, plasma, 1 [Source:HGNC Symbol;Acc:10751]	-4.954176215	3.489042959	69.9940387	5.95E-17	5.55E-15	5.039835608	4.131277536	-2.539967122	-1.70641174	2.306265771
ENSG00000239951	IGKV3-20	immunoglobulin kappa variable 3-20 [Source:HGNC Symbol;Acc:5817]	-5.312814341	8.288330083	69.86450806	6.35E-17	5.90E-15	9.71860995	8.754162584	4.307700297	2.371259556	8.04214606
ENSG00000105173	CCNE1	cyclin E1 [Source:HGNC Symbol;Acc:1589]	-3.361500366	4.044085069	69.41254571	7.99E-17	7.38E-15	5.120404941	5.073157632	1.32354911	1.36509501	3.108493252

ENSG00000187266	EPOR	erythropoietin receptor [Source:HGNC Symbol;Acc:3416]	-2.877568474	5.181606856	69.11804074	9.27E-17	8.52E-15	6.034740035	6.22282493	3.262115839	3.197776377	4.4668719
ENSG00000244575	IGKV1-27	immunoglobulin kappa variable 1-27 [Source:HGNC Symbol;Acc:5735]	-4.739444231	3.969341019	69.03968856	9.65E-17	8.82E-15	5.391992993	4.078396779	-0.010608983	-1.042179808	4.146151637
ENSG00000198959	TGM2	transglutaminase 2 [Source:HGNC Symbol;Acc:11778]	-3.194087386	4.530937942	68.83127756	1.07E-16	9.76E-15	5.67030504	5.548272454	1.932051102	2.126858114	3.225574983
ENSG00000224041	IGKV3D-15	immunoglobulin kappa variable 3D-15 (gene/pseudogene) [Source:HGNC Symbol;Acc:5824]	-4.778620626	3.590085018	68.52728181	1.25E-16	1.13E-14	4.938015281	3.768216544	-0.907612816	-1.231517591	3.880000416
ENSG00000051341	POLQ	polymerase (DNA directed), theta [Source:HGNC Symbol;Acc:9186]	-3.490746768	3.858543033	68.34519624	1.37E-16	1.24E-14	4.675958256	4.781402895	0.365283811	0.669468625	4.025090588
ENSG00000166851	PLK1	polo-like kinase 1 [Source:HGNC Symbol;Acc:9077]	-3.388061065	4.19332936	67.62361938	1.98E-16	1.77E-14	5.291768997	4.976626419	1.265630948	1.462107232	3.940421563
ENSG00000094804	CDC6	cell division cycle 6 [Source:HGNC Symbol;Acc:1744]	-3.582669956	3.742227221	67.58227008	2.02E-16	1.80E-14	4.688554464	4.686615325	0.328039018	0.58582888	3.621010335
ENSG00000184792	OSBP2	oxysterol binding protein 2 [Source:HGNC Symbol;Acc:8504]	-3.130405298	4.987369651	67.01659442	2.69E-16	2.39E-14	5.654799334	6.216720676	3.072456242	2.303059008	4.33330585
ENSG00000066926	FECH	ferrochelatase [Source:HGNC Symbol;Acc:3647]	-2.720528273	5.941993225	66.96998791	2.76E-16	2.43E-14	6.733649303	6.911873658	4.190675219	3.81483761	5.581908675
ENSG00000211936	IGHV4-4	immunoglobulin heavy variable 4-4 [Source:HGNC Symbol;Acc:5652]	-5.409826524	6.213570183	66.9479529	2.79E-16	2.45E-14	7.700440885	6.862967281	2.430758609	-0.525689211	5.331839976
ENSG00000174371	EXO1	exonuclease 1 [Source:HGNC Symbol;Acc:3511]	-3.702725736	3.436670808	66.85354851	2.92E-16	2.55E-14	4.567129289	4.38195804	-0.161352853	-0.244792505	2.912863619
ENSG00000165449	SLC16A9	solute carrier family 16, member 9 [Source:HGNC Symbol;Acc:23520]	-7.014093348	2.769762741	66.66479453	3.22E-16	2.80E-14	4.180552023	3.66113137	-7.002070102	-4.730697034	1.564363944
ENSG00000024526	DEPDC1	DEP domain containing 1 [Source:HGNC Symbol;Acc:22949]	-3.882726041	3.297394007	66.29432302	3.88E-16	3.36E-14	4.425965607	4.121805905	-0.589913612	-0.525689211	3.102938765
ENSG00000143416	SELENBP1	selenium binding protein 1 [Source:HGNC Symbol;Acc:10719]	-5.137210175	5.291106269	65.99610991	4.52E-16	3.89E-14	6.14852261	6.623913545	1.980862385	-2.419096061	4.44068937
ENSG00000167363	FN3K	fructosamine 3 kinase [Source:HGNC Symbol;Acc:24822]	-3.255482512	4.028285806	65.96328478	4.59E-16	3.93E-14	5.103034834	5.138021083	1.815785154	1.0274492	2.610226292
ENSG00000139734	DIAPH3	diaphanous-related formin 3 [Source:HGNC Symbol;Acc:15480]	-4.20583674	2.640778252	65.83420868	4.90E-16	4.18E-14	3.567603998	3.674150053	-1.57307192	-1.449512048	2.470556253
ENSG00000075218	GTSE1	G-2 and S-phase expressed 1 [Source:HGNC Symbol;Acc:13698]	-3.584876813	3.638194158	65.56838713	5.61E-16	4.76E-14	4.586488284	4.499985557	-0.161352853	0.192266291	3.729524826
ENSG00000211653	IGLV1-40	immunoglobulin lambda variable 1-40 [Source:HGNC Symbol;Acc:5877]	-5.029898657	6.646511673	65.4423906	5.98E-16	5.05E-14	7.968086305	7.212746789	3.061151464	0.466186565	6.526330041
ENSG00000211650	IGLV5-45	immunoglobulin lambda variable 5-45 [Source:HGNC Symbol;Acc:5924]	-4.887996513	4.189851254	64.73293298	8.58E-16	7.20E-14	5.836369856	4.741760566	0.805492218	-1.70641174	2.32547185
ENSG00000242580	IGKV1D-43	immunoglobulin kappa variable 1D-43 [Source:HGNC Symbol;Acc:5758]	-4.951977219	3.29472274	64.69364918	8.75E-16	7.28E-14	4.961930124	4.005590473	-0.271377577	-3.360122781	-0.726511138
ENSG00000211970	IGHV4-61	immunoglobulin heavy variable 4-61 [Source:HGNC Symbol;Acc:5655]	-4.703876609	4.904395558	64.70279723	8.71E-16	7.28E-14	6.31037526	5.65308557	1.774850102	-1.042179808	3.986396982
ENSG00000100479	POLE2	polymerase (DNA directed), epsilon 2, accessory subunit [Source:HGNC Symbol;Acc:9178]	-3.642152175	3.490822414	64.17213181	1.14E-15	9.44E-14	4.718695048	4.387896921	0.28980717	-0.146146183	2.701261324
ENSG00000211972	IGHV3-66	immunoglobulin heavy variable 3-66 [Source:HGNC Symbol;Acc:5619]	-5.059742633	3.742778315	63.89809136	1.31E-15	1.08E-13	5.26918452	4.204891931	-0.010608983	-3.888428289	3.102938765
ENSG00000170476	MZB1	marginal zone B and B1 cell-specific protein [Source:HGNC Symbol;Acc:30125]	-3.703517412	5.454824835	63.76069354	1.40E-15	1.15E-13	6.69225353	5.920186567	3.083673126	1.856429106	5.424931915
ENSG00000211939	IGHV1-8	immunoglobulin heavy variable 1-8 [Source:HGNC Symbol;Acc:5559]	-5.069216122	4.00820015	63.57774562	1.54E-15	1.26E-13	5.658462756	4.766284479	0.570569237	-3.888428289	-0.201737898
ENSG00000164045	CDC25A	cell division cycle 25A [Source:HGNC Symbol;Acc:1725]	-3.573779628	3.56553146	63.55148338	1.56E-15	1.27E-13	4.702816739	4.363993286	0.250534523	0.03296005	3.32911422
ENSG00000211652	IGLV7-43	immunoglobulin lambda variable 7-43 [Source:HGNC Symbol;Acc:5929]	-4.563831226	4.111284162	63.27350531	1.80E-15	1.46E-13	5.581437803	4.624255851	0.858697288	-1.449512048	3.512071509
ENSG00000211666	IGLV2-14	immunoglobulin lambda variable 2-14 [Source:HGNC Symbol;Acc:5888]	-4.931356822	8.037644085	62.90733159	2.17E-15	1.74E-13	9.561725963	8.782240931	4.569443612	2.483362921	6.462700312
ENSG00000066735	KIF26A	kinesin family member 26A [Source:HGNC Symbol;Acc:20226]	-3.748897206	3.108000808	62.31670653	2.92E-15	2.34E-13	4.144433979	4.434547869	-0.589913612	-0.956081001	1.177268129
ENSG00000119888	EPCAM	epithelial cell adhesion molecule [Source:HGNC Symbol;Acc:11529]	-5.372084099	2.838662408	62.02659862	3.39E-15	2.70E-13	4.271869887	3.740634939	-3.766908349	-3.888428289	1.3928929
ENSG00000160117	ANKLE1	ankyrin repeat and LEM domain containing 1 [Source:HGNC Symbol;Acc:26812]	-2.899779006	4.634937862	62.01201488	3.41E-15	2.71E-13	5.507195663	5.649794134	2.340128686	2.730566978	4.045503588
ENSG00000111665	CDCA3	cell division cycle associated 3 [Source:HGNC Symbol;Acc:14624]	-3.434174198	3.620484939	61.76891041	3.86E-15	3.05E-13	4.665073276	4.347834625	0.471574783	0.74852395	3.63262208
ENSG00000047597	XK	X-linked Kx blood group (McLeod syndrome) [Source:HGNC Symbol;Acc:12811]	-2.96661067	4.951623369	61.51869573	4.39E-15	3.45E-13	5.822558176	6.045075729	2.456873398	2.842914522	4.255334356

ENSG00000197465	GYPE	glycophorin E (MNS blood group) [Source:HGNC Symbol;Acc:4705]	-3.650435452	3.673154665	61.43002923	4.59E-15	3.60E-13	4.572964312	4.896964317	1.030832979	-0.655391447	2.841278738
ENSG00000162692	VCAM1	vascular cell adhesion molecule 1 [Source:HGNC Symbol;Acc:12663]	-4.641977924	2.651178911	61.24857966	5.03E-15	3.92E-13	4.069366531	3.703019536	-2.845623393	-2.670141381	-0.045536284
ENSG00000196517	SLC6A9	solute carrier family 6 (neurotransmitter transporter, glycine), member 9 [Source:HGNC Symbol;Acc:11056]	-4.155260927	3.08790157	61.17042774	5.23E-15	4.07E-13	3.927796389	4.333545794	-1.886652327	-2.205328685	2.656461736
ENSG00000162599	NFIA	nuclear factor I/A [Source:HGNC Symbol;Acc:7784]	-3.006135664	4.15222904	61.15532206	5.27E-15	4.08E-13	5.063515567	5.139195178	1.968813513	2.106994346	3.545215952
ENSG00000146670	CDCA5	cell division cycle associated 5 [Source:HGNC Symbol;Acc:14626]	-3.249311236	4.189460774	60.99151721	5.73E-15	4.41E-13	5.145421644	5.021885183	1.053829154	1.582074621	4.167595364
ENSG00000005381	MPO	myeloperoxidase [Source:HGNC Symbol;Acc:7218]	-5.554959054	8.964952798	60.98191024	5.76E-15	4.42E-13	9.037933614	9.543010449	2.448220886	4.434820745	10.22833208
ENSG00000137812	CASC5	cancer susceptibility candidate 5 [Source:HGNC Symbol;Acc:24054]	-3.029452879	4.33845145	60.89250426	6.03E-15	4.60E-13	5.051027549	5.287405779	1.534439871	1.844482366	4.46903248
ENSG00000198554	WDHD1	WD repeat and HMG-box DNA binding protein 1 [Source:HGNC Symbol;Acc:23170]	-3.021080812	4.120209641	60.69326871	6.67E-15	5.07E-13	5.041239358	5.053365096	1.689321579	1.462107232	3.860382081
ENSG00000163864	NMNAT3	nicotinamide nucleotide adenylyltransferase 3 [Source:HGNC Symbol;Acc:20989]	-3.2787839	3.737453118	60.27945342	8.23E-15	6.23E-13	4.865381865	4.673709144	1.007464306	0.894716898	2.968901188
ENSG00000171401	KRT13	keratin 13 [Source:HGNC Symbol;Acc:6415]	-10.6818494	2.13415043	60.25418027	8.34E-15	6.28E-13	3.495702697	3.189723558	-7.002070102	-7.002070102	0.264147325
ENSG00000107130	NCS1	neuronal calcium sensor 1 [Source:HGNC Symbol;Acc:3953]	-3.631692787	3.189556674	60.08803467	9.07E-15	6.81E-13	4.373437975	4.270652493	-0.329705368	-0.797916568	1.958084208
ENSG00000211668	IGLV2-11	immunoglobulin lambda variable 2-11 [Source:HGNC Symbol;Acc:5887]	-4.826645914	6.210595985	59.9575024	9.69E-15	7.24E-13	7.700885048	6.902915513	3.015025135	0.369457657	5.039358506
ENSG00000035499	DEPDC1B	DEP domain containing 1B [Source:HGNC Symbol;Acc:24902]	-3.410133839	3.543300162	59.57188229	1.18E-14	8.77E-13	4.592245686	4.33149293	0.28980717	0.192266291	3.507874397
ENSG00000130203	APOE	apolipoprotein E [Source:HGNC Symbol;Acc:613]	-4.724536459	2.746800647	59.4923256	1.23E-14	9.09E-13	4.325853136	3.340242669	-1.721358966	-2.670141381	1.3928929
ENSG00000101003	GINS1	GINS complex subunit 1 (Psf1 homolog) [Source:HGNC Symbol;Acc:28980]	-3.331504179	3.519524893	58.84663728	1.70E-14	1.26E-12	4.52360241	4.419164558	0.401591237	0.301169826	3.333864066
ENSG00000130208	APOC1	apolipoprotein C-I [Source:HGNC Symbol;Acc:607]	-5.632786663	2.64435536	58.80114205	1.74E-14	1.28E-12	4.240439676	3.171477064	-4.621297774	-3.360122781	1.548105377
ENSG00000101447	FAM83D	family with sequence similarity 83, member D [Source:HGNC Symbol;Acc:16122]	-3.412905372	3.35823291	58.67284606	1.86E-14	1.36E-12	4.457849968	4.272793714	0.365283811	-0.146146183	2.900109607
ENSG00000164692	COL1A2	collagen, type I, alpha 2 [Source:HGNC Symbol;Acc:2198]	-10.84915097	2.280112664	58.6173852	1.92E-14	1.40E-12	3.64719635	3.356486108	-7.002070102	-7.002070102	0.223773082
ENSG00000140525	FANCI	Fanconi anemia, complementation group I [Source:HGNC Symbol;Acc:25568]	-2.579641215	5.476841057	58.45562772	2.08E-14	1.51E-12	6.250953752	6.284611391	3.686701825	3.427354029	5.479534875
ENSG00000240834	IGKV1D-12	immunoglobulin kappa variable 1D-12 [Source:HGNC Symbol;Acc:5746]	-4.931587071	3.590354752	57.91032266	2.74E-14	1.98E-12	5.251101524	4.200399454	0.125863448	-7.002070102	1.239342106
ENSG00000168496	FEN1	flap structure-specific endonuclease 1 [Source:HGNC Symbol;Acc:3650]	-2.699737199	5.087522251	57.78305927	2.93E-14	2.11E-12	6.019864886	5.89816312	3.083673126	3.415289988	4.701801286
ENSG00000211952	IGHV4-28	immunoglobulin heavy variable 4-28 [Source:HGNC Symbol;Acc:5645]	-4.496905512	2.851522679	57.10284055	4.14E-14	2.96E-12	4.175447334	3.356486108	-0.907612816	-3.888428289	2.786891356
ENSG00000072274	TFRC	transferrin receptor [Source:HGNC Symbol;Acc:11763]	-3.686534192	9.025753789	57.06943081	4.21E-14	3.00E-12	9.881803674	10.27208667	6.307273993	6.411838892	8.030306792
ENSG00000116675	DNAJC6	DnaJ (Hsp40) homolog, subfamily C, member 6 [Source:HGNC Symbol;Acc:15469]	-3.329805294	3.701810891	56.9747718	4.41E-14	3.14E-12	4.732663871	4.769320864	0.437007322	1.148991974	2.867722395
ENSG00000161888	SPC24	SPC24, NDC80 kinetochore complex component [Source:HGNC Symbol;Acc:26913]	-3.51323041	3.204608382	56.81337688	4.79E-14	3.39E-12	4.245319886	4.010731282	-0.589913612	-0.525689211	3.225574983
ENSG00000093009	CDC45	cell division cycle 45 [Source:HGNC Symbol;Acc:1739]	-3.362440998	3.319353069	56.65155259	5.20E-14	3.67E-12	4.245319886	4.238146865	-0.329705368	0.074465887	3.309956893
ENSG00000211896	IGHG1	immunoglobulin heavy constant gamma 1 (G1m marker) [Source:HGNC Symbol;Acc:5525]	-6.159547317	11.45461382	56.16685901	6.66E-14	4.67E-12	12.97786904	12.12923501	6.448999297	4.766175279	10.4210557
ENSG00000118193	KIF14	kinesin family member 14 [Source:HGNC Symbol;Acc:19181]	-3.388987205	3.26509064	55.85888204	7.79E-14	5.45E-12	4.141819186	4.073491918	-0.271377577	-0.350682213	3.545215952
ENSG00000174951	FUT1	fucosyltransferase 1 (galactoside 2-alpha-L-fucosyltransferase, H blood group) [Source:HGNC Symbol;Acc:4012]	-4.691687598	2.414470369	55.04514069	1.18E-13	8.21E-12	3.567603998	3.431239168	-2.845623393	-3.888428289	1.802296017
ENSG00000151474	FRMD4A	FERM domain containing 4A [Source:HGNC Symbol;Acc:25491]	-4.360736902	2.068470124	54.86303876	1.29E-13	8.97E-12	2.907138859	3.311369006	-2.845623393	-1.70641174	1.463945021
ENSG00000099958	DERL3	derlin 3 [Source:HGNC Symbol;Acc:14236]	-2.967500051	4.698454951	54.72294432	1.39E-13	9.59E-12	5.873880975	5.20794766	2.766259878	2.346060651	4.46254101
ENSG00000184254	ALDH1A3	aldehyde dehydrogenase 1 family, member A3 [Source:HGNC Symbol;Acc:409]	-9.851096383	1.240962258	54.58012393	1.49E-13	1.03E-11	2.778381342	2.154261589	-7.002070102	-7.002070102	-2.526883755

ENSG00000149554	CHEK1	checkpoint kinase 1 [Source:HGNC Symbol;Acc:1925]	-3.080217712	3.849978396	54.12887324	1.88E-13	1.29E-11	4.773776708	4.665583755	0.934997507	1.069111613	3.912116199
ENSG00000184661	CDCA2	cell division cycle associated 2 [Source:HGNC Symbol;Acc:14623]	-3.405680832	3.086781931	54.02792251	1.98E-13	1.35E-11	3.993081991	4.061156311	-0.390491059	-0.525689211	3.016948344
ENSG00000222037	IGLC6	immunoglobulin lambda constant 6 (Kern+Oz- marker, gene/pseudogene) [Source:HGNC Symbol;Acc:5860]	-4.372640559	3.690782499	53.91283098	2.10E-13	1.43E-11	5.191664033	4.220506339	0.632982468	-1.854339469	2.861156782
ENSG00000034063	UHRF1	ubiquitin-like with PHD and ring finger domains 1 [Source:HGNC Symbol;Acc:12556]	-3.190015496	3.615842447	53.5756173	2.49E-13	1.69E-11	4.007516463	4.451660263	-0.161352853	0.301169826	4.386854181
ENSG00000211961	IGHV1-45	immunoglobulin heavy variable 1-45 [Source:HGNC Symbol;Acc:5553]	-5.027847576	2.128974441	53.09310262	3.18E-13	2.15E-11	3.687198663	2.932364153	-2.539967122	-4.730697034	-0.257803757
ENSG00000211658	IGLV3-27	immunoglobulin lambda variable 3-27 [Source:HGNC Symbol;Acc:5910]	-4.384857171	2.91438649	52.84348036	3.61E-13	2.43E-11	4.215787888	3.134276817	-0.907612816	-2.97427061	3.235762277
ENSG00000211679	IGLC3	immunoglobulin lambda constant 3 (Kern-Oz+ marker) [Source:HGNC Symbol;Acc:5857]	-5.015447672	8.775536552	52.60456281	4.08E-13	2.73E-11	10.21383059	9.419561065	5.213528089	3.017993511	8.093989886
ENSG00000132646	PCNA	proliferating cell nuclear antigen [Source:HGNC Symbol;Acc:8729]	-2.458082023	5.285409846	52.17754038	5.07E-13	3.38E-11	6.137408525	5.992127367	3.683019014	3.349187152	5.233595656
ENSG00000178999	AURKB	aurora kinase B [Source:HGNC Symbol;Acc:11390]	-3.23661313	3.679230864	52.01539017	5.51E-13	3.66E-11	4.725696366	4.453549171	0.570569237	1.314027603	3.512071509
ENSG00000149516	MS4A3	membrane-spanning 4-domains, subfamily A, member 3 (hematopoietic cell-specific) [Source:HGNC Symbol;Acc:7317]	-3.492812588	4.535978781	51.70808698	6.44E-13	4.27E-11	4.865381865	4.505458803	-0.740035668	1.069111613	5.935971906
ENSG00000171914	TLN2	talin 2 [Source:HGNC Symbol;Acc:15447]	-3.262488331	2.880768372	51.659237	6.60E-13	4.36E-11	3.697918598	3.93973528	0.036330944	0.114810932	2.602374247
ENSG00000211946	IGHV3-20	immunoglobulin heavy variable 3-20 [Source:HGNC Symbol;Acc:5585]	-4.438603321	2.648252902	51.56892449	6.91E-13	4.55E-11	4.112739262	2.959203556	-1.202308962	-2.670141381	2.435629945
ENSG00000112759	SLC29A1	solute carrier family 29 (equilibrative nucleoside transporter), member 1 [Source:HGNC Symbol;Acc:11003]	-2.463738764	5.716084308	51.49920034	7.16E-13	4.69E-11	6.654687631	6.601088773	3.993665308	4.238831357	4.900580057
ENSG00000184785	SMIM10	small integral membrane protein 10 [Source:HGNC Symbol;Acc:41913]	-3.685432913	2.761904977	51.21347231	8.28E-13	5.41E-11	3.881526851	3.566554811	-1.315629595	-0.350682213	2.546179737
ENSG00000164626	KCNK5	potassium channel, subfamily K, member 5 [Source:HGNC Symbol;Acc:6280]	-3.465391165	3.056494479	51.19330573	8.37E-13	5.44E-11	4.33045325	3.881994713	-0.66302287	-0.350682213	2.32547185
ENSG00000168477	TNXB	tenascin XB [Source:HGNC Symbol;Acc:11976]	-2.993975079	4.380004099	50.99949164	9.24E-13	5.98E-11	4.989823523	5.581474026	2.448220886	1.206127397	4.030952405
ENSG00000254395	IGHV4-55	immunoglobulin heavy variable 4-55 (pseudogene) [Source:HGNC Symbol;Acc:5653]	-4.751829038	2.578577501	50.98191756	9.32E-13	6.02E-11	4.007516463	2.932364153	-1.721358966	-4.730697034	2.470556253
ENSG00000187955	COL14A1	collagen, type XIV, alpha 1 [Source:HGNC Symbol;Acc:2191]	-4.552069005	2.184834579	50.67125364	1.09E-12	7.02E-11	3.628640226	3.216667762	-3.233942493	-2.97427061	-0.9858258
ENSG00000211677	IGLC2	immunoglobulin lambda constant 2 (Kern-Oz-marker) [Source:HGNC Symbol;Acc:5856]	-5.089593407	8.113483714	50.57244165	1.15E-12	7.36E-11	9.578621645	8.737882838	4.502066997	1.745170531	7.382663412
ENSG00000126088	UROD	uroporphyrinogen decarboxylase [Source:HGNC Symbol;Acc:12591]	-2.41878177	6.645339848	50.53955367	1.17E-12	7.46E-11	7.520157172	7.522451483	5.161544583	5.170781053	5.929710319
ENSG00000140403	DNAJA4	DnaJ (Hsp40) homolog, subfamily A, member 4 [Source:HGNC Symbol;Acc:14885]	-2.479186135	6.474040507	50.16420382	1.41E-12	8.99E-11	7.291553494	7.45498666	5.203280155	4.555153026	5.691442928
ENSG00000042062	FAM65C	family with sequence similarity 65, member C [Source:HGNC Symbol;Acc:16168]	-3.386113395	4.732973886	49.91897058	1.60E-12	1.02E-10	5.673024125	5.827089554	0.805492218	2.657338701	4.068484455
ENSG00000175449	RFESD	Rieske (Fe-S) domain containing [Source:HGNC Symbol;Acc:29587]	-3.105099528	3.430184676	49.65812065	1.83E-12	1.16E-10	4.484924323	4.527146478	1.285196495	-0.053815412	2.236956541
ENSG00000198826	ARHGAP11A	Rho GTPase activating protein 11A [Source:HGNC Symbol;Acc:15783]	-2.406845506	4.949287545	49.53169342	1.95E-12	1.23E-10	5.714967351	5.753866075	3.30587588	2.800257198	4.946271918
ENSG00000143797	MBOAT2	membrane bound O-acyltransferase domain containing 2 [Source:HGNC Symbol;Acc:25193]	-2.881539291	5.591373197	49.26957416	2.23E-12	1.40E-10	6.343181631	6.509633512	2.886421762	3.895212931	5.529043212
ENSG00000240671	IGKV1-8	immunoglobulin kappa variable 1-8 [Source:HGNC Symbol;Acc:5743]	-3.790429946	2.938046722	49.25871582	2.24E-12	1.40E-10	4.041581121	3.229953478	-0.271377577	-1.854339469	3.473850543
ENSG00000253655	IGJP1	immunoglobulin J polypeptide pseudogene 1 [Source:HGNC Symbol;Acc:5714]	-4.477407967	4.927007562	49.17398101	2.34E-12	1.46E-10	6.48763963	5.450457515	2.039642018	-0.956081001	3.779126901
ENSG00000211940	IGHV3-9	immunoglobulin heavy variable 3-9 [Source:HGNC Symbol;Acc:5628]	-5.894900553	5.945926912	49.15276843	2.37E-12	1.47E-10	7.64127178	6.697489003	2.004662384	-3.888428289	-1.3021922
ENSG00000134690	CDCA8	cell division cycle associated 8 [Source:HGNC Symbol;Acc:14629]	-2.997183338	3.674531095	49.11094995	2.42E-12	1.50E-10	4.681370103	4.48158997	0.934997507	1.12943269	3.516256447
ENSG00000211651	IGLV1-44	immunoglobulin lambda variable 1-44 [Source:HGNC Symbol;Acc:5879]	-5.202984192	6.871567366	49.00941927	2.55E-12	1.57E-10	8.208051042	7.410948087	3.262115839	-0.194626373	6.766161721

ENSG00000171320	ESCO2	establishment of sister chromatid cohesion N-acetyltransferase 2 [Source:HGNC Symbol;Acc:27230]	-3.152366257	3.194195304	48.99778333	2.56E-12	1.57E-10	4.154846016	4.105079432	-0.215316613	0.154058352	3.080504462
ENSG00000144485	HES6	hes family bHLH transcription factor 6 [Source:HGNC Symbol;Acc:18254]	-3.576612049	4.071085726	48.92086864	2.67E-12	1.63E-10	5.404045254	4.836002619	0.9100098	2.003353492	2.793802967
ENSG00000091513	TF	transferrin [Source:HGNC Symbol;Acc:11740]	-4.248961518	2.688963177	48.79379469	2.84E-12	1.73E-10	4.332747819	3.423550355	-2.539967122	-1.854339469	-0.726511138
ENSG00000184185	KCNJ12	potassium inwardly-rectifying channel, subfamily J, member 12 [Source:HGNC Symbol;Acc:6258]	-3.812435705	2.67376146	48.23754852	3.78E-12	2.29E-10	3.957838374	3.810099846	-3.233942493	-1.231517591	0.551948963
ENSG00000105205	CLC	Charcot-Leyden crystal galectin [Source:HGNC Symbol;Acc:2014]	-3.723052554	3.133710762	48.14982437	3.95E-12	2.39E-10	4.509472944	3.457831828	0.125863448	-0.655391447	3.02284327
ENSG00000143228	NUF2	NUF2, NDC80 kinetochore complex component [Source:HGNC Symbol;Acc:14621]	-2.935157602	3.677098625	48.01645622	4.23E-12	2.55E-10	4.661426622	4.395777591	1.285196495	0.798919003	3.715033827
ENSG00000137460	FHDC1	FH2 domain containing 1 [Source:HGNC Symbol;Acc:29363]	-3.334493673	3.797269233	47.64307252	5.11E-12	3.07E-10	4.466234785	5.002662158	-0.329705368	1.206127397	3.573605569
ENSG00000004939	SLC4A1	solute carrier family 4 (anion exchanger), member 1 (Diego blood group) [Source:HGNC Symbol;Acc:11027]	-5.898874378	7.368698236	47.57336609	5.30E-12	3.17E-10	7.60660933	8.990021765	3.46445507	-1.133744865	6.795727645
ENSG00000167815	PRDX2	peroxiredoxin 2 [Source:HGNC Symbol;Acc:9353]	-3.137875941	6.211486909	47.29242114	6.11E-12	3.65E-10	7.228257247	7.199863678	4.722595709	3.193073383	5.236135087
ENSG00000076003	MCM6	minichromosome maintenance complex component 6 [Source:HGNC Symbol;Acc:6949]	-2.391379723	6.022347552	47.25886463	6.22E-12	3.70E-10	6.811394892	6.731359671	4.652765747	3.857072211	6.055095074
ENSG00000175063	UBE2C	ubiquitin-conjugating enzyme E2C [Source:HGNC Symbol;Acc:15937]	-3.382187395	2.897876308	47.06630526	6.86E-12	4.07E-10	4.074859969	3.597635769	-0.740035668	0.192266291	2.648857997
ENSG00000164741	DLC1	deleted in liver cancer 1 [Source:HGNC Symbol;Acc:2897]	-3.023649787	3.046650007	47.04315913	6.94E-12	4.10E-10	4.052759582	4.036164092	0.505333341	0.669468625	2.31590077
ENSG00000211968	IGHV1-58	immunoglobulin heavy variable 1-58 [Source:HGNC Symbol;Acc:5555]	-4.319743339	2.250906933	46.97569572	7.19E-12	4.23E-10	3.559789652	2.632310473	-2.287883461	-2.670141381	2.372397358
ENSG00000077943	ITGA8	integrin, alpha 8 [Source:HGNC Symbol;Acc:6144]	-10.29327611	1.656659832	46.69972823	8.27E-12	4.86E-10	3.150283373	2.557000471	-7.002070102	-7.002070102	-0.57637152
ENSG00000115107	STEAP3	STEAP family member 3, metalloreductase [Source:HGNC Symbol;Acc:24592]	-2.743288408	5.133011584	46.67261866	8.39E-12	4.91E-10	5.897324146	6.182392918	2.580794782	3.43534104	4.705471168
ENSG00000073464	CLCN4	chloride channel, voltage-sensitive 4 [Source:HGNC Symbol;Acc:2022]	-2.614895366	4.344627941	46.35404793	9.87E-12	5.76E-10	5.136255356	5.402371952	2.096120255	2.572971709	3.761606998
ENSG00000164877	MICALL2	MICAL-like 2 [Source:HGNC Symbol;Acc:29672]	-2.599823257	4.196951389	46.15310455	1.09E-11	6.36E-10	5.015760478	5.173980935	2.016416749	2.156150191	3.850471957
ENSG00000087586	AURKA	aurora kinase A [Source:HGNC Symbol;Acc:11393]	-2.832053369	3.379718241	46.07469892	1.14E-11	6.59E-10	4.360001702	4.30453643	1.120696904	0.61425099	2.950462792
ENSG00000165304	MELK	maternal embryonic leucine zipper kinase [Source:HGNC Symbol;Acc:16870]	-3.060894767	3.051873322	46.01414056	1.17E-11	6.78E-10	3.930828895	3.912527782	0.168628767	-0.194626373	3.146787158
ENSG00000237810	CTD-2571E19.3		-3.620818073	2.262454821	45.91110113	1.24E-11	7.12E-10	3.411370615	3.081507914	-1.57307192	-1.336401299	1.945725455
ENSG00000100362	PVALB	parvalbumin [Source:HGNC Symbol;Acc:9704]	6.114606514	-0.57512767	45.52969637	1.50E-11	8.63E-10	-4.650190627	-4.777156855	-2.073364206	0.434658742	0.518960621
ENSG00000214009	PCNAP3	proliferating cell nuclear antigen pseudogene 3 [Source:HGNC Symbol;Acc:43736]	-2.3168236	4.875714686	45.47937385	1.54E-11	8.82E-10	5.66758082	5.679957148	3.271955645	2.756300061	4.806321398
ENSG00000117632	STMN1	stathmin 1 [Source:HGNC Symbol;Acc:6510]	-2.505893967	6.446667947	45.05885325	1.91E-11	1.09E-09	7.221772163	6.991063891	4.967809772	4.154255136	6.784928124
ENSG00000168268	NT5DC2	5'-nucleotidase domain containing 2 [Source:HGNC Symbol;Acc:25717]	-2.524239359	4.293413779	44.99979101	1.97E-11	1.12E-09	5.276354728	4.950112174	2.37706662	2.354509285	4.151542524
ENSG00000214049	UCA1	urothelial cancer associated 1 (non-protein coding) [Source:HGNC Symbol;Acc:37126]	-4.452189696	1.950941401	44.82091273	2.16E-11	1.22E-09	3.15547772	3.006291013	-4.621297774	-2.670141381	0.898226269
ENSG00000132465	IGJ	immunoglobulin J polypeptide, linker protein for immunoglobulin alpha and mu polypeptides [Source:HGNC Symbol;Acc:5713]	-4.804836014	7.929098313	44.79675252	2.19E-11	1.23E-09	9.434166502	8.528594032	5.085469664	2.175352965	6.892269576
ENSG00000213177	PRDX2P4	peroxiredoxin 2 pseudogene 4 [Source:HGNC Symbol;Acc:44969]	-2.910232029	5.724424493	44.74302193	2.25E-11	1.26E-09	6.69270022	6.788223555	4.148568005	2.528862927	4.730902235
ENSG00000215049	PRDX2P1	peroxiredoxin 2 pseudogene 1 [Source:HGNC Symbol;Acc:30020]	-2.846536747	4.663818747	44.73450769	2.26E-11	1.27E-09	5.69993678	5.640703774	3.061151464	1.538247531	3.733124947
ENSG00000151725	CENPU	centromere protein U [Source:HGNC Symbol;Acc:21348]	-2.880415495	3.809699403	44.45859719	2.60E-11	1.45E-09	4.843020085	4.670464478	1.053829154	1.493047208	3.425717652
ENSG00000256663	RP11-424C20.2		-3.219283089	3.427791987	44.23068018	2.92E-11	1.63E-09	3.897115495	4.211604524	-0.453950945	0.642123966	4.154230432
ENSG00000215580	BCORP1	BCL6 corepressor pseudogene 1 [Source:HGNC Symbol;Acc:23953]	9.626099804	1.412938588	44.08552609	3.14E-11	1.75E-09	-7.002070102	-7.002070102	-7.002070102	1.926103707	3.215315242
ENSG00000085999	RAD54L	RAD54-like (S. cerevisiae) [Source:HGNC Symbol;Acc:9826]	-3.099181079	2.950923015	44.02098854	3.25E-11	1.80E-09	3.884658084	3.664397071	-0.589913612	-0.194626373	3.230677622

ENSG00000164237	CMBL	carboxymethylenebutenolidase homolog (Pseudomonas) [Source:HGNC Symbol;Acc:25090]	-3.219469271	3.278289094	43.99157737	3.30E-11	1.82E-09	4.464143145	4.033640892	-0.589913612	0.61425099	2.919198591
ENSG00000076382	SPAG5	sperm associated antigen 5 [Source:HGNC Symbol;Acc:13452]	-2.387189873	4.575766026	43.75629597	3.72E-11	2.05E-09	5.259568469	5.463639162	2.642776537	2.724061344	4.563054379
ENSG00000162063	CCNF	cyclin F [Source:HGNC Symbol;Acc:1591]	-2.528688954	4.139369456	43.52546647	4.18E-11	2.30E-09	4.939521629	5.03708129	2.404162378	1.65228493	3.986396982
ENSG00000118113	MMP8	matrix metallopeptidase 8 (neutrophil collagenase) [Source:HGNC Symbol;Acc:7175]	-3.854048087	5.294282011	43.33663236	4.61E-11	2.51E-09	5.869936475	4.003013182	1.163613388	0.696304622	6.878518871
ENSG00000254244	PAICSP4	phosphoribosylaminoimidazole carboxylase, phosphoribosylaminoimidazole succinocarboxamide synthetase pseudogene 4 [Source:HGNC Symbol;Acc:38097]	-2.424940348	4.927097701	43.33549042	4.61E-11	2.51E-09	5.884868544	5.64732062	3.506736406	2.580193821	4.658912683
ENSG00000235169	SMIM1	small integral membrane protein 1 (Vel blood group) [Source:HGNC Symbol;Acc:44204]	-3.154006475	2.428984707	43.24763776	4.82E-11	2.62E-09	3.536089402	3.476530889	-0.271377577	-0.194626373	1.11240278
ENSG00000211890	IGHA2	immunoglobulin heavy constant alpha 2 (A2m marker) [Source:HGNC Symbol;Acc:5479]	-4.554279313	7.807400896	43.10500502	5.19E-11	2.81E-09	9.106923328	8.279054341	4.966295509	2.249705807	7.792661161
ENSG00000131153	GINS2	GINS complex subunit 2 (Psf2 homolog) [Source:HGNC Symbol;Acc:24575]	-3.125930774	2.704757814	43.0509546	5.33E-11	2.88E-09	3.736557285	3.516835107	-0.589913612	-0.350682213	2.602374247
ENSG00000077152	UBE2T	ubiquitin-conjugating enzyme E2T (putative) [Source:HGNC Symbol;Acc:25009]	-2.813833146	3.332946811	42.98952165	5.50E-11	2.96E-09	4.459950745	4.088156756	1.076464519	0.917704421	2.821122975
ENSG00000109255	NMU	neuromedin U [Source:HGNC Symbol;Acc:7859]	-5.161023161	2.479999119	42.96803363	5.56E-11	2.99E-09	4.215787888	2.916017525	-2.845623393	-7.002070102	0.415212929
ENSG00000004799	PDK4	pyruvate dehydrogenase kinase, isozyme 4 [Source:HGNC Symbol;Acc:8812]	2.263029826	5.39411854	42.91483723	5.72E-11	3.06E-09	3.794307631	4.078396779	5.870591667	6.061972391	5.753027148
ENSG00000100526	CDKN3	cyclin-dependent kinase inhibitor 3 [Source:HGNC Symbol;Acc:1791]	-2.908876918	3.001963766	42.85311627	5.90E-11	3.15E-09	3.995980462	3.839289117	0.437007322	-0.053815412	2.841278738
ENSG00000142731	PLK4	polo-like kinase 4 [Source:HGNC Symbol;Acc:11397]	-2.631417268	4.104233508	42.77884981	6.13E-11	3.26E-09	4.921340809	4.929901731	1.732719555	2.003353492	4.135309052
ENSG00000128050	PAICS	phosphoribosylaminoimidazole carboxylase, phosphoribosylaminoimidazole succinocarboxamide synthetase [Source:HGNC Symbol;Acc:8587]	-2.179570911	5.819591605	42.75045634	6.22E-11	3.30E-09	6.626909194	6.623494129	4.635719974	3.985013877	5.457941045
ENSG00000100162	CENPM	centromere protein M [Source:HGNC Symbol;Acc:18352]	-2.859745603	2.994806245	42.66061667	6.51E-11	3.44E-09	3.948891281	3.853665234	0.632982468	0.301169826	2.779946473
ENSG00000128683	GAD1	glutamate decarboxylase 1 (brain, 67kDa) [Source:HGNC Symbol;Acc:4092]	-9.536341807	0.991152044	42.53553153	6.94E-11	3.66E-09	2.429680882	1.607225949	-7.002070102	-7.002070102	0.264147325
ENSG00000214357	NEURL1B	neuralized E3 ubiquitin protein ligase 1B [Source:HGNC Symbol;Acc:35422]	-2.799439352	3.236148426	42.10156912	8.67E-11	4.55E-09	4.178001936	4.114661149	0.570569237	0.556835562	3.074840951
ENSG00000228716	DHFR	dihydrofolate reductase [Source:HGNC Symbol;Acc:2861]	-2.144377924	5.237230454	41.83806434	9.92E-11	5.20E-09	5.911209348	6.153052141	3.869549403	3.501502523	4.954005614
ENSG00000211664	IGLV2-18	immunoglobulin lambda variable 2-18 [Source:HGNC Symbol;Acc:5889]	-4.332741951	2.106899174	41.61372192	1.11E-10	5.80E-09	3.507937861	2.401300588	-2.539967122	-2.97427061	2.109797605
ENSG00000162337	LRP5	low density lipoprotein receptor-related protein 5 [Source:HGNC Symbol;Acc:6697]	-2.748735894	3.466526225	41.61019781	1.11E-10	5.80E-09	4.43453724	4.349864383	1.432844522	0.669468625	3.114026435
ENSG00000051180	RAD51	RAD51 recombinase [Source:HGNC Symbol;Acc:9817]	-2.895485208	3.019057066	41.47948498	1.19E-10	6.19E-09	3.84019003	3.90149947	-0.10933503	0.154058352	3.157544141
ENSG00000154620	TMSB4Y	thymosin beta 4, Y-linked [Source:HGNC Symbol;Acc:11882]	9.283181139	0.22920686	41.37677561	1.26E-10	6.50E-09	-7.002070102	-7.002070102	-4.621297774	1.692827451	1.198257616
ENSG00000171241	SHCBP1	SHC SH2-domain binding protein 1 [Source:HGNC Symbol;Acc:29547]	-2.643566308	3.953264864	41.25528102	1.34E-10	6.90E-09	4.761906008	4.817053504	1.415193986	1.79568083	3.971234054
ENSG00000173898	SPTBN2	spectrin, beta, non-erythrocytic 2 [Source:HGNC Symbol;Acc:11276]	-3.262370633	3.533255363	41.06155109	1.48E-10	7.59E-09	4.661426622	4.564332671	-1.57307192	1.168289627	2.586540774
ENSG00000211644	IGLV1-51	immunoglobulin lambda variable 1-51 [Source:HGNC Symbol;Acc:5882]	-3.910821489	3.606744294	40.66898153	1.80E-10	9.26E-09	5.010036873	4.090586468	1.007464306	-1.854339469	3.184090844
ENSG00000167325	RRM1	ribonucleotide reductase M1 [Source:HGNC Symbol;Acc:10451]	-2.13713607	5.896649132	40.42218473	2.05E-10	1.05E-08	6.639939052	6.610431387	4.724388461	4.038427289	5.839903917
ENSG00000136542	GALNT5	UDP-N-acetyl-alpha-D-galactosamine:polypeptide N-acetylgalactosaminyltransferase 5 (GalNAc-T5) [Source:HGNC Symbol;Acc:4127]	-5.868594767	1.61157069	40.12697239	2.38E-10	1.21E-08	2.80506858	2.860151049	-4.621297774	-7.002070102	-0.649489103
ENSG00000144130	NT5DC4	5'-nucleotidase domain containing 4 [Source:HGNC Symbol;Acc:27678]	5.023879403	-0.485649494	40.09691348	2.42E-10	1.23E-08	-3.79890385	-3.414167066	-1.315629595	0.527247606	0.378903451

ENSG00000088340	FER1L4	fer-1-like 4 (C. elegans), pseudogene [Source:HGNC Symbol;Acc:15801]	-3.532204313	2.766130002	40.05988959	2.46E-10	1.25E-08	4.072115865	3.446494941	-0.453950945	-2.97427061	2.363133567
ENSG00000183111	ARHGEF37	Rho guanine nucleotide exchange factor (GEF) 37 [Source:HGNC Symbol;Acc:34430]	-3.345840281	2.330808675	40.03607803	2.49E-10	1.26E-08	3.216393469	3.534789485	-1.315629595	-1.854339469	1.702990823
ENSG00000180537	RNF182	ring finger protein 182 [Source:HGNC Symbol;Acc:28522]	-3.880621039	2.213440885	40.02515056	2.51E-10	1.26E-08	3.458362025	3.110529943	-3.233942493	-2.419096061	1.564363944
ENSG00000213959	RP5-1100H13.3		-3.616622907	1.962214851	39.96417163	2.59E-10	1.30E-08	3.042264405	2.837184307	-2.539967122	-1.449512048	1.702990823
ENSG00000168447	SCNN1B	sodium channel, non-voltage-gated 1, beta subunit [Source:HGNC Symbol;Acc:10600]	-9.003121511	0.393161012	39.8729806	2.71E-10	1.36E-08	1.752861822	1.339492543	-7.002070102	-7.002070102	-1.55968627
ENSG00000166147	FBN1	fibrillin 1 [Source:HGNC Symbol;Acc:3603]	-2.984241789	2.984012712	39.46814515	3.33E-10	1.67E-08	4.170324518	3.92347243	-0.329705368	0.798919003	1.81594051
ENSG00000080819	CPOX	coproporphyrinogen oxidase [Source:HGNC Symbol;Acc:2321]	-2.026015378	5.657254672	39.28803416	3.66E-10	1.82E-08	6.291625332	6.570082921	4.535127562	4.066983011	5.274935181
ENSG00000136824	SMC2	structural maintenance of chromosomes 2 [Source:HGNC Symbol;Acc:14011]	-2.075291789	5.504726319	39.06673637	4.10E-10	2.04E-08	6.100853092	6.29201996	4.321978924	3.60780319	5.570886009
ENSG00000017483	SLC38A5	solute carrier family 38, member 5 [Source:HGNC Symbol;Acc:18070]	-2.676270765	5.31443829	39.02323579	4.19E-10	2.08E-08	6.241825604	6.25621489	4.096915113	2.580193821	4.550866558
ENSG00000080986	NDC80	NDC80 kinetochore complex component [Source:HGNC Symbol;Acc:16909]	-2.513536556	3.816675865	38.91732503	4.42E-10	2.19E-08	4.674149786	4.659050332	2.118106677	1.168289627	3.674416329
ENSG00000118523	CTGF	connective tissue growth factor [Source:HGNC Symbol;Acc:2500]	-9.794228633	1.288322115	38.86404692	4.54E-10	2.24E-08	2.730456787	2.163523194	-7.002070102	-7.002070102	-0.376928291
ENSG00000110400	PVRL1	poliovirus receptor-related 1 (herpesvirus entry mediator C) [Source:HGNC Symbol;Acc:9706]	-2.235297658	5.495525533	38.83979315	4.60E-10	2.26E-08	6.284530805	6.296236409	3.694039364	4.283760478	5.181845156
ENSG00000177324	BEND2	BEN domain containing 2 [Source:HGNC Symbol;Acc:28509]	3.58690193	2.872956123	38.64922442	5.07E-10	2.49E-08	0.401371743	1.050973916	2.814185793	4.192457932	2.93178569
ENSG00000204385	SLC44A4	solute carrier family 44, member 4 [Source:HGNC Symbol;Acc:13941]	-4.689491111	1.763900245	38.41013103	5.73E-10	2.80E-08	3.08644716	2.932364153	-4.621297774	-3.888428289	-1.3021922
ENSG00000242550	SERPINB10	serpin peptidase inhibitor, clade B (ovalbumin), member 10 [Source:HGNC Symbol;Acc:8942]	-2.637141936	3.759197196	38.1829641	6.44E-10	3.14E-08	4.304969154	4.403615446	1.746900214	0.335717742	4.451656561
ENSG00000123473	STIL	SCL/TAL1 interrupting locus [Source:HGNC Symbol;Acc:10879]	-2.514318483	3.649540726	38.11489472	6.67E-10	3.24E-08	4.511499931	4.550279288	1.501363361	1.477660161	3.403296679
ENSG00000047457	CP	ceruloplasmin (ferroxidase) [Source:HGNC Symbol;Acc:2295]	-4.179282876	2.040509193	38.03761239	6.94E-10	3.37E-08	3.54006658	2.948527637	-4.621297774	-2.419096061	-0.57637152
ENSG00000112312	GMNN	geminin, DNA replication inhibitor [Source:HGNC Symbol;Acc:17493]	-2.566696618	3.861707415	38.02546173	6.98E-10	3.38E-08	4.975210034	4.514534988	1.815785154	1.880029835	3.443406881
ENSG00000118513	MYB	v-myb avian myeloblastosis viral oncogene homolog [Source:HGNC Symbol;Acc:7545]	-2.811439606	5.708929199	37.91295642	7.40E-10	3.57E-08	6.507579784	6.323346988	2.687575605	3.848125445	6.115649361
ENSG00000250290	CTC-820M8.1		-3.171986091	2.301010599	37.67329883	8.36E-10	4.02E-08	3.371751013	3.061830389	-1.202308962	-0.46496394	2.164148811
ENSG00000127589	TUBBP1	tubulin, beta pseudogene 1 [Source:HGNC Symbol;Acc:12414]	-2.045652472	6.378496929	37.51524016	9.07E-10	4.35E-08	7.121490019	7.090008309	4.9201124	5.019609773	6.276024361
ENSG00000101938	CHRDL1	chordin-like 1 [Source:HGNC Symbol;Acc:29861]	-5.147307529	1.646762345	37.49305898	9.17E-10	4.39E-08	3.047861816	2.535760257	-7.002070102	-3.888428289	0.264147325
ENSG00000123975	CKS2	CDC28 protein kinase regulatory subunit 2 [Source:HGNC Symbol;Acc:2000]	-2.737579349	3.423267612	37.44232251	9.42E-10	4.49E-08	4.597980204	4.026044706	1.030832979	1.414416401	3.02284327
ENSG00000117411	B4GALT2	UDP-Gal:betaGlcNAc beta 1,4- galactosyltransferase, polypeptide 2 [Source:HGNC Symbol;Acc:925]	-2.571009307	3.241873035	37.21663307	1.06E-09	5.03E-08	4.276645101	4.138340674	1.163613388	0.984547783	2.529713762
ENSG00000213088	DARC	Duffy blood group, atypical chemokine receptor [Source:HGNC Symbol;Acc:4035]	-4.652760622	1.512940133	36.88707157	1.25E-09	5.93E-08	2.394966414	2.772072928	-3.766908349	-4.730697034	0.791775511
ENSG00000255075	RP11-23B7.3		-2.966993656	2.566774249	36.88620859	1.25E-09	5.93E-08	3.650879106	3.516835107	-0.329705368	0.154058352	1.760570749
ENSG00000211630	IGKV1D-13	immunoglobulin kappa variable 1D-13 [Source:HGNC Symbol;Acc:5747]	-3.405627817	2.874308625	36.84493712	1.28E-09	6.04E-08	3.636091341	3.143667191	-2.845623393	-0.296766077	3.827080113
ENSG00000154839	SKA1	spindle and kinetochore associated complex subunit 1 [Source:HGNC Symbol;Acc:28109]	-2.754821706	2.812316036	36.64285376	1.42E-09	6.68E-08	3.665516831	3.756023105	-0.059127673	0.154058352	2.708594515
ENSG00000257331	RP11-478B9.3		-2.415520832	3.705905943	36.48570321	1.54E-09	7.22E-08	4.60748737	4.455435609	2.016416749	1.65228493	3.516256447
ENSG00000182512	GLRX5	glutaredoxin 5 [Source:HGNC Symbol;Acc:20134]	-2.003217172	5.426901403	36.36982748	1.63E-09	7.64E-08	6.186444049	6.24207749	4.286013837	4.074673831	4.902179967
ENSG00000154917	RAB6B	RAB6B, member RAS oncogene family [Source:HGNC Symbol;Acc:14902]	-2.559531049	3.274367861	36.2518356	1.73E-09	8.10E-08	4.033140065	4.444079802	0.934997507	1.187332556	2.513057684
ENSG00000231535	LINC00278	long intergenic non-protein coding RNA 278 [Source:HGNC Symbol;Acc:38712]	8.903429359	-0.349526206	36.00855492	1.96E-09	9.15E-08	-7.002070102	-7.002070102	-7.002070102	1.12943269	0.518960621
ENSG00000167244	IGF2	insulin-like growth factor 2 (somatomedin A) [Source:HGNC Symbol;Acc:5466]	-5.242500763	1.327251199	35.95560555	2.02E-09	9.37E-08	2.882290283	2.163523194	-7.002070102	-3.888428289	-1.70800759
ENSG00000115163	CENPA	centromere protein A [Source:HGNC Symbol;Acc:1851]	-3.340015743	2.351055638	35.94913741	2.03E-09	9.38E-08	3.407021786	3.120075649	-2.539967122	-1.449512048	2.426764636
ENSG00000129810	SGOL1	shugoshin-like 1 (S. pombe) [Source:HGNC Symbol;Acc:25088]	-2.780471427	2.684909638	35.8501209	2.13E-09	9.82E-08	3.669153174	3.487635086	0.036330944	-0.350682213	2.610226292

ENSG00000157404	KIT	v-kit Hardy-Zuckerman 4 feline sarcoma viral oncogene homolog [Source:HGNC Symbol;Acc:6342]	-2.718878364	3.836745806	35.85519659	2.13E-09	9.82E-08	4.834948985	4.08084297	1.304500245	1.242998101	4.384566858
ENSG00000169397	RNASE3	ribonuclease, RNase A family, 3 [Source:HGNC Symbol;Acc:10046]	-3.304094888	2.856619418	35.72558212	2.27E-09	1.04E-07	3.757204751	3.435068262	-2.073364206	0.114810932	3.362039354
ENSG00000239571	IGKV2D-30	immunoglobulin kappa variable 2D-30 [Source:HGNC Symbol;Acc:5801]	-4.04842492	1.675881623	35.69456068	2.31E-09	1.06E-07	3.317173381	2.135558076	-2.539967122	-2.419096061	-0.045536284
ENSG00000188582	PAQR9	progestin and adipoQ receptor family member IX [Source:HGNC Symbol;Acc:30131]	-9.732642548	1.258546919	35.67360309	2.33E-09	1.07E-07	2.207636582	2.618906922	-7.002070102	-7.002070102	-0.257803757
ENSG00000156802	ATAD2	ATPase family, AAA domain containing 2 [Source:HGNC Symbol;Acc:30123]	-1.99948916	5.563681348	35.63220881	2.38E-09	1.09E-07	6.118249329	6.306723932	4.451010711	3.742650341	5.708916583
ENSG00000117650	NEK2	NIMA-related kinase 2 [Source:HGNC Symbol;Acc:7745]	-2.969761734	2.267645208	35.53137328	2.51E-09	1.14E-07	3.32180121	3.171477064	-0.82139248	-0.7248947	1.84284836
ENSG00000213005	PTTG3P	pituitary tumor-transforming 3, pseudogene [Source:HGNC Symbol;Acc:13422]	-2.657688107	3.103984614	35.53467164	2.51E-09	1.14E-07	4.118070306	3.867899509	0.6928072	0.773941511	2.887241838
ENSG00000071539	TRIP13	thyroid hormone receptor interactor 13 [Source:HGNC Symbol;Acc:12307]	-2.782609137	2.71380908	35.50518974	2.54E-09	1.15E-07	3.715610023	3.498654468	-0.329705368	-0.053815412	2.656461736
ENSG00000011465	DCN	decorin [Source:HGNC Symbol;Acc:2705]	-4.961398641	1.523649826	35.35021205	2.75E-09	1.24E-07	2.900966723	2.591720352	-4.621297774	-4.730697034	-1.083770229
ENSG00000162367	TAL1	T-cell acute lymphocytic leukemia 1 [Source:HGNC Symbol;Acc:11556]	-2.665267245	5.698249692	35.34140936	2.77E-09	1.25E-07	6.481448336	6.733304648	3.055465682	4.37586574	5.106126652
ENSG00000184371	CSF1	colony stimulating factor 1 (macrophage) [Source:HGNC Symbol;Acc:2432]	-2.279375405	4.651829404	35.30726529	2.82E-09	1.26E-07	5.804485679	5.390588949	3.531522919	2.60872607	3.305127547
ENSG00000119333	WDR34	WD repeat domain 34 [Source:HGNC Symbol;Acc:28296]	-2.185489101	4.333272383	35.29917316	2.83E-09	1.27E-07	5.088171916	5.156692896	2.490973667	2.730566978	4.21213175
ENSG00000261349	RP3-465N24.5		6.999395481	-0.28449975	35.14374707	3.06E-09	1.37E-07	-4.650190627	-7.002070102	-4.621297774	0.894716898	0.923655414
ENSG00000160949	TONSL	tonsoku-like, DNA repair protein [Source:HGNC Symbol;Acc:7801]	-2.148133283	4.460180336	35.12820297	3.09E-09	1.38E-07	5.327874839	5.120294025	3.061151464	2.650493526	4.335675838
ENSG00000231831	MTHFD1P1	methylenetetrahydrofolate dehydrogenase (NADP+ dependent) 1 pseudogene 1 [Source:HGNC Symbol;Acc:7433]	-1.966510883	5.570355166	35.09416709	3.14E-09	1.40E-07	6.338621987	6.252964688	4.543275669	4.092461225	5.311515536
ENSG00000023909	GCLM	glutamate-cysteine ligase, modifier subunit [Source:HGNC Symbol;Acc:4312]	-2.004635991	4.987492186	35.00135872	3.29E-09	1.46E-07	5.597747815	5.922234132	3.823456948	3.18362115	4.699962837
ENSG00000149150	SLC43A1	solute carrier family 43 (amino acid system L transporter), member 1 [Source:HGNC Symbol;Acc:9225]	-2.047650037	5.059639947	34.96255566	3.36E-09	1.49E-07	5.873092938	5.888421125	3.98172333	3.266541267	4.534454294
ENSG00000188486	H2AFX	H2A histone family, member X [Source:HGNC Symbol;Acc:4739]	-2.109782666	5.139731382	34.79306531	3.67E-09	1.62E-07	5.828261361	5.631555772	3.455848046	3.680166494	5.51760751
ENSG00000103202	NME4	NME/NM23 nucleoside diphosphate kinase 4 [Source:HGNC Symbol;Acc:7852]	-2.091382224	5.672188089	34.79015137	3.67E-09	1.62E-07	6.432008102	6.426964992	4.689939282	3.790136562	5.413758877
ENSG00000268119	CTD-2561J22.5		-3.322401754	2.061107019	34.78397815	3.68E-09	1.62E-07	2.811663962	3.368549783	-1.886652327	-1.70641174	1.374569317
ENSG00000173626	TRAPPC3L	trafficking protein particle complex 3-like [Source:HGNC Symbol;Acc:21090]	4.174920226	0.669016078	34.64615899	3.95E-09	1.73E-07	-1.921450438	-1.507080648	0.081791624	1.914722448	1.155968757
ENSG00000214888	Xyac-YM21GA2.7		-9.442761224	0.927461254	34.61100815	4.03E-09	1.76E-07	2.103982592	2.235550428	-7.002070102	-7.002070102	-2.832686105
ENSG00000273112	RP11-25K21.6		4.595948522	-0.706761297	34.58289229	4.08E-09	1.78E-07	-3.267125794	-3.414167066	-1.886652327	0.369457657	0.04992943
ENSG00000259358	RP11-456J20.1		-3.759379824	1.463031344	34.5494267	4.16E-09	1.81E-07	2.552892059	2.320804564	-3.766908349	-2.205328685	1.218946104
ENSG00000167747	C19orf48	chromosome 19 open reading frame 48 [Source:HGNC Symbol;Acc:29667]	-2.029135591	5.095505411	34.53589889	4.18E-09	1.81E-07	6.020576715	5.736128797	3.990689068	3.67004846	4.573132831
ENSG00000173848	NET1	neuroepithelial cell transforming 1 [Source:HGNC Symbol;Acc:14592]	-2.334168968	4.859591206	34.52666284	4.20E-09	1.82E-07	5.707913644	5.799878799	3.765432491	2.311762486	4.193959434
ENSG00000173262	SLC2A14	solute carrier family 2 (facilitated glucose transporter), member 14 [Source:HGNC Symbol;Acc:18301]	-3.015484871	2.298548921	34.51380101	4.23E-09	1.83E-07	3.437191842	3.260485804	-1.438615935	-0.7248947	1.531661491
ENSG00000068137	PLEKHH3	pleckstrin homology domain containing, family H (with MyTH4 domain) member 3 [Source:HGNC Symbol;Acc:26105]	-2.778972843	2.609361613	34.48873338	4.29E-09	1.84E-07	3.428636013	3.718811978	-0.453950945	0.301169826	2.098677261
ENSG00000241859	KALP	Kallmann syndrome sequence pseudogene [Source:HGNC Symbol;Acc:6214]	7.096322213	-0.206891639	34.37455466	4.55E-09	1.95E-07	-7.002070102	-4.777156855	-7.002070102	1.224680533	0.735452007
ENSG00000079257	LXN	latexin [Source:HGNC Symbol;Acc:13347]	-2.399180237	3.648845062	34.36950207	4.56E-09	1.95E-07	4.730925147	4.476025242	2.096120255	1.314027603	2.772967998
ENSG00000116774	OLFML3	olfactomedin-like 3 [Source:HGNC Symbol;Acc:24956]	-3.661190359	1.431130867	34.31977633	4.68E-09	2.00E-07	2.791786669	2.409108576	-3.233942493	-2.0191864	-0.80787826
ENSG00000188985	DHFRP1	dihydrofolate reductase pseudogene 1 [Source:HGNC Symbol;Acc:2862]	-2.085276549	4.312136203	34.28602757	4.76E-09	2.03E-07	5.117746192	5.089041497	3.009154251	2.543716201	4.033874407
ENSG00000211599	IGKV5-2	immunoglobulin kappa variable 5-2 [Source:HGNC Symbol;Acc:5835]	-3.898360973	1.916993619	34.21208066	4.94E-09	2.10E-07	2.961531071	2.126114598	-1.721358966	-4.730697034	2.618035833
ENSG00000239819	IGKV1D-8	immunoglobulin kappa variable 1D-8 [Source:HGNC Symbol;Acc:5759]	-3.717297103	1.768452289	34.1978428	4.98E-09	2.11E-07	3.260449604	2.304152017	-1.315629595	-2.97427061	0.763888603

ENSG00000211934	IGHV1-2	immunoglobulin heavy variable 1-2 [Source:HGNC Symbol;Acc:5550]	-4.392154364	7.0245231	34.13705405	5.14E-09	2.17E-07	8.519245345	7.694642444	4.373157081	0.917704421	5.754799245
ENSG00000168542	COL3A1	collagen, type III, alpha 1 [Source:HGNC Symbol;Acc:2201]	-9.613628133	1.064528481	34.08561839	5.27E-09	2.23E-07	2.560743753	2.01793956	-7.002070102	-7.002070102	-2.526883755
ENSG00000162062	C16orf59	chromosome 16 open reading frame 59 [Source:HGNC Symbol;Acc:25849]	-3.082694899	1.981467207	33.99718122	5.52E-09	2.32E-07	2.937609558	2.848713379	-1.315629595	-1.133744865	1.895203721
ENSG00000223519	KIF28P	kinesin family member 28, pseudogene [Source:HGNC Symbol;Acc:49205]	3.759092057	0.313549263	33.94007314	5.68E-09	2.39E-07	-1.47369337	-1.507080648	0.437007322	1.79568083	-1.42520211
ENSG00000134668	SPOCD1	SPOC domain containing 1 [Source:HGNC Symbol;Acc:26338]	4.206467013	1.995659798	33.93309346	5.70E-09	2.39E-07	-1.47369337	0.056395802	1.432844522	3.37017149	2.381602043
ENSG00000223723	BX842568.2		-1.87267929	5.513899674	33.8452959	5.97E-09	2.49E-07	6.21719769	6.322830407	4.345467014	4.102527846	5.177887994
ENSG00000211632	IGKV3D-11	immunoglobulin kappa variable 3D-11 [Source:HGNC Symbol;Acc:5823]	-3.324832306	2.281230092	33.79294873	6.13E-09	2.56E-07	3.621150429	2.605377675	-0.520331194	-1.572251189	2.206202022
ENSG00000211633	IGKV1D-42	immunoglobulin kappa variable 1D-42 (non-functional) [Source:HGNC Symbol;Acc:5757]	-4.491681418	1.434282551	33.77453363	6.19E-09	2.57E-07	2.785099575	1.986973075	-2.845623393	-7.002070102	1.155968757
ENSG00000154330	PGM5	phosphoglucomutase 5 [Source:HGNC Symbol;Acc:8908]	4.659045712	-0.030601481	33.54070403	6.98E-09	2.90E-07	-3.267125794	-2.726009782	-1.097244829	1.261086007	0.584199832
ENSG00000260197	RP11-424G14.1		8.463532297	-0.316095338	33.42453446	7.41E-09	3.07E-07	-7.002070102	-7.002070102	-7.002070102	0.798919003	0.997359302
ENSG00000165124	SVEP1	sushi, von Willebrand factor type A, EGF and pentraxin domain containing 1 [Source:HGNC Symbol;Acc:15985]	-9.255418109	0.82085954	33.35373247	7.68E-09	3.17E-07	2.332130562	1.761572815	-7.002070102	-7.002070102	-3.754655114
ENSG00000112212	TSPO2	translocator protein 2 [Source:HGNC Symbol;Acc:21256]	-5.717525953	1.405157882	33.2998756	7.90E-09	3.25E-07	1.832851647	2.877139309	-4.621297774	-7.002070102	0.948644093
ENSG00000092853	CLSPN	claspin [Source:HGNC Symbol;Acc:19715]	-2.697534922	3.244609183	33.29141347	7.93E-09	3.26E-07	3.909466259	4.159324949	-0.271377577	0.940331404	3.516256447
ENSG00000143479	DYRK3	dual-specificity tyrosine-(Y)-phosphorylation regulated kinase 3 [Source:HGNC Symbol;Acc:3094]	-2.8925842	2.270199914	33.14101739	8.57E-09	3.52E-07	3.165810641	3.281905652	-0.907612816	-1.042179808	2.030092686
ENSG00000227165	WDR11-AS1	WDR11 antisense RNA 1 [Source:HGNC Symbol;Acc:27437]	4.274608079	1.586418494	33.10147989	8.75E-09	3.58E-07	-0.856736938	-0.855949929	0.983710868	3.32789308	0.646616095
ENSG00000120158	RCL1	RNA terminal phosphate cyclase-like 1 [Source:HGNC Symbol;Acc:17687]	-2.164354298	4.630963537	33.06992248	8.89E-09	3.63E-07	5.610096937	5.433330763	3.622778389	2.664151552	3.68560772
ENSG00000100321	SYNGR1	synaptogyrin 1 [Source:HGNC Symbol;Acc:11498]	-1.931310678	5.293255651	32.9668333	9.37E-09	3.82E-07	6.040366658	6.034387989	3.901593689	3.951990081	5.086559371
ENSG00000137757	CASP5	caspase 5, apoptosis-related cysteine peptidase [Source:HGNC Symbol;Acc:1506]	3.364717153	1.464052938	32.92738709	9.57E-09	3.89E-07	-0.698419327	-0.204475541	1.342349735	2.175352965	2.32547185
ENSG00000116748	AMPD1	adenosine monophosphate deaminase 1 [Source:HGNC Symbol;Acc:468]	-4.607864376	1.765429326	32.90237121	9.69E-09	3.93E-07	3.33559629	2.439924693	-3.766908349	-4.730697034	0.095393329
ENSG00000136842	TMOD1	tropomodulin 1 [Source:HGNC Symbol;Acc:11871]	-2.387819897	3.63162505	32.85855355	9.91E-09	4.01E-07	4.380109475	4.724655254	2.213098418	1.187332556	2.900109607
ENSG00000153044	CENPH	centromere protein H [Source:HGNC Symbol;Acc:17268]	-2.29915903	3.793963214	32.8293588	1.01E-08	4.06E-07	4.666893152	4.607387401	2.358715867	1.39816257	3.524590078
ENSG00000237972	TUBG1P	tubulin, gamma 1 pseudogene [Source:HGNC Symbol;Acc:12418]	-2.177665836	4.055811469	32.72355576	1.06E-08	4.27E-07	4.805513338	5.085391481	2.723873993	2.165783527	3.295420086
ENSG00000233614	DDX11L10	DEAD/H (Asp-Glu-Ala-Asp/His) box helicase 11 like 10 [Source:HGNC Symbol;Acc:14125]	4.88956103	2.372965771	32.72789165	1.06E-08	4.27E-07	-1.921450438	-0.204475541	1.467510679	4.168699992	1.882291923
ENSG00000196584	XRCC2	X-ray repair complementing defective repair in Chinese hamster cells 2 [Source:HGNC Symbol;Acc:12829]	-2.473940172	3.070812261	32.62852955	1.12E-08	4.47E-07	4.024649331	3.881994713	1.163613388	0.434658742	2.8278729
ENSG00000131002	TXLNG2P	taxilin gamma 2, pseudogene [Source:HGNC Symbol;Acc:18473]	7.972080975	4.931235413	32.55406806	1.16E-08	4.64E-07	-2.108017771	-1.91149394	-1.886652327	6.233239397	6.253639536
ENSG00000211670	IGLV3-9	immunoglobulin lambda variable 3-9 (gene/pseudogene) [Source:HGNC Symbol;Acc:5918]	-3.014476952	3.211596472	32.46153693	1.22E-08	4.85E-07	4.18817535	2.980321272	0.28980717	-0.350682213	4.167595364
ENSG00000067646	ZFY	zinc finger protein, Y-linked [Source:HGNC Symbol;Acc:12870]	6.174245335	2.857026955	32.33243043	1.30E-08	5.17E-07	-2.574166106	-2.475439898	-2.539967122	3.823992547	4.414022873
ENSG00000250334	LINC00989	long intergenic non-protein coding RNA 989 [Source:HGNC Symbol;Acc:48918]	3.518116849	2.74429424	32.28347053	1.33E-08	5.29E-07	0.133035942	0.664086661	2.02807612	3.805624207	3.644141113
ENSG00000111339	ART4	ADP-ribosyltransferase 4 (Dombrock blood group) [Source:HGNC Symbol;Acc:726]	-5.47652359	1.589726749	32.16759111	1.41E-08	5.60E-07	2.197599956	2.865836059	-4.621297774	-7.002070102	1.481174441
ENSG00000272327	RP11-1002K11.1		3.543306934	1.731805867	32.13258238	1.44E-08	5.69E-07	-2.879434292	-1.507080648	0.210162819	0.497040107	3.743871718
ENSG00000181631	P2RY13	purinergic receptor P2Y, G-protein coupled, 13 [Source:HGNC Symbol;Acc:4537]	1.864106089	5.723203092	32.06651865	1.49E-08	5.88E-07	4.328155027	4.428798332	5.947049467	6.22116301	6.425913578
ENSG00000259179	UBE2CP4	ubiquitin-conjugating enzyme E2C pseudogene 4 [Source:HGNC Symbol;Acc:43553]	-3.25513104	1.721703763	32.04235774	1.51E-08	5.94E-07	2.857006212	2.564011614	-2.287883461	-1.336401299	1.279288931
ENSG00000012048	BRCA1	breast cancer 1, early onset [Source:HGNC Symbol;Acc:1100]	-2.086418567	4.461072301	31.6945379	1.80E-08	7.09E-07	5.1230588	5.202341784	2.650340322	2.947141733	4.595060667

ENSG00000106537	TSPAN13	tetraspanin 13 [Source:HGNC Symbol;Acc:21643]	-2.177821976	3.993036913	31.56965561	1.92E-08	7.54E-07	4.670526032	4.419164558	2.171650019	1.596392694	4.641776875
ENSG00000146592	CREB5	cAMP responsive element binding protein 5 [Source:HGNC Symbol;Acc:16844]	2.114604018	5.716981857	31.49275139	2.00E-08	7.83E-07	3.621150429	4.321184566	5.625934453	6.485326789	6.515395492
ENSG00000105507	CABP5	calcium binding protein 5 [Source:HGNC Symbol;Acc:13714]	3.603937037	1.520114341	31.48095359	2.01E-08	7.86E-07	-0.555769858	-0.408932621	1.184602189	2.730566978	1.895203721
ENSG00000233013	FAM157B	family with sequence similarity 157, member B [Source:HGNC Symbol;Acc:34080]	3.589048182	0.262973037	31.47489423	2.02E-08	7.86E-07	-1.756270337	-1.394066322	0.081791624	1.261086007	0.676839958
ENSG00000164292	RHOBTB3	Rho-related BTB domain containing 3 [Source:HGNC Symbol;Acc:18757]	-2.314414211	4.220160163	31.46921536	2.03E-08	7.87E-07	5.180241648	5.042111296	2.139763053	2.818693563	3.609304371
ENSG00000165702	GFI1B	growth factor independent 1B transcription repressor [Source:HGNC Symbol;Acc:4238]	-2.540341553	5.49982241	31.43322171	2.06E-08	8.00E-07	6.359590578	6.492739786	2.899175647	4.296970828	4.734498925
ENSG00000186603	HPDL	4-hydroxyphenylpyruvate dioxygenase-like [Source:HGNC Symbol;Acc:28242]	-3.038808596	1.973982483	31.41866786	2.08E-08	8.04E-07	3.236140248	2.73527877	-0.329705368	-1.572251189	1.090116303
ENSG00000172752	COL6A5	collagen, type VI, alpha 5 [Source:HGNC Symbol;Acc:26674]	-5.375117847	1.153884004	31.39336223	2.11E-08	8.13E-07	2.304339895	2.295753104	-7.002070102	-4.730697034	-0.147769603
ENSG00000099725	PRKY	protein kinase, Y-linked, pseudogene [Source:HGNC Symbol;Acc:9444]	7.113868035	4.104635847	31.22041502	2.30E-08	8.87E-07	-1.756270337	-2.475439898	-2.073364206	5.682866335	5.072418452
ENSG00000121621	KIF18A	kinesin family member 18A [Source:HGNC Symbol;Acc:29441]	-2.397402345	2.982060569	31.19351308	2.34E-08	8.97E-07	3.77756088	3.845056765	1.030832979	0.798919003	2.874258264
ENSG00000071794	HLTF	helicase-like transcription factor [Source:HGNC Symbol;Acc:11099]	-1.980036406	5.247334666	31.0643614	2.50E-08	9.57E-07	5.928000649	6.053195615	4.307700297	3.284338193	5.034989944
ENSG00000146918	NCAPG2	non-SMC condensin II complex, subunit G2 [Source:HGNC Symbol;Acc:21904]	-1.855493383	4.971278828	31.04392435	2.52E-08	9.65E-07	5.523357725	5.759221375	3.71943079	3.462952422	5.008497083
ENSG00000211598	IGKV4-1	immunoglobulin kappa variable 4-1 [Source:HGNC Symbol;Acc:5834]	-3.858604642	8.382615734	30.92785642	2.68E-08	1.02E-06	9.915681259	8.83870785	3.840088097	5.97902694	7.386669125
ENSG00000196139	AKR1C3	aldo-keto reductase family 1, member C3 [Source:HGNC Symbol;Acc:386]	-2.374405617	4.5966422	30.86212621	2.77E-08	1.05E-06	5.791198376	5.1578519	2.596541649	3.120624317	3.892932647
ENSG00000113924	HGD	homogentisate 1,2-dioxygenase [Source:HGNC Symbol;Acc:4892]	3.196542535	1.610531743	30.86683134	2.76E-08	1.05E-06	0.000762307	0.277241192	1.829176068	2.830854872	1.06748014
ENSG00000183878	UTY	ubiquitously transcribed tetratricopeptide repeat containing, Y-linked [Source:HGNC Symbol;Acc:12638]	7.48448167	4.065219902	30.86310185	2.77E-08	1.05E-06	-2.879434292	-1.763731145	-2.539967122	5.571315722	5.140421471
ENSG00000242766	IGKV1D-17	immunoglobulin kappa variable 1D-17 [Source:HGNC Symbol;Acc:5749]	-4.049596981	1.353554849	30.81528245	2.84E-08	1.07E-06	2.818229329	1.785765038	-3.766908349	-2.97427061	0.84598249
ENSG00000157734	SNX22	sorting nexin 22 [Source:HGNC Symbol;Acc:16315]	-2.354050617	4.715718059	30.78785175	2.88E-08	1.09E-06	5.544299326	5.290579393	3.510897178	2.13668837	4.882862945
ENSG00000111843	TMEM14C	transmembrane protein 14C [Source:HGNC Symbol;Acc:20952]	-1.844192467	5.418385976	30.77476966	2.90E-08	1.09E-06	6.24730942	6.136686079	4.357069198	4.390318387	4.6851703
ENSG00000167701	GPT	glutamic-pyruvate transaminase (alanine aminotransferase) [Source:HGNC Symbol;Acc:4552]	-3.300062759	1.13114873	30.68391411	3.04E-08	1.14E-06	2.341276415	2.097407515	-2.287883461	-2.0191864	-0.257803757
ENSG00000136982	DSCC1	DNA replication and sister chromatid cohesion 1 [Source:HGNC Symbol;Acc:24453]	-2.520989212	2.692687342	30.65726199	3.08E-08	1.15E-06	3.628640226	3.590786525	0.125863448	0.335717742	2.390748373
ENSG00000076248	UNG	uracil-DNA glycosylase [Source:HGNC Symbol;Acc:12572]	-1.9198069	4.652781641	30.45560634	3.42E-08	1.28E-06	5.394191815	5.249855047	3.49837869	2.918936894	4.729100523
ENSG00000214688	C10orf105	chromosome 10 open reading frame 105 [Source:HGNC Symbol;Acc:20304]	3.258555061	1.919877748	30.45588673	3.42E-08	1.28E-06	0.000762307	0.527293245	1.829176068	3.140302572	1.933259916
ENSG00000185198	PRSS57	protease, serine, 57 [Source:HGNC Symbol;Acc:31397]	-2.898620117	4.222433372	30.44028799	3.44E-08	1.28E-06	5.070406928	4.828743922	0.210162819	2.36290873	4.628306871
ENSG00000114374	USP9Y	ubiquitin specific peptidase 9, Y-linked [Source:HGNC Symbol;Acc:12633]	7.53284949	4.169554711	30.41259178	3.49E-08	1.30E-06	-1.921450438	-3.940353384	-3.233942493	5.49503679	5.465535705
ENSG00000169313	P2RY12	purinergic receptor P2Y, G-protein coupled, 12 [Source:HGNC Symbol;Acc:18124]	3.148419191	2.165288887	30.37808694	3.56E-08	1.32E-06	0.174562846	0.859104642	2.321298905	3.361814346	1.97033799
ENSG00000156265	MAP3K7CL	MAP3K7 C-terminal like [Source:HGNC Symbol;Acc:16457]	1.946038287	5.688356144	30.36082225	3.59E-08	1.33E-06	4.269476339	4.681789027	5.975178438	6.497298392	5.891559125
ENSG00000012817	KDM5D	lysine (K)-specific demethylase 5D [Source:HGNC Symbol;Acc:11115]	7.920254969	5.692849322	30.3279666	3.65E-08	1.35E-06	-1.237502295	-1.100062965	-1.721358966	6.979620611	7.033273063
ENSG00000271736	RP11-85G21.3		-3.687051604	1.208134812	30.09585814	4.11E-08	1.52E-06	1.819824215	2.57098885	-2.539967122	-3.360122781	0.551948963
ENSG00000231331	AC103563.2		-4.382163068	1.27138033	30.05016345	4.21E-08	1.55E-06	2.850615345	1.761572815	-2.845623393	-7.002070102	0.04992943
ENSG00000134755	DSC2	desmocollin 2 [Source:HGNC Symbol;Acc:3036]	3.761447406	3.63404551	29.9661423	4.40E-08	1.61E-06	-0.365194284	1.128690501	2.430758609	4.199509687	5.134988789
ENSG00000135446	CDK4	cyclin-dependent kinase 4 [Source:HGNC Symbol;Acc:1773]	-1.829495829	5.756033295	29.95404194	4.42E-08	1.62E-06	6.55207786	6.348943042	4.854504969	4.45852833	5.448117365
ENSG00000211895	IGHA1	immunoglobulin heavy constant alpha 1 [Source:HGNC Symbol;Acc:5478]	-4.323794933	10.91200984	29.92010596	4.50E-08	1.65E-06	12.26482637	11.57775588	7.695510513	5.291193806	10.46085151

ENSG00000081803	CADPS2	Ca++-dependent secretion activator 2 [Source:HGNC Symbol;Acc:16018]	-9.22237299	0.683647474	29.8672518	4.63E-08	1.69E-06	2.197599956	1.339492543	-7.002070102	-7.002070102	-0.726511138
ENSG00000221988	PPT2	palmitoyl-protein thioesterase 2 [Source:HGNC Symbol;Acc:9326]	-2.290022859	3.641673391	29.86197572	4.64E-08	1.69E-06	4.693919354	4.453549171	2.386155201	1.381723529	2.693890668
ENSG00000101825	MXRA5	matrix-remodelling associated 5 [Source:HGNC Symbol;Acc:7539]	-9.085282266	0.679309807	29.8292397	4.72E-08	1.71E-06	2.177314774	1.607225949	-7.002070102	-7.002070102	-3.221241555
ENSG00000151023	ENKUR	enkurin, TRPC channel interacting protein [Source:HGNC Symbol;Acc:28388]	3.392352672	2.295024625	29.81860229	4.74E-08	1.72E-06	0.566455146	0.498306264	2.096120255	3.676801694	2.098677261
ENSG00000103569	AQP9	aquaporin 9 [Source:HGNC Symbol;Acc:643]	2.899737583	6.291271799	29.75774182	4.90E-08	1.77E-06	3.807566461	4.528939153	5.72596636	7.508400022	6.897492756
ENSG00000139329	LUM	lumican [Source:HGNC Symbol;Acc:6724]	-9.205919345	0.63677357	29.53078301	5.50E-08	1.99E-06	2.114689606	1.508923295	-7.002070102	-7.002070102	-1.87334355
ENSG00000249992	TMEM158	transmembrane protein 158 (gene/pseudogene) [Source:HGNC Symbol;Acc:30293]	3.233995323	0.255514397	29.49631035	5.60E-08	2.02E-06	-1.921450438	-1.394066322	0.602113338	0.527247606	1.090116303
ENSG00000134571	MYBPC3	myosin binding protein C, cardiac [Source:HGNC Symbol;Acc:7551]	3.303305891	2.677578388	29.39990265	5.89E-08	2.12E-06	0.365960855	0.881727766	1.980862385	3.793247421	3.430160334
ENSG00000185480	PARPBP	PARP1 binding protein [Source:HGNC Symbol;Acc:26074]	-2.289420015	3.433810024	29.30344946	6.19E-08	2.22E-06	4.44729995	4.102674026	1.517996407	1.462107232	3.189342067
ENSG00000225523	IGKV6D-21	immunoglobulin kappa variable 6D-21 (non-functional) [Source:HGNC Symbol;Acc:5837]	-4.165727936	1.11136374	29.13740478	6.74E-08	2.42E-06	2.429680882	1.537699846	-3.233942493	-4.730697034	1.06748014
ENSG00000137135	ARHGEF39	Rho guanine nucleotide exchange factor (GEF) 39 [Source:HGNC Symbol;Acc:25909]	-2.564840864	2.370300668	29.05445291	7.04E-08	2.52E-06	3.201403963	3.212211862	-0.390491059	-0.296766077	2.461903625
ENSG00000115641	FHL2	four and a half LIM domains 2 [Source:HGNC Symbol;Acc:3703]	-2.544137403	3.034601417	29.02690616	7.14E-08	2.55E-06	4.096626906	3.990057211	-0.010608983	1.224680533	2.109797605
ENSG00000238201	AC114752.3		4.260318813	-0.2249337	28.9568768	7.40E-08	2.64E-06	-3.79890385	-2.726009782	-1.57307192	0.527247606	1.021113618
ENSG00000150048	CLEC1A	C-type lectin domain family 1, member A [Source:HGNC Symbol;Acc:24355]	3.525664107	0.398012129	28.9453326	7.44E-08	2.65E-06	-1.921450438	-0.647226338	0.437007322	1.508271876	0.264147325
ENSG00000135776	ABCB10	ATP-binding cassette, sub-family B (MDR/TAP), member 10 [Source:HGNC Symbol;Acc:41]	-1.764299372	5.491288697	28.88532835	7.68E-08	2.72E-06	5.976499327	6.412957065	4.336117616	4.277109662	5.202768638
ENSG00000181634	TNFSF15	tumor necrosis factor (ligand) superfamily, member 15 [Source:HGNC Symbol;Acc:11931]	3.707769928	0.053430127	28.85757292	7.79E-08	2.76E-06	-2.322346157	-1.763731145	-0.907612816	0.722650538	1.177268129
ENSG00000236304	AP001189.4		3.609184464	3.509677127	28.85363812	7.81E-08	2.76E-06	0.46968809	1.32305554	2.694908712	4.996853041	3.843827185
ENSG00000163735	CXCL5	chemokine (C-X-C motif) ligand 5 [Source:HGNC Symbol;Acc:10642]	2.542858975	3.622051044	28.8496208	7.82E-08	2.76E-06	1.431768352	2.181869923	3.591687772	4.390318387	4.395967379
ENSG00000137310	TCF19	transcription factor 19 [Source:HGNC Symbol;Acc:11629]	-1.920905207	4.879307837	28.79042586	8.06E-08	2.84E-06	5.46284102	5.651440791	3.904759317	3.105687454	4.853393618
ENSG00000123243	ITIH5	inter-alpha-trypsin inhibitor heavy chain family, member 5 [Source:HGNC Symbol;Acc:21449]	-8.443245244	-0.092164154	28.77371879	8.13E-08	2.86E-06	1.361587606	0.812765477	-7.002070102	-7.002070102	-4.610216385
ENSG00000250771	RP11-153M7.3		5.773897674	1.952047645	28.76524879	8.17E-08	2.86E-06	-1.608074432	-3.414167066	-1.721358966	3.736199089	2.31590077
ENSG00000117394	SLC2A1	solute carrier family 2 (facilitated glucose transporter), member 1 [Source:HGNC Symbol;Acc:11005]	-2.048025858	6.401625688	28.75146506	8.23E-08	2.88E-06	6.92942515	7.417495033	5.588036618	4.519907545	5.870464764
ENSG00000092200	RPGRIP1	retinitis pigmentosa GTPase regulator interacting protein 1 [Source:HGNC Symbol;Acc:13436]	3.344344782	1.934459769	28.71772143	8.37E-08	2.92E-06	-0.555769858	0.610915408	1.829176068	3.145180481	2.076176073
ENSG00000106714	CNTNAP3	contactin associated protein-like 3 [Source:HGNC Symbol;Acc:13834]	4.397702077	0.766332978	28.6423663	8.71E-08	3.03E-06	-2.322346157	-3.414167066	-3.233942493	1.381723529	2.399837082
ENSG00000198692	EIF1AY	eukaryotic translation initiation factor 1A, Y-linked [Source:HGNC Symbol;Acc:3252]	5.678500441	2.022970549	28.61710167	8.82E-08	3.07E-06	-2.322346157	-3.414167066	-3.233942493	2.991153022	3.553384437
ENSG00000161800	RACGAP1	Rac GTPase activating protein 1 [Source:HGNC Symbol;Acc:9804]	-2.186778875	3.759002896	28.59696516	8.91E-08	3.09E-06	4.709895349	4.370006427	1.980862385	1.90325069	3.68560772
ENSG00000204872	AC092653.5		3.87233307	0.575343756	28.58516111	8.97E-08	3.10E-06	-1.608074432	-1.014020025	0.328039018	2.035234912	-0.045536284
ENSG00000088882	CPXM1	carboxypeptidase X (M14 family), member 1 [Source:HGNC Symbol;Acc:15771]	-2.756061792	3.164023204	28.58102284	8.99E-08	3.11E-06	4.160023991	3.845056765	-0.66302287	1.314027603	3.194574245
ENSG00000198176	TFDP1	transcription factor Dp-1 [Source:HGNC Symbol;Acc:11749]	-1.745000451	6.177792936	28.54952715	9.13E-08	3.15E-06	6.744892565	6.953514507	5.191663542	5.022263589	5.940650325
ENSG00000234819	RP11-336N8.4		-2.668697459	2.333097675	28.53459908	9.20E-08	3.17E-06	3.583106845	2.819716087	-0.215316613	0.074465887	1.982488568
ENSG00000108244	KRT23	keratin 23 (histone deacetylase inducible) [Source:HGNC Symbol;Acc:6438]	3.903213589	0.637213661	28.41007129	9.82E-08	3.37E-06	-1.608074432	-1.394066322	-0.215316613	1.948600359	1.044483142
ENSG00000224259	LINC01133	long intergenic non-protein coding RNA 1133 [Source:HGNC Symbol;Acc:49447]	-5.260919017	1.140489522	28.34817646	1.01E-07	3.47E-06	2.521050858	1.997369264	-4.621297774	-7.002070102	-0.095747566
ENSG00000111602	TIMELESS	timeless circadian clock [Source:HGNC Symbol;Acc:11813]	-1.749997498	5.491577766	28.3025734	1.04E-07	3.55E-06	6.136752081	6.062508275	4.300507637	4.146978108	5.645377617
ENSG00000216775	RP1-152L7.5		-2.888665085	1.727031865	28.28214972	1.05E-07	3.58E-06	2.673662624	2.477541651	-1.57307192	-1.042179808	1.802296017
ENSG00000183625	CCR3	chemokine (C-C motif) receptor 3 [Source:HGNC Symbol;Acc:1604]	2.9998543	0.103348136	28.18115836	1.10E-07	3.77E-06	-1.237502295	-1.191564836	0.602113338	0.556835562	0.341656497

ENSG00000197256	KANK2	KN motif and ankyrin repeat domains 2 [Source:HGNC Symbol;Acc:29300]	-2.347987641	3.921557626	28.17210584	1.11E-07	3.78E-06	4.618813883	4.890006675	1.304500245	2.329013491	3.779126901
ENSG00000225356	RP11-433O3.1		-3.924028996	0.601238702	28.05076094	1.18E-07	4.01E-06	1.908638473	1.523383318	-3.766908349	-3.888428289	-0.9858258
ENSG00000156140	ADAMTS3	ADAM metallopeptidase with thrombospondin type 1 motif, 3 [Source:HGNC Symbol;Acc:219]	-3.632548263	1.330156829	28.02665487	1.20E-07	4.06E-06	2.496700121	2.295753104	-2.845623393	-3.888428289	0.584199832
ENSG00000211645	IGLV1-50	immunoglobulin lambda variable 1-50 (non-functional) [Source:HGNC Symbol;Acc:5881]	-3.318923052	1.597409448	28.00240914	1.21E-07	4.10E-06	2.791786669	1.912023327	-1.886652327	-2.670141381	1.908000984
ENSG00000164879	CA3	carbonic anhydrase III, muscle specific [Source:HGNC Symbol;Acc:1374]	-3.619983365	1.784552296	27.97699521	1.23E-07	4.14E-06	2.332130562	3.286151722	-2.539967122	-3.888428289	0.872340852
ENSG00000171772	SYCE1	synaptonemal complex central element protein 1 [Source:HGNC Symbol;Acc:28852]	3.595669334	0.085986365	27.92999752	1.26E-07	4.24E-06	-1.756270337	-1.507080648	-0.520331194	1.39816257	0.04992943
ENSG00000180573	HIST1H2AC	histone cluster 1, H2ac [Source:HGNC Symbol;Acc:4733]	2.405598976	5.798357322	27.88326526	1.29E-07	4.33E-06	4.080332569	4.447875012	5.658180351	6.988468895	5.948414135
ENSG00000258445	RP11-307P22.1		-2.340624379	3.09800257	27.84778322	1.31E-07	4.41E-06	4.128673657	3.721949827	1.450281721	0.527247606	2.938038295
ENSG00000183098	GPC6	glypican 6 [Source:HGNC Symbol;Acc:4454]	-5.141621216	0.102007709	27.82675646	1.33E-07	4.44E-06	1.32517403	1.289608822	-4.621297774	-7.002070102	-4.610216385
ENSG00000254709	IGLL5	immunoglobulin lambda-like polypeptide 5 [Source:HGNC Symbol;Acc:38476]	-2.733894646	2.594151788	27.79461939	1.35E-07	4.51E-06	3.617390898	2.658749679	-0.329705368	-0.296766077	3.285646864
ENSG00000140937	CDH11	cadherin 11, type 2, OB-cadherin (osteoblast) [Source:HGNC Symbol;Acc:1750]	-8.963791489	0.510440861	27.76955549	1.37E-07	4.56E-06	2.167064212	1.16603322	-7.002070102	-7.002070102	-3.754655114
ENSG00000168078	PBK	PDZ binding kinase [Source:HGNC Symbol;Acc:18282]	-2.396359137	2.775804737	27.4142669	1.64E-07	5.47E-06	3.909466259	3.570041503	0.505333341	0.642123966	2.018338105
ENSG00000109265	KIAA1211	KIAA1211 [Source:HGNC Symbol;Acc:29219]	-2.619777054	3.260919612	27.38319658	1.67E-07	5.54E-06	4.183097611	4.13363576	-0.329705368	1.523337554	3.04038489
ENSG00000067048	DDX3Y	DEAD (Asp-Glu-Ala-Asp) box helicase 3, Y-linked [Source:HGNC Symbol;Acc:2699]	7.427643273	6.002986998	27.38293313	1.67E-07	5.54E-06	-0.250832646	0.095631612	-0.390491059	7.6412226	6.88338704
ENSG00000127920	GNG11	guanine nucleotide binding protein (G protein), gamma 11 [Source:HGNC Symbol;Acc:4403]	2.697581591	4.946711885	27.37534465	1.68E-07	5.55E-06	2.6444045	3.356486108	4.470369175	6.089437782	5.559778477
ENSG00000163535	SGOL2	shugoshin-like 2 (S. pombe) [Source:HGNC Symbol;Acc:30812]	-1.981350224	3.994686598	27.34116976	1.71E-07	5.64E-06	4.706360385	4.693025375	2.34945221	2.303059008	4.121640215
ENSG00000157168	NRG1	neuregulin 1 [Source:HGNC Symbol;Acc:7997]	2.503944362	3.874552093	27.25578386	1.78E-07	5.88E-06	0.597320328	1.418980604	2.56487414	3.946411867	5.573900552
ENSG00000162407	PPAP2B	phosphatidic acid phosphatase type 2B [Source:HGNC Symbol;Acc:9229]	-2.718831022	2.119977312	27.24707727	1.79E-07	5.90E-06	3.462559037	2.916017525	-0.66302287	-0.406691783	0.551948963
ENSG00000119865	CNRIP1	cannabinoid receptor interacting protein 1 [Source:HGNC Symbol;Acc:24546]	-3.113106038	3.918335105	27.07541974	1.96E-07	6.43E-06	5.133625684	4.707344697	-0.99931534	1.914722448	3.290541751
ENSG00000170522	ELOVL6	ELOVL fatty acid elongase 6 [Source:HGNC Symbol;Acc:15829]	-2.029044029	4.260465503	27.06032373	1.97E-07	6.47E-06	5.158416133	4.95944769	3.286590651	2.249705807	3.827080113
ENSG00000138411	HECW2	HECT, C2 and WW domain containing E3 ubiquitin protein ligase 2 [Source:HGNC Symbol;Acc:29853]	3.382835494	0.189184233	27.01487699	2.02E-07	6.61E-06	-1.608074432	-1.629706251	-0.059127673	1.36509501	0.303422373
ENSG00000205639	MFSD2B	major facilitator superfamily domain containing 2B [Source:HGNC Symbol;Acc:37207]	-2.488078634	4.007209093	27.01061722	2.02E-07	6.61E-06	4.760202192	5.076838692	1.076464519	2.551085884	3.490962768
ENSG00000175746	C15orf54	chromosome 15 open reading frame 54 [Source:HGNC Symbol;Acc:33797]	3.691270113	0.281778527	26.90131288	2.14E-07	6.99E-06	-2.322346157	-0.932821174	0.471574783	1.745170531	-1.70800759
ENSG00000130770	ATPIF1	ATPase inhibitory factor 1 [Source:HGNC Symbol;Acc:871]	-1.737096194	6.207730144	26.87490682	2.17E-07	7.07E-06	6.990648113	6.828810736	5.345190169	5.073060563	5.757453315
ENSG00000112936	C7	complement component 7 [Source:HGNC Symbol;Acc:1346]	-5.111235137	0.702176071	26.84990598	2.20E-07	7.15E-06	2.071376358	1.773719635	-4.621297774	-7.002070102	-2.832686105
ENSG00000007541	PIGQ	phosphatidylinositol glycan anchor biosynthesis, class Q [Source:HGNC Symbol;Acc:14135]	-1.698979848	6.396892099	26.76155709	2.30E-07	7.47E-06	7.025108654	7.152682415	5.518446459	5.403344377	5.914729217
ENSG00000165152	TMEM246	transmembrane protein 246 [Source:HGNC Symbol;Acc:28180]	-2.91531355	2.171519986	26.75432665	2.31E-07	7.48E-06	3.420029142	2.932364153	-2.845623393	-0.406691783	1.580441325
ENSG00000203999	RP11-290F20.1		3.980501681	1.250520337	26.70354975	2.37E-07	7.67E-06	-1.47369337	-2.475439898	-1.57307192	1.937395883	2.800681624
ENSG00000248323	LUCAT1	lung cancer associated transcript 1 (non-protein coding) [Source:HGNC Symbol;Acc:48498]	4.430500279	1.547395022	26.64811546	2.44E-07	7.88E-06	-0.942926472	-1.763731145	-0.453950945	2.947141733	2.3444256
ENSG00000159189	C1QC	complement component 1, q subcomponent, C chain [Source:HGNC Symbol;Acc:1245]	-2.895027602	1.945464255	26.62791831	2.47E-07	7.94E-06	3.326414242	2.361613919	-0.66302287	-1.042179808	1.428856327
ENSG00000225342	AC079630.4		3.946202256	-0.424353064	26.60167411	2.50E-07	8.04E-06	-3.79890385	-1.91149394	-0.907612816	0.369457657	0.378903451
ENSG00000112118	MCM3	minichromosome maintenance complex component 3 [Source:HGNC Symbol;Acc:6945]	-1.831836807	6.659171465	26.5903903	2.51E-07	8.07E-06	7.322478468	7.301680528	5.841151855	5.044626073	6.598170193

ENSG00000100350	FOXRED2	FAD-dependent oxidoreductase domain containing 2 [Source:HGNC Symbol;Acc:26264]	-2.014041535	3.770194571	26.54242008	2.58E-07	8.26E-06	4.52961579	4.595461025	2.507726234	1.75796486	3.532875847
ENSG00000175305	CCNE2	cyclin E2 [Source:HGNC Symbol;Acc:1590]	-2.240269143	2.954012939	26.49221612	2.65E-07	8.46E-06	3.449931193	3.75908114	0.6928072	0.669468625	3.430160334
ENSG00000171385	KCND3	potassium voltage-gated channel, Shal-related subfamily, member 3 [Source:HGNC Symbol;Acc:6239]	3.270711272	0.620630574	26.44791039	2.71E-07	8.64E-06	-0.942926472	-1.289265519	0.210162819	1.856429106	0.763888603
ENSG00000224607	IGKV1D-27	immunoglobulin kappa variable 1D-27 (pseudogene) [Source:HGNC Symbol;Acc:5751]	-3.903529313	1.150530636	26.34044641	2.86E-07	9.12E-06	2.688072143	1.403429499	-2.539967122	-4.730697034	0.615745481
ENSG00000164087	POC1A	POC1 centriolar protein A [Source:HGNC Symbol;Acc:24488]	-2.184476768	3.174676464	26.32689048	2.88E-07	9.17E-06	4.001759993	3.918010484	1.007464306	1.331251704	3.199787516
ENSG00000197496	SLC2A10	solute carrier family 2 (facilitated glucose transporter), member 10 [Source:HGNC Symbol;Acc:13444]	-4.448229266	1.041863982	26.26899878	2.97E-07	9.43E-06	2.37729083	1.85599892	-7.002070102	-3.888428289	0.139468177
ENSG00000181856	SLC2A4	solute carrier family 2 (facilitated glucose transporter), member 4 [Source:HGNC Symbol;Acc:11009]	-5.037181025	0.913214285	26.2447832	3.01E-07	9.53E-06	1.780020869	2.107040151	-4.621297774	-7.002070102	0.378903451
ENSG00000075702	WDR62	WD repeat domain 62 [Source:HGNC Symbol;Acc:24502]	-2.056640528	3.609950066	26.2393016	3.02E-07	9.54E-06	4.386750265	4.16855421	1.907011864	1.567613022	3.921613159
ENSG00000167536	DHRS13	dehydrogenase/reductase (SDR family) member 13 [Source:HGNC Symbol;Acc:28326]	-1.722400193	4.837734553	26.17285748	3.12E-07	9.86E-06	5.525365311	5.600352436	3.95448688	3.628851426	4.338041939
ENSG00000128322	IGLL1	immunoglobulin lambda-like polypeptide 1 [Source:HGNC Symbol;Acc:5870]	-2.22947271	3.344133474	26.16121436	3.14E-07	9.90E-06	3.621150429	3.086385644	0.505333341	0.61425099	4.745235504
ENSG00000136840	ST6GALNAC4	ST6 (alpha-N-acetyl-neuraminyl-2,3-beta-galactosyl-1,3)-N-acetylgalactosaminide alpha-2,6-sialyltransferase 4 [Source:HGNC Symbol;Acc:17846]	-1.801489658	5.158796639	26.14395789	3.17E-07	9.97E-06	5.854049734	5.973159515	4.384539572	3.642714894	4.600983593
ENSG00000166793	YPEL4	yippee-like 4 (Drosophila) [Source:HGNC Symbol;Acc:18328]	-2.521842763	2.796789332	26.10720568	3.23E-07	1.01E-05	3.449931193	3.987451996	1.379231566	-0.099242291	2.030092686
ENSG00000151650	VENTX	VENT homeobox [Source:HGNC Symbol;Acc:13639]	2.700805161	4.242916055	26.07498989	3.28E-07	1.03E-05	1.968888231	2.825562359	3.939130343	5.59200618	4.306975745
ENSG00000111837	MAK	male germ cell-associated kinase [Source:HGNC Symbol;Acc:6816]	3.841411087	1.12629384	25.98450471	3.44E-07	1.08E-05	-0.942926472	-1.289265519	-0.390491059	2.50629229	1.702990823
ENSG00000237075	RP11-497H16.2		4.825767864	3.458404887	25.9730183	3.46E-07	1.08E-05	-0.14487434	0.376157587	1.205290011	5.493125	2.334979851
ENSG00000137699	TRIM29	tripartite motif containing 29 [Source:HGNC Symbol;Acc:17274]	-5.124709048	0.402168791	25.95861091	3.49E-07	1.09E-05	1.696960037	1.464652754	-7.002070102	-4.730697034	-2.060109799
ENSG00000104147	OIP5	Opa interacting protein 5 [Source:HGNC Symbol;Acc:20300]	-2.611617678	2.134619797	25.92879715	3.54E-07	1.10E-05	3.241034999	2.831385036	-0.589913612	-0.797916568	1.945725455
ENSG00000230481	IGKV1OR22-5	immunoglobulin kappa variable 1/OR22-5 (pseudogene) [Source:HGNC Symbol;Acc:5773]	-8.720518914	0.356772217	25.92032862	3.56E-07	1.11E-05	1.623891846	1.202433699	-7.002070102	-7.002070102	-0.506781561
ENSG00000260903	XKR7	XK, Kell blood group complex subunit-related family, member 7 [Source:HGNC Symbol;Acc:23062]	3.078008124	-0.02086228	25.82423731	3.74E-07	1.16E-05	-1.47369337	-1.191564836	-0.215316613	0.894716898	0.182236344
ENSG00000136859	ANGPTL2	angiopoietin-like 2 [Source:HGNC Symbol;Acc:490]	-8.079867731	-0.350073707	25.80516498	3.78E-07	1.17E-05	1.018120824	0.555709266	-7.002070102	-7.002070102	-3.221241555
ENSG00000196230	TUBB	tubulin, beta class I [Source:HGNC Symbol;Acc:20778]	-2.276798478	8.914288153	25.79301573	3.80E-07	1.17E-05	9.669927692	9.640336798	7.505547211	7.543462522	8.751957553
ENSG00000135916	ITM2C	integral membrane protein 2C [Source:HGNC Symbol;Acc:6175]	-1.944054769	5.941476481	25.79144446	3.80E-07	1.17E-05	6.608088569	6.392917765	5.005158829	3.886500298	6.295025729
ENSG00000140451	PIF1	PIF1 5'-to-3' DNA helicase [Source:HGNC Symbol;Acc:26220]	-2.2286959	3.001324092	25.76066825	3.86E-07	1.19E-05	3.984351473	3.442696095	1.076464519	0.847612951	3.194574245
ENSG00000075618	FSCN1	fascin homolog 1, actin-bundling protein (Strongylocentrotus purpuratus) [Source:HGNC Symbol;Acc:11148]	-1.981326776	3.895344279	25.74714057	3.89E-07	1.20E-05	4.763607814	4.399701841	2.404162378	2.096958951	4.025090588
ENSG00000172236	TPSAB1	tryptase alpha/beta 1 [Source:HGNC Symbol;Acc:12019]	-4.266043637	3.744238733	25.74354533	3.90E-07	1.20E-05	5.797857325	2.948527637	-0.390491059	1.0274492	-0.726511138
ENSG00000253705	IGHV3-41	immunoglobulin heavy variable 3-41 (pseudogene) [Source:HGNC Symbol;Acc:5602]	-3.801571891	0.674870021	25.73231721	3.92E-07	1.20E-05	1.945089405	1.479561055	-3.766908349	-3.888428289	-0.147769603
ENSG00000049759	NEDD4L	neural precursor cell expressed, developmentally down-regulated 4-like, E3 ubiquitin protein ligase [Source:HGNC Symbol;Acc:7728]	-1.944112511	3.792474293	25.73140846	3.92E-07	1.20E-05	4.519579521	4.530729604	2.413082378	2.475638093	3.589580856
ENSG00000123119	NECAB1	N-terminal EF-hand calcium binding protein 1 [Source:HGNC Symbol;Acc:20983]	-3.455996593	1.413140854	25.6604304	4.07E-07	1.24E-05	2.636996308	2.329059307	-4.621297774	-2.419096061	0.615745481

ENSG00000135929	CYP27A1	cytochrome P450, family 27, subfamily A, polypeptide 1 [Source:HGNC Symbol;Acc:2605]	2.463366205	5.653431562	25.58830105	4.23E-07	1.29E-05	3.528102002	4.505458803	5.445328656	6.844445403	5.873730193
ENSG00000063241	ISOC2	isochorismatase domain containing 2 [Source:HGNC Symbol;Acc:26278]	-1.843133957	4.240193447	25.56859294	4.27E-07	1.30E-05	5.138880244	4.903888565	3.180883743	2.824787032	3.68560772
ENSG00000134121	CHL1	cell adhesion molecule L1-like [Source:HGNC Symbol;Acc:1939]	-2.838974589	1.797521294	25.46008212	4.52E-07	1.37E-05	2.80506858	2.813846027	-2.845623393	-0.7248947	1.198257616
ENSG00000233864	TTTY15	testis-specific transcript, Y-linked 15 (non-protein coding) [Source:HGNC Symbol;Acc:18567]	5.388786505	2.072000589	25.38985505	4.68E-07	1.42E-05	-2.108017771	-2.076136033	-4.621297774	3.225676865	3.447795505
ENSG00000271375	RP11-295P22.2		-3.98472658	0.746968763	25.37542733	4.72E-07	1.43E-05	2.332130562	1.255368251	-3.233942493	-4.730697034	-0.894110176
ENSG00000258648	UBE2CP1	ubiquitin-conjugating enzyme E2C pseudogene 1 [Source:HGNC Symbol;Acc:19670]	-3.00643398	1.12804579	25.34618751	4.79E-07	1.45E-05	2.026722384	2.068117593	-2.073364206	-2.419096061	0.948644093
ENSG00000171798	KNDC1	kinase non-catalytic C-lobe domain (KIND) containing 1 [Source:HGNC Symbol;Acc:29374]	2.617598211	2.301052942	25.30456815	4.90E-07	1.48E-05	0.566455146	1.551875699	2.694908712	3.42334388	1.446507348
ENSG00000232528	RP4-673D20.3		3.426382032	-0.235454482	25.28146085	4.95E-07	1.49E-05	-1.921450438	-2.262017103	-0.740035668	0.823471408	0.182236344
ENSG00000241527	CA15P1	carbonic anhydrase XV, pseudogene 1 [Source:HGNC Symbol;Acc:44364]	4.046877262	-0.734971712	25.23441054	5.08E-07	1.53E-05	-2.879434292	-3.940353384	-2.073364206	0.301169826	0.095393329
ENSG00000230910	RP3-525N10.2		3.855758604	1.091552689	25.21998122	5.11E-07	1.53E-05	-2.108017771	-0.30308518	1.076464519	2.724061344	-0.80787826
ENSG00000086506	HBQ1	hemoglobin, theta 1 [Source:HGNC Symbol;Acc:4833]	-2.845285833	1.287983618	25.18557765	5.21E-07	1.56E-05	2.463579632	1.965953212	-1.202308962	-1.231517591	0.706443614
ENSG00000188153	COL4A5	collagen, type IV, alpha 5 [Source:HGNC Symbol;Acc:2207]	-4.364562814	0.808049875	25.1700016	5.25E-07	1.57E-05	2.247099092	1.832965453	-3.766908349	-7.002070102	-4.610216385
ENSG00000127954	STEAP4	STEAP family member 4 [Source:HGNC Symbol;Acc:21923]	2.108407791	4.89298552	25.06944102	5.53E-07	1.65E-05	2.973344712	3.520443902	4.51455366	5.376632009	6.013512509
ENSG00000090013	BLVRB	biliverdin reductase B (flavin reductase (NADPH)) [Source:HGNC Symbol;Acc:1063]	-2.051603014	6.795554803	25.05850202	5.56E-07	1.66E-05	7.546344528	7.508663577	4.927912984	5.930406088	6.564719069
ENSG00000138792	ENPEP	glutamyl aminopeptidase (aminopeptidase A) [Source:HGNC Symbol;Acc:3355]	-4.960793037	0.512691899	25.04805894	5.59E-07	1.66E-05	1.752861822	1.711936244	-4.621297774	-7.002070102	-3.221241555
ENSG00000130558	OLFM1	olfactomedin 1 [Source:HGNC Symbol;Acc:17187]	3.271426376	1.97652044	25.0246624	5.66E-07	1.68E-05	0.000762307	0.836121102	1.613958159	3.520398046	1.021113618
ENSG00000106070	GRB10	growth factor receptor-bound protein 10 [Source:HGNC Symbol;Acc:4564]	-1.97330756	4.175734033	25.01987615	5.67E-07	1.68E-05	4.927426575	4.888611111	2.430758609	3.070221344	4.019204856
ENSG00000138180	CEP55	centrosomal protein 55kDa [Source:HGNC Symbol;Acc:1161]	-2.160508027	2.980377083	24.98610912	5.77E-07	1.71E-05	3.628640226	3.419690528	0.805492218	0.466186565	3.700395796
ENSG00000119326	CTNNAL1	catenin (cadherin-associated protein), alpha-like 1 [Source:HGNC Symbol;Acc:2512]	-2.303981083	3.574199504	24.97394855	5.81E-07	1.72E-05	4.432399102	4.385920009	0.9100098	1.959718487	3.456532906
ENSG00000248810	RP11-362F19.1		2.636782858	2.752959213	24.94813148	5.89E-07	1.74E-05	1.018120824	1.809558268	3.11133962	4.009294922	1.643016926
ENSG00000129824	RPS4Y1	ribosomal protein S4, Y-linked 1 [Source:HGNC Symbol;Acc:10425]	5.802949069	4.921510214	24.93807215	5.92E-07	1.74E-05	0.254194054	-0.112176905	-0.329705368	6.159216549	6.264875363
ENSG00000257878	RP11-256L6.3		3.711416657	-0.012077043	24.89457782	6.06E-07	1.78E-05	-1.47369337	-2.726009782	-0.99931534	1.224680533	0.415212929
ENSG00000235513	RP4-756G23.5		3.777929101	0.070631397	24.82738026	6.27E-07	1.84E-05	-2.879434292	-1.289265519	-0.740035668	1.314027603	0.415212929
ENSG00000123700	KCNJ2	potassium inwardly-rectifying channel, subfamily J, member 2 [Source:HGNC Symbol;Acc:6263]	2.948508541	2.58930131	24.8221538	6.29E-07	1.84E-05	0.435934254	-0.583862591	1.703933103	2.622783341	4.206962975
ENSG00000253525	CTD-2114J12.1		-4.956820244	0.334048638	24.74130661	6.56E-07	1.92E-05	1.623891846	1.387708937	-7.002070102	-4.730697034	-2.060109799
ENSG00000106546	AHR	aryl hydrocarbon receptor [Source:HGNC Symbol;Acc:348]	2.366773403	6.676855818	24.67941757	6.77E-07	1.98E-05	4.851046282	5.568459271	6.6538419	8.03154189	6.181690116
ENSG00000139514	SLC7A1	solute carrier family 7 (cationic amino acid transporter, y+ system), member 1 [Source:HGNC Symbol;Acc:11057]	-1.7027906	5.793718559	24.65763201	6.85E-07	2.00E-05	6.274420125	6.662803985	5.064392409	4.388262566	5.456852822
ENSG00000237781	RP11-54A4.2		3.517598461	1.094318728	24.63131484	6.94E-07	2.02E-05	-1.132483902	-0.713501401	-0.215316613	2.36290873	1.732068029
ENSG00000109685	WHSC1	Wolf-Hirschhorn syndrome candidate 1 [Source:HGNC Symbol;Acc:12766]	-1.661843599	6.752684289	24.55010779	7.24E-07	2.10E-05	7.20777661	7.480686038	5.927728104	5.667691125	6.654312605
ENSG00000100181	TPTEP1	transmembrane phosphatase with tensin homology pseudogene 1 [Source:HGNC Symbol;Acc:43648]	3.814001435	4.056845149	24.52107855	7.35E-07	2.13E-05	0.714573228	1.889873947	2.709464044	5.625553375	4.436279042
ENSG00000213318	RP11-331F4.1		5.876950059	4.960227185	24.48830534	7.48E-07	2.17E-05	-0.094677427	-0.252938016	-0.010608983	6.090070331	6.401234604
ENSG00000103257	SLC7A5	solute carrier family 7 (amino acid transporter light chain, L system), member 5 [Source:HGNC Symbol;Acc:11063]	-1.618637107	5.299956568	24.4257039	7.72E-07	2.23E-05	5.830698698	6.035018876	4.340799889	3.923880795	5.276169588
ENSG00000186354	C9orf47	chromosome 9 open reading frame 47 [Source:HGNC Symbol;Acc:23669]	4.281677283	1.540853488	24.40147443	7.82E-07	2.26E-05	-0.775407551	-1.394066322	-0.161352853	3.284338193	1.3928929
ENSG00000137563	GGH	gamma-glutamyl hydrolase (conjugase, folylpolygammaglutamyl hydrolase) [Source:HGNC Symbol;Acc:4248]	-2.148644577	3.232620037	24.31682337	8.17E-07	2.36E-05	4.139199645	3.69346042	1.142314722	1.12943269	3.557451401

ENSG00000179588	ZFPM1	zinc finger protein, FOG family member 1 [Source:HGNC Symbol;Acc:19762]	-1.670597452	4.765375267	24.20431003	8.66E-07	2.49E-05	5.538346965	5.459885251	3.816750433	3.632329802	4.224973246
ENSG00000108576	SLC6A4	solute carrier family 6 (neurotransmitter transporter), member 4 [Source:HGNC Symbol;Acc:11050]	3.39948568	0.312836465	24.19674309	8.70E-07	2.50E-05	-2.108017771	-0.408932621	0.210162819	1.348272594	0.303422373
ENSG00000237249	RP1-102G20.2		-3.978760376	0.614147193	24.18610058	8.75E-07	2.51E-05	1.414540624	1.867379203	-7.002070102	-3.360122781	-0.147769603
ENSG00000135045	C9orf40	chromosome 9 open reading frame 40 [Source:HGNC Symbol;Acc:23433]	-2.085009129	3.025719754	24.17174153	8.81E-07	2.52E-05	3.98142952	3.743725731	1.732719555	1.069111613	2.537970241
ENSG00000112175	BMP5	bone morphogenetic protein 5 [Source:HGNC Symbol;Acc:1072]	-8.954581938	0.39321376	24.16524451	8.84E-07	2.53E-05	2.071376358	1.03087254	-7.002070102	-7.002070102	-7.002070102
ENSG00000173210	ABLIM3	actin binding LIM protein family, member 3 [Source:HGNC Symbol;Acc:29132]	2.592074784	3.304193947	24.1533364	8.90E-07	2.54E-05	1.287817526	1.986973075	3.072456242	4.479923268	3.581615324
ENSG00000127252	HRASLS	HRAS-like suppressor [Source:HGNC Symbol;Acc:14922]	3.724260427	-0.4427703	24.14602941	8.93E-07	2.54E-05	-2.108017771	-2.475439898	-2.539967122	0.722650538	0.223773082
ENSG00000174776	WDR49	WD repeat domain 49 [Source:HGNC Symbol;Acc:26587]	3.558889903	1.259333563	24.13695127	8.97E-07	2.55E-05	-0.775407551	-0.408932621	0.168628767	2.794059013	1.177268129
ENSG00000163251	FZD5	frizzled family receptor 5 [Source:HGNC Symbol;Acc:4043]	-2.339116399	2.880626113	24.10498908	9.12E-07	2.59E-05	3.54006658	3.765177832	0.168628767	1.261086007	2.956635145
ENSG00000159713	TPPP3	tubulin polymerization-promoting protein family member 3 [Source:HGNC Symbol;Acc:24162]	2.722631076	3.553862179	24.0726972	9.28E-07	2.63E-05	1.306616686	2.261659784	3.334328805	4.923509977	3.421261246
ENSG00000180190	TDRP	testis development related protein [Source:HGNC Symbol;Acc:26951]	3.722397465	1.221698542	24.03142525	9.48E-07	2.68E-05	-1.921450438	-0.025429826	0.401591237	2.60872607	1.428856327
ENSG00000164109	MAD2L1	MAD2 mitotic arrest deficient-like 1 (yeast) [Source:HGNC Symbol;Acc:6763]	-2.024149889	3.400532977	23.96434716	9.81E-07	2.77E-05	4.321238306	4.138340674	2.253389193	1.224680533	2.962781202
ENSG00000113810	SMC4	structural maintenance of chromosomes 4 [Source:HGNC Symbol;Acc:14013]	-1.633459198	6.688061311	23.933113	9.97E-07	2.82E-05	7.213079789	7.35368656	5.829538813	5.382840447	6.71063621
ENSG00000113231	PDE8B	phosphodiesterase 8B [Source:HGNC Symbol;Acc:8794]	2.823368066	0.066827687	23.92462344	1.00E-06	2.82E-05	-1.350769713	-0.713501401	0.210162819	0.847612951	0.002986071
ENSG00000198732	SMOC1	SPARC related modular calcium binding 1 [Source:HGNC Symbol;Acc:20318]	-3.675858669	0.974377863	23.88382492	1.02E-06	2.88E-05	2.446729819	1.85599892	-7.002070102	-2.670141381	-1.87334355
ENSG00000099377	HSD3B7	hydroxy-delta-5-steroid dehydrogenase, 3 beta- and steroid delta-isomerase 7 [Source:HGNC Symbol;Acc:18324]	-2.320076411	2.356844948	23.868083	1.03E-06	2.89E-05	3.437191842	3.086385644	0.168628767	0.556835562	1.760570749
ENSG00000131462	TUBG1	tubulin, gamma 1 [Source:HGNC Symbol;Acc:12417]	-2.093337992	3.078906016	23.86901015	1.03E-06	2.89E-05	3.906388466	4.026044706	1.32354911	1.745170531	2.257101262
ENSG00000186871	ERCC6L	excision repair cross-complementing rodent repair deficiency, complementation group 6-like [Source:HGNC Symbol;Acc:20794]	-2.47935499	2.665429083	23.79961486	1.07E-06	2.99E-05	3.639802517	3.576989707	1.184602189	-1.231517591	2.109797605
ENSG00000048140	TSPAN17	tetraspanin 17 [Source:HGNC Symbol;Acc:13594]	-1.602213184	6.391783173	23.80139073	1.07E-06	2.99E-05	7.037470417	7.096973808	5.467976758	5.545720136	5.913936412
ENSG00000174837	EMR1	egf-like module containing, mucin-like, hormone receptor-like 1 [Source:HGNC Symbol;Acc:3336]	2.028312223	5.504134532	23.76056894	1.09E-06	3.04E-05	3.733087128	4.72152329	5.660991275	6.480992576	5.555718077
ENSG00000272839	RP11-452C13.1		4.017188794	0.047412644	23.69828292	1.13E-06	3.14E-05	-2.879434292	-1.629706251	-1.097244829	1.27894994	0.615745481
ENSG00000226091	LINC00937	long intergenic non-protein coding RNA 937 [Source:HGNC Symbol;Acc:48629]	2.981106411	3.922047042	23.66063205	1.15E-06	3.20E-05	1.431768352	2.477541651	3.380537789	5.450411635	3.707733377
ENSG00000258944	RP4-647C14.3		3.050788333	1.468964982	23.57705669	1.20E-06	3.33E-05	-0.196880952	-0.30308518	1.225685365	2.717526241	1.564363944
ENSG00000138080	EMILIN1	elastin microfibril interfacer 1 [Source:HGNC Symbol;Acc:19880]	-2.11313037	2.758568233	23.57293651	1.20E-06	3.34E-05	3.376207305	3.667655396	1.142314722	0.642123966	2.737560122
ENSG00000106341	PPP1R17	protein phosphatase 1, regulatory subunit 17 [Source:HGNC Symbol;Acc:16973]	2.755638924	-0.12308817	23.56087258	1.21E-06	3.35E-05	-1.237502295	-0.713501401	0.437007322	0.335717742	-0.57637152
ENSG00000108846	ABCC3	ATP-binding cassette, sub-family C (CFTR/MRP), member 3 [Source:HGNC Symbol;Acc:54]	2.542799409	5.019566108	23.40648646	1.31E-06	3.63E-05	2.70942011	3.918010484	4.749255852	6.380638865	4.906969078
ENSG00000204169	AGAP7	ArfGAP with GTPase domain, ankyrin repeat and PH domain 7 [Source:HGNC Symbol;Acc:23465]	4.142520822	1.054243487	23.39049069	1.32E-06	3.65E-05	-1.756270337	-0.583862591	-0.66302287	2.818693563	0.485200289
ENSG00000168874	ATOH8	atonal homolog 8 (Drosophila) [Source:HGNC Symbol;Acc:24126]	3.170972186	1.401428706	23.35707975	1.35E-06	3.71E-05	-0.250832646	-0.408932621	0.210162819	2.436381066	2.257101262
ENSG00000118520	ARG1	arginase 1 [Source:HGNC Symbol;Acc:663]	-2.862413338	2.831085295	23.2333335	1.43E-06	3.95E-05	3.402659809	1.900991143	-0.99931534	-1.336401299	4.335675838
ENSG00000090889	KIF4A	kinesin family member 4A [Source:HGNC Symbol;Acc:13339]	-2.771453332	1.646309731	23.19602361	1.46E-06	4.02E-05	2.521050858	2.528610122	-1.886652327	-2.205328685	1.746389777
ENSG00000198336	MYL4	myosin, light chain 4, alkali; atrial, embryonic [Source:HGNC Symbol;Acc:7585]	-2.251143083	4.018968197	23.18577214	1.47E-06	4.03E-05	4.725696366	5.220204443	1.613958159	2.794059013	2.800681624
ENSG00000184551	AC132872.1		3.973387113	0.619101761	23.16434907	1.49E-06	4.07E-05	-1.350769713	-1.629706251	-1.097244829	2.258736156	0.485200289
ENSG00000129988	LBP	lipopolysaccharide binding protein [Source:HGNC Symbol;Acc:6517]	-5.216586289	0.731192571	23.15386579	1.50E-06	4.09E-05	2.47193131	1.16603322	-4.621297774	-7.002070102	-2.526883755

ENSG00000237803	LINC00211	long intergenic non-protein coding RNA 211 [Source:HGNC Symbol;Acc:37459]	2.8109062	0.297360406	23.12611491	1.52E-06	4.14E-05	-1.034594152	-0.855949929	0.036330944	0.917704421	0.948644093
ENSG00000127831	VIL1	villin 1 [Source:HGNC Symbol;Acc:12690]	2.940102028	1.406434532	23.07379392	1.56E-06	4.25E-05	-0.625332215	0.376157587	1.076464519	2.379562322	1.895203721
ENSG00000128383	APOBEC3A	apolipoprotein B mRNA editing enzyme, catalytic polypeptide-like 3A [Source:HGNC Symbol;Acc:17343]	1.792936198	5.802631057	22.99551265	1.62E-06	4.42E-05	4.344166237	4.61584628	5.674964329	6.549744331	6.479408743
ENSG00000231650	RFESDP1	Rieske (Fe-S) domain containing pseudogene 1 [Source:HGNC Symbol;Acc:39420]	-2.867545425	1.193647409	22.97481702	1.64E-06	4.46E-05	2.266432806	2.068117593	-0.99931534	-3.360122781	0.615745481
ENSG00000114054	PCCB	propionyl CoA carboxylase, beta polypeptide [Source:HGNC Symbol;Acc:8654]	-1.624331815	5.283210088	22.93912385	1.67E-06	4.54E-05	6.051554497	5.930395455	4.206153886	4.320880041	4.8401004
ENSG00000137962	ARHGAP29	Rho GTPase activating protein 29 [Source:HGNC Symbol;Acc:30207]	2.877580066	0.50580046	22.86459229	1.74E-06	4.71E-05	-0.425964666	-0.782968247	0.328039018	1.538247531	0.415212929
ENSG00000100427	MLC1	megalencephalic leukoencephalopathy with subcortical cysts 1 [Source:HGNC Symbol;Acc:17082]	-2.024208285	5.814493165	22.85683689	1.75E-06	4.72E-05	6.761168998	6.333127059	3.914214735	4.849230022	5.58788573
ENSG00000102384	CENPI	centromere protein I [Source:HGNC Symbol;Acc:3968]	-2.338431996	2.155116432	22.79627344	1.80E-06	4.86E-05	3.145070257	2.948527637	-0.161352853	-0.46496394	1.920685729
ENSG00000230006	ANKRD36BP2	ankyrin repeat domain 36B pseudogene 2 [Source:HGNC Symbol;Acc:33607]	-1.991612219	3.418865823	22.78098243	1.82E-06	4.90E-05	4.440932708	4.227146839	2.439516168	1.348272594	2.247064062
ENSG00000012223	LTF	lactotransferrin [Source:HGNC Symbol;Acc:6720]	-4.56963865	6.6654719	22.76796936	1.83E-06	4.92E-05	7.999657807	4.846103827	0.505333341	3.306279995	7.723535322
ENSG00000100473	COCH	cochlin [Source:HGNC Symbol;Acc:2180]	-1.773809302	4.246339016	22.75793321	1.84E-06	4.94E-05	5.305852844	4.975312216	3.154531226	2.974805722	2.765955603
ENSG00000186567	CEACAM19	carcinoembryonic antigen-related cell adhesion molecule 19 [Source:HGNC Symbol;Acc:31951]	3.371723877	1.227233051	22.72101314	1.87E-06	5.03E-05	-0.942926472	-0.112176905	0.125863448	2.580193821	1.531661491
ENSG00000250138	RP11-848G14.5		2.803479628	4.704199763	22.69486022	1.90E-06	5.09E-05	2.537059301	2.79012399	3.679326777	6.0913346	5.183161802
ENSG00000175445	LPL	lipoprotein lipase [Source:HGNC Symbol;Acc:6677]	-2.610595599	2.378331791	22.66236867	1.93E-06	5.17E-05	3.890900236	2.975070752	-0.82139248	0.335717742	0.223773082
ENSG00000162747	FCGR3B	Fc fragment of IgG, low affinity IIIb, receptor (CD16b) [Source:HGNC Symbol;Acc:3620]	2.483380931	5.254498623	22.65088982	1.94E-06	5.19E-05	3.54006658	3.29881546	4.485248201	6.391456188	5.972219808
ENSG00000108821	COL1A1	collagen, type I, alpha 1 [Source:HGNC Symbol;Acc:2197]	-3.204610406	1.316466169	22.64113818	1.95E-06	5.21E-05	2.256798335	2.772072928	-2.539967122	-3.888428289	-2.060109799
ENSG00000111247	RAD51AP1	RAD51 associated protein 1 [Source:HGNC Symbol;Acc:16956]	-2.182059342	2.492121025	22.6031384	1.99E-06	5.31E-05	3.245913199	3.225538486	0.168628767	0.229488395	2.74471156
ENSG00000203813	HIST1H3H	histone cluster 1, H3h [Source:HGNC Symbol;Acc:4775]	2.533379049	2.301819326	22.55285735	2.04E-06	5.44E-05	1.084982398	1.07079906	2.292585253	3.419322554	2.185328638
ENSG00000140090	SLC24A4	solute carrier family 24 (sodium/potassium/calcium exchanger), member 4 [Source:HGNC Symbol;Acc:10978]	1.955594157	5.236080168	22.52103728	2.08E-06	5.52E-05	3.609842318	4.231556919	5.149585589	6.323150454	5.37856212
ENSG00000181908	AP003774.4	HCG1652096, isoform CRA_a; Uncharacterized protein; cDNA FLJ37045 fis, clone BRACE2012185 [Source:UniProtKB/TrEMBL;Acc:Q8N9J4]	2.855385977	1.04229536	22.5050633	2.10E-06	5.56E-05	-0.625332215	-0.523165112	0.721814282	1.981700954	1.627625634
ENSG00000110987	BCL7A	B-cell CLL/lymphoma 7A [Source:HGNC Symbol;Acc:1004]	-1.875806029	4.392182599	22.49539398	2.11E-06	5.58E-05	5.042641743	4.652487188	3.089249033	2.106994346	5.079506237
ENSG00000090104	RGS1	regulator of G-protein signaling 1 [Source:HGNC Symbol;Acc:9991]	-2.611373449	5.137281833	22.48410053	2.12E-06	5.60E-05	6.754766009	5.129775555	3.196467253	3.874800997	3.146787158
ENSG00000253957	IGHV3-22	immunoglobulin heavy variable 3-22 (pseudogene) [Source:HGNC Symbol;Acc:5587]	-3.29747528	1.112934432	22.47361724	2.13E-06	5.62E-05	2.651774847	1.355744383	-1.886652327	-3.360122781	0.415212929
ENSG00000249138	RP11-701P16.1		4.275801005	0.165228061	22.40023122	2.21E-06	5.83E-05	-1.921450438	-2.262017103	-1.886652327	1.692827451	0.415212929
ENSG00000074696	PTPLAD1	protein tyrosine phosphatase-like A domain containing 1 [Source:HGNC Symbol;Acc:24175]	-1.549984799	5.375308273	22.39074714	2.22E-06	5.85E-05	6.029091381	6.111185334	4.57938316	4.183001653	4.941611722
ENSG00000175216	CKAP5	cytoskeleton associated protein 5 [Source:HGNC Symbol;Acc:28959]	-1.601285603	6.264731902	22.354003	2.27E-06	5.96E-05	6.645948858	6.979311433	5.525661302	4.872947848	6.365727161
ENSG00000136010	ALDH1L2	aldehyde dehydrogenase 1 family, member L2 [Source:HGNC Symbol;Acc:26777]	-2.486207033	2.003061417	22.3373246	2.29E-06	6.00E-05	3.201403963	2.69752161	-0.66302287	-0.7248947	1.498200525
ENSG00000138821	SLC39A8	solute carrier family 39 (zinc transporter), member 8 [Source:HGNC Symbol;Acc:20862]	-1.785733587	5.701625697	22.28381494	2.35E-06	6.16E-05	6.476787366	6.405657863	5.158895582	4.038427289	5.083742254
ENSG00000162998	FRZB	frizzled-related protein [Source:HGNC Symbol;Acc:3959]	-8.745775965	0.317604185	22.22970684	2.42E-06	6.31E-05	1.752861822	1.220295226	-7.002070102	-7.002070102	-2.274700861
ENSG00000151090	THRB	thyroid hormone receptor, beta [Source:HGNC Symbol;Acc:11799]	-2.552787057	1.855193883	22.23158668	2.42E-06	6.31E-05	3.08644716	2.48494876	-0.10933503	-1.042179808	1.198257616

ENSG00000189060	H1F0	H1 histone family, member 0 [Source:HGNC Symbol;Acc:4714]	-1.677813385	6.465541572	22.23359054	2.41E-06	6.31E-05	6.651014597	6.921117846	4.784053235	5.39517774	7.231463478
ENSG00000135338	LCA5	Leber congenital amaurosis 5 [Source:HGNC Symbol;Acc:31923]	-2.68180137	1.491678138	22.21905164	2.43E-06	6.32E-05	2.629549878	2.401300588	-1.315629595	-2.0191864	0.706443614
ENSG00000154920	EME1	essential meiotic structure-specific endonuclease 1 [Source:HGNC Symbol;Acc:24965]	-2.350011069	1.968723778	22.21994416	2.43E-06	6.32E-05	2.591730062	2.759912177	-0.271377577	-0.7248947	2.334979851
ENSG00000267288	RP13-890H12.2		2.763976346	0.068928942	22.21906591	2.43E-06	6.32E-05	-1.132483902	-1.394066322	0.125863448	0.61425099	0.615745481
ENSG00000111886	GABRR2	gamma-aminobutyric acid (GABA) A receptor, rho 2 [Source:HGNC Symbol;Acc:4091]	2.871862	0.54772493	22.19840564	2.46E-06	6.38E-05	-1.034594152	-0.713501401	-0.215316613	1.242998101	1.446507348
ENSG00000211672	IGLV4-3	immunoglobulin lambda variable 4-3 [Source:HGNC Symbol;Acc:5919]	-3.689532627	0.894379886	22.17775864	2.49E-06	6.44E-05	2.636996308	1.147482679	-2.539967122	-4.730697034	-1.42520211
ENSG00000261253	AC137932.6		2.978782923	0.535829424	22.16544645	2.50E-06	6.47E-05	-1.237502295	-0.464918547	0.538319988	1.75796486	-0.045536284
ENSG00000253616	RP11-875O11.3		2.982861786	0.177717189	22.16269106	2.50E-06	6.47E-05	-1.132483902	-1.191564836	0.036330944	1.348272594	-0.147769603
ENSG00000257698	RP11-620J15.3		-2.150092686	3.34305507	22.14752913	2.52E-06	6.51E-05	4.35097416	3.873554127	1.245796404	1.959718487	3.189342067
ENSG00000255197	RP11-750H9.5		2.176202504	5.103853185	22.14091488	2.53E-06	6.52E-05	3.241034999	4.121805905	4.91854721	6.191406778	5.37395741
ENSG00000154928	EPHB1	EPH receptor B1 [Source:HGNC Symbol;Acc:3392]	3.672602641	0.996912807	22.12748048	2.55E-06	6.56E-05	-2.322346157	-2.726009782	-2.845623393	1.109604583	2.83459139
ENSG00000165891	E2F7	E2F transcription factor 7 [Source:HGNC Symbol;Acc:23820]	-2.489646703	2.088180261	22.0629742	2.64E-06	6.77E-05	2.512979527	3.115310691	-1.721358966	-0.589083246	2.487707366
ENSG00000152990	GPR125	G protein-coupled receptor 125 [Source:HGNC Symbol;Acc:13839]	-2.126570662	2.66552168	22.05742764	2.65E-06	6.78E-05	3.579246723	3.229953478	0.959559782	0.335717742	2.786891356
ENSG00000254326	IGHV7-27	immunoglobulin heavy variable 7-27 (pseudogene) [Source:HGNC Symbol;Acc:5663]	-8.652219871	0.163474557	22.04986687	2.66E-06	6.80E-05	1.832851647	0.583576386	-7.002070102	-7.002070102	-2.060109799
ENSG00000115590	IL1R2	interleukin 1 receptor, type II [Source:HGNC Symbol;Acc:5994]	3.202755092	2.517260659	22.02452882	2.69E-06	6.88E-05	0.848890306	0.664086661	1.644577544	3.985013877	2.578558393
ENSG00000015413	DPEP1	dipeptidase 1 (renal) [Source:HGNC Symbol;Acc:3002]	-8.05842773	-0.302520825	21.94606621	2.80E-06	7.16E-05	1.040754067	0.095631612	-7.002070102	-7.002070102	-0.649489103
ENSG00000177335	C8orf31	chromosome 8 open reading frame 31 [Source:HGNC Symbol;Acc:26731]	2.961351461	0.258998263	21.92078648	2.84E-06	7.23E-05	-1.034594152	-1.014020025	-0.390491059	1.27894994	0.615745481
ENSG00000272555	RP11-459I19.1		3.831245397	-0.158649023	21.92190021	2.84E-06	7.23E-05	-1.921450438	-2.076136033	-1.315629595	1.109604583	0.182236344
ENSG00000127507	EMR2	egf-like module containing, mucin-like, hormone receptor-like 2 [Source:HGNC Symbol;Acc:3337]	2.177653085	6.695187727	21.89455957	2.88E-06	7.32E-05	4.699264367	5.484112873	6.34330282	7.777445234	7.194727388
ENSG00000182492	BGN	biglycan [Source:HGNC Symbol;Acc:1044]	-8.277677254	-0.128195601	21.89124647	2.89E-06	7.32E-05	1.210071084	0.740340508	-7.002070102	-7.002070102	-1.87334355
ENSG00000242472	IGHJ5	immunoglobulin heavy joining 5 [Source:HGNC Symbol;Acc:5539]	-1.87450445	3.413751884	21.88253128	2.90E-06	7.34E-05	4.474571151	3.516835107	1.613958159	3.275467173	2.71589062
ENSG00000153721	CNKSR3	CNKSR family member 3 [Source:HGNC Symbol;Acc:23034]	-2.757393312	1.66652282	21.80555822	3.02E-06	7.63E-05	2.811663962	2.492318033	-3.233942493	-1.042179808	1.218946104
ENSG00000213160	KLHL23	kelch-like family member 23 [Source:HGNC Symbol;Acc:27506]	-2.268472097	2.209750512	21.79063088	3.04E-06	7.68E-05	3.181172573	2.92693583	0.081791624	-0.406691783	2.120832889
ENSG00000177989	ODF3B	outer dense fiber of sperm tails 3B [Source:HGNC Symbol;Acc:34388]	2.162374805	5.248786727	21.77739196	3.06E-06	7.72E-05	3.30320015	3.830594178	4.9843626	6.384253659	5.659636098
ENSG00000244682	FCGR2C	Fc fragment of IgG, low affinity IIc, receptor for (CD32) (gene/pseudogene) [Source:HGNC Symbol;Acc:15626]	1.784419231	5.062968694	21.760766	3.09E-06	7.78E-05	3.890900236	4.255574294	5.207131666	5.924743627	5.139065217
ENSG00000166664	CHRFAM7A	CHRNA7 (cholinergic receptor, nicotinic, alpha 7, exons 5-10) and FAM7A (family with sequence similarity 7A, exons A-E) fusion [Source:HGNC Symbol;Acc:15781]	3.490381901	-0.583861443	21.73345332	3.13E-06	7.88E-05	-2.322346157	-2.076136033	-3.233942493	0.466186565	0.182236344
ENSG00000119457	SLC46A2	solute carrier family 46, member 2 [Source:HGNC Symbol;Acc:16055]	2.611751637	4.119300123	21.7161737	3.16E-06	7.94E-05	1.89628059	2.729054134	3.547813796	5.341999652	4.585134766
ENSG00000046604	DSG2	desmoglein 2 [Source:HGNC Symbol;Acc:3049]	-2.454774064	2.130582511	21.64270201	3.28E-06	8.24E-05	3.18625708	2.932364153	-1.721358966	-0.053815412	1.84284836
ENSG00000259749	CTD-2022H16.2		-3.702902176	0.387142403	21.62350317	3.32E-06	8.31E-05	1.32517403	1.403429499	-3.766908349	-4.730697034	-0.045536284
ENSG00000164125	FAM198B	family with sequence similarity 198, member B [Source:HGNC Symbol;Acc:25312]	2.023095562	6.58857071	21.61502722	3.33E-06	8.33E-05	4.332747819	5.281037529	6.160725479	7.071849427	7.753978556
ENSG00000109674	NEIL3	nei endonuclease VIII-like 3 (E. coli) [Source:HGNC Symbol;Acc:24573]	-2.566378474	1.809426445	21.57270945	3.41E-06	8.48E-05	2.673662624	2.577932504	-1.886652327	-1.336401299	2.076176073
ENSG00000235534	FECHP1	ferrochelatase pseudogene 1 [Source:HGNC Symbol;Acc:3648]	-2.129334111	2.348737474	21.57470653	3.40E-06	8.48E-05	3.160653431	3.243117961	0.805492218	0.114810932	1.97033799
ENSG00000197451	HNRNPAB	heterogeneous nuclear ribonucleoprotein A/B [Source:HGNC Symbol;Acc:5034]	-1.556196089	6.327738668	21.57487925	3.40E-06	8.48E-05	7.001505717	6.8594097	5.313383893	5.64720597	6.070117803
ENSG00000171611	PTCRA	pre T-cell antigen receptor alpha [Source:HGNC Symbol;Acc:21290]	2.715699224	2.491811313	21.56656298	3.42E-06	8.50E-05	0.534915169	1.109650305	2.1289755	3.710101675	2.758908956
ENSG00000265625	RP11-68I3.11		4.033698773	1.090032806	21.55119867	3.45E-06	8.55E-05	-1.237502295	-0.782968247	-0.215316613	2.830854872	0.415212929

ENSG00000166928	MS4A14	membrane-spanning 4-domains, subfamily A, member 14 [Source:HGNC Symbol;Acc:30706]	2.650473215	3.965298816	21.52955757	3.48E-06	8.64E-05	1.883815937	2.905015959	3.686701825	5.295588046	3.853782901
ENSG00000143942	CHAC2	ChaC, cation transport regulator homolog 2 (E. coli) [Source:HGNC Symbol;Acc:32363]	-2.359917252	2.029894866	21.50148808	3.54E-06	8.75E-05	3.042264405	2.825562359	0.505333341	-0.956081001	1.515028015
ENSG00000165507	C10orf10	chromosome 10 open reading frame 10 [Source:HGNC Symbol;Acc:23355]	-2.673289708	1.506536675	21.4773925	3.58E-06	8.85E-05	2.837748047	2.126114598	-1.438615935	-1.449512048	0.735452007
ENSG00000100079	LGALS2	lectin, galactoside-binding, soluble, 2 [Source:HGNC Symbol;Acc:6562]	1.878742357	5.099249727	21.47221043	3.59E-06	8.86E-05	3.160653431	4.16855421	5.170777967	5.678666986	5.832371273
ENSG00000114268	PFKFB4	6-phosphofructo-2-kinase/fructose-2,6-biphosphatase 4 [Source:HGNC Symbol;Acc:8875]	1.864228074	5.077945394	21.43714258	3.66E-06	9.01E-05	3.621150429	4.114661149	4.969322447	5.937453053	5.535242871
ENSG00000086200	IPO11	importin 11 [Source:HGNC Symbol;Acc:20628]	-1.615331547	4.320633551	21.41080906	3.71E-06	9.11E-05	5.046840739	4.998786624	3.324906709	3.20246409	3.940421563
ENSG00000154856	APCDD1	adenomatosis polyposis coli down-regulated 1 [Source:HGNC Symbol;Acc:15718]	2.813860538	1.30553667	21.41193082	3.70E-06	9.11E-05	-0.698419327	-0.025429826	0.28980717	1.856429106	2.504656975
ENSG00000235036	RP5-1099D15.1		-1.775796363	3.732771064	21.403102	3.72E-06	9.14E-05	4.507443104	4.550279288	2.665349954	2.156150191	3.199787516
ENSG00000048462	TNFRSF17	tumor necrosis factor receptor superfamily, member 17 [Source:HGNC Symbol;Acc:11913]	-2.413559302	2.04477967	21.38919145	3.75E-06	9.19E-05	3.260449604	2.46261236	-0.010608983	-0.244792505	1.774613685
ENSG00000148053	NTRK2	neurotrophic tyrosine kinase, receptor, type 2 [Source:HGNC Symbol;Acc:8032]	-3.222321716	1.045411402	21.3576162	3.81E-06	9.33E-05	2.688072143	1.634124584	-2.287883461	-3.360122781	-2.526883755
ENSG00000108950	FAM20A	family with sequence similarity 20, member A [Source:HGNC Symbol;Acc:23015]	2.481536185	2.559998992	21.31971456	3.89E-06	9.50E-05	-0.14487434	0.376157587	0.778135316	2.106994346	4.398236712
ENSG00000176208	ATAD5	ATPase family, AAA domain containing 5 [Source:HGNC Symbol;Acc:25752]	-1.700635018	4.064615813	21.27068087	3.99E-06	9.72E-05	4.741326197	4.749469165	2.847469331	2.580193821	4.048396296
ENSG00000131355	EMR3	egf-like module containing, mucin-like, hormone receptor-like 3 [Source:HGNC Symbol;Acc:23647]	2.031143266	4.031703171	21.27327903	3.98E-06	9.72E-05	2.622064814	3.315529363	4.249129201	5.141787525	3.465217709
ENSG00000106462	EZH2	enhancer of zeste homolog 2 (Drosophila) [Source:HGNC Symbol;Acc:3527]	-1.569105273	4.793542142	21.26256499	4.00E-06	9.75E-05	5.454436398	5.339408959	3.664462604	3.572025663	4.881241457
ENSG00000029993	HMGB3	high mobility group box 3 [Source:HGNC Symbol;Acc:5004]	-2.084558712	3.449716037	21.22158975	4.09E-06	9.95E-05	4.318925344	4.207132934	1.184602189	2.231473747	3.157544141
ENSG00000101868	POLA1	polymerase (DNA directed), alpha 1, catalytic subunit [Source:HGNC Symbol;Acc:9173]	-1.603056934	4.703783711	21.19880118	4.14E-06	0.000100389	5.296478906	5.36170963	3.91735282	3.275467173	4.632168294
ENSG00000146070	PLA2G7	phospholipase A2, group VII (platelet-activating factor acetylhydrolase, plasma) [Source:HGNC Symbol;Acc:9040]	2.384057507	3.759783522	21.20080869	4.14E-06	0.000100389	1.883815937	2.66528446	3.531522919	4.743838665	4.252828525
ENSG00000094963	FMO2	flavin containing monooxygenase 2 (non-functional) [Source:HGNC Symbol;Acc:3770]	-4.693784099	0.656070407	21.18333614	4.17E-06	0.000100924	2.060341768	1.634124584	-4.621297774	-7.002070102	-2.060109799
ENSG00000124469	CEACAM8	carcinoembryonic antigen-related cell adhesion molecule 8 [Source:HGNC Symbol;Acc:1820]	-3.193631032	3.738387664	21.18399892	4.17E-06	0.000100924	4.470408989	2.424598913	-2.287883461	0.369457657	5.22977811
ENSG00000123374	CDK2	cyclin-dependent kinase 2 [Source:HGNC Symbol;Acc:1771]	-1.488872049	5.284843861	21.14267332	4.26E-06	0.000102946	5.865188795	5.843028919	4.415940919	4.41678084	5.179208255
ENSG00000243063	IGKV3-7	immunoglobulin kappa variable 3-7 (non-functional) [Source:HGNC Symbol;Acc:5821]	-3.319491573	0.648178593	21.10273368	4.35E-06	0.00010497	2.026722384	1.202433699	-2.845623393	-3.888428289	-0.045536284
ENSG00000173218	VANGL1	VANGL planar cell polarity protein 1 [Source:HGNC Symbol;Acc:15512]	-1.73705853	4.019366634	21.08840297	4.39E-06	0.000105613	4.744776617	4.834553798	2.635172888	2.848906753	3.585603588
ENSG00000119681	LTBP2	latent transforming growth factor beta binding protein 2 [Source:HGNC Symbol;Acc:6715]	-2.094341839	2.721151096	21.07616655	4.41E-06	0.000106144	3.820704546	3.502308971	1.397324829	0.497040107	1.658245749
ENSG00000229762	RP11-419M24.1		-2.615425195	1.371388047	21.07130065	4.43E-06	0.000106268	2.304339895	2.385556684	-2.073364206	-0.956081001	0.763888603
ENSG00000204577	LILRB3	leukocyte immunoglobulin-like receptor, subfamily B (with TM and ITIM domains), member 3 [Source:HGNC Symbol;Acc:6607]	2.256173997	4.725779423	21.06228599	4.45E-06	0.000106623	2.504862786	3.43888722	4.14321713	5.544797556	5.677496869
ENSG00000068078	FGFR3	fibroblast growth factor receptor 3 [Source:HGNC Symbol;Acc:3690]	-3.993906339	0.710261491	21.02295676	4.54E-06	0.000108555	1.562650064	1.933837788	-7.002070102	-3.888428289	0.002986071
ENSG00000224397	RP11-290F20.3		1.833872233	5.661260078	21.02265062	4.54E-06	0.000108555	4.190707534	4.924464118	5.804334409	6.697360094	5.474166643
ENSG00000240563	L1TD1	LINE-1 type transposase domain containing 1 [Source:HGNC Symbol;Acc:25595]	2.90893603	-0.038238859	20.93801998	4.74E-06	0.000113304	-1.921450438	-0.464918547	0.081791624	0.894716898	-0.57637152
ENSG00000177483	RBM44	RNA binding motif protein 44 [Source:HGNC Symbol;Acc:24756]	2.590469427	1.282174343	20.92762097	4.77E-06	0.000113766	0.133035942	0.207316148	1.205290011	2.096958951	1.612068369

ENSG00000173338	KCNK7	potassium channel, subfamily K, member 7 [Source:HGNC Symbol;Acc:6282]	3.35815462	1.317397854	20.90996323	4.81E-06	0.000114664	-0.489407866	-0.523165112	0.328039018	2.872629851	1.11240278
ENSG00000069482	GAL	galanin/GMAP prepropeptide [Source:HGNC Symbol;Acc:4114]	-8.614328423	0.20952839	20.90338586	4.83E-06	0.000114902	1.957037891	0.637745977	-7.002070102	-7.002070102	-4.610216385
ENSG00000251442	LINC01094	long intergenic non-protein coding RNA 1094 [Source:HGNC Symbol;Acc:49219]	3.033014931	1.254297654	20.89915223	4.84E-06	0.000115	-1.47369337	0.277241192	0.884581662	2.045707388	2.041752268
ENSG00000225217	HSPA7	heat shock 70kDa protein 7 (HSP70B) [Source:HGNC Symbol;Acc:5240]	1.834503913	4.478302145	20.86021353	4.94E-06	0.000117204	3.499792625	3.446494941	4.52898614	5.410452458	4.544733818
ENSG00000197146	AL133458.1		1.544275871	4.902521461	20.83901482	5.00E-06	0.000118188	4.069366531	4.339686916	5.407889352	5.725829945	4.074172926
ENSG00000264868	CTB-167B5.2		2.638374356	2.25290895	20.84017195	4.99E-06	0.000118188	-0.196880952	-0.025429826	1.142314722	2.444318394	3.82032647
ENSG00000148218	ALAD	aminolevulinate dehydratase [Source:HGNC Symbol;Acc:395]	-1.547232701	6.858948841	20.819348	5.05E-06	0.000119248	7.328239757	7.640949063	6.091568225	5.920482148	6.508059478
ENSG00000140284	SLC27A2	solute carrier family 27 (fatty acid transporter), member 2 [Source:HGNC Symbol;Acc:10996]	-2.187222778	2.636776956	20.79821789	5.10E-06	0.000120408	3.613621545	3.435068262	-0.161352853	0.894716898	2.31590077
ENSG00000187554	TLR5	toll-like receptor 5 [Source:HGNC Symbol;Acc:11851]	1.989322332	4.512802358	20.77319567	5.17E-06	0.000121828	2.5449974	3.450283811	4.263996407	5.108457793	5.440430209
ENSG00000160789	LMNA	lamin A/C [Source:HGNC Symbol;Acc:6636]	-1.605603615	5.778167561	20.7586075	5.21E-06	0.000122595	6.584693598	6.409068754	4.618470392	5.065361115	5.132264757
ENSG00000156206	C15orf26	chromosome 15 open reading frame 26 [Source:HGNC Symbol;Acc:26782]	2.838651547	0.495992423	20.73220729	5.28E-06	0.000124131	-1.756270337	-0.157588231	0.328039018	1.242998101	0.973207307
ENSG00000198157	HMGN5	high mobility group nucleosome binding domain 5 [Source:HGNC Symbol;Acc:8013]	-2.061342829	2.552132043	20.72749373	5.30E-06	0.00012427	3.575376245	3.29881546	0.778135316	0.642123966	1.994537666
ENSG00000131398	KCNC3	potassium voltage-gated channel, Shaw-related subfamily, member 3 [Source:HGNC Symbol;Acc:6235]	2.380351016	3.649085721	20.71150905	5.34E-06	0.000125145	1.89628059	2.598565174	3.4384784	4.823595141	3.754539027
ENSG00000233392	AC104809.4		1.95496231	4.36865702	20.69817285	5.38E-06	0.000125851	2.622064814	3.909778597	5.093815094	5.240813742	3.300281982
ENSG00000148346	LCN2	lipocalin 2 [Source:HGNC Symbol;Acc:6526]	-3.179043665	5.273477477	20.67772063	5.43E-06	0.000127033	6.137408525	3.69346042	1.342349735	2.615771827	6.661415443
ENSG00000006042	TMEM98	transmembrane protein 98 [Source:HGNC Symbol;Acc:24529]	-2.777154867	1.282814421	20.65801904	5.49E-06	0.000128177	2.394966414	2.439924693	-3.766908349	-1.449512048	-0.506781561
ENSG00000128578	STRIP2	striatin interacting protein 2 [Source:HGNC Symbol;Acc:22209]	-2.126769457	2.299165242	20.64735536	5.52E-06	0.000128721	2.979215452	3.43888722	0.036330944	0.335717742	1.702990823
ENSG00000101162	TUBB1	tubulin, beta 1 class VI [Source:HGNC Symbol;Acc:16257]	2.447850692	7.576415893	20.64086313	5.54E-06	0.000128987	5.226632795	6.238247477	7.324739942	8.808404155	7.860890036
ENSG00000144647	POMGNT2	protein O-linked mannose N-acetylglucosaminyltransferase 2 (beta 1,4-) [Source:HGNC Symbol;Acc:25902]	-1.737439271	3.720641836	20.63227181	5.57E-06	0.000129396	4.474571151	4.612468678	2.6198443	2.231473747	3.011029232
ENSG00000233901	RP11-65J3.1		2.87526322	2.101585518	20.62329624	5.59E-06	0.000129832	0.000762307	0.98980953	1.450281721	3.178871708	2.679035416
ENSG00000237476	XXbac-B135H6.15		2.589035702	0.765110884	20.60078192	5.66E-06	0.000131194	-0.625332215	-0.252938016	0.632982468	1.582074621	1.134350211
ENSG00000213557	RP11-240E2.2		3.05019282	0.926119421	20.58695589	5.70E-06	0.000131971	-1.034594152	-0.025429826	0.934997507	2.294302704	-0.147769603
ENSG00000185128	TBC1D3F	TBC1 domain family, member 3F [Source:HGNC Symbol;Acc:18257]	2.662801701	0.061811748	20.56471007	5.76E-06	0.000133337	-1.237502295	-0.782968247	-0.271377577	0.722650538	0.551948963
ENSG00000213551	DNAJC9	DnaJ (Hsp40) homolog, subfamily C, member 9 [Source:HGNC Symbol;Acc:19123]	-1.550455463	5.14265895	20.54139944	5.84E-06	0.000134793	5.852451395	5.768355894	4.386805333	3.765005219	4.884482612
ENSG00000176619	LMNB2	lamin B2 [Source:HGNC Symbol;Acc:6638]	-1.44867446	5.917569085	20.51613346	5.91E-06	0.000136405	6.43574878	6.523636984	5.145577126	4.73415894	5.939092537
ENSG00000130560	UBAC1	UBA domain containing 1 [Source:HGNC Symbol;Acc:30221]	-1.49277265	6.320906032	20.47177313	6.05E-06	0.00013942	6.892182271	7.070139413	5.40900449	5.592899069	5.794106566
ENSG00000205213	LGR4	leucine-rich repeat containing G protein-coupled receptor 4 [Source:HGNC Symbol;Acc:13299]	-2.014824232	2.690129009	20.45015724	6.12E-06	0.000140819	3.420029142	3.69346042	0.6928072	1.0274492	2.216526545
ENSG00000123131	PRDX4	peroxiredoxin 4 [Source:HGNC Symbol;Acc:17169]	-1.716366774	4.730386072	20.44195045	6.15E-06	0.000141239	5.73852217	5.086609179	3.622778389	3.403124214	4.471189828
ENSG00000147394	ZNF185	zinc finger protein 185 (LIM domain) [Source:HGNC Symbol;Acc:12976]	2.172345305	5.966811048	20.43919462	6.16E-06	0.000141257	4.190707534	4.808223102	5.511195355	7.199266765	6.201316827
ENSG00000163918	RFC4	replication factor C (activator 1) 4, 37kDa [Source:HGNC Symbol;Acc:9972]	-1.616077268	4.449264623	20.4270218	6.19E-06	0.000141973	5.151933519	5.123856905	3.615068323	2.806428868	4.267798636
ENSG00000229754	CXCR2P1	chemokine (C-X-C motif) receptor 2 pseudogene 1 [Source:HGNC Symbol;Acc:6028]	1.791847591	4.868496078	20.42450865	6.20E-06	0.000141974	3.326414242	4.088156756	5.060139753	5.724200942	5.023275359
ENSG00000126861	OMG	oligodendrocyte myelin glycoprotein [Source:HGNC Symbol;Acc:8135]	2.825901971	0.804206204	20.42034785	6.22E-06	0.000142098	-0.14487434	-1.289265519	0.570569237	1.414416401	1.596341512
ENSG00000143382	ADAMTSL4	ADAMTS-like 4 [Source:HGNC Symbol;Acc:19706]	1.928793001	6.05622184	20.41566249	6.23E-06	0.000142261	4.085784489	4.898351828	5.739326907	6.806845016	6.910269821
ENSG00000104267	CA2	carbonic anhydrase II [Source:HGNC Symbol;Acc:1373]	-2.38257491	6.467048113	20.35642378	6.43E-06	0.000146543	7.175861076	7.545216297	3.690375259	5.497899733	5.714390898
ENSG00000102174	PHEX	phosphate regulating endopeptidase homolog, X-linked [Source:HGNC Symbol;Acc:8918]	2.973880234	0.131847739	20.33907203	6.49E-06	0.000147686	-2.322346157	-1.394066322	-1.315629595	0.154058352	1.658245749
ENSG00000258732	RP11-603B24.1		2.595264703	-0.04075893	20.33421848	6.50E-06	0.000147869	-1.034594152	-1.014020025	0.401591237	0.798919003	-0.894110176

ENSG00000130700	GATA5	GATA binding protein 5 [Source:HGNC Symbol;Acc:15802]	-8.325402345	-0.155477427	20.28611116	6.67E-06	0.000151438	1.431768352	0.555709266	-7.002070102	-7.002070102	-7.002070102
ENSG00000254056	IGHV3-71	immunoglobulin heavy variable 3-71 (pseudogene) [Source:HGNC Symbol;Acc:5621]	-3.704145793	0.75782681	20.28250867	6.68E-06	0.000151528	2.187493018	0.968831255	-2.845623393	-7.002070102	0.615745481
ENSG00000101412	E2F1	E2F transcription factor 1 [Source:HGNC Symbol;Acc:3113]	-1.740643773	3.956600257	20.27682587	6.70E-06	0.000151703	4.337326037	4.585159354	2.253389193	2.580193821	4.484066609
ENSG00000256448	RP11-809N8.4		2.422241753	1.313847596	20.27536427	6.71E-06	0.000151703	-0.094677427	0.171040209	1.517996407	2.203686226	1.3928929
ENSG00000175262	C1orf127	chromosome 1 open reading frame 127 [Source:HGNC Symbol;Acc:26730]	2.582933165	0.249605311	20.24883544	6.80E-06	0.000153623	-1.237502295	-0.157588231	0.28980717	0.940331404	0.264147325
ENSG00000147883	CDKN2B	cyclin-dependent kinase inhibitor 2B (p15, inhibits CDK4) [Source:HGNC Symbol;Acc:1788]	2.785102993	1.505216418	20.24597454	6.81E-06	0.000153655	-0.365194284	-0.112176905	0.9100098	2.354509285	2.390748373
ENSG00000197721	CR1L	complement component (3b/4b) receptor 1-like [Source:HGNC Symbol;Acc:2335]	-2.445401227	1.6168938	20.22096096	6.90E-06	0.000155477	2.42108026	2.671789775	-1.721358966	-1.231517591	1.35601001
ENSG00000198208	RPS6KL1	ribosomal protein S6 kinase-like 1 [Source:HGNC Symbol;Acc:20222]	-2.094138371	2.674492681	20.21697502	6.91E-06	0.000155602	3.609842318	3.690259922	1.517996407	0.03296005	1.374569317
ENSG00000218809	RP1-229K20.5		4.51205772	1.336422255	20.20086345	6.97E-06	0.000156717	-1.034594152	-2.262017103	-1.438615935	2.812574249	2.087470534
ENSG00000141574	SECTM1	secreted and transmembrane 1 [Source:HGNC Symbol;Acc:10707]	2.02330199	6.316225493	20.16509526	7.10E-06	0.000159471	4.474571151	5.270360911	6.125883162	7.379536813	6.657631615
ENSG00000118515	SGK1	serum/glucocorticoid regulated kinase 1 [Source:HGNC Symbol;Acc:10810]	1.959422658	6.985002021	20.1462013	7.17E-06	0.000160848	5.640051903	5.499736832	6.777716269	8.09531238	7.274099455
ENSG00000213694	S1PR3	sphingosine-1-phosphate receptor 3 [Source:HGNC Symbol;Acc:3167]	2.355294408	4.430929457	20.13615547	7.21E-06	0.000161489	2.636996308	3.185183524	3.926726325	5.682866335	4.681448344
ENSG00000248429	RP11-597D13.9		3.1758246	3.092598168	20.06129122	7.50E-06	0.000167722	0.848890306	1.494316873	1.992811462	4.521784194	3.430160334
ENSG00000185499	MUC1	mucin 1, cell surface associated [Source:HGNC Symbol;Acc:7508]	-1.991125914	2.534055616	20.01246736	7.69E-06	0.00017184	3.598444871	3.251828018	1.053829154	0.962608982	1.580441325
ENSG00000151892	GFRA1	GDNF family receptor alpha 1 [Source:HGNC Symbol;Acc:4243]	-7.967291865	-0.337021073	20.00230313	7.73E-06	0.000172425	0.742455703	0.904001603	-7.002070102	-7.002070102	-3.754655114
ENSG00000164023	SGMS2	sphingomyelin synthase 2 [Source:HGNC Symbol;Acc:28395]	1.967493994	3.937180513	20.00111155	7.74E-06	0.000172425	2.038016118	3.105733301	4.068967903	4.462441992	4.632168294
ENSG00000103196	CRISPLD2	cysteine-rich secretory protein LCCL domain containing 2 [Source:HGNC Symbol;Acc:25248]	1.729918206	4.962175166	19.99481942	7.77E-06	0.000172774	3.211414251	4.198147959	5.097969776	5.604456613	5.49446052
ENSG00000088827	SIGLEC1	sialic acid binding Ig-like lectin 1, sialoadhesin [Source:HGNC Symbol;Acc:11127]	2.94175205	3.926823639	19.95843061	7.91E-06	0.00017587	1.845762494	2.038220677	2.709464044	5.299968942	4.4668719
ENSG00000273174	RP11-434H6.6		2.859602723	1.611515072	19.95016935	7.95E-06	0.000176409	0.254194054	0.133828548	1.265630948	2.918936894	1.446507348
ENSG00000215458	AP001053.11		2.06352041	4.288994527	19.94589964	7.97E-06	0.00017658	2.394966414	3.491317573	4.370869767	5.340937098	4.302136394
ENSG00000197299	BLM	Bloom syndrome, RecQ helicase-like [Source:HGNC Symbol;Acc:1058]	-1.618895449	4.201296242	19.93861954	8.00E-06	0.00017703	4.782196375	4.825830186	3.206763523	2.622783341	4.352157627
ENSG00000095002	MSH2	mutS homolog 2 [Source:HGNC Symbol;Acc:7325]	-1.76658684	5.769093578	19.89826091	8.17E-06	0.000180579	6.397898246	6.50599754	5.264935772	3.998553766	5.417119883
ENSG00000101935	AMMECR1	Alport syndrome, mental retardation, midface hypoplasia and elliptocytosis chromosomal region gene 1 [Source:HGNC Symbol;Acc:467]	-1.527353955	5.177385752	19.8956459	8.18E-06	0.000180599	5.779470742	5.983655362	4.612674392	3.985013877	4.4668719
ENSG00000259529	RP11-468E2.4	E3 ubiquitin-protein ligase RNF31 [Source:UniProtKB/TrEMBL;Acc:H0YM83]	2.810940713	1.182595122	19.85745705	8.34E-06	0.000184011	-0.365194284	0.207316148	0.6928072	2.354509285	1.259453775
ENSG00000188158	NHS	Nance-Horan syndrome (congenital cataracts and dental anomalies) [Source:HGNC Symbol;Acc:7820]	2.799677983	2.37557879	19.79731204	8.61E-06	0.000189655	0.292419846	0.881727766	1.689321579	3.745865181	2.5705316
ENSG00000100749	VRK1	vaccinia related kinase 1 [Source:HGNC Symbol;Acc:12718]	-1.590945193	4.903521522	19.786998	8.66E-06	0.000190234	5.619524849	5.51157143	4.193266557	3.344953407	4.707302615
ENSG00000144381	HSPD1	heat shock 60kDa protein 1 (chaperonin) [Source:HGNC Symbol;Acc:5261]	-1.471399913	6.31921762	19.78430729	8.67E-06	0.000190234	6.999701781	6.903606579	5.637397835	5.244227703	5.990383954
ENSG00000101188	NTSR1	neurotensin receptor 1 (high affinity) [Source:HGNC Symbol;Acc:8039]	2.78006547	1.036715727	19.78485454	8.67E-06	0.000190234	-1.608074432	-0.782968247	-1.438615935	0.74852395	2.8278729
ENSG00000206538	VGLL3	vestigial like 3 (Drosophila) [Source:HGNC Symbol;Acc:24327]	-8.372528688	-0.043234618	19.70570031	9.03E-06	0.000197974	1.249467958	1.09035546	-7.002070102	-7.002070102	-7.002070102
ENSG00000186638	KIF24	kinesin family member 24 [Source:HGNC Symbol;Acc:19916]	-2.17925183	2.118689949	19.69936909	9.06E-06	0.000198383	2.979215452	2.865836059	-0.453950945	-0.099242291	2.19580308
ENSG00000119280	C1orf198	chromosome 1 open reading frame 198 [Source:HGNC Symbol;Acc:25900]	-1.546886962	4.29157192	19.66916961	9.21E-06	0.000201042	5.011469904	5.002662158	3.281728793	3.140302572	3.883244358
ENSG00000159714	ZDHHC1	zinc finger, DHHC-type containing 1 [Source:HGNC Symbol;Acc:17916]	2.558837866	1.290135465	19.67129604	9.20E-06	0.000201042	0.174562846	0.376157587	1.030832979	2.267710332	1.410986673
ENSG00000085063	CD59	CD59 molecule, complement regulatory protein [Source:HGNC Symbol;Acc:1689]	-1.729906581	5.55626239	19.64754784	9.31E-06	0.000203077	6.329459238	6.169809228	4.976862123	3.67004846	5.261286535

ENSG00000135519	KCNH3	potassium voltage-gated channel, subfamily H (eag-related), member 3 [Source:HGNC Symbol;Acc:6252]	1.988711157	3.456102263	19.63865481	9.36E-06	0.000203519	1.752861822	2.612158157	3.571910301	4.092461225	4.016252961
ENSG00000236345	RP11-59D5__B.2		2.491536275	0.290832734	19.63913837	9.35E-06	0.000203519	-1.034594152	-0.408932621	0.505333341	0.58582888	0.763888603
ENSG00000213430	HSPD1P1	heat shock 60kDa protein 1 (chaperonin) pseudogene 1 [Source:HGNC Symbol;Acc:35133]	-1.503294469	6.523281354	19.60125658	9.54E-06	0.000207285	7.243886426	7.123911359	5.821186175	5.253292298	6.20715315
ENSG00000253250	C8orf88	chromosome 8 open reading frame 88 [Source:HGNC Symbol;Acc:44672]	-2.383970219	1.621356286	19.59302416	9.58E-06	0.000207922	2.591730062	2.377619831	-0.99931534	-0.655391447	1.446507348
ENSG00000183722	LHFP	lipoma HMGIC fusion partner [Source:HGNC Symbol;Acc:6586]	-2.334350585	1.62859108	19.56651306	9.72E-06	0.000210567	2.606977165	2.470096317	-1.438615935	-0.46496394	1.279288931
ENSG00000183508	FAM46C	family with sequence similarity 46, member C [Source:HGNC Symbol;Acc:24712]	-1.825598766	6.368085904	19.5600213	9.75E-06	0.000211024	7.355860198	6.890767956	5.855128878	4.57878161	5.598779761
ENSG00000127423	AUNIP	aurora kinase A and ninein interacting protein [Source:HGNC Symbol;Acc:28363]	-2.415417425	1.596279877	19.5445636	9.83E-06	0.000212232	2.403723654	2.48494876	-1.438615935	-1.572251189	1.688229459
ENSG00000138182	KIF20B	kinesin family member 20B [Source:HGNC Symbol;Acc:7212]	-1.540621085	4.821440681	19.54441133	9.83E-06	0.000212232	5.341612239	5.562345304	4.145895048	3.234858397	4.709131739
ENSG00000138028	CGREF1	cell growth regulator with EF-hand domain 1 [Source:HGNC Symbol;Acc:16962]	-2.81277409	1.218646975	19.50564918	1.00E-05	0.000216316	2.496700121	2.077947121	-2.539967122	-2.670141381	0.182236344
ENSG00000102098	SCML2	sex comb on midleg-like 2 (Drosophila) [Source:HGNC Symbol;Acc:10581]	-1.870610552	2.80462609	19.48092129	1.02E-05	0.000218866	3.503870991	3.583904609	1.285196495	0.871357159	2.814341322
ENSG00000270472	IGHV3OR16-9	immunoglobulin heavy variable 3/OR16-9 (non-functional) [Source:HGNC Symbol;Acc:5644]	-4.711288012	0.222554211	19.39556201	1.06E-05	0.000228589	1.806678074	0.527293245	-4.621297774	-7.002070102	-0.80787826
ENSG00000166866	MYO1A	myosin IA [Source:HGNC Symbol;Acc:7595]	2.364601713	0.088315897	19.34477296	1.09E-05	0.000234462	-0.775407551	-0.408932621	0.721814282	0.369457657	-0.376928291
ENSG00000143452	HORMAD1	HORMA domain containing 1 [Source:HGNC Symbol;Acc:25245]	2.772491641	0.226106299	19.29853185	1.12E-05	0.000239916	-1.034594152	-0.583862591	-0.329705368	1.168289627	0.450630967
ENSG00000165392	WRN	Werner syndrome, RecQ helicase-like [Source:HGNC Symbol;Acc:12791]	-1.497239166	4.703469339	19.26724382	1.14E-05	0.000243582	5.272774078	5.464576116	4.043343998	3.37017149	4.345117047
ENSG00000137959	IFI44L	interferon-induced protein 44-like [Source:HGNC Symbol;Acc:17817]	2.170916401	4.554196322	19.25082619	1.15E-05	0.000245386	2.857006212	3.256163405	4.054788623	5.392103294	5.385441744
ENSG00000223552	RP11-24F11.2		2.459700806	0.598291954	19.22164267	1.16E-05	0.000248863	-1.132483902	-0.408932621	-0.010608983	0.402426505	1.933259916
ENSG00000170891	CYTL1	cytokine-like 1 [Source:HGNC Symbol;Acc:24435]	-2.639873086	2.799616957	19.2149766	1.17E-05	0.000249429	3.887782536	3.494990685	-1.57307192	1.314027603	2.487707366
ENSG00000233822	HIST1H2BN	histone cluster 1, H2bn [Source:HGNC Symbol;Acc:4749]	2.640485003	-0.089992043	19.20725221	1.17E-05	0.000250136	-0.775407551	-1.394066322	-0.215316613	0.527247606	0.223773082
ENSG00000166927	MS4A7	membrane-spanning 4-domains, subfamily A, member 7 [Source:HGNC Symbol;Acc:13378]	2.050979035	6.208719111	19.19267201	1.18E-05	0.000251748	4.453639214	5.351615674	6.056417168	7.332733458	6.304130865
ENSG00000173535	TNFRSF10C	tumor necrosis factor receptor superfamily, member 10c, decoy without an intracellular domain [Source:HGNC Symbol;Acc:11906]	1.970046681	4.233123628	19.15452448	1.21E-05	0.000256477	2.831271105	3.323814258	4.105194787	5.213207262	4.479787106
ENSG00000172243	CLEC7A	C-type lectin domain family 7, member A [Source:HGNC Symbol;Acc:14558]	2.328346774	7.19255208	19.15251988	1.21E-05	0.000256477	5.162291765	6.045075729	6.876957304	8.462085472	7.313408764
ENSG00000214548	MEG3	maternally expressed 3 (non-protein coding) [Source:HGNC Symbol;Acc:14575]	3.554328653	1.95183827	19.14281564	1.21E-05	0.000257473	0.329658892	0.095631612	-0.059127673	3.848125445	0.378903451
ENSG00000180535	BHLHA15	basic helix-loop-helix family, member a15 [Source:HGNC Symbol;Acc:22265]	-3.807824195	0.326103744	19.12746978	1.22E-05	0.000259238	1.79341104	0.715367718	-3.233942493	-7.002070102	-0.376928291
ENSG00000272632	RP11-362F19.3		3.205440155	0.549380773	19.12208183	1.23E-05	0.000259657	-1.608074432	-0.583862591	0.250534523	1.79568083	0.450630967
ENSG00000196873	CBWD3	COBW domain containing 3 [Source:HGNC Symbol;Acc:18519]	2.507748906	0.103169808	19.11640294	1.23E-05	0.000260117	-0.425964666	-0.523165112	0.210162819	0.984547783	-0.9858258
ENSG00000161944	ASGR2	asialoglycoprotein receptor 2 [Source:HGNC Symbol;Acc:743]	1.838985901	4.984638236	19.09695814	1.24E-05	0.000262465	3.449931193	4.359970556	5.153582945	5.972180623	4.816538401
ENSG00000187715	KBTBD12	kelch repeat and BTB (POZ) domain containing 12 [Source:HGNC Symbol;Acc:25731]	-2.716530264	1.221625959	19.08461644	1.25E-05	0.00026385	2.350364654	2.287305009	-3.233942493	-2.0191864	0.002986071
ENSG00000136732	GYPC	glycophorin C (Gerbich blood group) [Source:HGNC Symbol;Acc:4704]	-1.855893312	6.678169304	18.99853416	1.31E-05	0.000275693	7.371010443	7.456164799	6.162710168	4.693112741	6.191536847
ENSG00000186407	CD300E	CD300e molecule [Source:HGNC Symbol;Acc:28874]	2.536461786	7.692766177	18.98546308	1.32E-05	0.000277256	5.034206909	6.111783523	6.950025987	8.875250741	8.405371625
ENSG00000223511	RP13-297E16.4		2.441097458	0.114665937	18.94471186	1.35E-05	0.000282902	-0.489407866	-0.464918547	0.125863448	0.847612951	-0.316136847
ENSG00000113303	BTNL8	butyrophilin-like 8 [Source:HGNC Symbol;Acc:26131]	3.368886857	0.835653741	18.92975535	1.36E-05	0.000284787	-1.132483902	-2.076136033	-3.233942493	1.493047208	2.3444256
ENSG00000018280	SLC11A1	solute carrier family 11 (proton-coupled divalent metal ion transporter), member 1 [Source:HGNC Symbol;Acc:10907]	2.456634936	8.032631787	18.89346463	1.38E-05	0.00028991	5.900421316	6.615501972	7.573759962	9.357259047	8.251804454
ENSG00000106991	ENG	endoglin [Source:HGNC Symbol;Acc:3349]	-1.591544437	6.142002829	18.85164239	1.41E-05	0.000295983	6.806862961	6.78034359	4.821385224	5.507402052	5.83656093

ENSG00000087842	PIR	pirin (iron-binding nuclear protein) [Source:HGNC Symbol;Acc:30048]	-2.600714238	1.299099508	18.81704932	1.44E-05	0.000301041	2.504862786	2.253009061	-2.845623393	-1.572251189	0.095393329
ENSG00000203804	ADAMTSL4-AS1	ADAMTSL4 antisense RNA 1 [Source:HGNC Symbol;Acc:32041]	3.87618509	-0.623996686	18.75147	1.49E-05	0.000311201	-2.108017771	-3.940353384	-3.233942493	0.61425099	0.04992943
ENSG00000111644	ACRBP	acrosin binding protein [Source:HGNC Symbol;Acc:17195]	1.725283545	4.785702225	18.73749445	1.50E-05	0.000312976	3.483362881	3.958479768	4.782333156	5.692895183	4.984532378
ENSG00000152207	CYSLTR2	cysteinyl leukotriene receptor 2 [Source:HGNC Symbol;Acc:18274]	2.048266289	2.45912255	18.73609205	1.50E-05	0.000312976	1.16956809	1.711936244	2.840873769	3.32789308	2.164148811
ENSG00000270120	RP11-327F22.6		3.009301752	0.521450651	18.7150627	1.52E-05	0.000316071	-1.237502295	-0.647226338	0.081791624	1.679439471	0.646616095
ENSG00000175793	SFN	stratifin [Source:HGNC Symbol;Acc:10773]	3.314673323	-0.022842732	18.70582663	1.53E-05	0.000317229	-1.237502295	-2.262017103	-1.202308962	1.206127397	0.341656497
ENSG00000118785	SPP1	secreted phosphoprotein 1 [Source:HGNC Symbol;Acc:11255]	-3.06949206	0.766183059	18.69775966	1.53E-05	0.000318197	1.498692903	2.244306154	-1.886652327	-7.002070102	-1.70800759
ENSG00000261056	RP11-454F8.2		-2.514653571	0.813313867	18.68067714	1.55E-05	0.000320681	1.89628059	1.607225949	-1.721358966	-2.0191864	0.223773082
ENSG00000261997	RP11-212I21.4		2.920152584	0.639549426	18.67360358	1.55E-05	0.000321493	-1.034594152	-0.932821174	0.28980717	1.770646721	0.898226269
ENSG00000152455	SUV39H2	suppressor of variegation 3-9 homolog 2 (Drosophila) [Source:HGNC Symbol;Acc:17287]	-1.957557666	2.520917175	18.6264507	1.59E-05	0.000329154	3.358298947	3.269091943	1.120696904	0.03296005	2.408868893
ENSG00000184678	HIST2H2BE	histone cluster 2, H2be [Source:HGNC Symbol;Acc:4760]	1.88856517	4.680802778	18.6213343	1.59E-05	0.00032965	3.536089402	3.247479563	4.283583984	5.575838958	5.311515536
ENSG00000119686	FLVCR2	feline leukemia virus subgroup C cellular receptor family, member 2 [Source:HGNC Symbol;Acc:20105]	1.967188884	3.983820968	18.58675848	1.62E-05	0.000335289	2.071376358	2.964512035	3.730177502	4.643988414	4.836757869
ENSG00000136492	BRIP1	BRCA1 interacting protein C-terminal helicase 1 [Source:HGNC Symbol;Acc:20473]	-1.916301795	2.844932716	18.5838027	1.63E-05	0.000335414	3.385078807	3.563059673	1.582674799	0.402426505	3.178820438
ENSG00000226971	AF196972.4		-1.515557505	4.434633104	18.57956516	1.63E-05	0.000335766	5.170011935	5.093893865	3.46445507	3.48235623	3.949734605
ENSG00000124313	IQSEC2	IQ motif and Sec7 domain 2 [Source:HGNC Symbol;Acc:29059]	1.873535447	4.23005775	18.57108483	1.64E-05	0.000336868	2.869703637	3.229953478	4.083009179	5.186245939	4.554940616
ENSG00000135919	SERPINE2	serpin peptidase inhibitor, clade E (nexin, plasminogen activator inhibitor type 1), member 2 [Source:HGNC Symbol;Acc:8951]	-2.050529109	3.109883825	18.55684487	1.65E-05	0.000338996	4.118070306	3.82768418	2.118106677	0.369457657	2.504656975
ENSG00000010030	ETV7	ets variant 7 [Source:HGNC Symbol;Acc:18160]	2.368630261	0.901327705	18.54541804	1.66E-05	0.000340636	-0.196880952	0.468724906	1.098750223	1.75796486	0.341656497
ENSG00000114698	PLSCR4	phospholipid scramblase 4 [Source:HGNC Symbol;Acc:16497]	-2.74951417	1.166709116	18.54028346	1.66E-05	0.000341156	2.103982592	2.135558076	-3.233942493	-2.419096061	0.948644093
ENSG00000125885	MCM8	minichromosome maintenance complex component 8 [Source:HGNC Symbol;Acc:16147]	-1.488264601	4.633792255	18.532264	1.67E-05	0.000342093	5.277546306	5.233457778	3.840088097	3.095642933	4.618607854
ENSG00000260929	RP11-327F22.1		2.588116659	0.351202202	18.53060323	1.67E-05	0.000342093	-1.034594152	-0.464918547	0.365283811	1.27894994	0.223773082
ENSG00000154096	THY1	Thy-1 cell surface antigen [Source:HGNC Symbol;Acc:11801]	-8.134494681	-0.223546725	18.51569925	1.69E-05	0.000344378	1.040754067	0.812765477	-7.002070102	-7.002070102	-2.832686105
ENSG00000241489	IDS	Iduronate 2-sulfatase (Hunter syndrome), isoform CRA_e; Iduronate 2-sulfatase 14 kDa chain; cDNA FLJ42669 fis, clone BRAMY2022168, highly similar to IDURONATE 2-SULFATASE [Source:UniProtKB/TrEMBL;Acc:B3KWA1]	2.542500487	0.08614527	18.48310146	1.71E-05	0.000349911	-1.756270337	-0.355038393	0.250534523	0.03296005	0.819133578
ENSG00000109881	CCDC34	coiled-coil domain containing 34 [Source:HGNC Symbol;Acc:25079]	-1.865150955	2.72072927	18.43222645	1.76E-05	0.000358934	3.583106845	3.480241792	1.415193986	0.527247606	2.435629945
ENSG00000225062	AC021016.6		2.964966766	0.192098931	18.43015407	1.76E-05	0.000358934	-1.608074432	-1.191564836	-1.097244829	0.871357159	1.259453775
ENSG00000204634	TBC1D8	TBC1 domain family, member 8 (with GRAM domain) [Source:HGNC Symbol;Acc:17791]	1.856392672	5.143375009	18.40310735	1.79E-05	0.000363643	3.613621545	4.182288334	4.926356237	6.241234525	5.321115655
ENSG00000230724	LINC01001	long intergenic non-protein coding RNA 1001 [Source:HGNC Symbol;Acc:38540]	2.388850915	2.230793588	18.39804721	1.79E-05	0.000364188	0.94800933	0.689955027	1.842443831	3.145180481	2.854561153
ENSG00000222009	BTBD19	BTB (POZ) domain containing 19 [Source:HGNC Symbol;Acc:27145]	2.652846379	1.720523814	18.39022563	1.80E-05	0.000365263	0.292419846	0.277241192	1.120696904	2.918936894	2.006486965
ENSG00000061656	SPAG4	sperm associated antigen 4 [Source:HGNC Symbol;Acc:11214]	-2.516993628	1.452398239	18.38077448	1.81E-05	0.000366656	2.702339162	1.933837788	-0.740035668	-2.205328685	1.177268129
ENSG00000104889	RNASEH2A	ribonuclease H2, subunit A [Source:HGNC Symbol;Acc:18518]	-1.58809023	3.940376241	18.36314756	1.83E-05	0.000369637	4.646747126	4.571308358	2.860570679	2.836897298	3.725915699
ENSG00000214881	TMEM14D	transmembrane protein 14D (pseudogene) [Source:HGNC Symbol;Acc:15660]	-1.389370692	5.535914984	18.35285079	1.84E-05	0.000371212	6.367445835	6.13315478	4.717204047	4.414762411	4.967822647
ENSG00000082014	SMARCD3	SWI/SNF related, matrix associated, actin dependent regulator of chromatin, subfamily d, member 3 [Source:HGNC Symbol;Acc:11108]	1.874245365	5.293351843	18.31532307	1.87E-05	0.000378161	3.658216533	4.512724313	5.106243416	6.25314475	5.632905242

ENSG00000113140	SPARC	secreted protein, acidic, cysteine-rich (osteonectin) [Source:HGNC Symbol;Acc:11219]	1.701217316	6.691548237	18.30625546	1.88E-05	0.000379528	5.042641743	5.73845476	6.51625548	7.395233088	7.412157921
ENSG00000215440	NPEPL1	aminopeptidase-like 1 [Source:HGNC Symbol;Acc:16244]	1.501714858	4.434171062	18.28330468	1.90E-05	0.000383687	3.544032825	3.950476219	5.019831858	4.96548179	4.088296742
ENSG00000255163	HSPE1P18	heat shock 10kDa protein 1 pseudogene 18 [Source:HGNC Symbol;Acc:49337]	2.887304804	1.105094052	18.27642607	1.91E-05	0.000384634	-1.47369337	0.498306264	1.956663165	1.992567844	-0.257803757
ENSG00000205846	CLEC6A	C-type lectin domain family 6, member A [Source:HGNC Symbol;Acc:14556]	2.694910376	0.9958681	18.26853294	1.92E-05	0.000385788	-0.856736938	0.277241192	0.721814282	1.856429106	1.410986673
ENSG00000261222	CTD-2006K23.1		2.368292452	2.847341596	18.26468054	1.92E-05	0.000386127	0.686141241	1.844528154	2.71668701	3.900992258	3.168221511
ENSG00000173894	CBX2	chromobox homolog 2 [Source:HGNC Symbol;Acc:1552]	-2.20388562	2.872997379	18.26170975	1.93E-05	0.000386288	3.690780833	3.607849048	-0.66302287	1.43048915	2.981063822
ENSG00000142178	SIK1	salt-inducible kinase 1 [Source:HGNC Symbol;Acc:11142]	-1.563079252	6.321159335	18.23859097	1.95E-05	0.000390558	7.297145635	6.681863005	5.483939025	5.585740437	5.547562832
ENSG00000226259	GTF2H2B	general transcription factor IIH, polypeptide 2B (pseudogene) [Source:HGNC Symbol;Acc:31393]	2.481694015	0.375398695	18.22730042	1.96E-05	0.000392432	-0.365194284	-0.157588231	0.250534523	1.261086007	-0.095747566
ENSG00000144354	CDCA7	cell division cycle associated 7 [Source:HGNC Symbol;Acc:14628]	-1.574887607	3.996264561	18.1997738	1.99E-05	0.000397692	4.34870842	4.359970556	2.680205034	2.213008328	4.785667729
ENSG00000167995	BEST1	bestrophin 1 [Source:HGNC Symbol;Acc:12703]	2.305345394	3.85359224	18.18526831	2.00E-05	0.000400276	2.093195522	2.455089377	3.154531226	4.864099294	4.599011985
ENSG00000182704	TSKU	tsukushi, small leucine rich proteoglycan [Source:HGNC Symbol;Acc:28850]	-3.874486584	0.369746701	18.18091745	2.01E-05	0.000400736	1.752861822	1.202433699	-3.766908349	-7.002070102	-1.3021922
ENSG00000223855	AC147651.3		2.986136609	1.333432818	18.16999596	2.02E-05	0.000402583	-1.034594152	0.133828548	0.632982468	2.572971709	1.688229459
ENSG00000196209	SIRPB2	signal-regulatory protein beta 2 [Source:HGNC Symbol;Acc:16247]	1.782762529	5.643306681	18.16604384	2.02E-05	0.000402962	4.149649391	4.976626419	5.656303354	6.584276941	5.754799245
ENSG00000272625	RP11-737O24.5		2.772172365	0.360165946	18.15361707	2.04E-05	0.000405141	-1.350769713	-0.583862591	0.125863448	1.296595379	0.615745481
ENSG00000187902	SHISA7	shisa family member 7 [Source:HGNC Symbol;Acc:35409]	-8.173336294	-0.162578136	18.13251095	2.06E-05	0.000409193	0.365960855	1.237938318	-7.002070102	-7.002070102	-1.083770229
ENSG00000164694	FNDC1	fibronectin type III domain containing 1 [Source:HGNC Symbol;Acc:21184]	-4.444023385	-0.031240429	18.08607085	2.11E-05	0.000418821	1.268770164	1.010487126	-7.002070102	-4.730697034	-3.221241555
ENSG00000137309	HMGA1	high mobility group AT-hook 1 [Source:HGNC Symbol;Acc:5010]	-1.528056799	7.332690561	18.0791247	2.12E-05	0.000419878	7.997490037	7.929748255	6.581999324	6.495867034	6.909872245
ENSG00000160298	C21orf58	chromosome 21 open reading frame 58 [Source:HGNC Symbol;Acc:1300]	-1.576695151	4.026505675	18.07470313	2.12E-05	0.000420379	4.449416135	4.702587364	2.918097532	2.311762486	4.377683051
ENSG00000260528	FAM157C	family with sequence similarity 157, member C [Source:HGNC Symbol;Acc:34081]	2.781717162	3.871801942	18.05771739	2.14E-05	0.000423669	1.819824215	1.955327308	2.532495892	5.159976647	4.583141364
ENSG00000166483	WEE1	WEE1 G2 checkpoint kinase [Source:HGNC Symbol;Acc:12761]	-1.561335935	4.087353338	18.03770017	2.17E-05	0.000427665	4.825203682	4.707344697	2.997340322	2.756300061	3.95282564
ENSG00000235323	COTL1P2	coactosin-like 1 (Dictyostelium) pseudogene 2 [Source:HGNC Symbol;Acc:24322]	1.663614044	4.53792989	18.00932839	2.20E-05	0.000433599	3.424338996	4.048714319	4.704544584	5.263422351	4.591098498
ENSG00000138623	SEMA7A	semaphorin 7A, GPI membrane anchor (John Milton Hagen blood group) [Source:HGNC Symbol;Acc:10741]	-1.629099503	3.855623214	17.99823465	2.21E-05	0.000435644	4.654105544	4.571308358	2.548775847	2.615771827	3.478147665
ENSG00000261596	CTB-31N19.3		2.30618842	2.023358443	17.98712023	2.22E-05	0.000437703	0.657137645	1.050973916	1.932051102	2.824787032	2.461903625
ENSG00000197253	TPSB2	tryptase beta 2 (gene/pseudogene) [Source:HGNC Symbol;Acc:14120]	-3.627205534	2.509209291	17.93009957	2.29E-05	0.000450509	4.52961579	1.355744383	-1.57307192	-0.146146183	0.415212929
ENSG00000178175	ZNF366	zinc finger protein 366 [Source:HGNC Symbol;Acc:18316]	2.815835086	0.248327835	17.90115779	2.33E-05	0.0004569	-2.108017771	-0.157588231	0.401591237	0.669468625	0.706443614
ENSG00000152253	SPC25	SPC25, NDC80 kinetochore complex component [Source:HGNC Symbol;Acc:24031]	-2.23840744	1.91648747	17.89447821	2.33E-05	0.000457994	3.036645193	2.521424373	-0.82139248	-0.350682213	1.71760268
ENSG00000134780	DAGLA	diacylglycerol lipase, alpha [Source:HGNC Symbol;Acc:1165]	2.255637879	2.809222642	17.88675258	2.34E-05	0.000459344	0.874315765	2.08771013	2.873554121	3.774480849	2.938038295
ENSG00000235117	RP11-229P13.20		1.89130817	2.287279981	17.87870646	2.35E-05	0.000460776	1.16956809	1.494316873	2.814185793	2.475638093	2.701261324
ENSG00000256433	RP1-102E24.8		3.68014212	-0.323788572	17.82311387	2.42E-05	0.000473908	-4.650190627	-1.394066322	-1.097244829	0.58582888	0.415212929
ENSG00000211899	IGHM	immunoglobulin heavy constant mu [Source:HGNC Symbol;Acc:5541]	-2.383073493	10.67707846	17.80672046	2.45E-05	0.000477477	12.06634136	11.14435456	8.54751425	8.866139395	9.631185998
ENSG00000225936	RP11-501J20.5		2.530789636	1.587087557	17.78101196	2.48E-05	0.000483434	-0.196880952	0.277241192	1.360908506	2.664151552	1.908000984
ENSG00000116991	SIPA1L2	signal-induced proliferation-associated 1 like 2 [Source:HGNC Symbol;Acc:23800]	1.98024839	3.341819341	17.76889706	2.49E-05	0.000485982	1.79341104	1.965953212	2.905510557	3.963082217	4.321397158
ENSG00000133739	LRRCC1	leucine rich repeat and coiled-coil centrosomal protein 1 [Source:HGNC Symbol;Acc:29373]	-1.546452266	4.012182014	17.75953261	2.51E-05	0.000487839	4.521592368	4.67694653	2.955212673	2.420374188	4.227527885
ENSG00000140479	PCSK6	proprotein convertase subtilisin/kexin type 6 [Source:HGNC Symbol;Acc:8569]	2.687528131	3.240862835	17.73932557	2.53E-05	0.000492502	0.329658892	1.965953212	2.627528952	4.400553776	3.860382081

ENSG00000182310	SPACA6P	sperm acrosome associated 6, pseudogene [Source:HGNC Symbol;Acc:27113]	2.435360437	0.14160091	17.71647492	2.56E-05	0.000497902	-1.132483902	-0.252938016	0.036330944	0.722650538	0.341656497
ENSG00000166508	MCM7	minichromosome maintenance complex component 7 [Source:HGNC Symbol;Acc:6950]	-1.579564498	7.380988375	17.71342209	2.57E-05	0.000498151	7.950637677	8.025555626	6.762964825	6.226350987	7.146608044
ENSG00000196565	HBG2	hemoglobin, gamma G [Source:HGNC Symbol;Acc:4832]	-3.046683997	4.467338929	17.71082316	2.57E-05	0.000498282	5.592012371	5.597792608	3.425312536	-0.589083246	1.134350211
ENSG00000168497	SDPR	serum deprivation response [Source:HGNC Symbol;Acc:10690]	2.235255862	6.501993429	17.63511624	2.68E-05	0.000517372	4.633778887	5.33121339	5.892283867	7.774693597	6.7736102
ENSG00000152503	TRIM36	tripartite motif containing 36 [Source:HGNC Symbol;Acc:16280]	2.497873766	0.615813147	17.63693869	2.67E-05	0.000517372	-0.775407551	0.056395802	0.210162819	1.381723529	0.997359302
ENSG00000182022	CHST15	carbohydrate (N-acetylgalactosamine 4-sulfate 6-O) sulfotransferase 15 [Source:HGNC Symbol;Acc:18137]	1.806903198	6.788033246	17.63067355	2.68E-05	0.000518012	5.298828106	5.64732062	6.446286176	7.858201765	7.165382769
ENSG00000143226	FCGR2A	Fc fragment of IgG, low affinity IIa, receptor (CD32) [Source:HGNC Symbol;Acc:3616]	1.822780633	7.155258899	17.6149041	2.70E-05	0.000521751	5.492903791	6.050702052	6.91748469	7.999072743	7.810023262
ENSG00000170323	FABP4	fatty acid binding protein 4, adipocyte [Source:HGNC Symbol;Acc:3559]	-8.080298762	-0.399194143	17.58945025	2.74E-05	0.000528201	1.210071084	0.095631612	-7.002070102	-7.002070102	-3.221241555
ENSG00000225177	RP11-390P2.4		2.424979197	0.157580426	17.55249329	2.79E-05	0.000537976	-1.132483902	-0.523165112	0.28980717	0.917704421	0.04992943
ENSG00000164070	HSPA4L	heat shock 70kDa protein 4-like [Source:HGNC Symbol;Acc:17041]	-2.788181923	0.998465502	17.54974234	2.80E-05	0.000538165	2.015339542	2.048255313	-4.621297774	-2.205328685	0.303422373
ENSG00000070731	ST6GALNAC2	ST6 (alpha-N-acetyl-neuraminyl-2,3-beta-galactosyl-1,3)-N-acetylgalactosaminide alpha-2,6-sialyltransferase 2 [Source:HGNC Symbol;Acc:10867]	2.282578368	2.6659139	17.54187005	2.81E-05	0.000539806	1.431768352	1.010487126	2.150470541	3.427354029	3.532875847
ENSG00000117114	LPHN2	latrophilin 2 [Source:HGNC Symbol;Acc:18582]	-7.843227794	-0.573210396	17.53294732	2.82E-05	0.000541497	0.657137645	0.468724906	-7.002070102	-7.002070102	-3.754655114
ENSG00000105808	RASA4	RAS p21 protein activator 4 [Source:HGNC Symbol;Acc:23181]	2.446249775	1.913442503	17.53176729	2.83E-05	0.000541497	0.174562846	1.355744383	1.89432737	2.947141733	1.774613685
ENSG00000272168	CASC15	cancer susceptibility candidate 15 (non-protein coding) [Source:HGNC Symbol;Acc:28245]	3.113867159	-0.358716023	17.52737724	2.83E-05	0.000542157	-1.756270337	-2.475439898	-2.073364206	0.773941511	0.264147325
ENSG00000269925	RP3-467L1.6		3.069470153	0.502254798	17.4959238	2.88E-05	0.0005506	-0.775407551	-1.100062965	-0.215316613	1.75796486	0.584199832
ENSG00000183853	KIRREL	kin of IRRE like (Drosophila) [Source:HGNC Symbol;Acc:15734]	-3.668526536	0.319734583	17.47492427	2.91E-05	0.00055611	1.79341104	1.147482679	-3.233942493	-7.002070102	-2.832686105
ENSG00000232486	RP11-18B3.2		-2.607603653	0.626088893	17.47119157	2.92E-05	0.000556597	1.46561866	1.418980604	-2.845623393	-2.205328685	0.706443614
ENSG00000228519	RP11-298C3.2		-1.388118335	5.022815489	17.46262311	2.93E-05	0.000558505	5.737656593	5.550039086	4.110688288	3.808701883	4.943166794
ENSG00000167703	SLC43A2	solute carrier family 43 (amino acid system L transporter), member 2 [Source:HGNC Symbol;Acc:23087]	1.800813717	7.000392982	17.45155056	2.95E-05	0.000561158	5.487765045	6.032493671	6.790180281	8.074686826	7.221886413
ENSG00000204131	NHSL2	NHS-like 2 [Source:HGNC Symbol;Acc:33737]	1.554888375	4.620172576	17.44404755	2.96E-05	0.000562767	3.298512169	3.830594178	4.68073531	5.351527589	5.007010895
ENSG00000238113	RP11-262H14.1		2.094325404	4.197519923	17.39949442	3.03E-05	0.00057549	2.695223288	3.476530889	4.094144628	5.425567145	3.782605494
ENSG00000211665	IGLV3-16	immunoglobulin lambda variable 3-16 [Source:HGNC Symbol;Acc:5901]	-3.062810018	0.594810574	17.39286455	3.04E-05	0.000576876	2.093195522	0.904001603	-2.845623393	-3.360122781	-0.147769603
ENSG00000109084	TMEM97	transmembrane protein 97 [Source:HGNC Symbol;Acc:28106]	-1.708508372	3.053524934	17.37125444	3.07E-05	0.000582843	3.780925818	3.774274833	1.802268781	1.567613022	2.874258264
ENSG00000059573	ALDH18A1	aldehyde dehydrogenase 18 family, member A1 [Source:HGNC Symbol;Acc:9722]	-1.507772031	5.633145823	17.3467032	3.11E-05	0.000589784	6.260024507	6.294656685	5.178645532	4.046271435	5.321115655
ENSG00000250510	GPR162	G protein-coupled receptor 162 [Source:HGNC Symbol;Acc:16693]	2.376462237	3.892439641	17.31813031	3.16E-05	0.000598073	1.546924617	2.671789775	3.242232575	4.976471071	4.569109895
ENSG00000184113	CLDN5	claudin 5 [Source:HGNC Symbol;Acc:2047]	3.032165627	1.390282936	17.28661635	3.21E-05	0.00060742	-0.856736938	0.171040209	0.538319988	2.848906753	1.298855074
ENSG00000105270	CLIP3	CAP-GLY domain containing linker protein 3 [Source:HGNC Symbol;Acc:24314]	2.23842148	0.625993489	17.25966978	3.26E-05	0.000615433	-0.046168491	-0.30308518	0.805492218	1.048430793	0.898226269
ENSG00000173559	NABP1	nucleic acid binding protein 1 [Source:HGNC Symbol;Acc:26232]	1.467244738	6.030108833	17.25196877	3.27E-05	0.000617269	5.113748858	5.076838692	6.060680796	6.612289061	6.54744251
ENSG00000137168	PPIL1	peptidylprolyl isomerase (cyclophilin)-like 1 [Source:HGNC Symbol;Acc:9260]	-1.619546852	4.169678348	17.24940671	3.28E-05	0.000617439	5.001408664	4.817053504	3.5025636	2.460063232	3.659358136
ENSG00000166523	CLEC4E	C-type lectin domain family 4, member E [Source:HGNC Symbol;Acc:14555]	1.864982124	5.11309732	17.24522247	3.29E-05	0.000618137	3.424338996	4.180008374	4.838040135	5.972180623	5.715301268
ENSG00000236537	RP11-732M18.3		2.334118139	-0.009812258	17.2293268	3.31E-05	0.000622663	-0.698419327	-1.014020025	0.125863448	0.154058352	0.485200289
ENSG00000166527	CLEC4D	C-type lectin domain family 4, member D [Source:HGNC Symbol;Acc:14554]	2.19428068	4.012347985	17.18905218	3.38E-05	0.000635324	2.463579632	2.796091168	3.442840456	5.043320185	4.591098498
ENSG00000105011	ASF1B	anti-silencing function 1B histone chaperone [Source:HGNC Symbol;Acc:20996]	-1.543254389	4.039627094	17.17792629	3.40E-05	0.000638373	4.476647738	4.637610028	2.650340322	2.749909693	4.445086256

ENSG00000168393	DTYMK	deoxythymidylate kinase (thymidylate kinase) [Source:HGNC Symbol;Acc:3061]	-1.494465646	4.480325237	17.16597028	3.43E-05	0.000641034	5.252314135	5.053365096	3.799846468	3.130496995	4.113376315
ENSG00000255328	RP11-326C3.12		2.322498046	-0.128330922	17.16696589	3.42E-05	0.000641034	-0.775407551	-0.932821174	0.471574783	0.669468625	-2.274700861
ENSG00000170779	CDCA4	cell division cycle associated 4 [Source:HGNC Symbol;Acc:14625]	-1.373550487	4.863584795	17.14149086	3.47E-05	0.000647967	5.464934547	5.485959793	4.153899108	3.774480849	4.632168294
ENSG00000273272	CTA-384D8.34		3.564418676	0.688193084	17.14285462	3.47E-05	0.000647967	-1.47369337	-1.191564836	-1.438615935	1.992567844	1.3928929
ENSG00000253981	ALG1L13P	asparagine-linked glycosylation 1-like 13, pseudogene [Source:HGNC Symbol;Acc:44382]	2.700102379	0.106960636	17.12846018	3.49E-05	0.000651734	-0.942926472	-1.014020025	-0.99931534	1.12943269	0.485200289
ENSG00000165959	CLMN	calmin (calponin-like, transmembrane) [Source:HGNC Symbol;Acc:19972]	2.054668241	5.122079124	17.12033392	3.51E-05	0.000653834	3.733087128	3.590786525	4.426992082	6.298184651	5.593838124
ENSG00000212127	TAS2R14	taste receptor, type 2, member 14 [Source:HGNC Symbol;Acc:14920]	2.292316043	-0.173354284	17.07084044	3.60E-05	0.000670385	-0.942926472	-0.713501401	0.28980717	0.229488395	-0.649489103
ENSG00000164104	HMGB2	high mobility group box 2 [Source:HGNC Symbol;Acc:5000]	-1.527980374	7.125228825	17.06278705	3.62E-05	0.000672521	7.59781201	7.694043382	6.468919368	5.938858325	7.199940107
ENSG00000137266	SLC22A23	solute carrier family 22, member 23 [Source:HGNC Symbol;Acc:21106]	-1.701915111	3.816725701	17.04648162	3.65E-05	0.000677602	4.449416135	4.72152329	3.133097051	2.086853261	3.152175675
ENSG00000169515	CCDC8	coiled-coil domain containing 8 [Source:HGNC Symbol;Acc:25367]	-8.089704138	-0.22944329	17.00804088	3.72E-05	0.000690729	1.127895066	0.277241192	-7.002070102	-7.002070102	-0.80787826
ENSG00000184497	TMEM255B	transmembrane protein 255B [Source:HGNC Symbol;Acc:28297]	2.731091691	-0.220163459	16.99196363	3.75E-05	0.000695867	-1.756270337	-0.713501401	-1.202308962	0.58582888	0.223773082
ENSG00000241566	IGKV2D-24	immunoglobulin kappa variable 2D-24 (non-functional) [Source:HGNC Symbol;Acc:5797]	-3.093789355	0.684778121	16.98207835	3.77E-05	0.000698762	2.187493018	0.904001603	-2.073364206	-4.730697034	0.095393329
ENSG00000189316	RP11-797H7.5		-3.370203382	0.325315861	16.95564376	3.83E-05	0.000707812	1.806678074	0.925936776	-7.002070102	-2.97427061	-1.18885156
ENSG00000189127	ANKRD34B	ankyrin repeat domain 34B [Source:HGNC Symbol;Acc:33736]	2.338323989	-0.003600961	16.94081057	3.86E-05	0.000712614	-0.425964666	-0.855949929	0.365283811	0.696304622	-0.9858258
ENSG00000151948	GLT1D1	glycosyltransferase 1 domain containing 1 [Source:HGNC Symbol;Acc:26483]	1.870982414	5.652804699	16.93213795	3.87E-05	0.000715124	4.099324826	4.560832138	5.12807726	6.723686917	6.126640144
ENSG00000272053	RP11-367G6.3		1.942652143	2.671667741	16.92249411	3.89E-05	0.000718011	1.832851647	1.660530868	2.867077005	3.497693538	2.648857997
ENSG00000105767	CADM4	cell adhesion molecule 4 [Source:HGNC Symbol;Acc:30825]	2.303773488	0.410871289	16.89779405	3.94E-05	0.000726652	-0.856736938	0.016063025	0.471574783	0.894716898	0.646616095
ENSG00000262944	RP11-473I1.9		1.537675044	5.388163893	16.89274125	3.96E-05	0.000727825	4.557351498	4.662320742	5.486054045	6.275562429	5.246248345
ENSG00000079308	TNS1	tensin 1 [Source:HGNC Symbol;Acc:11973]	-2.035912801	4.71434713	16.88624234	3.97E-05	0.000729556	4.922864659	5.880018163	2.253389193	3.938003897	4.431855191
ENSG00000213876	RPL7AP64	ribosomal protein L7a pseudogene 64 [Source:HGNC Symbol;Acc:35821]	2.34380476	2.617006297	16.87148994	4.00E-05	0.00073448	0.796654307	1.647388141	2.150470541	3.520398046	3.265899528
ENSG00000137210	TMEM14B	transmembrane protein 14B [Source:HGNC Symbol;Acc:21384]	-1.386754391	5.194221186	16.86721186	4.01E-05	0.0007346	5.913510612	5.815385668	4.628078944	4.156672678	4.561030213
ENSG00000074964	ARHGEF10L	Rho guanine nucleotide exchange factor (GEF) 10-like [Source:HGNC Symbol;Acc:25540]	1.943318616	5.076875826	16.86728989	4.01E-05	0.0007346	3.008216069	4.277066647	4.842999345	6.135513082	5.393426727
ENSG00000116062	MSH6	mutS homolog 6 [Source:HGNC Symbol;Acc:7329]	-1.37262096	5.84695732	16.85947623	4.03E-05	0.000736064	6.350560454	6.371097946	5.289363194	4.701415938	5.858153151
ENSG00000205085	FAM71F2	family with sequence similarity 71, member F2 [Source:HGNC Symbol;Acc:27998]	2.704235493	0.015327786	16.86061976	4.02E-05	0.000736064	-1.921450438	-0.355038393	-0.453950945	0.962608982	0.04992943
ENSG00000170190	SLC16A5	solute carrier family 16 (monocarboxylate transporter), member 5 [Source:HGNC Symbol;Acc:10926]	1.545846953	4.681785177	16.84872421	4.05E-05	0.000739475	3.650879106	4.056192383	4.789201202	5.461209116	4.76295413
ENSG00000197558	SSPO	SCO-spondin [Source:HGNC Symbol;Acc:21998]	2.037443077	2.276877246	16.82798498	4.09E-05	0.000746823	1.040754067	1.724506406	2.456873398	3.20713662	1.945725455
ENSG00000136867	SLC31A2	solute carrier family 31 (copper transporter), member 2 [Source:HGNC Symbol;Acc:11017]	2.036237704	5.545713858	16.80884686	4.13E-05	0.000753609	3.875243923	4.447875012	4.946464078	6.71304952	5.931278265
ENSG00000169085	C8orf46	chromosome 8 open reading frame 46 [Source:HGNC Symbol;Acc:28498]	2.288592547	0.068157219	16.79452209	4.17E-05	0.000758532	-1.034594152	-0.204475541	0.28980717	0.402426505	0.095393329
ENSG00000111275	ALDH2	aldehyde dehydrogenase 2 family (mitochondrial) [Source:HGNC Symbol;Acc:404]	1.741743625	6.509138819	16.76107164	4.24E-05	0.000771222	5.032796296	5.661281456	6.302481455	7.518773637	6.774483969
ENSG00000237491	RP11-206L10.9		2.347100164	0.40930636	16.74553163	4.27E-05	0.00077676	-0.046168491	-0.583862591	0.75024962	0.894716898	0.264147325
ENSG00000106976	DNM1	dynamin 1 [Source:HGNC Symbol;Acc:2972]	2.20934842	1.930053838	16.72619191	4.32E-05	0.000783907	0.401371743	1.434365866	2.051115931	2.697741416	2.041752268
ENSG00000149503	INCENP	inner centromere protein antigens 135/155kDa [Source:HGNC Symbol;Acc:6058]	-1.376606523	4.830962982	16.71463526	4.34E-05	0.000787881	5.449158537	5.360703406	4.051935967	3.579252501	4.843435205
ENSG00000162543	UBXN10	UBX domain protein 10 [Source:HGNC Symbol;Acc:26354]	-1.762725771	2.970510757	16.69935724	4.38E-05	0.000792387	3.951879815	3.641380386	2.073793572	1.168289627	2.164148811
ENSG00000139318	DUSP6	dual specificity phosphatase 6 [Source:HGNC Symbol;Acc:3072]	1.810521733	7.67343262	16.70082462	4.38E-05	0.000792387	5.840407077	6.477503674	7.50502584	8.487755024	8.381196346

ENSG00000198121	LPAR1	lysophosphatidic acid receptor 1 [Source:HGNC Symbol;Acc:3166]	2.601012818	2.661408141	16.69794669	4.38E-05	0.000792387	0.769809504	1.272590116	1.613958159	3.865963837	3.260920169
ENSG00000124731	TREM1	triggering receptor expressed on myeloid cells 1 [Source:HGNC Symbol;Acc:17760]	2.657101643	6.449345854	16.68000471	4.42E-05	0.000799094	4.366735481	4.507278613	4.960222513	7.805639866	7.112807219
ENSG00000104093	DMXL2	Dmx-like 2 [Source:HGNC Symbol;Acc:2938]	1.982681764	7.327144822	16.67467645	4.44E-05	0.000800518	5.212995974	6.32024472	7.112173624	8.420819471	7.676397272
ENSG00000005189	AC004381.6	Putative RNA exonuclease NEF-sp [Source:UniProtKB/Swiss-Prot;Acc:Q96IC2]	-1.882609698	2.621978488	16.66296301	4.46E-05	0.000804649	3.479226043	3.315529363	0.401591237	0.962608982	2.610226292
ENSG00000161911	TREML1	triggering receptor expressed on myeloid cells-like 1 [Source:HGNC Symbol;Acc:20434]	2.001483449	4.269141775	16.64591498	4.50E-05	0.00081025	2.038016118	3.411939754	4.175028678	5.127069289	4.826683557
ENSG00000115155	OTOF	otoferlin [Source:HGNC Symbol;Acc:8515]	2.27391729	0.295090576	16.64781036	4.50E-05	0.00081025	-0.856736938	0.016063025	0.983710868	0.335717742	0.095393329
ENSG00000135709	KIAA0513	KIAA0513 [Source:HGNC Symbol;Acc:29058]	1.641144877	6.776067954	16.62590072	4.55E-05	0.000818007	5.389790814	5.938510869	6.646769245	7.780387605	6.958738962
ENSG00000138798	EGF	epidermal growth factor [Source:HGNC Symbol;Acc:3229]	2.513966276	2.404509516	16.5643215	4.70E-05	0.000844138	0.046214424	1.464652754	2.073793572	3.284338193	3.080504462
ENSG00000225101	OR52K3P	olfactory receptor, family 52, subfamily K, member 3 pseudogene [Source:HGNC Symbol;Acc:15224]	2.854062136	2.403576574	16.55515495	4.73E-05	0.000847362	-0.094677427	1.010487126	1.245796404	3.524147633	3.225574983
ENSG00000254017	IGHEP2	immunoglobulin heavy constant epsilon P2 (pseudogene) [Source:HGNC Symbol;Acc:5524]	3.519050424	0.962761299	16.54817548	4.74E-05	0.000849619	-0.555769858	-1.289265519	-1.097244829	2.483362921	1.198257616
ENSG00000244482	LILRA6	leukocyte immunoglobulin-like receptor, subfamily A (with TM domain), member 6 [Source:HGNC Symbol;Acc:15495]	1.916097381	6.367768435	16.51352267	4.83E-05	0.000864406	4.652269455	5.522410173	6.05071266	7.500808448	6.55610665
ENSG00000141497	ZMYND15	zinc finger, MYND-type containing 15 [Source:HGNC Symbol;Acc:20997]	1.985010667	2.229573032	16.47335807	4.93E-05	0.000882014	0.823008709	1.579816252	2.650340322	3.105687454	1.81594051
ENSG00000100292	HMOX1	heme oxygenase (decycling) 1 [Source:HGNC Symbol;Acc:5013]	1.513303236	6.272193368	16.47059839	4.94E-05	0.0008824	5.10168997	5.422763437	6.225453304	7.080151331	6.626514479
ENSG00000154146	NRGN	neurogranin (protein kinase C substrate, RC3) [Source:HGNC Symbol;Acc:8000]	1.955781251	7.296979618	16.4502512	4.99E-05	0.000891016	5.253525726	6.100977991	6.902518994	8.345602691	7.87215837
ENSG00000181938	GINS3	GINS complex subunit 3 (Psf3 homolog) [Source:HGNC Symbol;Acc:25851]	-1.764564593	2.657902903	16.43232883	5.04E-05	0.000898566	3.371751013	3.344320706	0.959559782	1.006157955	2.765955603
ENSG00000114346	ECT2	epithelial cell transforming sequence 2 oncogene [Source:HGNC Symbol;Acc:3155]	-1.697188308	3.874582132	16.422239	5.07E-05	0.000902445	4.597980204	4.468572057	2.243421497	2.737043408	3.9341793
ENSG00000226046	AC007316.3		1.913019608	2.328199481	16.41194014	5.10E-05	0.000906443	1.361587606	1.634124584	2.465474326	2.895969157	2.633529225
ENSG00000261015	RP1-90J20.11		2.191131795	1.099235585	16.37433281	5.20E-05	0.000923499	-0.555769858	-0.157588231	0.884581662	1.381723529	2.153441144
ENSG00000207980	MIR23A	microRNA 23a [Source:HGNC Symbol;Acc:31605]	3.366518776	-0.810112641	16.37276764	5.20E-05	0.000923499	-2.322346157	-7.002070102	-2.845623393	0.192266291	0.139468177
ENSG00000008516	MMP25	matrix metallopeptidase 25 [Source:HGNC Symbol;Acc:14246]	2.202812023	6.193616418	16.35573848	5.25E-05	0.000930893	4.659599832	4.083285021	5.132134572	7.160342999	7.183587426
ENSG00000183773	AIFM3	apoptosis-inducing factor, mitochondrion-associated, 3 [Source:HGNC Symbol;Acc:26398]	2.053945206	3.399279123	16.35339982	5.26E-05	0.000931102	1.819824215	2.612158157	3.201624573	4.442766639	3.569583953
ENSG00000198039	ZNF273	zinc finger protein 273 [Source:HGNC Symbol;Acc:13067]	-1.662721631	3.871503624	16.34973056	5.27E-05	0.000931545	4.668710735	4.630948391	3.186096972	1.981700954	3.265899528
ENSG00000112902	SEMA5A	sema domain, seven thrombospondin repeats (type 1 and type 1-like), transmembrane domain (TM) and short cytoplasmic domain, (semaphorin) 5A [Source:HGNC Symbol;Acc:10736]	2.299333112	0.408837131	16.34867671	5.27E-05	0.000931545	-1.132483902	-0.408932621	0.471574783	0.962608982	0.872340852
ENSG00000261356	RP11-46D6.5		1.776295991	3.419687103	16.32888272	5.32E-05	0.000940379	2.146341971	2.66528446	3.29626549	3.886500298	4.159591272
ENSG00000121957	GPSM2	G-protein signaling modulator 2 [Source:HGNC Symbol;Acc:29501]	-1.797604894	3.858577692	16.32649204	5.33E-05	0.00094062	4.484924323	4.474165552	1.629349082	2.622783341	4.199174923
ENSG00000105520	DKFZP761J1410	Lipid phosphate phosphatase-related protein type 2 [Source:UniProtKB/Swiss-Prot;Acc:Q96GM1]	1.559820134	5.712822866	16.30709834	5.39E-05	0.000949341	4.440932708	4.977939425	5.663796734	6.594120875	5.940650325
ENSG00000219088	RP3-359N14.1		-2.030971026	1.642614869	16.30171967	5.40E-05	0.000950842	2.504862786	2.361613919	-0.329705368	-0.296766077	1.531661491
ENSG00000174125	TLR1	toll-like receptor 1 [Source:HGNC Symbol;Acc:11847]	1.622149767	5.917028912	16.30030448	5.41E-05	0.000950842	4.505410405	4.929901731	5.554165184	6.580230437	6.727880313
ENSG00000111142	METAP2	methionyl aminopeptidase 2 [Source:HGNC Symbol;Acc:16672]	-1.307536487	6.136774894	16.29734171	5.41E-05	0.000951376	6.716179048	6.772041963	5.56621082	5.174364615	5.744133867
ENSG00000082397	EPB41L3	erythrocyte membrane protein band 4.1-like 3 [Source:HGNC Symbol;Acc:3380]	1.598212537	5.109146108	16.2580158	5.53E-05	0.000969711	3.628640226	4.50181228	5.231288657	5.922614461	5.287231979
ENSG00000197852	FAM212B	family with sequence similarity 212, member B [Source:HGNC Symbol;Acc:28045]	2.289200146	4.922409794	16.25738294	5.53E-05	0.000969711	3.260449604	3.286151722	3.853256341	6.068406509	5.656795653
ENSG00000167261	DPEP2	dipeptidase 2 [Source:HGNC Symbol;Acc:23028]	1.371237888	6.288956797	16.23662201	5.59E-05	0.000979417	5.36309909	5.671866971	6.571081935	7.070247415	6.11012241
ENSG00000258734	RP11-112J1.3		-3.00667232	0.194509381	16.18889054	5.73E-05	0.001003092	1.268770164	1.237938318	-7.002070102	-2.670141381	-1.083770229

ENSG00000263823	RP11-326K13.4		2.213372876	-0.121648939	16.18759677	5.74E-05	0.001003092	-0.775407551	-0.782968247	0.570569237	0.229488395	-0.9858258
ENSG00000204103	MAFB	v-maf avian musculoaponeurotic fibrosarcoma oncogene homolog B [Source:HGNC Symbol;Acc:6408]	1.858905816	6.532016841	16.18417977	5.75E-05	0.001003903	5.007166533	5.132136233	5.840325445	7.592724165	7.178645258
ENSG00000136108	CKAP2	cytoskeleton associated protein 2 [Source:HGNC Symbol;Acc:1990]	-1.384450713	5.197696074	16.16783955	5.80E-05	0.001011594	5.70082528	5.764556871	3.993665308	4.199509687	5.410390022
ENSG00000253368	TRNP1	TMF1-regulated nuclear protein 1 [Source:HGNC Symbol;Acc:34348]	2.213573222	0.203969049	16.16179383	5.82E-05	0.00101382	-0.625332215	-0.583862591	0.250534523	0.669468625	0.485200289
ENSG00000179869	ABCA13	ATP-binding cassette, sub-family A (ABC1), member 13 [Source:HGNC Symbol;Acc:14638]	-2.387248586	3.544292489	16.15062767	5.85E-05	0.001018803	3.650879106	1.976501425	-0.907612816	0.61425099	5.307898957
ENSG00000102743	SLC25A15	solute carrier family 25 (mitochondrial carrier; ornithine transporter) member 15 [Source:HGNC Symbol;Acc:10985]	-1.687306544	3.127654011	16.1392869	5.89E-05	0.001023905	4.021807945	3.810099846	2.161099145	1.296595379	2.633529225
ENSG00000180914	OXTR	oxytocin receptor [Source:HGNC Symbol;Acc:8529]	2.178148987	0.216773418	16.13082131	5.91E-05	0.001027474	-0.775407551	-0.112176905	0.505333341	0.497040107	0.264147325
ENSG00000180061	TMEM150B	transmembrane protein 150B [Source:HGNC Symbol;Acc:34415]	2.100476422	3.228104116	16.05080669	6.17E-05	0.001070757	1.608823392	2.361613919	2.899175647	4.238831357	3.589580856
ENSG00000134779	TPGS2	tubulin polyglutamylase complex subunit 2 [Source:HGNC Symbol;Acc:24561]	-1.286236201	6.061499719	16.04175031	6.20E-05	0.001073819	6.694039461	6.700275842	5.514307435	5.279040176	5.377412319
ENSG00000120949	TNFRSF8	tumor necrosis factor receptor superfamily, member 8 [Source:HGNC Symbol;Acc:11923]	2.350527143	3.697057899	16.04165805	6.20E-05	0.001073819	1.696960037	2.584842899	2.905510557	4.939041784	4.099497077
ENSG00000129219	PLD2	phospholipase D2 [Source:HGNC Symbol;Acc:9068]	1.4392118	4.776228438	16.01664098	6.28E-05	0.001087028	3.784282926	4.289810032	5.022748641	5.467064767	4.736293913
ENSG00000122863	CHST3	carbohydrate (chondroitin 6) sulfotransferase 3 [Source:HGNC Symbol;Acc:1971]	-4.22979662	-0.289445491	15.98980752	6.37E-05	0.001101458	0.874315765	0.836121102	-4.621297774	-7.002070102	-3.221241555
ENSG00000149054	ZNF215	zinc finger protein 215 [Source:HGNC Symbol;Acc:13007]	-2.546412419	1.354707007	15.98687543	6.38E-05	0.00110208	2.5449974	1.85599892	-1.721358966	-2.97427061	1.3928929
ENSG00000104522	TSTA3	tissue specific transplantation antigen P35B [Source:HGNC Symbol;Acc:12390]	-1.282679681	5.605624038	15.98118815	6.40E-05	0.001104309	6.197188107	6.147811841	5.195546149	4.817496626	5.089370998
ENSG00000130962	PRRG1	proline rich Gla (G-carboxyglutamic acid) 1 [Source:HGNC Symbol;Acc:9469]	2.244672476	-0.150784987	15.9696464	6.44E-05	0.00110997	-0.365194284	-0.713501401	0.036330944	0.61425099	-1.87334355
ENSG00000123095	BHLHE41	basic helix-loop-helix family, member e41 [Source:HGNC Symbol;Acc:16617]	-2.080385587	1.881858853	15.96357573	6.46E-05	0.001112443	3.08644716	2.287305009	0.036330944	0.074465887	1.428856327
ENSG00000170893	TRH	thyrotropin-releasing hormone [Source:HGNC Symbol;Acc:12298]	-3.663524958	0.455454996	15.93918513	6.54E-05	0.001125765	0.742455703	1.523383318	-7.002070102	-3.888428289	0.948644093
ENSG00000182326	C1S	complement component 1, s subcomponent [Source:HGNC Symbol;Acc:1247]	-2.119155293	1.767290866	15.93224045	6.57E-05	0.00112876	3.091876065	2.549955088	-0.010608983	-0.589083246	-0.57637152
ENSG00000010165	METTL13	methyltransferase like 13 [Source:HGNC Symbol;Acc:24248]	-1.328390622	5.587477065	15.93044741	6.57E-05	0.00112876	6.267240271	6.051949372	4.994798186	4.45852833	5.422704219
ENSG00000196664	TLR7	toll-like receptor 7 [Source:HGNC Symbol;Acc:15631]	1.560397692	5.048190009	15.92301843	6.60E-05	0.001130046	3.606053165	4.33559574	4.961743161	5.482564629	5.83153188
ENSG00000269220	LINC00528	long intergenic non-protein coding RNA 528 [Source:HGNC Symbol;Acc:26875]	1.801831743	3.426746986	15.92406799	6.59E-05	0.001130046	2.744312733	2.270258944	3.481516588	4.386203812	3.305127547
ENSG00000137462	TLR2	toll-like receptor 2 [Source:HGNC Symbol;Acc:11848]	2.021788213	6.975346138	15.9227446	6.60E-05	0.001130046	5.078633344	5.83652954	6.482754511	8.00528999	7.581732434
ENSG00000180891	CUEDC1	CUE domain containing 1 [Source:HGNC Symbol;Acc:31350]	1.961496506	4.069412988	15.8888201	6.72E-05	0.001148355	2.521050858	3.129558609	3.66819303	5.156357122	4.377683051
ENSG00000197046	SIGLEC15	sialic acid binding Ig-like lectin 15 [Source:HGNC Symbol;Acc:27596]	2.43470737	1.685873338	15.88863673	6.72E-05	0.001148355	-0.306880589	1.010487126	1.534439871	2.428399826	2.236956541
ENSG00000231419	LINC00689	long intergenic non-protein coding RNA 689 [Source:HGNC Symbol;Acc:27217]	2.402439952	0.543308532	15.87121268	6.78E-05	0.001157849	0.174562846	-0.025429826	0.328039018	1.844482366	-7.002070102
ENSG00000183696	UPP1	uridine phosphorylase 1 [Source:HGNC Symbol;Acc:12576]	1.284356456	6.092062498	15.86586312	6.80E-05	0.001159998	5.257154408	5.608852542	6.605049683	6.631969868	5.839068896
ENSG00000114013	CD86	CD86 molecule [Source:HGNC Symbol;Acc:1705]	1.638760754	6.51675101	15.85329834	6.84E-05	0.001166592	5.048237693	5.780445976	6.39430624	7.381088304	6.891061537
ENSG00000153823	PID1	phosphotyrosine interaction domain containing 1 [Source:HGNC Symbol;Acc:26084]	1.558893411	3.993110112	15.84762618	6.87E-05	0.001168959	2.695223288	3.465340561	4.55339651	4.485703603	3.995418829
ENSG00000105472	CLEC11A	C-type lectin domain family 11, member A [Source:HGNC Symbol;Acc:10576]	-1.692648489	3.991557203	15.84480945	6.88E-05	0.001169566	4.240439676	4.479737444	2.02807612	2.830854872	4.747017192
ENSG00000133808	MICALCL	MICAL C-terminal like [Source:HGNC Symbol;Acc:25933]	2.303878288	2.071787816	15.8418715	6.89E-05	0.001170249	0.435934254	1.109650305	1.517996407	2.958271065	2.723150012
ENSG00000172322	CLEC12A	C-type lectin domain family 12, member A [Source:HGNC Symbol;Acc:31713]	1.693966321	6.433575953	15.83378375	6.92E-05	0.001174125	4.913697322	5.601204704	6.171936119	7.239735604	7.022280309
ENSG00000224307	RP11-344B5.2		2.548418853	2.114079852	15.82836178	6.94E-05	0.001176356	0.000762307	0.637745977	1.098750223	3.080443796	3.04038489
ENSG00000228974	AC006483.5		1.622431791	4.366689869	15.8183269	6.97E-05	0.00118147	3.181172573	3.93162677	4.44232286	5.087070373	4.511578169

ENSG00000205730	ITPRIPL2	inositol 1,4,5-trisphosphate receptor interacting protein-like 2 [Source:HGNC Symbol;Acc:27257]	1.615187314	3.485102364	15.81374921	6.99E-05	0.00118205	2.156740297	2.69752161	3.72302193	3.926716498	4.033874407
ENSG00000160013	PTGIR	prostaglandin I2 (prostacyclin) receptor (IP) [Source:HGNC Symbol;Acc:9602]	1.902655813	3.647162487	15.81456675	6.99E-05	0.00118205	1.79341104	3.091246938	4.011394951	4.504805816	3.528738911
ENSG00000216490	IFI30	interferon, gamma-inducible protein 30 [Source:HGNC Symbol;Acc:5398]	3.146234071	3.975290895	15.80698971	7.01E-05	0.001185138	2.227502762	1.565913615	2.139763053	5.791861012	3.439004866
ENSG00000147894	C9orf72	chromosome 9 open reading frame 72 [Source:HGNC Symbol;Acc:28337]	1.795105932	6.849263559	15.7890401	7.08E-05	0.001195285	5.373166446	5.983655362	6.594305985	7.857087322	7.160043447
ENSG00000135525	MAP7	microtubule-associated protein 7 [Source:HGNC Symbol;Acc:6869]	-1.718493081	2.913288376	15.76316191	7.18E-05	0.001210583	3.606053165	3.545455828	1.120696904	1.610570062	3.05196192
ENSG00000134817	APLNR	apelin receptor [Source:HGNC Symbol;Acc:339]	-7.86163599	-0.527308718	15.75746337	7.20E-05	0.001212355	0.742455703	0.277241192	-7.002070102	-7.002070102	-1.87334355
ENSG00000174705	SH3PXD2B	SH3 and PX domains 2B [Source:HGNC Symbol;Acc:29242]	2.47837621	1.457315713	15.75676295	7.20E-05	0.001212355	-0.555769858	0.376157587	-0.059127673	2.126858114	2.625803327
ENSG00000187164	KIAA1598	KIAA1598 [Source:HGNC Symbol;Acc:29319]	1.626347522	5.333815851	15.75136325	7.22E-05	0.001214656	3.903304094	4.699407071	5.339352777	6.280558759	5.399103349
ENSG00000269600	AC016629.3		-7.810534268	-0.530220459	15.74933686	7.23E-05	0.001214794	0.848890306	0.343933181	-7.002070102	-7.002070102	-3.754655114
ENSG00000120885	CLU	clusterin [Source:HGNC Symbol;Acc:2095]	1.823109963	6.635325167	15.7283284	7.31E-05	0.001227186	4.934997859	5.482263587	6.05071266	7.535357162	7.406814325
ENSG00000132122	SPATA6	spermatogenesis associated 6 [Source:HGNC Symbol;Acc:18309]	1.930727024	2.968459622	15.72084101	7.34E-05	0.001230876	1.46561866	2.181869923	2.886421762	3.868915576	3.255923565
ENSG00000068489	PRR11	proline rich 11 [Source:HGNC Symbol;Acc:25619]	-1.572560137	4.479538181	15.70485137	7.40E-05	0.001240141	5.138880244	5.078063628	2.860570679	3.568398624	4.530321888
ENSG00000129009	ISLR	immunoglobulin superfamily containing leucine-rich repeat [Source:HGNC Symbol;Acc:6133]	-7.695077952	-0.625149325	15.69418519	7.45E-05	0.001244969	0.657137645	0.343933181	-7.002070102	-7.002070102	-3.754655114
ENSG00000169184	MN1	meningioma (disrupted in balanced translocation) 1 [Source:HGNC Symbol;Acc:7180]	2.299201418	0.31122283	15.69389625	7.45E-05	0.001244969	-0.365194284	-1.014020025	-0.271377577	0.61425099	1.218946104
ENSG00000235105	RP11-329A14.1		-1.363238388	4.56102153	15.68251406	7.49E-05	0.001251258	5.121732481	5.253112255	3.836777169	3.301918251	4.36613654
ENSG00000137393	RNF144B	ring finger protein 144B [Source:HGNC Symbol;Acc:21578]	1.743962119	5.423714901	15.68076658	7.50E-05	0.001251258	4.297940103	4.620897903	5.195546149	6.530299841	5.356557658
ENSG00000271178	IGHV3OR16-13	immunoglobulin heavy variable 3/OR16-13 (non-functional) [Source:HGNC Symbol;Acc:5637]	-4.301429769	-0.147688753	15.67431513	7.52E-05	0.001254341	1.343495699	0.310972527	-4.621297774	-7.002070102	-1.3021922
ENSG00000156273	BACH1	BTB and CNC homology 1, basic leucine zipper transcription factor 1 [Source:HGNC Symbol;Acc:935]	1.536719232	6.809568667	15.66982248	7.54E-05	0.001256131	5.409490446	5.901626523	6.757284464	7.830836215	6.935879984
ENSG00000164047	CAMP	cathelicidin antimicrobial peptide [Source:HGNC Symbol;Acc:1472]	-3.274684894	3.04624742	15.65640019	7.60E-05	0.001262355	4.743052439	1.749322856	-1.315629595	0.696304622	3.157544141
ENSG00000166046	TCP11L2	t-complex 11, testis-specific-like 2 [Source:HGNC Symbol;Acc:28627]	1.306436052	4.952297892	15.65629847	7.60E-05	0.001262355	4.133946251	4.516343393	5.512233461	5.260053564	4.91332993
ENSG00000085514	PILRA	paired immunoglobin-like type 2 receptor alpha [Source:HGNC Symbol;Acc:20396]	1.5365182	6.673005341	15.65509902	7.60E-05	0.001262355	5.374280717	6.097962036	6.788467495	7.589819901	6.596194734
ENSG00000204136	GGTA1P	glycoprotein, alpha-galactosyltransferase 1 pseudogene [Source:HGNC Symbol;Acc:4253]	1.965881604	3.30171527	15.64672873	7.63E-05	0.001266756	1.711139862	2.038220677	2.672796614	3.982290583	4.285069672
ENSG00000242498	C15orf38	chromosome 15 open reading frame 38 [Source:HGNC Symbol;Acc:28782]	1.88202408	3.353770552	15.63648297	7.68E-05	0.001272435	2.237334199	2.921487006	3.622778389	4.623161458	0.615745481
ENSG00000270346	RP1-90J20.12		2.502378824	0.029544286	15.60704972	7.80E-05	0.001291176	-1.608074432	-0.932821174	-0.453950945	0.642123966	0.763888603
ENSG00000177575	CD163	CD163 molecule [Source:HGNC Symbol;Acc:1631]	1.730922486	6.560127283	15.59292931	7.85E-05	0.001299629	4.582637211	5.64732062	6.211460264	7.069285353	7.5628906
ENSG00000104524	PYCRL	pyrroline-5-carboxylate reductase-like [Source:HGNC Symbol;Acc:25846]	-1.664054916	2.93925165	15.55916545	8.00E-05	0.001321532	3.823970458	3.559556046	1.992811462	1.331251704	2.487707366
ENSG00000196559	LINC00610	long intergenic non-protein coding RNA 610 [Source:HGNC Symbol;Acc:23262]	2.381216437	0.579936194	15.55776857	8.00E-05	0.001321532	-0.555769858	-0.355038393	0.401591237	1.706092334	0.182236344
ENSG00000141540	TTYH2	tweety family member 2 [Source:HGNC Symbol;Acc:13877]	1.507204371	5.337492266	15.5410886	8.07E-05	0.001331991	4.215787888	4.76780347	5.362561955	6.196702673	5.377412319
ENSG00000128283	CDC42EP1	CDC42 effector protein (Rho GTPase binding) 1 [Source:HGNC Symbol;Acc:17014]	2.343017335	2.525743486	15.53816809	8.09E-05	0.001332797	0.769809504	1.387708937	1.89432737	3.696873774	2.906500707
ENSG00000182054	IDH2	isocitrate dehydrogenase 2 (NADP+), mitochondrial [Source:HGNC Symbol;Acc:5383]	-1.363475023	7.007225085	15.53100971	8.12E-05	0.001336323	7.46657301	7.63201114	6.199909595	6.277229796	6.865456199
ENSG00000262877	RP11-1055B8.4		2.186483854	2.074831979	15.52962701	8.12E-05	0.001336323	0.174562846	1.387708937	2.253389193	2.924622147	2.226777706
ENSG00000198948	MFAP3L	microfibrillar-associated protein 3-like [Source:HGNC Symbol;Acc:29083]	2.069313496	3.533298495	15.51116758	8.20E-05	0.001348172	2.082327188	2.703883491	3.201624573	4.62141223	3.704069251

ENSG00000137872	SEMA6D	sema domain, transmembrane domain (TM), and cytoplasmic domain, (semaphorin) 6D [Source:HGNC Symbol;Acc:16770]	-7.61305732	-0.709542947	15.50895	8.21E-05	0.001348492	0.597320328	0.242702272	-7.002070102	-7.002070102	-4.610216385
ENSG00000008441	NFIX	nuclear factor I/X (CCAAT-binding transcription factor) [Source:HGNC Symbol;Acc:7788]	-1.597916281	4.437528641	15.49916821	8.25E-05	0.001354222	5.070406928	5.221313549	2.766259878	3.758653379	4.068484455
ENSG00000100304	TTLL12	tubulin tyrosine ligase-like family, member 12 [Source:HGNC Symbol;Acc:28974]	-1.259418032	5.961893314	15.47447791	8.36E-05	0.001370746	6.478860767	6.489521302	5.214803978	5.098432609	5.928925706
ENSG00000185842	DNAH14	dynein, axonemal, heavy chain 14 [Source:HGNC Symbol;Acc:2945]	-3.016079878	0.133824465	15.471322	8.38E-05	0.001371756	1.229903997	1.128690501	-4.621297774	-3.360122781	-0.9858258
ENSG00000170456	DENND5B	DENN/MADD domain containing 5B [Source:HGNC Symbol;Acc:28338]	-1.532770171	3.852408518	15.46792285	8.39E-05	0.001372944	4.613161742	4.279198377	2.702204734	2.697741416	3.912116199
ENSG00000104447	TRPS1	trichorhinophalangeal syndrome I [Source:HGNC Symbol;Acc:12340]	1.42965905	5.071540321	15.46213672	8.42E-05	0.001375872	3.881526851	4.499985557	5.268626356	5.889567528	5.029144542
ENSG00000186010	NDUFA13	NADH dehydrogenase (ubiquinone) 1 alpha subcomplex, 13 [Source:HGNC Symbol;Acc:17194]	2.184635919	-0.002190016	15.45076666	8.47E-05	0.001382886	-0.942926472	-0.157588231	0.471574783	0.154058352	-0.316136847
ENSG00000255397	AC022182.2		-1.28522692	5.557333777	15.4238649	8.59E-05	0.001400274	6.055727669	6.103386224	4.9843626	4.45656751	5.532146371
ENSG00000139970	RTN1	reticulon 1 [Source:HGNC Symbol;Acc:10467]	1.613132252	5.215602797	15.42364006	8.59E-05	0.001400274	3.609842318	4.595461025	5.25876374	6.039879229	5.493399508
ENSG00000185215	TNFAIP2	tumor necrosis factor, alpha-induced protein 2 [Source:HGNC Symbol;Acc:11895]	2.336560572	8.954436169	15.40966515	8.65E-05	0.001409359	6.926769471	7.609315131	8.55848514	10.30838193	9.017148667
ENSG00000249795	RP11-18O11.2		2.185607106	4.425535695	15.40376742	8.68E-05	0.001412454	3.307872948	3.327938933	3.64944369	5.636853273	4.637941118
ENSG00000137634	NXPE4	neurexophilin and PC-esterase domain family, member 4 [Source:HGNC Symbol;Acc:23117]	-8.130746819	-0.197583642	15.40075872	8.70E-05	0.001413395	1.287817526	0.610915408	-7.002070102	-7.002070102	-3.754655114
ENSG00000182118	FAM89A	family with sequence similarity 89, member A [Source:HGNC Symbol;Acc:25057]	-1.723274519	2.589646932	15.39455213	8.72E-05	0.001414117	3.462559037	3.221109942	1.225685365	0.773941511	2.435629945
ENSG00000171860	C3AR1	complement component 3a receptor 1 [Source:HGNC Symbol;Acc:1319]	1.770568708	4.820252781	15.39513131	8.72E-05	0.001414117	3.206417789	3.743725731	4.278711965	5.512129844	5.736979558
ENSG00000236438	FAM157A	family with sequence similarity 157, member A [Source:HGNC Symbol;Acc:34079]	2.424143608	1.97847816	15.39680288	8.71E-05	0.001414117	0.742455703	0.527293245	1.076464519	2.8843467	2.772967998
ENSG00000158578	ALAS2	aminolevulinate, delta-, synthase 2 [Source:HGNC Symbol;Acc:397]	-3.436963638	6.569769039	15.38941099	8.75E-05	0.001415036	7.207151423	7.815235762	5.134833122	-0.244792505	6.037004484
ENSG00000166451	CENPN	centromere protein N [Source:HGNC Symbol;Acc:30873]	-1.726884178	2.788589907	15.38809693	8.75E-05	0.001415036	3.669153174	3.527634524	1.098750223	1.39816257	2.381602043
ENSG00000134955	SLC37A2	solute carrier family 37 (glucose-6-phosphate transporter), member 2 [Source:HGNC Symbol;Acc:20644]	1.714540998	4.709955548	15.39135458	8.74E-05	0.001415036	3.367280913	4.117046668	4.583339883	5.690394499	4.769981094
ENSG00000147536	GINS4	GINS complex subunit 4 (Sld5 homolog) [Source:HGNC Symbol;Acc:28226]	-1.596806599	3.226558188	15.3845761	8.77E-05	0.001416371	3.912537499	3.884797304	2.243421497	1.446384807	3.204982016
ENSG00000204020	LIPN	lipase, family member N [Source:HGNC Symbol;Acc:23452]	2.017888743	2.410202931	15.37595101	8.81E-05	0.001421543	1.210071084	1.773719635	2.540658833	3.399046051	2.064792492
ENSG00000226818	SLC6A6P1	solute carrier family 6, member 6 pseudogene 1 [Source:HGNC Symbol;Acc:11053]	1.761023513	3.07351584	15.34313773	8.96E-05	0.00144382	2.103982592	2.172725723	3.049757403	3.863006047	3.348020491
ENSG00000111181	SLC6A12	solute carrier family 6 (neurotransmitter transporter), member 12 [Source:HGNC Symbol;Acc:11045]	2.332333873	1.651020338	15.34310322	8.96E-05	0.00144382	0.090278201	1.128690501	1.450281721	2.677681489	1.596341512
ENSG00000162733	DDR2	discoidin domain receptor tyrosine kinase 2 [Source:HGNC Symbol;Acc:2731]	-1.970423561	1.713033086	15.33700899	8.99E-05	0.001447157	2.824764954	2.638965838	-0.390491059	0.03296005	-0.045536284
ENSG00000164010	ERMAP	erythroblast membrane-associated protein (Scianna blood group) [Source:HGNC Symbol;Acc:15743]	-1.404391868	5.585282152	15.33309624	9.01E-05	0.001448829	5.944598765	6.439409225	4.379997347	4.890483788	5.353052382
ENSG00000006327	TNFRSF12A	tumor necrosis factor receptor superfamily, member 12A [Source:HGNC Symbol;Acc:18152]	2.615150004	1.530985319	15.32881906	9.03E-05	0.001450784	0.329658892	0.056395802	0.778135316	2.9638037	1.218946104
ENSG00000145623	OSMR	oncostatin M receptor [Source:HGNC Symbol;Acc:8507]	-4.137257509	-0.070577189	15.31676116	9.09E-05	0.001458739	1.18996172	1.050973916	-7.002070102	-4.730697034	-4.610216385
ENSG00000196305	IARS	isoleucyl-tRNA synthetase [Source:HGNC Symbol;Acc:5330]	-1.471037835	6.778363626	15.31031668	9.12E-05	0.001462387	7.458978129	7.351155636	6.385835512	5.440525121	6.362825211
ENSG00000110203	FOLR3	folate receptor 3 (gamma) [Source:HGNC Symbol;Acc:3795]	2.355453067	2.909079159	15.30719915	9.14E-05	0.001463466	1.397104685	1.607225949	2.073793572	4.059250973	3.421261246
ENSG00000013306	SLC25A39	solute carrier family 25, member 39 [Source:HGNC Symbol;Acc:24279]	-1.396837743	6.669922986	15.2957478	9.19E-05	0.001471023	7.211210303	7.351155636	6.295865611	5.63944842	6.107350974

ENSG00000238225	CRIP1P2	cysteine-rich protein 1 (intestinal) pseudogene 2 [Source:HGNC Symbol;Acc:44517]	1.501810555	3.69362554	15.2849237	9.25E-05	0.001478129	3.181172573	2.969801053	3.929837342	4.327332549	3.609304371
ENSG00000165801	ARHGEF40	Rho guanine nucleotide exchange factor (GEF) 40 [Source:HGNC Symbol;Acc:25516]	1.812028296	6.194723463	15.27720241	9.28E-05	0.001481485	4.443058246	5.380695729	5.857581416	7.176217485	6.642874821
ENSG00000261888	AC144831.1		2.543909152	0.923008487	15.27868851	9.28E-05	0.001481485	0.046214424	-0.713501401	0.538319988	2.126858114	0.791775511
ENSG00000159131	GART	phosphoribosylglycinamide formyltransferase, phosphoribosylglycinamide synthetase, phosphoribosylaminoimidazole synthetase [Source:HGNC Symbol;Acc:4163]	-1.293262884	6.141816475	15.24566649	9.44E-05	0.00150506	6.701161162	6.707813094	5.64119881	5.066647213	5.924996229
ENSG00000218537	AP000350.4	Uncharacterized protein [Source:UniProtKB/TrEMBL;Acc:B5MCZ7]	2.264403174	0.0820185	15.23241	9.51E-05	0.001514286	-1.608074432	-0.713501401	0.036330944	0.03296005	1.044483142
ENSG00000145335	SNCA	synuclein, alpha (non A4 component of amyloid precursor) [Source:HGNC Symbol;Acc:11138]	-1.429520845	5.156165529	15.21785816	9.58E-05	0.001524615	5.619524849	5.827089554	3.836777169	4.412741153	5.188416401
ENSG00000092470	WDR76	WD repeat domain 76 [Source:HGNC Symbol;Acc:25773]	-1.471835412	4.172271542	15.19645034	9.69E-05	0.0015406	4.749936823	4.853275919	3.481516588	2.46787168	4.116136211
ENSG00000267174	CTC-510F12.4		2.361519489	1.904913594	15.1873607	9.74E-05	0.001546633	0.365960855	1.03087254	1.285196495	2.924622147	2.3444256
ENSG00000148680	HTR7	5-hydroxytryptamine (serotonin) receptor 7, adenylate cyclase-coupled [Source:HGNC Symbol;Acc:5302]	2.171955738	0.798257607	15.18285993	9.76E-05	0.001548922	-0.489407866	0.277241192	0.538319988	1.27894994	1.3928929
ENSG00000019169	MARCO	macrophage receptor with collagenous structure [Source:HGNC Symbol;Acc:6895]	2.050014361	2.710085666	15.16263182	9.86E-05	0.00156419	1.32517403	2.107040151	2.642776537	3.84212996	2.32547185
ENSG00000259337	IGHV1OR15-2	immunoglobulin heavy variable 1/OR15-2 (pseudogene) [Source:HGNC Symbol;Acc:5564]	-2.504002941	0.774895871	15.15251311	9.92E-05	0.001571176	2.103982592	1.220295226	-3.233942493	-1.572251189	0.264147325
ENSG00000255734	HNRNPABP1	heterogeneous nuclear ribonucleoprotein A/B pseudogene 1 [Source:HGNC Symbol;Acc:48744]	-1.306106789	5.083524189	15.13468506	0.000100107	0.001584106	5.658462756	5.715811808	3.984718105	4.192457932	5.002543123
ENSG00000256660	CLEC12B	C-type lectin domain family 12, member B [Source:HGNC Symbol;Acc:31966]	1.912553809	2.615076425	15.13209005	0.000100245	0.001584106	1.431768352	1.523383318	2.499374267	3.349187152	3.162892704
ENSG00000179542	SLITRK4	SLIT and NTRK-like family, member 4 [Source:HGNC Symbol;Acc:23502]	2.254036809	2.376327185	15.13193448	0.000100253	0.001584106	0.502670228	1.289608822	1.829176068	3.164528843	3.220454233
ENSG00000129538	RNASE1	ribonuclease, RNase A family, 1 (pancreatic) [Source:HGNC Symbol;Acc:10044]	-2.258731834	0.838690749	15.11918679	0.000100932	0.001590543	1.711139862	1.508923295	-2.287883461	-1.449512048	0.973207307
ENSG00000184445	KNTC1	kinetochore associated 1 [Source:HGNC Symbol;Acc:17255]	-1.238413571	5.632806697	15.11986854	0.000100896	0.001590543	6.076414358	6.262693411	5.129430966	4.587767468	5.475241888
ENSG00000254614	AP003068.23	Uncharacterized protein [Source:UniProtKB/TrEMBL;Acc:E9PK70]	1.839838108	3.293419596	15.12253298	0.000100753	0.001590543	2.217603867	2.424598913	3.201624573	4.426830846	2.999117651
ENSG00000188282	RUFY4	RUN and FYVE domain containing 4 [Source:HGNC Symbol;Acc:24804]	2.312033622	1.564576891	15.11731193	0.000101033	0.001590696	0.329658892	1.010487126	1.225685365	2.794059013	0.948644093
ENSG00000141198	TOM1L1	target of myb1 (chicken)-like 1 [Source:HGNC Symbol;Acc:11983]	-2.273387498	1.20258933	15.10940214	0.000101457	0.001595943	2.5449974	1.997369264	-1.886652327	-1.336401299	-0.506781561
ENSG00000248712	CCDC153	coiled-coil domain containing 153 [Source:HGNC Symbol;Acc:27446]	2.232942638	0.68034965	15.10589331	0.000101645	0.001597481	-0.425964666	-0.157588231	0.437007322	1.508271876	0.923655414
ENSG00000232912	RP5-1115A15.1		2.275905589	0.002911798	15.09019978	0.000102494	0.001609376	-0.489407866	-1.394066322	0.471574783	0.074465887	0.303422373
ENSG00000179331	RAB39A	RAB39A, member RAS oncogene family [Source:HGNC Symbol;Acc:16521]	2.202596812	1.149816919	15.07270428	0.000103448	0.00162291	0.174562846	0.555709266	0.934997507	2.02468586	1.155968757
ENSG00000198142	SOWAHC	sosondowah ankyrin repeat domain family member C [Source:HGNC Symbol;Acc:26149]	2.208695184	1.720999959	15.0647806	0.000103884	0.001628282	0.435934254	0.468724906	1.432844522	2.768996501	1.982488568
ENSG00000100095	SEZ6L	seizure related 6 homolog (mouse)-like [Source:HGNC Symbol;Acc:10763]	2.129033418	2.415256745	15.02346093	0.000106183	0.001662838	0.742455703	1.773719635	2.413082378	3.60780319	1.908000984
ENSG00000248702	RP11-294J22.5		1.628075258	3.189880495	15.01499716	0.00010666	0.001668823	2.429680882	2.492318033	3.227138203	3.817895715	3.443406881
ENSG00000269929	RP11-2B6.2		2.009127525	1.826165108	14.99134537	0.000108005	0.001688366	1.287817526	0.881727766	2.213098418	2.749909693	0.84598249
ENSG00000136231	IGF2BP3	insulin-like growth factor 2 mRNA binding protein 3 [Source:HGNC Symbol;Acc:28868]	2.114866853	1.273058679	14.97241732	0.000109094	0.001703869	0.254194054	0.343933181	1.007464306	1.948600359	1.802296017
ENSG00000271335	RP11-324I22.4		2.147350895	0.437349407	14.96184724	0.000109707	0.001711918	-0.856736938	-0.025429826	0.9100098	0.114810932	1.021113618
ENSG00000258476	RP11-76E17.3		2.551033138	0.316581259	14.95961237	0.000109837	0.001712424	-0.425964666	-0.782968247	-0.66302287	1.296595379	0.615745481
ENSG00000235245	RP11-122K13.12		1.772192793	2.148968756	14.95577729	0.000110061	0.001714386	1.343495699	1.749322856	2.709464044	2.743490894	1.463945021
ENSG00000238005	RP11-443B7.1		2.437957212	0.347386057	14.94703969	0.000110571	0.001720817	-0.856736938	-0.713501401	-0.907612816	0.984547783	1.279288931
ENSG00000084110	HAL	histidine ammonia-lyase [Source:HGNC Symbol;Acc:4806]	1.94448169	4.532985989	14.93940969	0.000111019	0.001726259	3.08644716	3.380513419	3.836777169	5.415508302	5.307898957
ENSG00000214283	RP11-85F14.1		-2.114314001	1.474175043	14.93584681	0.000111229	0.001727992	2.341276415	2.007691074	-1.202308962	-0.655391447	1.788521245
ENSG00000089127	OAS1	2'-5'-oligoadenylate synthetase 1, 40/46kDa [Source:HGNC Symbol;Acc:8086]	1.474432763	6.1637703	14.9195001	0.000112197	0.001739949	4.96341172	5.390588949	6.038515166	6.836551042	6.714739006

ENSG00000183023	SLC8A1	solute carrier family 8 (sodium/calcium exchanger), member 1 [Source:HGNC Symbol;Acc:11068]	1.568814612	5.342856525	14.92085957	0.000112116	0.001739949	3.865767927	4.546744448	5.100732931	6.286090018	5.709830413
ENSG00000177602	GSG2	germ cell associated 2 (haspin) [Source:HGNC Symbol;Acc:19682]	-2.024566913	2.027979108	14.91677401	0.000112359	0.001740927	2.818229329	2.825562359	-0.740035668	0.669468625	1.908000984
ENSG00000180155	LYNX1	Ly6/neurotoxin 1 [Source:HGNC Symbol;Acc:29604]	1.797195525	2.882630526	14.90810876	0.000112877	0.0017474	1.752861822	2.424598913	3.201624573	3.895212931	1.908000984
ENSG00000226029	RP4-798A10.2		1.95781068	1.298927049	14.90328533	0.000113166	0.00175033	0.254194054	0.438524285	1.613958159	1.914722448	1.410986673
ENSG00000079931	MOXD1	monooxygenase, DBH-like 1 [Source:HGNC Symbol;Acc:21063]	-2.314482913	1.164226873	14.88920122	0.000114014	0.001761894	2.463579632	1.900991143	-1.315629595	-2.419096061	0.095393329
ENSG00000188290	HES4	hes family bHLH transcription factor 4 [Source:HGNC Symbol;Acc:24149]	2.603309944	0.374283841	14.88425632	0.000114313	0.001764965	-1.237502295	-1.100062965	0.210162819	1.462107232	0.485200289
ENSG00000096006	CRISP3	cysteine-rich secretory protein 3 [Source:HGNC Symbol;Acc:16904]	-2.389407418	2.337146443	14.87592034	0.000114819	0.001771224	3.520070133	2.172725723	0.858697288	-1.572251189	2.786891356
ENSG00000100288	CHKB	choline kinase beta [Source:HGNC Symbol;Acc:1938]	1.996342179	0.805962018	14.87101467	0.000115118	0.001774278	0.329658892	0.095631612	1.205290011	1.148991974	0.706443614
ENSG00000170955	PRKCDBP	protein kinase C, delta binding protein [Source:HGNC Symbol;Acc:9400]	3.11673599	-0.108770573	14.86840931	0.000115277	0.001775172	-1.608074432	-2.262017103	-3.233942493	0.823471408	0.973207307
ENSG00000198838	RYR3	ryanodine receptor 3 [Source:HGNC Symbol;Acc:10485]	-2.359528659	2.223470658	14.84464692	0.000116739	0.001796101	3.241034999	3.091246938	-2.539967122	1.148991974	1.410986673
ENSG00000008438	PGLYRP1	peptidoglycan recognition protein 1 [Source:HGNC Symbol;Acc:8904]	-2.056608915	2.533494837	14.79777066	0.000119677	0.001838082	3.617390898	2.671789775	1.32354911	-0.296766077	2.751827722
ENSG00000166035	LIPC	lipase, hepatic [Source:HGNC Symbol;Acc:6619]	2.095151837	0.233453511	14.79821387	0.000119649	0.001838082	-0.625332215	-0.408932621	0.168628767	0.229488395	0.948644093
ENSG00000100297	MCM5	minichromosome maintenance complex component 5 [Source:HGNC Symbol;Acc:6948]	-1.31364389	6.833425983	14.79573459	0.000119806	0.001838459	7.34652278	7.324825424	6.004245915	6.129987298	6.815389977
ENSG00000137331	IER3	immediate early response 3 [Source:HGNC Symbol;Acc:5392]	2.036975964	4.469885326	14.7901279	0.000120163	0.001842323	3.462559037	2.969801053	3.740844752	5.547563528	4.978478481
ENSG00000042832	TG	thyroglobulin [Source:HGNC Symbol;Acc:11764]	2.276012753	-0.168666175	14.77873977	0.000120891	0.001851866	-0.856736938	-1.507080648	0.168628767	-0.296766077	0.450630967
ENSG00000171793	CTPS1	CTP synthase 1 [Source:HGNC Symbol;Acc:2519]	-1.32230954	4.566585861	14.77550355	0.000121098	0.001853429	5.187866611	5.110749769	3.796441777	3.394956328	4.509480421
ENSG00000167772	ANGPTL4	angiopoietin-like 4 [Source:HGNC Symbol;Acc:16039]	-7.936440861	-0.414608307	14.7326398	0.000123883	0.001894395	1.148882041	0.242702272	-7.002070102	-7.002070102	-4.610216385
ENSG00000100234	TIMP3	TIMP metallopeptidase inhibitor 3 [Source:HGNC Symbol;Acc:11822]	-2.015939542	2.40462596	14.72719817	0.000124241	0.001898217	3.376207305	3.344320706	-1.097244829	1.168289627	1.531661491
ENSG00000243244	STON1	stonin 1 [Source:HGNC Symbol;Acc:17003]	1.934481748	1.110508793	14.72394947	0.000124455	0.001899837	0.435934254	0.498306264	1.450281721	1.538247531	1.090116303
ENSG00000196562	SULF2	sulfatase 2 [Source:HGNC Symbol;Acc:20392]	1.645263585	7.282379359	14.70911589	0.000125438	0.001913181	5.515299326	6.563101292	7.384351513	8.298898396	7.283337198
ENSG00000254995	STX16-NPEPL1	STX16-NPEPL1 readthrough (NMD candidate) [Source:HGNC Symbol;Acc:41993]	1.52379355	3.374119987	14.7041087	0.000125772	0.001916604	2.446729819	3.056868784	4.017256742	4.02790164	2.399837082
ENSG00000213934	HBG1	hemoglobin, gamma A [Source:HGNC Symbol;Acc:4831]	-3.386428467	4.542722798	14.68469535	0.000127074	0.001933326	5.754014405	5.697597441	3.232187228	-3.360122781	-0.257803757
ENSG00000171777	RASGRP4	RAS guanyl releasing protein 4 [Source:HGNC Symbol;Acc:18958]	1.652755649	6.637436279	14.68446345	0.00012709	0.001933326	5.142808642	5.762272646	6.308469644	7.559994571	7.1286112
ENSG00000101425	BPI	bactericidal/permeability-increasing protein [Source:HGNC Symbol;Acc:1095]	-1.478363626	4.991026761	14.67783588	0.000127537	0.001936375	5.728100811	5.110749769	3.697694186	4.02790164	5.388869293
ENSG00000062822	POLD1	polymerase (DNA directed), delta 1, catalytic subunit [Source:HGNC Symbol;Acc:9175]	-1.263685319	5.311463468	14.67659836	0.000127621	0.001936375	5.820107035	5.910592651	4.686264737	4.149407864	5.252533318
ENSG00000134830	C5AR2	complement component 5a receptor 2 [Source:HGNC Symbol;Acc:4527]	1.697402925	3.905464904	14.67762058	0.000127552	0.001936375	2.831271105	2.932364153	3.708603425	4.770917032	4.287520171
ENSG00000237632	S100A11P1	S100 calcium binding protein A11 pseudogene 1 [Source:HGNC Symbol;Acc:10491]	1.810556286	3.11654199	14.66215027	0.000128603	0.00194959	1.806678074	2.564011614	3.11133962	3.827031329	3.439004866
ENSG00000260898	ADPGK-AS1	ADPGK antisense RNA 1 [Source:HGNC Symbol;Acc:44144]	2.934918842	0.590995282	14.64749966	0.000129606	0.001963107	-0.250832646	-1.014020025	-1.202308962	2.076676283	0.303422373
ENSG00000165244	ZNF367	zinc finger protein 367 [Source:HGNC Symbol;Acc:18320]	-1.479195661	3.568955356	14.64547879	0.000129746	0.001963519	4.165183448	4.268508089	2.36792042	2.240618578	3.577616005
ENSG00000117877	CD3EAP	CD3e molecule, epsilon associated protein [Source:HGNC Symbol;Acc:24219]	-1.654060985	2.689122621	14.63974666	0.000130141	0.001966341	3.511993298	3.392378661	1.746900214	1.048430793	2.206202022
ENSG00000128944	KNSTRN	kinetochore-localized astrin/SPAG5 binding protein [Source:HGNC Symbol;Acc:30767]	-1.516292933	3.824960734	14.63952567	0.000130156	0.001966341	4.599886656	4.353915352	2.556847448	2.824787032	3.689318974
ENSG00000248098	BCKDHA	branched chain keto acid dehydrogenase E1, alpha polypeptide [Source:HGNC Symbol;Acc:986]	2.040306535	0.170329476	14.60781673	0.000132364	0.001996268	-0.196880952	-0.30308518	0.28980717	0.434658742	0.139468177

ENSG00000244607	CCDC13	coiled-coil domain containing 13 [Source:HGNC Symbol;Acc:26358]	2.118808504	0.396937173	14.60829354	0.000132331	0.001996268	-0.365194284	0.095631612	0.081791624	1.109604583	0.303422373
ENSG00000148737	TCF7L2	transcription factor 7-like 2 (T-cell specific, HMG-box) [Source:HGNC Symbol;Acc:11641]	1.621656503	4.313713002	14.59993072	0.000132919	0.002002919	3.002462395	3.818918804	4.39583296	5.394153653	3.796436594
ENSG00000185710	RP11-645C24.2		2.130561932	0.082928063	14.58977716	0.000133638	0.002012013	-0.14487434	-0.464918547	0.250534523	0.642123966	-0.649489103
ENSG00000158402	CDC25C	cell division cycle 25C [Source:HGNC Symbol;Acc:1727]	-2.171386459	1.417487004	14.56520345	0.000135392	0.002035165	2.313663059	2.107040151	-2.073364206	-1.336401299	1.658245749
ENSG00000152672	CLEC4F	C-type lectin domain family 4, member F [Source:HGNC Symbol;Acc:25357]	2.468405655	1.706701612	14.56498949	0.000135407	0.002035165	-0.698419327	1.09035546	2.096120255	2.543716201	1.673315497
ENSG00000204054	LINC00963	long intergenic non-protein coding RNA 963 [Source:HGNC Symbol;Acc:48716]	1.422327481	5.651398296	14.56257718	0.000135581	0.002036029	4.493153696	4.98970302	5.546078521	6.336006582	6.113579235
ENSG00000138092	CENPO	centromere protein O [Source:HGNC Symbol;Acc:28152]	-1.391600525	4.159134349	14.53504198	0.000137577	0.002062474	4.810461175	4.776883982	3.133097051	3.059925941	4.039700715
ENSG00000133216	EPHB2	EPH receptor B2 [Source:HGNC Symbol;Acc:3393]	2.064132508	1.759727429	14.53524317	0.000137562	0.002062474	0.435934254	0.836121102	1.245796404	2.50629229	2.453198788
ENSG00000225774	SIRPAP1	signal-regulatory protein alpha pseudogene 1 [Source:HGNC Symbol;Acc:9663]	1.554794748	4.943378415	14.51578862	0.00013899	0.002081882	3.524091657	4.235953559	4.717204047	5.600910297	5.596805137
ENSG00000225169	BRI3P1	brain protein I3 pseudogene 1 [Source:HGNC Symbol;Acc:33533]	1.768468887	3.405922645	14.50973313	0.000139437	0.002086804	2.32292636	2.753793145	3.216986831	4.19716293	3.736716106
ENSG00000143158	MPC2	mitochondrial pyruvate carrier 2 [Source:HGNC Symbol;Acc:24515]	-1.284083491	5.548822354	14.50200362	0.000140011	0.002093599	6.301030756	6.005064844	4.731537276	4.7550502	5.214406097
ENSG00000144407	PTH2R	parathyroid hormone 2 receptor [Source:HGNC Symbol;Acc:9609]	-2.678647847	0.561463722	14.48130163	0.000141558	0.002113134	1.498692903	1.147482679	-4.621297774	-2.419096061	0.872340852
ENSG00000205913	SRRM2-AS1	SRRM2 antisense RNA 1 [Source:HGNC Symbol;Acc:44162]	2.116283736	-0.109684564	14.48148463	0.000141544	0.002113134	-0.365194284	-0.782968247	0.168628767	0.074465887	-0.257803757
ENSG00000107242	PIP5K1B	phosphatidylinositol-4-phosphate 5-kinase, type I, beta [Source:HGNC Symbol;Acc:8995]	-1.777685552	4.011202722	14.45225516	0.000143758	0.00214415	4.755078638	4.680176667	1.907011864	3.301918251	3.813541063
ENSG00000233038	AC011899.9		2.141314117	3.508857958	14.44760586	0.000144113	0.002147625	1.593595892	2.618906922	2.979436032	4.596697704	3.943532595
ENSG00000264456	RP11-848P1.2		2.24305235	1.400116	14.44045406	0.000144661	0.002153967	0.090278201	0.527293245	1.225685365	2.460063232	1.35601001
ENSG00000147459	DOCK5	dedicator of cytokinesis 5 [Source:HGNC Symbol;Acc:23476]	1.766944157	6.401090915	14.41740058	0.000146443	0.002177515	4.924386901	5.233457778	5.80178958	7.527906484	6.832272507
ENSG00000179299	NSUN7	NOP2/Sun domain family, member 7 [Source:HGNC Symbol;Acc:25857]	2.732542291	0.866742923	14.41678498	0.000146491	0.002177515	0.046214424	-1.100062965	-0.66302287	1.959718487	1.498200525
ENSG00000268942	CKS1B	CDC28 protein kinase regulatory subunit 1B, isoform CRA_b; cDNA, FLJ92030, Homo sapiens CDC28 protein kinase regulatory subunit 1B (CKS1B),mRNA [Source:UniProtKB/TrEMBL;Acc:Q5T178]	-1.823761375	2.042727476	14.41485941	0.000146641	0.002177899	3.047861816	2.598565174	0.663204908	0.114810932	1.732068029
ENSG00000238039	AF011889.2		2.354095237	-0.169259645	14.40421157	0.000147472	0.002188397	-1.921450438	-0.713501401	0.365283811	-0.874832399	0.584199832
ENSG00000167207	NOD2	nucleotide-binding oligomerization domain containing 2 [Source:HGNC Symbol;Acc:5331]	1.54280107	5.577497396	14.39271115	0.000148376	0.002199943	4.27425947	4.979251238	5.438791925	6.44237844	5.839068896
ENSG00000163507	KIAA1524	KIAA1524 [Source:HGNC Symbol;Acc:29302]	-1.530675337	3.805556564	14.38412874	0.000149053	0.002208128	4.432399102	4.321184566	2.474024282	2.580193821	4.045503588
ENSG00000143595	AQP10	aquaporin 10 [Source:HGNC Symbol;Acc:16029]	1.839548442	1.924168185	14.37108121	0.00015009	0.002221607	0.597320328	1.184348258	2.404162378	2.412303653	2.109797605
ENSG00000198814	GK	glycerol kinase [Source:HGNC Symbol;Acc:4289]	1.968955085	3.3168978	14.36629422	0.000150472	0.002225386	1.752861822	2.116608898	2.572856422	4.017298632	4.302136394
ENSG00000175899	A2M	alpha-2-macroglobulin [Source:HGNC Symbol;Acc:7]	-1.961243389	1.481463094	14.36064187	0.000150924	0.002226449	2.512979527	2.439924693	-0.520331194	-0.874832399	0.341656497
ENSG00000124207	CSE1L	CSE1 chromosome segregation 1-like (yeast) [Source:HGNC Symbol;Acc:2431]	-1.275428747	6.241598673	14.36242799	0.000150781	0.002226449	6.773045739	6.810871874	5.79583413	5.135673164	6.03773248
ENSG00000048342	CC2D2A	coiled-coil and C2 domain containing 2A [Source:HGNC Symbol;Acc:29253]	2.11703961	0.448158589	14.3611759	0.000150881	0.002226449	-0.856736938	0.056395802	0.28980717	0.74852395	1.044483142
ENSG00000255200	AP003068.18		2.262222242	0.637086114	14.34910751	0.000151852	0.00223825	-0.14487434	-0.355038393	0.632982468	1.79568083	-0.376928291
ENSG00000181444	ZNF467	zinc finger protein 467 [Source:HGNC Symbol;Acc:23154]	1.608221466	5.635050352	14.33833653	0.000152723	0.002249203	4.041581121	4.89279376	5.386536308	6.475193297	6.154427687
ENSG00000139835	GRTP1	growth hormone regulated TBC protein 1 [Source:HGNC Symbol;Acc:20310]	-1.852298768	1.87036752	14.32903832	0.000153479	0.002258445	2.811663962	2.778115078	-0.10933503	-0.053815412	1.134350211
ENSG00000140836	ZFHX3	zinc finger homeobox 3 [Source:HGNC Symbol;Acc:777]	1.71883001	4.375366581	14.31744093	0.000154427	0.002270498	2.723478589	3.570041503	4.028909353	5.380773935	4.755892772
ENSG00000136869	TLR4	toll-like receptor 4 [Source:HGNC Symbol;Acc:11850]	1.762052208	7.259581802	14.29973406	0.000155887	0.002290037	5.372051314	6.230556812	6.897760548	8.024444566	8.075674702
ENSG00000073150	PANX2	pannexin 2 [Source:HGNC Symbol;Acc:8600]	2.408143777	1.324183819	14.2890722	0.000156772	0.002301119	0.090278201	0.056395802	0.437007322	2.404187718	1.802296017

ENSG00000150681	RGS18	regulator of G-protein signaling 18 [Source:HGNC Symbol;Acc:14261]	1.656981662	6.583506525	14.28708103	0.000156938	0.00230163	5.112413948	5.715811808	6.315028071	7.426372905	7.134067905
ENSG00000157551	KCNJ15	potassium inwardly-rectifying channel, subfamily J, member 15 [Source:HGNC Symbol;Acc:6261]	3.181926372	4.220822452	14.28410907	0.000157186	0.002303343	2.177314774	1.09035546	1.703933103	5.524350082	5.210537365
ENSG00000187244	BCAM	basal cell adhesion molecule (Lutheran blood group) [Source:HGNC Symbol;Acc:6722]	-2.288628264	1.757273938	14.25350371	0.000159763	0.00233915	2.313663059	2.948527637	0.036330944	-4.730697034	1.498200525
ENSG00000254419	RP11-261P9.4		1.843265358	1.662744739	14.24902481	0.000160144	0.00234277	0.714573228	1.272590116	2.273120332	2.22227058	0.997359302
ENSG00000123416	TUBA1B	tubulin, alpha 1b [Source:HGNC Symbol;Acc:18809]	-1.608864586	8.559140229	14.24658138	0.000160352	0.00234386	9.185277518	9.110369539	7.317335015	7.838761823	8.501252674
ENSG00000164825	DEFB1	defensin, beta 1 [Source:HGNC Symbol;Acc:2766]	-3.404990837	-0.00055943	14.23993848	0.000160919	0.002350191	0.823008709	1.03087254	-7.002070102	-3.888428289	-0.316136847
ENSG00000135407	AVIL	advillin [Source:HGNC Symbol;Acc:14188]	1.750079094	2.189781106	14.23462107	0.000161374	0.002354882	1.18996172	1.523383318	2.482523865	2.756300061	2.334979851
ENSG00000071246	VASH1	vasohibin 1 [Source:HGNC Symbol;Acc:19964]	1.288784537	5.917151958	14.23143189	0.000161648	0.002356917	4.777150473	5.374727068	6.08181195	6.373381992	6.363406068
ENSG00000225439	BOLA3-AS1	BOLA3 antisense RNA 1 (head to head) [Source:HGNC Symbol;Acc:42922]	-1.775606452	2.066295261	14.22805298	0.000161938	0.002359193	2.949619895	2.937772127	0.538319988	0.696304622	1.11240278
ENSG00000124635	HIST1H2BJ	histone cluster 1, H2bj [Source:HGNC Symbol;Acc:4761]	2.192538169	1.594930577	14.22415275	0.000162274	0.002362127	0.534915169	0.376157587	1.342349735	2.636704962	1.732068029
ENSG00000116990	MYCL	v-myc avian myelocytomatosis viral oncogene lung carcinoma derived homolog [Source:HGNC Symbol;Acc:7555]	1.535420303	4.986534894	14.21958917	0.000162668	0.0023659	3.265262705	4.314964006	5.072860299	5.5149591	5.625175993
ENSG00000089041	P2RX7	purinergic receptor P2X, ligand-gated ion channel, 7 [Source:HGNC Symbol;Acc:8537]	1.562012786	4.942636551	14.2100741	0.000163493	0.002375925	3.78763224	4.287693936	4.752773603	5.841541259	5.163284825
ENSG00000139618	BRCA2	breast cancer 2, early onset [Source:HGNC Symbol;Acc:1101]	-1.485498168	3.572429565	14.19920236	0.00016444	0.002387715	3.85941584	4.407518463	2.404162378	2.066427005	3.803302735
ENSG00000087245	MMP2	matrix metallopeptidase 2 (gelatinase A, 72kDa gelatinase, 72kDa type IV collagenase) [Source:HGNC Symbol;Acc:7166]	-3.277355605	0.815224896	14.19316846	0.000164968	0.002391428	2.038016118	1.109650305	-4.621297774	-3.888428289	1.11240278
ENSG00000208028	MIR616	microRNA 616 [Source:HGNC Symbol;Acc:32872]	3.008739182	0.786738283	14.19421092	0.000164877	0.002391428	-0.942926472	-1.289265519	-1.097244829	2.536308679	0.139468177
ENSG00000175643	RMI2	RecQ mediated genome instability 2 [Source:HGNC Symbol;Acc:28349]	-1.679761175	2.391246995	14.14881449	0.000168904	0.002444436	3.02534068	3.100920657	0.632982468	0.642123966	2.610226292
ENSG00000055732	MCOLN3	mucolipin 3 [Source:HGNC Symbol;Acc:13358]	2.478780429	0.420076082	14.15012969	0.000168785	0.002444436	-1.608074432	0.376157587	1.815785154	0.265774258	-3.754655114
ENSG00000088305	DNMT3B	DNA (cytosine-5-)-methyltransferase 3 beta [Source:HGNC Symbol;Acc:2979]	-1.713645358	2.610034772	14.13465885	0.000170179	0.00245899	3.547988195	3.152996839	0.832340008	1.109604583	2.513057684
ENSG00000103313	MEFV	Mediterranean fever [Source:HGNC Symbol;Acc:6998]	1.592180626	6.26911443	14.1345476	0.000170189	0.00245899	4.661426622	5.564094797	6.102638058	7.094408075	6.746257499
ENSG00000184221	OLIG1	oligodendrocyte transcription factor 1 [Source:HGNC Symbol;Acc:16983]	1.995894571	3.343417191	14.11764061	0.000171726	0.002477112	1.448792784	2.837184307	3.434103115	4.430831327	3.03456135
ENSG00000265666	CTD-2267D19.2		2.176881391	1.270726716	14.11771547	0.000171719	0.002477112	0.046214424	0.095631612	0.959559782	2.146452098	1.732068029
ENSG00000163435	ELF3	E74-like factor 3 (ets domain transcription factor, epithelial-specific) [Source:HGNC Symbol;Acc:3318]	2.447239032	0.915275299	14.11308958	0.000172142	0.002481074	-0.425964666	0.277241192	0.168628767	2.213008328	0.485200289
ENSG00000106952	TNFSF8	tumor necrosis factor (ligand) superfamily, member 8 [Source:HGNC Symbol;Acc:11938]	1.284067119	5.439840294	14.11120295	0.000172314	0.002481526	4.537594837	5.183117208	6.044985507	5.889567528	4.995065952
ENSG00000139946	PELI2	pellino E3 ubiquitin protein ligase family member 2 [Source:HGNC Symbol;Acc:8828]	1.434758228	5.312193314	14.10494666	0.000172889	0.002487752	4.180552023	4.605689658	5.224970667	6.097639375	5.644422022
ENSG00000025708	TYMP	thymidine phosphorylase [Source:HGNC Symbol;Acc:3148]	1.79417547	7.683265806	14.08824535	0.00017443	0.002507884	5.924201569	6.615080104	7.443503286	8.696863537	8.127374541
ENSG00000095637	SORBS1	sorbin and SH3 domain containing 1 [Source:HGNC Symbol;Acc:14565]	-1.739834001	2.105725903	14.08369436	0.000174853	0.002511903	2.961531071	2.985552753	0.632982468	0.74852395	1.177268129
ENSG00000141338	ABCA8	ATP-binding cassette, sub-family A (ABC1), member 8 [Source:HGNC Symbol;Acc:38]	-3.992886802	-0.069772199	14.06671865	0.000176438	0.002530555	0.899300886	1.306429105	-7.002070102	-4.730697034	-4.610216385
ENSG00000125618	PAX8	paired box 8 [Source:HGNC Symbol;Acc:8622]	2.317363595	0.645289533	14.06670407	0.00017644	0.002530555	-0.094677427	-0.204475541	-0.589913612	1.508271876	1.155968757
ENSG00000142405	NLRP12	NLR family, pyrin domain containing 12 [Source:HGNC Symbol;Acc:22938]	1.540919007	5.438111213	14.05803033	0.000177255	0.002536037	4.01610833	4.784407659	5.271081512	6.164631713	5.963817525
ENSG00000171368	TPPP	tubulin polymerization promoting protein [Source:HGNC Symbol;Acc:24164]	1.848539678	0.966754178	14.06051586	0.000177021	0.002536037	0.502670228	0.637745977	1.534439871	1.261086007	0.303422373
ENSG00000225331	AP001055.6		2.200564289	0.43495637	14.05914474	0.00017715	0.002536037	-0.489407866	-0.855949929	0.250534523	0.894716898	1.134350211
ENSG00000107623	GDF10	growth differentiation factor 10 [Source:HGNC Symbol;Acc:4215]	-3.35951982	0.153155819	14.03113462	0.000179809	0.002570473	1.414540624	1.255368251	-4.621297774	-4.730697034	-3.221241555

ENSG00000108960	MMD	monocyte to macrophage differentiation-associated [Source:HGNC Symbol;Acc:7153]	1.48048425	5.499070948	14.02085215	0.000180795	0.002582464	4.631916728	4.840340371	5.438791925	6.355078486	5.559778477
ENSG00000165555	NOXRED1	NADP-dependent oxidoreductase domain containing 1 [Source:HGNC Symbol;Acc:20487]	2.095968262	0.128362661	14.01928814	0.000180945	0.002582511	-0.306880589	-0.464918547	-0.010608983	0.58582888	0.223773082
ENSG00000134809	TIMM10	translocase of inner mitochondrial membrane 10 homolog (yeast) [Source:HGNC Symbol;Acc:11814]	-1.440012601	4.123006886	14.01630683	0.000181232	0.002584508	5.011469904	4.544973774	3.420897078	2.98027543	3.659358136
ENSG00000101916	TLR8	toll-like receptor 8 [Source:HGNC Symbol;Acc:15632]	1.635507528	6.418834516	14.00651281	0.000182179	0.002595896	4.535604208	5.588367519	6.133998707	6.956912787	7.337850514
ENSG00000214402	LCNL1	lipocalin-like 1 [Source:HGNC Symbol;Acc:34436]	2.133972647	0.307312521	13.99027135	0.000183759	0.002616293	-0.856736938	-0.157588231	0.505333341	1.069111613	-0.095747566
ENSG00000118263	KLF7	Kruppel-like factor 7 (ubiquitous) [Source:HGNC Symbol;Acc:6350]	1.223673808	5.926929616	13.96750849	0.000185997	0.002646015	4.998521088	5.484112873	6.259853396	6.46303607	5.962284565
ENSG00000139132	FGD4	FYVE, RhoGEF and PH domain containing 4 [Source:HGNC Symbol;Acc:19125]	1.620076468	4.881721345	13.94096554	0.000188642	0.002681467	3.547988195	4.107480834	4.575415554	5.862425804	5.176566525
ENSG00000124491	F13A1	coagulation factor XIII, A1 polypeptide [Source:HGNC Symbol;Acc:3531]	2.095090937	8.0232118	13.93563345	0.000189178	0.002686908	5.116414977	6.489060932	7.149681355	8.729810333	9.251393779
ENSG00000237840	FAM21FP	family with sequence similarity 21, member F, pseudogene [Source:HGNC Symbol;Acc:45011]	2.095664197	-0.02905503	13.92560719	0.00019019	0.002699094	-0.942926472	-0.30308518	-0.161352853	0.265774258	0.264147325
ENSG00000167608	TMC4	transmembrane channel-like 4 [Source:HGNC Symbol;Acc:22998]	2.100655043	1.052011946	13.91948006	0.000190811	0.002705719	0.502670228	0.242702272	0.983710868	2.035234912	0.485200289
ENSG00000169403	PTAFR	platelet-activating factor receptor [Source:HGNC Symbol;Acc:9582]	1.687943255	7.107589568	13.89870413	0.000192931	0.002733585	5.445982527	6.278230767	6.794453369	8.125553892	7.473976735
ENSG00000184060	ADAP2	ArfGAP with dual PH domains 2 [Source:HGNC Symbol;Acc:16487]	1.642790525	4.79807329	13.89055573	0.00019377	0.002743248	3.64350417	4.018408312	4.444499733	5.78718771	5.049500665
ENSG00000134508	CABLES1	Cdk5 and Abl enzyme substrate 1 [Source:HGNC Symbol;Acc:25097]	-2.009803295	2.604682318	13.8517021	0.000197817	0.002796043	3.590796241	3.509590321	-1.097244829	1.610570062	1.643016926
ENSG00000142583	SLC2A5	solute carrier family 2 (facilitated glucose/fructose transporter), member 5 [Source:HGNC Symbol;Acc:11010]	-1.69856462	3.007122547	13.85232492	0.000197752	0.002796043	3.53210123	2.916017525	1.285196495	0.497040107	4.088296742
ENSG00000140450	ARRDC4	arrestin domain containing 4 [Source:HGNC Symbol;Acc:28087]	1.49729236	4.902466016	13.83869444	0.000199192	0.0028132	4.154846016	4.224936733	4.83638327	5.784845369	4.859994575
ENSG00000132669	RIN2	Ras and Rab interactor 2 [Source:HGNC Symbol;Acc:18750]	1.626067051	4.045719621	13.83165935	0.000199939	0.002821483	2.576320097	3.138979644	3.708603425	4.694777208	4.836757869
ENSG00000120318	ARAP3	ArfGAP with RhoGAP domain, ankyrin repeat and PH domain 3 [Source:HGNC Symbol;Acc:24097]	1.400972929	5.19052664	13.8157214	0.000201642	0.002843232	4.269476339	4.436459301	5.058719411	5.864645607	5.637715078
ENSG00000088726	TMEM40	transmembrane protein 40 [Source:HGNC Symbol;Acc:25620]	2.229759725	2.532292911	13.80497077	0.000202799	0.002854961	0.534915169	1.955327308	2.36792042	3.600718313	2.586540774
ENSG00000238160	AC116366.5		2.286727683	0.219750193	13.80622035	0.000202664	0.002854961	-0.555769858	-1.394066322	-0.271377577	0.669468625	1.06748014
ENSG00000072401	UBE2D1	ubiquitin-conjugating enzyme E2D 1 [Source:HGNC Symbol;Acc:12474]	1.596099673	5.121398021	13.79134391	0.000204275	0.002873439	3.884658084	4.385920009	4.796036708	5.862425804	5.704338732
ENSG00000104974	LILRA1	leukocyte immunoglobulin-like receptor, subfamily A (with TM domain), member 1 [Source:HGNC Symbol;Acc:6602]	1.685799606	6.840126806	13.78857171	0.000204577	0.002875378	5.064896475	6.080955172	6.723172814	7.899744678	6.988037245
ENSG00000105974	CAV1	caveolin 1, caveolae protein, 22kDa [Source:HGNC Symbol;Acc:1527]	-2.423150175	0.937941012	13.78397904	0.000205078	0.00288011	2.015339542	1.565913615	-2.845623393	-2.670141381	0.997359302
ENSG00000251440	STMN1P2	stathmin 1 pseudogene 2 [Source:HGNC Symbol;Acc:44063]	-1.914610066	1.571952154	13.77634843	0.000205912	0.002889521	2.5449974	2.068117593	-0.10933503	-0.797916568	1.612068369
ENSG00000053372	MRTO4	mRNA turnover 4 homolog (S. cerevisiae) [Source:HGNC Symbol;Acc:18477]	-1.354793736	4.112453279	13.76484251	0.000207177	0.002904951	4.855840655	4.686615325	3.468739403	3.007317163	3.628761873
ENSG00000076043	REXO2	RNA exonuclease 2 [Source:HGNC Symbol;Acc:17851]	-1.363426868	5.012323505	13.75660797	0.000208088	0.002913059	5.831510229	5.55971708	4.682580808	3.642714894	4.232623634
ENSG00000232063	RP11-307E17.8		2.443519152	-0.100243522	13.75679174	0.000208067	0.002913059	-0.775407551	-1.394066322	-1.721358966	0.58582888	0.706443614
ENSG00000091136	LAMB1	laminin, beta 1 [Source:HGNC Symbol;Acc:6486]	-3.370312235	0.147393701	13.73936941	0.000210006	0.002937574	1.448792784	1.184348258	-4.621297774	-4.730697034	-2.832686105
ENSG00000173110	HSPA6	heat shock 70kDa protein 6 (HSP70B') [Source:HGNC Symbol;Acc:5239]	1.928755475	4.759711558	13.72132953	0.000212033	0.002963562	3.402659809	3.026735387	3.645664403	5.598244827	5.794968012
ENSG00000108813	DLX4	distal-less homeobox 4 [Source:HGNC Symbol;Acc:2917]	2.194440255	0.402484096	13.71893151	0.000212304	0.002964988	-0.856736938	0.056395802	0.471574783	1.224680533	0.002986071
ENSG00000163751	CPA3	carboxypeptidase A3 (mast cell) [Source:HGNC Symbol;Acc:2298]	-2.477132391	3.717653498	13.70038148	0.000214411	0.002989662	5.124383901	3.368549783	0.081791624	2.086853261	3.908936602
ENSG00000100985	MMP9	matrix metallopeptidase 9 (gelatinase B, 92kDa gelatinase, 92kDa type IV collagenase) [Source:HGNC Symbol;Acc:7176]	-1.462287709	3.99049932	13.70166248	0.000214265	0.002989662	4.597980204	4.1453694	3.026695712	2.320413773	4.595060667

ENSG00000164283	ESM1	endothelial cell-specific molecule 1 [Source:HGNC Symbol;Acc:3466]	-2.013583765	1.347626996	13.6974966	0.000214741	0.002991881	2.521050858	2.329059307	-0.329705368	-1.70641174	-0.894110176
ENSG00000129951	LPPR3	hsa-mir-3187 [Source:miRBase;Acc:MI0014231]	-2.572303518	1.29123135	13.69323398	0.000215229	0.002994507	1.431768352	1.565913615	-3.766908349	-2.97427061	2.618035833
ENSG00000004897	CDC27	cell division cycle 27 [Source:HGNC Symbol;Acc:1728]	-1.167717728	5.849285033	13.69287045	0.00021527	0.002994507	6.273225961	6.485834221	5.196838033	5.087070373	5.652999673
ENSG00000112303	VNN2	vanin 2 [Source:HGNC Symbol;Acc:12706]	1.406920882	6.758020245	13.6727694	0.000217587	0.003024336	5.74111579	6.112381464	6.819829477	7.584667222	6.823855937
ENSG00000111961	SASH1	SAM and SH3 domain containing 1 [Source:HGNC Symbol;Acc:19182]	1.604100775	3.736985971	13.63634666	0.000221849	0.003078699	2.32292636	3.105733301	3.768911065	4.544115616	3.983377119
ENSG00000131669	NINJ1	ninjurin 1 [Source:HGNC Symbol;Acc:7824]	1.674096628	5.9735714	13.63738432	0.000221726	0.003078699	4.626315788	5.247679482	5.635493586	7.104148066	5.997885367
ENSG00000161647	MPP3	membrane protein, palmitoylated 3 (MAGUK p55 subfamily member 3) [Source:HGNC Symbol;Acc:7221]	2.280761339	-0.335426867	13.62871552	0.000222752	0.003088796	-1.608074432	-0.713501401	-1.315629595	0.114810932	0.341656497
ENSG00000110446	SLC15A3	solute carrier family 15 (oligopeptide transporter), member 3 [Source:HGNC Symbol;Acc:18068]	1.618138953	6.084385466	13.61669095	0.000224183	0.003106189	4.813750332	5.355661735	5.782989063	7.156724394	6.173101579
ENSG00000099785	Mar-02	membrane-associated ring finger (C3HC4) 2, E3 ubiquitin protein ligase [Source:HGNC Symbol;Acc:28038]	1.39577187	5.419663679	13.61144688	0.000224811	0.003109968	4.410841602	4.758665442	5.331140544	6.144066896	5.766265118
ENSG00000225889	AC074289.1		2.311789522	4.363434318	13.61254766	0.000224679	0.003109968	3.118718315	2.910527228	3.329625449	5.856489577	4.201775615
ENSG00000236592	S100A11P2	S100 calcium binding protein A11 pseudogene 2 [Source:HGNC Symbol;Acc:37840]	1.678370904	3.005852781	13.60604798	0.000225458	0.003116469	1.883815937	2.521424373	3.026695712	3.642714894	3.295420086
ENSG00000159055	MIS18A	MIS18 kinetochore protein A [Source:HGNC Symbol;Acc:1286]	-1.43342926	3.390558109	13.58883155	0.000227535	0.003142707	4.096626906	3.947798472	2.439516168	2.02468586	3.338598326
ENSG00000185291	IL3RA	interleukin 3 receptor, alpha (low affinity) [Source:HGNC Symbol;Acc:6012]	1.936831375	2.11567819	13.56669757	0.000230234	0.003177484	0.365960855	0.968831255	0.959559782	2.045707388	3.601447293
ENSG00000141744	PNMT	phenylethanolamine N-methyltransferase [Source:HGNC Symbol;Acc:9160]	-2.293209327	0.434698284	13.53403534	0.000234276	0.003230724	1.63880454	1.272590116	-2.845623393	-1.854339469	-0.9858258
ENSG00000226380	MIR29A	microRNA 29a [Source:HGNC Symbol;Acc:31616]	2.334174497	0.724707265	13.51295944	0.000236922	0.003264646	0.329658892	-0.713501401	0.401591237	1.692827451	0.646616095
ENSG00000133063	CHIT1	chitinase 1 (chitotriosidase) [Source:HGNC Symbol;Acc:1936]	-2.568230134	3.640774064	13.49726513	0.000238911	0.003286904	4.397750769	2.217877931	-1.438615935	0.984547783	5.092177156
ENSG00000126262	FFAR2	free fatty acid receptor 2 [Source:HGNC Symbol;Acc:4501]	3.065476661	4.873277006	13.49856961	0.000238745	0.003286904	2.778381342	1.128690501	2.28288562	5.950051493	6.140939638
ENSG00000188739	RBM34	RNA binding motif protein 34 [Source:HGNC Symbol;Acc:28965]	1.947715692	0.784746361	13.48770929	0.000240131	0.003299762	0.254194054	0.468724906	0.983710868	1.582074621	-0.376928291
ENSG00000176681	LRRC37A	leucine rich repeat containing 37A [Source:HGNC Symbol;Acc:29069]	2.095487323	0.109633573	13.48700038	0.000240222	0.003299762	-0.489407866	-0.647226338	-0.520331194	0.369457657	0.84598249
ENSG00000187951	ARHGAP11B	Rho GTPase activating protein 11B [Source:HGNC Symbol;Acc:15782]	-1.394269686	3.798107616	13.47815273	0.000241357	0.003312766	4.438804034	4.314964006	2.860570679	2.558418111	3.860382081
ENSG00000267736	HMGB2P1	high mobility group box 2 pseudogene 1 [Source:HGNC Symbol;Acc:39174]	-1.20922072	5.436584415	13.46691133	0.000242808	0.00333007	5.972825997	6.047579027	4.726178989	4.225075169	5.39683538
ENSG00000256616	RP11-815J4.6		-2.016695153	0.919570839	13.46089779	0.000243587	0.003338152	1.752861822	1.508923295	-1.315629595	-1.336401299	1.11240278
ENSG00000133119	RFC3	replication factor C (activator 1) 3, 38kDa [Source:HGNC Symbol;Acc:9971]	-1.451618031	3.717688577	13.45694362	0.000244101	0.00333998	4.425965607	4.222723237	2.474024282	2.657338701	3.750992014
ENSG00000122497	NBPF14	neuroblastoma breakpoint family, member 14 [Source:HGNC Symbol;Acc:25232]	1.291862485	5.510450446	13.45785395	0.000243983	0.00333998	4.756788512	5.259604691	5.871400925	6.340798246	4.503168814
ENSG00000196668	LINC00173	long intergenic non-protein coding RNA 173 [Source:HGNC Symbol;Acc:33791]	2.28963693	1.593677122	13.44038131	0.000246266	0.003366972	0.133035942	0.610915408	1.098750223	2.860817063	1.463945021
ENSG00000268279	RP11-434D12.1		1.832907618	0.99639209	13.43401522	0.000247103	0.003375788	0.534915169	0.438524285	1.484536312	1.582074621	0.139468177
ENSG00000221829	FANCG	Fanconi anemia, complementation group G [Source:HGNC Symbol;Acc:3588]	-1.275477366	4.332149056	13.42203882	0.000248685	0.003392129	4.884277045	4.857562122	3.645664403	3.266541267	4.304558099
ENSG00000166226	CCT2	chaperonin containing TCP1, subunit 2 (beta) [Source:HGNC Symbol;Acc:1615]	-1.246323737	6.890103716	13.42298066	0.00024856	0.003392129	7.439414943	7.459693454	6.346216488	5.996001926	6.614365337
ENSG00000152556	PFKM	phosphofructokinase, muscle [Source:HGNC Symbol;Acc:8877]	-1.285147684	4.559680848	13.41933728	0.000249044	0.003392919	5.192927623	5.299008392	4.063312903	3.353408508	3.880000416
ENSG00000204110	RP1-153P14.8		2.204587675	0.402114731	13.4186893	0.00024913	0.003392919	-0.856736938	0.171040209	1.415193986	-0.296766077	0.264147325
ENSG00000131724	IL13RA1	interleukin 13 receptor, alpha 1 [Source:HGNC Symbol;Acc:5974]	1.557117411	6.432992689	13.38795908	0.000253245	0.003443324	4.906013124	5.809497926	6.257379042	7.253917993	6.864635841
ENSG00000178146	RP1-232L22__B.1		1.663033142	4.072879325	13.38666745	0.000253419	0.003443324	3.053437594	3.036849946	3.634266777	4.764591224	4.796031523
ENSG00000231027	AC079325.6		1.991878799	3.156118245	13.388339	0.000253194	0.003443324	1.397104685	2.287305009	2.723873993	4.18537154	3.581615324
ENSG00000134245	WNT2B	wingless-type MMTV integration site family, member 2B [Source:HGNC Symbol;Acc:12781]	2.116060141	-0.181164101	13.38260448	0.000253969	0.003448123	-1.350769713	0.016063025	0.471574783	-0.009775356	-1.55968627
ENSG00000102003	SYP	synaptophysin [Source:HGNC Symbol;Acc:11506]	1.979548169	0.296733501	13.3756369	0.000254914	0.003458281	-0.046168491	-0.464918547	0.778135316	0.556835562	0.002986071
ENSG00000179115	FARSA	phenylalanyl-tRNA synthetase, alpha subunit [Source:HGNC Symbol;Acc:3592]	-1.161037515	5.533979172	13.36401451	0.000256499	0.003477092	6.041769892	5.984308824	4.878855169	4.759828599	5.513426495

ENSG00000100592	DAAM1	dishevelled associated activator of morphogenesis 1 [Source:HGNC Symbol;Acc:18142]	-1.286367546	4.846029848	13.35929611	0.000257145	0.003483161	5.127030457	5.627378336	4.300507637	3.597162784	4.7717325
ENSG00000010327	STAB1	stabilin 1 [Source:HGNC Symbol;Acc:18628]	1.76671394	8.013462203	13.35657824	0.000257518	0.003485523	6.183268745	6.977343349	7.672943612	8.960654563	8.610124806
ENSG00000158517	NCF1	neutrophil cytosolic factor 1 [Source:HGNC Symbol;Acc:7660]	1.490700295	5.890837696	13.3514046	0.000258229	0.00349246	4.70812895	5.049623575	5.547091836	6.733841745	6.391585327
ENSG00000121053	EPX	eosinophil peroxidase [Source:HGNC Symbol;Acc:3423]	-3.333944703	1.674635683	13.34260645	0.000259443	0.003506182	3.719122423	0.583576386	-2.539967122	-2.0191864	-0.649489103
ENSG00000137261	KIAA0319	KIAA0319 [Source:HGNC Symbol;Acc:21580]	2.411915378	-0.060801179	13.3244739	0.000261964	0.003537527	-0.856736938	-1.507080648	-1.886652327	0.642123966	0.84598249
ENSG00000138587	MNS1	meiosis-specific nuclear structural 1 [Source:HGNC Symbol;Acc:29636]	-2.438119793	0.769745751	13.31778836	0.0002629	0.003547433	1.752861822	1.537699846	-3.766908349	-2.419096061	0.763888603
ENSG00000161642	ZNF385A	zinc finger protein 385A [Source:HGNC Symbol;Acc:17521]	1.620135535	7.155253348	13.29165245	0.00026659	0.003594466	5.707029501	6.445591319	6.990069448	8.20098638	7.244450159
ENSG00000241975	TCEB1P19	transcription elongation factor B (SIII), polypeptide 1 pseudogene 19 [Source:HGNC Symbol;Acc:38155]	2.177307604	0.397581207	13.2852539	0.000267502	0.003603986	-0.942926472	-0.025429826	0.663204908	1.187332556	-0.095747566
ENSG00000095303	PTGS1	prostaglandin-endoperoxide synthase 1 (prostaglandin G/H synthase and cyclooxygenase) [Source:HGNC Symbol;Acc:9604]	1.624794647	6.738549814	13.27660518	0.000268739	0.003617874	5.429996748	5.930395455	6.376174842	7.832916182	6.888238838
ENSG00000176124	DLEU1	deleted in lymphocytic leukemia 1 (non-protein coding) [Source:HGNC Symbol;Acc:13747]	-1.663557879	2.837047558	13.26435747	0.0002705	0.003636259	3.632370594	3.491317573	2.118106677	1.0274492	2.435629945
ENSG00000145936	KCNMB1	potassium large conductance calcium-activated channel, subfamily M, beta member 1 [Source:HGNC Symbol;Acc:6285]	2.083021519	2.905333577	13.26422935	0.000270519	0.003636259	1.018120824	2.261659784	2.580794782	3.95477112	3.146787158
ENSG00000162878	PKDCC	protein kinase domain containing, cytoplasmic [Source:HGNC Symbol;Acc:25123]	-2.291073691	0.931344676	13.2587291	0.000271314	0.003644154	1.397104685	2.144940142	-2.539967122	-2.205328685	0.819133578
ENSG00000239672	NME1	NME/NM23 nucleoside diphosphate kinase 1 [Source:HGNC Symbol;Acc:7849]	-1.535145217	3.273524796	13.22523185	0.000276206	0.003707025	4.271869887	3.664397071	2.330744516	2.014059105	2.807527639
ENSG00000247950	SEC24B-AS1	SEC24B antisense RNA 1 [Source:HGNC Symbol;Acc:44003]	1.978370335	0.099341665	13.22104631	0.000276823	0.003712474	-0.775407551	0.133828548	0.75024962	0.192266291	-0.80787826
ENSG00000117215	PLA2G2D	phospholipase A2, group IID [Source:HGNC Symbol;Acc:9033]	-2.157864389	0.919283337	13.21928424	0.000277083	0.003713129	2.266432806	1.565913615	-0.82139248	-2.419096061	-0.649489103
ENSG00000144554	FANCD2	Fanconi anemia, complementation group D2 [Source:HGNC Symbol;Acc:3585]	-1.226077654	5.272735075	13.20192256	0.000279662	0.003744827	5.751443891	5.938510869	4.844648634	4.079778361	5.007010895
ENSG00000160593	AMICA1	adhesion molecule, interacts with CXADR antigen 1 [Source:HGNC Symbol;Acc:19084]	1.803349922	8.736034899	13.19770654	0.000280292	0.0037504	7.181919154	7.828215361	8.767241674	9.674037828	8.984671054
ENSG00000164951	PDP1	pyruvate dehyrogenase phosphatase catalytic subunit 1 [Source:HGNC Symbol;Acc:9279]	1.17258672	5.35689613	13.19419306	0.000280818	0.003754576	4.76870122	4.779898163	5.898649044	5.681188062	5.283553932
ENSG00000033327	GAB2	GRB2-associated binding protein 2 [Source:HGNC Symbol;Acc:14458]	1.584899963	6.199561234	13.17746643	0.000283336	0.003785355	4.896737732	5.224635763	5.74816569	7.219932093	6.602605111
ENSG00000186314	PRELID2	PRELI domain containing 2 [Source:HGNC Symbol;Acc:28306]	-1.874131407	1.568812483	13.17548203	0.000283636	0.003786483	2.5449974	2.116608898	-0.059127673	-0.46496394	1.428856327
ENSG00000206053	HN1L	hematological and neurological expressed 1-like [Source:HGNC Symbol;Acc:14137]	-1.225181809	5.524431333	13.13391212	0.000289999	0.003865547	6.036852591	6.127841572	5.148250672	4.301347534	5.288455913
ENSG00000113916	BCL6	B-cell CLL/lymphoma 6 [Source:HGNC Symbol;Acc:1001]	1.628888636	6.990349434	13.13517101	0.000289804	0.003865547	5.708797246	5.61223861	6.376174842	7.789957742	7.844757048
ENSG00000111729	CLEC4A	C-type lectin domain family 4, member A [Source:HGNC Symbol;Acc:13257]	1.660553009	5.101984032	13.13051877	0.000290524	0.003869616	3.669153174	4.503636694	4.854504969	5.985160969	5.451399363
ENSG00000178921	PFAS	phosphoribosylformylglycinamidine synthase [Source:HGNC Symbol;Acc:8863]	-1.277065455	5.261994236	13.12605026	0.000291218	0.003875916	5.776945226	5.960595701	4.872401862	3.906748526	4.91491577
ENSG00000156968	MPV17L	MPV17 mitochondrial membrane protein-like [Source:HGNC Symbol;Acc:26827]	-2.20438506	0.915166821	13.11227313	0.000293368	0.003901566	1.871242652	1.955327308	-3.766908349	-1.336401299	0.139468177
ENSG00000248019	FAM13A-AS1	FAM13A antisense RNA 1 [Source:HGNC Symbol;Acc:19370]	1.405105327	4.52953018	13.10340239	0.00029476	0.003917116	3.567603998	4.244706846	4.834724499	5.173171083	4.304558099
ENSG00000234062	RP11-308D16.4		1.993962562	-0.11059726	13.09965451	0.00029535	0.003921992	-0.856736938	-0.112176905	0.081791624	0.229488395	-0.649489103
ENSG00000175471	MCTP1	multiple C2 domains, transmembrane 1 [Source:HGNC Symbol;Acc:26183]	1.454203792	5.033962141	13.09038412	0.000296815	0.00393847	3.632370594	4.287693936	4.724388461	5.902589236	5.502920638
ENSG00000038945	MSR1	macrophage scavenger receptor 1 [Source:HGNC Symbol;Acc:7376]	1.53122116	2.887432017	13.07670789	0.00029899	0.003964334	1.725181674	1.997369264	2.961306749	3.399046051	3.473850543
ENSG00000105810	CDK6	cyclin-dependent kinase 6 [Source:HGNC Symbol;Acc:1777]	-1.237013054	6.731384465	13.05276736	0.000302837	0.004012299	7.148443281	7.283743127	6.323923603	5.822635386	6.5937216
ENSG00000260078	RP11-44F14.1		1.891255429	3.104105456	13.03942169	0.000305002	0.004037945	1.806678074	2.163523194	2.680205034	4.017298632	3.601447293

ENSG00000253325	IGHV7-34-1	immunoglobulin heavy variable 7-34-1 (pseudogene) [Source:HGNC Symbol;Acc:5664]	-2.947616851	-0.098871178	13.02248062	0.000307774	0.004071567	1.37945544	0.343933181	-3.233942493	-4.730697034	-1.42520211
ENSG00000262766	RP11-196G11.4		1.948381033	1.620837751	13.00853732	0.000310074	0.004098908	0.97176043	0.859104642	1.674560554	2.551085884	1.177268129
ENSG00000186047	DLEU7	deleted in lymphocytic leukemia, 7 [Source:HGNC Symbol;Acc:17567]	1.91830351	1.628422812	13.00179248	0.000311193	0.004110604	0.46968809	1.128690501	1.598401271	2.420374188	1.688229459
ENSG00000125257	ABCC4	ATP-binding cassette, sub-family C (CFTR/MRP), member 4 [Source:HGNC Symbol;Acc:55]	-1.180109587	4.857783853	12.99409377	0.000312475	0.004124436	5.404045254	5.498822445	4.278711965	4.199509687	4.272754352
ENSG00000155189	AGPAT5	1-acylglycerol-3-phosphate O-acyltransferase 5 [Source:HGNC Symbol;Acc:20886]	-1.209185985	4.886318638	12.99159781	0.000312892	0.004126834	5.415997664	5.435243807	4.266459447	3.653025764	4.897374905
ENSG00000157306	RP11-66N24.4		1.785716893	1.394281245	12.98942809	0.000313255	0.004128517	0.534915169	1.109650305	1.788624578	1.75796486	1.279288931
ENSG00000240041	IGHJ4	immunoglobulin heavy joining 4 [Source:HGNC Symbol;Acc:5538]	-1.632343768	2.948838894	12.98127704	0.000314621	0.004143419	3.942895577	2.995959146	0.75024962	3.140302572	2.153441144
ENSG00000149573	MPZL2	myelin protein zero-like 2 [Source:HGNC Symbol;Acc:3496]	1.675579575	2.38954834	12.97603198	0.000315504	0.004151927	1.084982398	1.508923295	2.330744516	2.650493526	3.275806983
ENSG00000168255	POLR2J3	polymerase (RNA) II (DNA directed) polypeptide J3 [Source:HGNC Symbol;Acc:33853]	1.594821204	2.614394649	12.96052399	0.000318128	0.004183325	1.752861822	2.278807152	3.026695712	3.378480503	1.829457167
ENSG00000161921	CXCL16	chemokine (C-X-C motif) ligand 16 [Source:HGNC Symbol;Acc:16642]	1.569657411	5.73867773	12.9524393	0.000319505	0.004195145	4.147044042	5.117913874	5.575179501	6.736672335	5.905186683
ENSG00000166825	ANPEP	alanyl (membrane) aminopeptidase [Source:HGNC Symbol;Acc:500]	1.714628741	7.538688044	12.95317505	0.000319379	0.004195145	5.85803785	6.956514725	7.578723449	8.711017077	7.097526627
ENSG00000181234	TMEM132C	transmembrane protein 132C [Source:HGNC Symbol;Acc:25436]	-2.645009075	0.096349119	12.9441256	0.000320927	0.004210667	1.084982398	1.255368251	-2.845623393	-4.730697034	-1.55968627
ENSG00000131389	SLC6A6	solute carrier family 6 (neurotransmitter transporter), member 6 [Source:HGNC Symbol;Acc:11052]	1.437435607	7.081052563	12.90188375	0.000328252	0.004303552	5.87781472	6.234407269	6.919442354	7.996881928	7.376058358
ENSG00000246203	RP11-29H23.5		1.952960238	-0.168830407	12.89686556	0.000329133	0.004311886	-0.625332215	-0.204475541	0.210162819	0.074465887	-1.18885156
ENSG00000141873	SLC39A3	solute carrier family 39 (zinc transporter), member 3 [Source:HGNC Symbol;Acc:17128]	-1.209393568	4.627740111	12.89151841	0.000330075	0.004320998	5.281115145	5.120294025	3.879237643	3.836109456	4.368453247
ENSG00000246430	LINC00968	long intergenic non-protein coding RNA 968 [Source:HGNC Symbol;Acc:48727]	2.043767211	1.289168938	12.88998876	0.000330344	0.004321309	-0.046168491	0.764888368	0.884581662	1.86827773	1.920685729
ENSG00000267169	CTB-55O6.12		1.648993262	3.044840495	12.88337471	0.000331514	0.004333378	2.049222129	2.361613919	2.955212673	3.817895715	3.300281982
ENSG00000108839	ALOX12	arachidonate 12-lipoxygenase [Source:HGNC Symbol;Acc:429]	2.224313307	3.688675089	12.86780314	0.000334284	0.004366333	1.63880454	2.959203556	3.066814926	5.028876849	3.613216922
ENSG00000075399	VPS9D1	VPS9 domain containing 1 [Source:HGNC Symbol;Acc:13526]	1.186236607	5.241396468	12.86289982	0.000335161	0.004374534	4.503374838	4.6359475	5.437699584	5.728270006	5.491275141
ENSG00000007968	E2F2	E2F transcription factor 2 [Source:HGNC Symbol;Acc:3114]	-1.323955862	4.92347364	12.85756434	0.000336118	0.004380511	5.298828106	5.51700098	3.527421294	4.139664188	5.214406097
ENSG00000114405	C3orf14	chromosome 3 open reading frame 14 [Source:HGNC Symbol;Acc:25024]	2.265207072	0.980113505	12.85783693	0.000336069	0.004380511	-0.489407866	0.527293245	0.602113338	1.914722448	1.090116303
ENSG00000197122	SRC	v-src avian sarcoma (Schmidt-Ruppin A-2) viral oncogene homolog [Source:HGNC Symbol;Acc:11283]	1.621854418	5.359321359	12.85303858	0.000336932	0.00438659	4.077598864	4.710507559	4.960222513	6.452252305	5.432701873
ENSG00000185168	LINC00482	long intergenic non-protein coding RNA 482 [Source:HGNC Symbol;Acc:26816]	2.616816796	0.813778248	12.85219262	0.000337084	0.00438659	-1.608074432	0.376157587	0.602113338	2.165783527	-0.257803757
ENSG00000115457	IGFBP2	insulin-like growth factor binding protein 2, 36kDa [Source:HGNC Symbol;Acc:5471]	-1.550249795	2.505683281	12.84381576	0.000338596	0.004396197	3.424338996	3.408048696	1.415193986	1.109604583	0.923655414
ENSG00000121989	ACVR2A	activin A receptor, type IIA [Source:HGNC Symbol;Acc:173]	1.392122446	3.434641776	12.8424621	0.000338841	0.004396197	2.636996308	3.138979644	4.153899108	3.86004218	2.686482162
ENSG00000164308	ERAP2	endoplasmic reticulum aminopeptidase 2 [Source:HGNC Symbol;Acc:29499]	1.495237087	5.811854181	12.84485068	0.000338409	0.004396197	5.141500364	5.284225168	5.807720546	6.989825366	4.534454294
ENSG00000256604	AC093668.2		2.034174396	-0.235335893	12.84117614	0.000339074	0.004396197	-0.856736938	-0.855949929	0.036330944	0.192266291	-0.506781561
ENSG00000267990	CTD-3187F8.15		2.493168999	0.308541187	12.84264824	0.000338808	0.004396197	-1.132483902	-0.713501401	-0.99931534	1.069111613	1.177268129
ENSG00000180953	ST20	suppressor of tumorigenicity 20 [Source:HGNC Symbol;Acc:33520]	1.815896955	2.395246707	12.83846565	0.000339566	0.004399321	1.210071084	1.634124584	2.202847421	3.03386134	2.968901188
ENSG00000169604	ANTXR1	anthrax toxin receptor 1 [Source:HGNC Symbol;Acc:21014]	-3.983310485	-0.068324457	12.83368774	0.000340434	0.004404069	1.37945544	0.664086661	-7.002070102	-4.730697034	-2.060109799
ENSG00000204681	GABBR1	gamma-aminobutyric acid (GABA) B receptor, 1 [Source:HGNC Symbol;Acc:4070]	1.181820448	6.584013646	12.83459063	0.00034027	0.004404069	5.736790496	6.112979157	7.000833835	7.199266765	6.354668562
ENSG00000181619	GPR135	G protein-coupled receptor 135 [Source:HGNC Symbol;Acc:19991]	1.964604081	0.699375221	12.8319526	0.00034075	0.004404909	-0.196880952	0.498306264	0.778135316	1.224680533	0.584199832
ENSG00000173846	PLK3	polo-like kinase 3 [Source:HGNC Symbol;Acc:2154]	1.51245256	5.4506114	12.82691113	0.000341669	0.004413543	4.432399102	4.781402895	5.273532496	6.62718374	4.987549827

ENSG00000004478	FKBP4	FK506 binding protein 4, 59kDa [Source:HGNC Symbol;Acc:3720]	-1.220090496	5.176774021	12.82011668	0.000342912	0.004426343	5.813550226	5.686396784	4.300507637	4.626653563	4.828367502
ENSG00000143502	SUSD4	sushi domain containing 4 [Source:HGNC Symbol;Acc:25470]	1.919700664	0.964187851	12.81294911	0.000344229	0.004440068	-0.250832646	0.610915408	1.674560554	0.696304622	1.218946104
ENSG00000014257	ACPP	acid phosphatase, prostate [Source:HGNC Symbol;Acc:125]	1.578368556	4.709364907	12.80652097	0.000345413	0.004452078	3.598444871	4.046212993	4.357069198	5.560401742	5.096376185
ENSG00000213240	NOTCH2NL	notch 2 N-terminal like [Source:HGNC Symbol;Acc:31862]	1.498766883	3.874107592	12.80280474	0.0003461	0.004457657	3.047861816	3.234355	3.926726325	4.630137237	3.943532595
ENSG00000188690	UROS	uroporphyrinogen III synthase [Source:HGNC Symbol;Acc:12592]	-1.162126904	5.356032295	12.79319338	0.000347883	0.004477333	5.891109787	5.996668693	4.874017897	4.414762411	4.936936424
ENSG00000149548	CCDC15	coiled-coil domain containing 15 [Source:HGNC Symbol;Acc:25798]	-1.636492755	2.393366915	12.78315182	0.000349755	0.004498133	3.11338967	3.323814258	1.501363361	0.265774258	1.702990823
ENSG00000090861	AARS	alanyl-tRNA synthetase [Source:HGNC Symbol;Acc:20]	-1.18353948	5.891745775	12.76949542	0.000352318	0.004527775	6.390758198	6.432242965	5.48816597	4.819023673	5.733389055
ENSG00000064547	LPAR2	lysophosphatidic acid receptor 2 [Source:HGNC Symbol;Acc:3168]	1.177599808	5.702562343	12.76519096	0.00035313	0.004534886	4.881144984	4.837449986	5.703420094	6.25257982	6.203913664
ENSG00000253293	HOXA10	homeobox A10 [Source:HGNC Symbol;Acc:5100]	-1.771079877	1.810708303	12.75980158	0.000354148	0.004544646	2.446729819	2.329059307	-0.059127673	-0.406691783	2.3444256
ENSG00000155090	KLF10	Kruppel-like factor 10 [Source:HGNC Symbol;Acc:11810]	1.694917882	7.381366537	12.75763194	0.000354559	0.004546597	6.530750089	6.290435609	7.303909608	8.757847616	6.163794847
ENSG00000204397	CARD16	caspase recruitment domain family, member 16 [Source:HGNC Symbol;Acc:33701]	1.338337534	5.632560941	12.75570575	0.000354925	0.00454796	4.763607814	5.032033685	5.557186035	6.384769319	5.839068896
ENSG00000076067	RBMS2	RNA binding motif, single stranded interacting protein 2 [Source:HGNC Symbol;Acc:9909]	1.713627297	2.253852834	12.74467552	0.000357024	0.004568191	1.084982398	1.551875699	2.192523064	2.806428868	2.800681624
ENSG00000182795	C1orf116	chromosome 1 open reading frame 116 [Source:HGNC Symbol;Acc:28667]	2.007649926	0.517612557	12.74582776	0.000356804	0.004568191	-0.196880952	0.016063025	0.28980717	1.224680533	0.518960621
ENSG00000214274	ANG	angiogenin, ribonuclease, RNase A family, 5 [Source:HGNC Symbol;Acc:483]	2.140073655	0.63909667	12.73359747	0.000359145	0.004591982	-0.196880952	-0.068151468	-0.010608983	1.446384807	0.973207307
ENSG00000257743	RP11-1220K2.2	Putative inactive maltase-glucoamylase-like protein LOC93432 [Source:UniProtKB/Swiss-Prot;Acc:Q2M2H8]	2.34310796	0.198345154	12.73093001	0.000359658	0.004595189	-0.250832646	-2.076136033	-0.520331194	0.669468625	1.090116303
ENSG00000203306	AP001007.1		1.703457018	3.705838066	12.72635032	0.000360539	0.00459976	2.599373756	3.086385644	3.477270088	4.730917879	3.666906879
ENSG00000109272	PF4V1	platelet factor 4 variant 1 [Source:HGNC Symbol;Acc:8862]	1.728622347	2.36619712	12.72751753	0.000360314	0.00459976	0.769809504	1.724506406	2.404162378	2.498689587	3.260920169
ENSG00000261827	RP11-340F14.5		2.053894389	2.660945383	12.72441608	0.000360913	0.004601176	1.229903997	2.107040151	2.192523064	3.582852389	3.02284327
ENSG00000130826	DKC1	dyskeratosis congenita 1, dyskerin [Source:HGNC Symbol;Acc:2890]	-1.207570722	6.190735838	12.72210589	0.000361359	0.00460352	6.738416765	6.725118065	5.849390023	5.173171083	5.873730193
ENSG00000167670	CHAF1A	chromatin assembly factor 1, subunit A (p150) [Source:HGNC Symbol;Acc:1910]	-1.2087536	4.961713303	12.71347835	0.00036303	0.004621453	5.402953745	5.576282205	3.966655493	4.231969659	4.957087522
ENSG00000136161	RCBTB2	regulator of chromosome condensation (RCC1) and BTB (POZ) domain containing protein 2 [Source:HGNC Symbol;Acc:1914]	1.278457538	5.807372276	12.7098227	0.00036374	0.004627141	4.810461175	5.318832075	5.854310438	6.383221785	6.13822683
ENSG00000078399	HOXA9	homeobox A9 [Source:HGNC Symbol;Acc:5109]	-1.957917665	1.403204509	12.69788625	0.000366069	0.004653399	2.093195522	1.797710702	-0.82139248	-1.70641174	2.120832889
ENSG00000112210	RAB23	RAB23, member RAS oncogene family [Source:HGNC Symbol;Acc:14263]	-1.814867972	1.595945067	12.69108855	0.000367402	0.004666967	2.332130562	2.591720352	-0.10933503	-0.956081001	1.198257616
ENSG00000177706	FAM20C	family with sequence similarity 20, member C [Source:HGNC Symbol;Acc:22140]	1.920780433	3.78234343	12.68696284	0.000368214	0.004673893	1.968888231	2.871498754	3.009154251	4.761417889	4.4668719
ENSG00000266389	CTB-41I6.1		1.944182561	0.870402418	12.68428249	0.000368742	0.004677216	0.174562846	-0.112176905	0.884581662	1.493047208	1.11240278
ENSG00000141505	ASGR1	asialoglycoprotein receptor 1 [Source:HGNC Symbol;Acc:742]	1.653037796	4.072747936	12.68257759	0.000369078	0.004678102	2.758036868	3.55604389	3.98172333	5.150304328	3.806723591
ENSG00000214517	PPME1	protein phosphatase methylesterase 1 [Source:HGNC Symbol;Acc:30178]	-1.178020664	4.763948154	12.67964803	0.000369657	0.004678681	5.221688852	5.480411926	4.034700554	3.892314563	4.481928444
ENSG00000241163	LINC00877	long intergenic non-protein coding RNA 877 [Source:HGNC Symbol;Acc:27706]	1.873159476	3.691798458	12.68020165	0.000369548	0.004678681	2.480234919	2.66528446	3.149202463	4.922089668	3.72229752
ENSG00000091127	PUS7	pseudouridylate synthase 7 homolog (S. cerevisiae) [Source:HGNC Symbol;Acc:26033]	-1.378445568	3.579652245	12.6710406	0.000371363	0.004696883	4.304969154	4.30244184	2.847469331	2.258736156	2.999117651
ENSG00000257576	RP11-153M3.1		-1.370371515	3.682623088	12.6650374	0.000372557	0.004708596	4.406491176	4.277066647	2.918097532	2.36290873	3.407808836
ENSG00000174516	PELI3	pellino E3 ubiquitin protein ligase family member 3 [Source:HGNC Symbol;Acc:30010]	1.419353795	3.575558035	12.66143836	0.000373275	0.004710885	2.599373756	3.0717027	3.859795663	4.144544252	3.663137444
ENSG00000100767	PAPLN	papilin, proteoglycan-like sulfated glycoprotein [Source:HGNC Symbol;Acc:19262]	1.961607092	0.915856499	12.66203924	0.000373155	0.004710885	0.133035942	-0.30308518	-0.010608983	0.917704421	2.14265341

ENSG00000249506	ZEB2P1	zinc finger E-box binding homeobox 2 pseudogene 1 [Source:HGNC Symbol;Acc:33346]	2.034111827	1.947513845	12.65958675	0.000373645	0.004712166	0.874315765	1.109650305	1.450281721	2.848906753	2.3444256
ENSG00000123360	PDE1B	phosphodiesterase 1B, calmodulin-dependent [Source:HGNC Symbol;Acc:8775]	1.607547784	3.180072005	12.65682392	0.000374197	0.004715746	2.266432806	2.564011614	3.170400428	3.995855935	3.225574983
ENSG00000224505	AC002117.1		1.751183663	1.294498072	12.65333721	0.000374896	0.00472116	0.435934254	0.740340508	1.980862385	1.523337554	1.11240278
ENSG00000234119	AC079248.1		-1.174949186	4.65831754	12.6399748	0.000377585	0.004751617	5.243804382	5.294800048	4.074600823	3.628851426	4.292408717
ENSG00000082516	GEMIN5	gem (nuclear organelle) associated protein 5 [Source:HGNC Symbol;Acc:20043]	-1.234517984	4.627006779	12.63094852	0.000379413	0.004771195	5.245023138	5.221313549	4.107944152	3.340707202	4.368453247
ENSG00000140678	ITGAX	integrin, alpha X (complement component 3 receptor 4 subunit) [Source:HGNC Symbol;Acc:6152]	1.849088227	8.418318018	12.62865857	0.000379878	0.004773624	6.867468202	7.374025033	8.102342888	9.549294248	8.656600438
ENSG00000183814	LIN9	lin-9 homolog (C. elegans) [Source:HGNC Symbol;Acc:30830]	-1.487049781	3.066136482	12.62469431	0.000380684	0.004780336	3.79763378	3.752958575	2.253389193	1.27894994	2.807527639
ENSG00000135740	SLC9A5	solute carrier family 9, subfamily A (NHE5, cation proton antiporter 5), member 5 [Source:HGNC Symbol;Acc:11078]	1.921577777	0.130628398	12.58600148	0.000388647	0.004876836	-0.489407866	0.016063025	0.778135316	-0.099242291	-0.257803757
ENSG00000174442	ZWILCH	zwilch kinetochore protein [Source:HGNC Symbol;Acc:25468]	-1.226600061	4.387805595	12.56957279	0.000392078	0.004916381	4.970796966	4.972680213	3.634266777	3.284338193	4.282615004
ENSG00000272065	U91328.20		2.586473339	0.062400846	12.55324438	0.000395519	0.004955986	-1.350769713	-0.782968247	-0.907612816	1.089500161	0.378903451
ENSG00000235568	NFAM1	NFAT activating protein with ITAM motif 1 [Source:HGNC Symbol;Acc:29872]	1.636856558	7.743019642	12.54638673	0.000396973	0.004970659	6.034740035	6.906022708	7.529328845	8.689995164	8.168172141
ENSG00000164136	IL15	interleukin 15 [Source:HGNC Symbol;Acc:5977]	1.779426577	3.780366702	12.54285993	0.000397723	0.00497392	2.744312733	2.84296036	3.357618296	4.795946491	4.045503588
ENSG00000254952	AP001257.1		2.724902313	0.30788238	12.54249759	0.0003978	0.00497392	-1.921450438	-0.252938016	-0.10933503	1.446384807	0.182236344
ENSG00000183486	MX2	myxovirus (influenza virus) resistance 2 (mouse) [Source:HGNC Symbol;Acc:7533]	1.325102861	6.673131821	12.53149358	0.00040015	0.004999737	5.499046223	5.990827216	6.562588821	7.401362161	7.104144929
ENSG00000173083	HPSE	heparanase [Source:HGNC Symbol;Acc:5164]	1.583412617	5.679158659	12.52704695	0.000401103	0.005008085	3.846627143	5.108353807	5.45939078	6.275562429	6.419224707
ENSG00000116221	MRPL37	mitochondrial ribosomal protein L37 [Source:HGNC Symbol;Acc:14034]	-1.142165088	5.522926902	12.52012736	0.000402591	0.005023094	6.1075688	6.054440781	4.841348169	4.865577827	5.184477247
ENSG00000239392	RP11-537H15.4		1.452890996	3.673197988	12.51852616	0.000402937	0.00502383	2.894768068	3.056868784	3.697694186	4.294777486	3.921613159
ENSG00000173207	CKS1B	CDC28 protein kinase regulatory subunit 1B [Source:HGNC Symbol;Acc:19083]	-1.585480241	2.218207792	12.51616941	0.000403445	0.005026601	2.913284701	2.910527228	1.120696904	0.642123966	2.064792492
ENSG00000163421	PROK2	prokineticin 2 [Source:HGNC Symbol;Acc:18455]	2.236958	4.621136132	12.51120649	0.000404518	0.005036396	2.850615345	2.69752161	2.880002288	5.394153653	5.899590916
ENSG00000237188	RP11-337C18.8		1.874509025	0.530851514	12.50518626	0.000405824	0.00504907	0.000762307	0.133828548	0.602113338	0.773941511	0.706443614
ENSG00000229749	COTL1P1	coactosin-like 1 (Dictyostelium) pseudogene 1 [Source:HGNC Symbol;Acc:2086]	1.733043007	1.727830545	12.49273391	0.000408538	0.005079237	0.94800933	1.128690501	1.907011864	2.294302704	1.81594051
ENSG00000231486	AC096579.7		-1.717495538	3.605895177	12.47865319	0.000411629	0.005114044	4.787224689	3.53121644	2.321298905	1.970751589	3.647960442
ENSG00000233429	HOTAIRM1	HOXA transcript antisense RNA, myeloid-specific 1 [Source:HGNC Symbol;Acc:37117]	1.414261069	3.691252684	12.46340358	0.000415003	0.005152319	2.876010686	2.995959146	3.826798551	4.406660334	3.765128041
ENSG00000058804	NDC1	NDC1 transmembrane nucleoporin [Source:HGNC Symbol;Acc:25525]	-1.169976088	5.264838534	12.43731888	0.000420839	0.005221086	5.754870226	5.856654423	4.799442354	4.097503316	5.13090081
ENSG00000162444	RBP7	retinol binding protein 7, cellular [Source:HGNC Symbol;Acc:30316]	1.443606757	4.108518989	12.43473438	0.000421422	0.005224624	3.11338967	3.703019536	4.241637747	4.88028041	3.992417811
ENSG00000215769	hsa-mir-6080	hsa-mir-6080 [Source:miRBase;Acc:MI0020357]	1.409031393	3.5012135	12.43281141	0.000421856	0.005226315	2.75119112	2.831385036	3.747912684	4.009294922	3.696712964
ENSG00000099889	ARVCF	armadillo repeat gene deleted in velocardiofacial syndrome [Source:HGNC Symbol;Acc:728]	-1.394796575	3.665688748	12.42202469	0.0004243	0.005252882	4.478721339	4.337642778	2.507726234	2.704366551	3.074840951
ENSG00000116729	WLS	wntless Wnt ligand secretion mediator [Source:HGNC Symbol;Acc:30238]	1.932433898	4.028494122	12.41462521	0.000425984	0.00527002	2.114689606	3.061830389	3.19129143	4.912108157	4.877993003
ENSG00000156509	FBXO43	F-box protein 43 [Source:HGNC Symbol;Acc:28521]	-2.209270339	0.867467863	12.40738913	0.000427638	0.005286754	1.531025871	1.761572815	-3.766908349	-1.70641174	1.11240278
ENSG00000243970	PPIEL	peptidylprolyl isomerase E-like pseudogene [Source:HGNC Symbol;Acc:33195]	1.939898458	0.064990044	12.38887608	0.000431899	0.005335672	-0.698419327	0.171040209	0.9100098	0.03296005	-1.70800759
ENSG00000062282	DGAT2	diacylglycerol O-acyltransferase 2 [Source:HGNC Symbol;Acc:16940]	1.71128319	4.227529738	12.38581017	0.000432609	0.005340681	2.949619895	3.516835107	3.786179228	5.246499197	4.488333455
ENSG00000253522	MIR146A	microRNA 146a [Source:HGNC Symbol;Acc:31533]	1.911239854	0.844190767	12.3794129	0.000434094	0.005355245	-0.365194284	0.664086661	1.450281721	0.940331404	0.763888603
ENSG00000141096	DPEP3	dipeptidase 3 [Source:HGNC Symbol;Acc:23029]	2.43028119	0.511616578	12.37146426	0.000435946	0.005374315	-0.046168491	-1.629706251	0.081791624	1.508271876	0.735452007
ENSG00000065154	OAT	ornithine aminotransferase [Source:HGNC Symbol;Acc:8091]	-1.247855905	5.394632525	12.3648888	0.000437484	0.005389491	5.899647647	6.077284614	4.326707234	4.786611257	5.134988789
ENSG00000177469	PTRF	polymerase I and transcript release factor [Source:HGNC Symbol;Acc:9688]	-1.742558437	2.724155718	12.35006318	0.000440972	0.00542865	3.594625625	3.476530889	0.036330944	1.638513559	2.426764636

ENSG00000091986	CCDC80	coiled-coil domain containing 80 [Source:HGNC Symbol;Acc:30649]	-2.623595275	0.562397597	12.34642537	0.000441832	0.005431621	2.071376358	1.355744383	-2.539967122	-4.730697034	-3.221241555
ENSG00000130529	TRPM4	transient receptor potential cation channel, subfamily M, member 4 [Source:HGNC Symbol;Acc:17993]	2.123651998	1.17274207	12.3475184	0.000441573	0.005431621	0.534915169	0.438524285	0.328039018	2.258736156	1.090116303
ENSG00000169385	RNASE2	ribonuclease, RNase A family, 2 (liver, eosinophil-derived neurotoxin) [Source:HGNC Symbol;Acc:10045]	-1.383682311	5.66496551	12.32623319	0.000446637	0.00548685	5.789528825	6.126066145	3.761945509	4.747050861	6.456724691
ENSG00000076864	RAP1GAP	RAP1 GTPase activating protein [Source:HGNC Symbol;Acc:9858]	-2.020032251	1.537757665	12.32448757	0.000447055	0.005488143	2.217603867	2.948527637	-0.059127673	-2.97427061	-0.506781561
ENSG00000171943	SRGAP2C	SLIT-ROBO Rho GTPase activating protein 2C [Source:HGNC Symbol;Acc:30584]	1.904986152	1.161954002	12.31676513	0.000448908	0.005507044	0.329658892	0.277241192	0.805492218	1.55300499	1.920685729
ENSG00000138463	DIRC2	disrupted in renal carcinoma 2 [Source:HGNC Symbol;Acc:16628]	1.534911559	3.716107894	12.30797636	0.000451027	0.005529172	2.70942011	3.120075649	3.664462604	4.470237611	3.940421563
ENSG00000213070	HMGB3P6	high mobility group box 3 pseudogene 6 [Source:HGNC Symbol;Acc:39283]	-1.846453065	2.663931729	12.30218711	0.000452428	0.005542478	3.579246723	3.415820346	0.168628767	1.493047208	2.267069113
ENSG00000110628	SLC22A18	solute carrier family 22, member 18 [Source:HGNC Symbol;Acc:10964]	1.49106074	3.670710947	12.29759308	0.000453543	0.005552262	2.785099575	3.03180153	3.70497617	4.373789206	3.863670386
ENSG00000156299	TIAM1	T-cell lymphoma invasion and metastasis 1 [Source:HGNC Symbol;Acc:11805]	1.253542114	5.803382552	12.27529147	0.000458995	0.005615092	4.443058246	5.337364427	6.197975461	6.335473191	5.968406654
ENSG00000257433	RP1-197B17.3		1.779120517	0.749494718	12.27075288	0.000460113	0.005624845	0.254194054	0.242702272	1.030832979	0.847612951	0.948644093
ENSG00000091106	NLRC4	NLR family, CARD domain containing 4 [Source:HGNC Symbol;Acc:16412]	1.219057048	5.057098541	12.24954281	0.000465372	0.005685183	3.865767927	4.528939153	5.133484478	5.235105793	5.80184113
ENSG00000120594	PLXDC2	plexin domain containing 2 [Source:HGNC Symbol;Acc:21013]	1.481419937	6.665080307	12.24086798	0.000467541	0.005707704	4.860619147	5.853796571	6.397680654	7.500332646	7.275025896
ENSG00000145391	SETD7	SET domain containing (lysine methyltransferase) 7 [Source:HGNC Symbol;Acc:30412]	1.257374723	5.410591166	12.23103395	0.000470011	0.005733878	4.314288266	4.830198586	5.397814138	5.97902694	5.878614521
ENSG00000211945	IGHV1-18	immunoglobulin heavy variable 1-18 [Source:HGNC Symbol;Acc:5549]	-2.421162617	6.303414066	12.21478538	0.000474123	0.005778119	7.558159384	6.744534096	2.263288494	5.293392599	5.778511916
ENSG00000196678	ERI2	ERI1 exoribonuclease family member 2 [Source:HGNC Symbol;Acc:30541]	-1.350997803	3.911485982	12.21280848	0.000474625	0.005778119	4.497250847	4.451660263	2.657864658	2.98027543	4.025090588
ENSG00000227398	KIF9-AS1	KIF9 antisense RNA 1 [Source:HGNC Symbol;Acc:26822]	1.737755623	0.958836467	12.21389208	0.00047435	0.005778119	0.365960855	0.468724906	1.265630948	1.12943269	1.134350211
ENSG00000187017	ESPN	espin [Source:HGNC Symbol;Acc:13281]	-1.921869833	1.767813769	12.20982157	0.000475386	0.005783366	2.723478589	2.865836059	0.437007322	-2.419096061	0.341656497
ENSG00000143653	SCCPDH	saccharopine dehydrogenase (putative) [Source:HGNC Symbol;Acc:24275]	-1.15297431	5.07313092	12.19624388	0.000478858	0.005821578	5.62140306	5.614772957	4.273823438	4.229675155	4.986041891
ENSG00000173409	ARV1	ARV1 homolog (S. cerevisiae) [Source:HGNC Symbol;Acc:29561]	-1.250815775	4.443721932	12.18749742	0.000481109	0.00584489	5.237695108	4.927185486	3.836777169	3.301918251	4.019204856
ENSG00000267102	RP11-686D22.7		1.824142403	1.107443669	12.17694365	0.000483839	0.005873988	0.502670228	0.095631612	1.265630948	1.538247531	1.446507348
ENSG00000155465	SLC7A7	solute carrier family 7 (amino acid transporter light chain, y+L system), member 7 [Source:HGNC Symbol;Acc:11065]	1.594929854	7.779662061	12.15783644	0.00048882	0.005930369	6.160843748	7.046555665	7.691850148	8.819508299	7.883339374
ENSG00000116016	EPAS1	endothelial PAS domain protein 1 [Source:HGNC Symbol;Acc:3374]	-1.683751038	2.037085416	12.15625241	0.000489236	0.005931308	3.145070257	2.837184307	0.471574783	0.669468625	0.303422373
ENSG00000127249	ATP13A4	ATPase type 13A4 [Source:HGNC Symbol;Acc:25422]	1.81114913	0.413350528	12.14336066	0.000492629	0.005968327	0.090278201	0.095631612	0.9100098	0.894716898	-0.9858258
ENSG00000197182	FLJ27365	hsa-mir-4763 [Source:miRBase;Acc:MI0017404]	2.038098465	0.862792755	12.12600553	0.000497235	0.006015825	0.597320328	0.016063025	0.437007322	1.679439471	0.791775511
ENSG00000240704	KLF7P1	kruppel-like factor 7 pseudogene 1 [Source:HGNC Symbol;Acc:17207]	2.276725643	-0.074419399	12.12663119	0.000497068	0.006015825	-2.108017771	-0.355038393	0.401591237	0.192266291	0.002986071
ENSG00000105369	CD79A	CD79a molecule, immunoglobulin-associated alpha [Source:HGNC Symbol;Acc:1698]	-1.258490326	6.790902886	12.1231962	0.000497985	0.006020746	7.485773789	6.935380982	6.186966114	5.963231562	6.864225488
ENSG00000118985	ELL2	elongation factor, RNA polymerase II, 2 [Source:HGNC Symbol;Acc:17064]	-1.237707969	4.538191019	12.11028088	0.000501446	0.006057313	5.109740417	5.160167117	3.547813796	3.761832794	4.345117047
ENSG00000180539	C9orf139	chromosome 9 open reading frame 139 [Source:HGNC Symbol;Acc:31426]	1.382811566	3.316345342	12.10933808	0.000501699	0.006057313	2.177314774	2.658749679	3.71583069	3.497693538	3.837151666
ENSG00000139354	GAS2L3	growth arrest-specific 2 like 3 [Source:HGNC Symbol;Acc:27475]	-1.881703392	2.586708604	12.10427917	0.000503062	0.006069595	3.415706374	3.392378661	-0.907612816	1.36509501	2.444441111
ENSG00000124224	PPP4R1L	protein phosphatase 4, regulatory subunit 1-like [Source:HGNC Symbol;Acc:15755]	1.623404946	3.605129127	12.0974017	0.000504921	0.00608784	2.6444045	2.69752161	3.324906709	4.41071706	4.048396296
ENSG00000181409	AATK	apoptosis-associated tyrosine kinase [Source:HGNC Symbol;Acc:21]	1.256680572	4.597846185	12.09109012	0.000506633	0.00610429	3.833724066	4.317040508	4.88207103	5.320598436	4.099497077
ENSG00000224940	PRRT4	proline-rich transmembrane protein 4 [Source:HGNC Symbol;Acc:37280]	-1.982195925	2.964780158	12.08770129	0.000507555	0.0061112	3.547988195	1.832965453	0.168628767	0.074465887	4.420735849
ENSG00000162078	ZG16B	zymogen granule protein 16B [Source:HGNC Symbol;Acc:30456]	2.000719517	0.428524905	12.08466284	0.000508383	0.006116972	-0.698419327	0.095631612	0.328039018	0.984547783	0.615745481

ENSG00000115919	KYNU	kynureninase [Source:HGNC Symbol;Acc:6469]	1.463057668	4.174348407	12.07837189	0.000510101	0.006133443	3.236140248	3.527634524	4.066143174	5.023588668	4.304558099
ENSG00000268849	SIGLEC22P	sialic acid binding Ig-like lectin 22, pseudogene [Source:HGNC Symbol;Acc:15611]	1.896411371	2.361894252	12.057173	0.000515935	0.006199339	0.874315765	1.922971788	2.243421497	3.188355007	2.5705316
ENSG00000111845	PAK1IP1	PAK1 interacting protein 1 [Source:HGNC Symbol;Acc:20882]	-1.237958748	4.203650755	12.0450898	0.00051929	0.006235385	4.86696595	4.766284479	3.420897078	3.193073383	3.962059208
ENSG00000213462	ERV3-1	endogenous retrovirus group 3, member 1 [Source:HGNC Symbol;Acc:3454]	1.498216134	3.690460327	12.03248677	0.000522813	0.006273396	2.831271105	3.061830389	3.70497617	4.468292652	3.768640511
ENSG00000080839	RBL1	retinoblastoma-like 1 (p107) [Source:HGNC Symbol;Acc:9893]	-1.245891975	4.934048856	12.02735907	0.000524253	0.006286381	5.398579432	5.58492489	4.535127562	3.553798585	4.77871694
ENSG00000113594	LIFR	leukemia inhibitory factor receptor alpha [Source:HGNC Symbol;Acc:6597]	-2.537908371	0.526390082	12.01815483	0.000526848	0.006313189	2.082327188	1.237938318	-3.766908349	-2.670141381	-3.754655114
ENSG00000186204	CYP4F12	cytochrome P450, family 4, subfamily F, polypeptide 12 [Source:HGNC Symbol;Acc:18857]	2.069881	-0.171579375	12.01454043	0.000527871	0.00632113	-1.132483902	-0.464918547	0.437007322	-1.231517591	0.303422373
ENSG00000239998	LILRA2	leukocyte immunoglobulin-like receptor, subfamily A (with TM domain), member 2 [Source:HGNC Symbol;Acc:6603]	1.563597014	6.565692753	12.00963569	0.000529262	0.006333466	5.133625684	5.810972116	6.154754978	7.598292908	6.871185677
ENSG00000117586	TNFSF4	tumor necrosis factor (ligand) superfamily, member 4 [Source:HGNC Symbol;Acc:11934]	1.731949897	3.209752712	11.98017727	0.000537695	0.006425616	2.207636582	2.492318033	2.911817771	4.061832926	3.524590078
ENSG00000237206	IMPDH1P4	IMP (inosine monophosphate) dehydrogenase 1 pseudogene 4 [Source:HGNC Symbol;Acc:33959]	1.734259537	1.31727943	11.98134235	0.000537359	0.006425616	0.566455146	0.555709266	1.598401271	1.844482366	1.3928929
ENSG00000112029	FBXO5	F-box protein 5 [Source:HGNC Symbol;Acc:13584]	-1.316567812	3.753514935	11.96169478	0.000543055	0.006481454	4.230629451	4.438368203	2.955212673	2.379562322	3.779126901
ENSG00000056736	IL17RB	interleukin 17 receptor B [Source:HGNC Symbol;Acc:18015]	1.992825516	0.021786624	11.9615197	0.000543106	0.006481454	-0.698419327	-0.464918547	0.778135316	-0.874832399	0.303422373
ENSG00000160326	SLC2A6	solute carrier family 2 (facilitated glucose transporter), member 6 [Source:HGNC Symbol;Acc:11011]	1.331431745	5.546489475	11.94626971	0.000547569	0.006530281	4.525609656	5.03708129	5.50599359	6.486768637	5.405885943
ENSG00000205683	DPF3	D4, zinc and double PHD fingers, family 3 [Source:HGNC Symbol;Acc:17427]	-1.606226114	2.07383636	11.94136922	0.000549011	0.006543035	2.744312733	2.778115078	0.570569237	0.669468625	2.041752268
ENSG00000186594	MIR22HG	MIR22 host gene (non-protein coding) [Source:HGNC Symbol;Acc:28219]	1.933930993	5.961715585	11.92080563	0.000555105	0.006611169	4.70812895	4.550279288	4.899631261	7.38676287	6.012031481
ENSG00000122574	WIPF3	WAS/WASL interacting protein family, member 3 [Source:HGNC Symbol;Acc:22004]	-3.980511944	-0.397910639	11.9180754	0.000555919	0.006612834	1.084982398	0.095631612	-4.621297774	-7.002070102	-1.87334355
ENSG00000243696	RP5-966M1.6	Musculoskeletal embryonic nuclear protein 1 [Source:UniProtKB/TrEMBL;Acc:D6R8Y8]	1.715903909	1.034726574	11.91781057	0.000555998	0.006612834	0.657137645	0.610915408	1.397324829	1.39816257	0.676839958
ENSG00000126215	XRCC3	X-ray repair complementing defective repair in Chinese hamster cells 3 [Source:HGNC Symbol;Acc:12830]	-1.262498441	4.032581858	11.91499136	0.00055684	0.006618365	4.679568409	4.705760662	3.100336608	3.135408114	3.636471986
ENSG00000136404	TM6SF1	transmembrane 6 superfamily member 1 [Source:HGNC Symbol;Acc:11860]	1.670797867	3.790570026	11.91306135	0.000557417	0.006620743	2.32292636	3.247479563	3.551857933	4.547804143	4.270278622
ENSG00000166578	IQCD	IQ motif containing D [Source:HGNC Symbol;Acc:25168]	2.081082358	-0.102344917	11.89657733	0.000562372	0.006675076	-0.555769858	-0.932821174	-0.99931534	0.434658742	0.415212929
ENSG00000111684	LPCAT3	lysophosphatidylcholine acyltransferase 3 [Source:HGNC Symbol;Acc:30244]	-1.176835931	6.100752784	11.89296703	0.000563463	0.006683508	6.671554818	6.591256372	5.274756428	5.583046766	5.854025822
ENSG00000179304	FAM156B	family with sequence similarity 156, member B [Source:HGNC Symbol;Acc:31962]	1.593392877	1.997641897	11.8913466	0.000563953	0.006684808	1.448792784	1.255368251	2.642776537	2.320413773	1.746389777
ENSG00000143067	ZNF697	zinc finger protein 697 [Source:HGNC Symbol;Acc:32034]	1.943544186	2.023854419	11.88975322	0.000564436	0.006686015	0.769809504	1.355744383	1.582674799	2.8843467	2.435629945
ENSG00000196503	ARL9	ADP-ribosylation factor-like 9 [Source:HGNC Symbol;Acc:23592]	-2.886746522	0.083785571	11.87925757	0.000567626	0.006719264	1.287817526	1.07079906	-7.002070102	-3.360122781	-1.55968627
ENSG00000166965	RCCD1	RCC1 domain containing 1 [Source:HGNC Symbol;Acc:30457]	-1.343174841	3.815047178	11.86218588	0.000572853	0.006769537	4.346439116	4.379972967	3.149202463	2.387817579	3.889710441
ENSG00000112773	FAM46A	family with sequence similarity 46, member A [Source:HGNC Symbol;Acc:18345]	1.218474678	5.450132064	11.86117917	0.000573162	0.006769537	4.792235539	4.578250478	5.589020897	6.137350316	5.590864997
ENSG00000178814	OPLAH	5-oxoprolinase (ATP-hydrolysing) [Source:HGNC Symbol;Acc:8149]	1.524476604	2.713921967	11.86035831	0.000573415	0.006769537	1.546924617	2.107040151	2.942946747	3.211794066	3.063446788
ENSG00000232114	AC018693.5		2.232854806	-0.10034792	11.86132824	0.000573117	0.006769537	-0.555769858	-1.507080648	-0.215316613	0.556835562	0.04992943
ENSG00000106211	HSPB1	heat shock 27kDa protein 1 [Source:HGNC Symbol;Acc:5246]	-1.288681678	4.299382072	11.84970491	0.000576705	0.006797625	4.951516125	4.920372416	3.895241519	2.918936894	3.857086264
ENSG00000134072	CAMK1	calcium/calmodulin-dependent protein kinase I [Source:HGNC Symbol;Acc:1459]	1.407163891	4.688099202	11.84889432	0.000576956	0.006797625	3.520070133	4.296139761	4.700907097	5.517782818	4.681448344

ENSG00000186806	VSIG10L	V-set and immunoglobulin domain containing 10 like [Source:HGNC Symbol;Acc:27111]	1.894418149	0.381858866	11.85132072	0.000576205	0.006797625	-0.094677427	-0.355038393	0.210162819	0.74852395	0.763888603
ENSG00000102524	TNFSF13B	tumor necrosis factor (ligand) superfamily, member 13b [Source:HGNC Symbol;Acc:11929]	1.412710303	6.171633483	11.84326343	0.000578703	0.006813638	4.753366735	5.579745494	5.984184815	6.853787473	6.733283275
ENSG00000141084	RANBP10	RAN binding protein 10 [Source:HGNC Symbol;Acc:29285]	-1.146479281	6.052231423	11.83750781	0.000580495	0.00683015	6.403366644	6.718063829	5.720587303	5.114688341	5.760102512
ENSG00000226174	TEX22	testis expressed 22 [Source:HGNC Symbol;Acc:40026]	1.80926327	0.254566236	11.83091557	0.000582553	0.006845198	0.000762307	0.016063025	0.250534523	0.369457657	0.264147325
ENSG00000235257	AC093415.2		2.069437874	0.16296489	11.83104546	0.000582513	0.006845198	-0.250832646	-0.464918547	-0.82139248	0.962608982	0.303422373
ENSG00000170909	OSCAR	osteoclast associated, immunoglobulin-like receptor [Source:HGNC Symbol;Acc:29960]	1.180007713	5.952270051	11.82429081	0.00058463	0.006865	4.715181607	5.210183925	5.839498562	6.348220457	6.745812048
ENSG00000207652	MIR621	microRNA 621 [Source:HGNC Symbol;Acc:32877]	-1.817519905	1.432625039	11.80651167	0.000590239	0.006926236	2.332130562	2.126114598	0.168628767	-0.244792505	0.872340852
ENSG00000206077	ZDHHC11B	zinc finger, DHHC-type containing 11B [Source:HGNC Symbol;Acc:32962]	1.85300141	0.482472205	11.77238754	0.000601158	0.007049654	-0.094677427	0.095631612	1.098750223	-0.009775356	0.676839958
ENSG00000111331	OAS3	2'-5'-oligoadenylate synthetase 3, 100kDa [Source:HGNC Symbol;Acc:8088]	1.253900634	6.491345239	11.76690136	0.000602933	0.007065743	5.356348278	6.050077988	6.606022427	7.157931605	6.674113089
ENSG00000226507	IPMKP1	inositol polyphosphate multikinase pseudogene 1 [Source:HGNC Symbol;Acc:39354]	2.125628099	0.059175346	11.75782483	0.00060588	0.007095545	-1.350769713	-0.713501401	-0.453950945	0.369457657	0.948644093
ENSG00000254528	RP11-728F11.4		1.843586189	0.511891806	11.75192174	0.000607804	0.007113339	-0.14487434	0.095631612	0.778135316	1.36509501	-0.80787826
ENSG00000049323	LTBP1	latent transforming growth factor beta binding protein 1 [Source:HGNC Symbol;Acc:6714]	-1.590763295	5.253919156	11.74665032	0.000609528	0.007124015	5.471196963	6.197124414	3.315422674	4.968236968	4.97544198
ENSG00000197415	VEPH1	ventricular zone expressed PH domain-containing 1 [Source:HGNC Symbol;Acc:25735]	1.786487494	1.130739875	11.74722144	0.000609341	0.007124015	0.597320328	0.277241192	1.32354911	1.719236363	1.11240278
ENSG00000152939	MARVELD2	MARVEL domain containing 2 [Source:HGNC Symbol;Acc:26401]	-1.914281996	0.934899839	11.74497256	0.000610078	0.007124476	1.920891399	1.976501425	-1.202308962	-2.0191864	-0.257803757
ENSG00000115112	TFCP2L1	transcription factor CP2-like 1 [Source:HGNC Symbol;Acc:17925]	1.70954623	0.87485362	11.74405323	0.00061038	0.007124476	0.254194054	0.836121102	1.467510679	1.224680533	-0.316136847
ENSG00000128266	GNAZ	guanine nucleotide binding protein (G protein), alpha z polypeptide [Source:HGNC Symbol;Acc:4395]	1.780492024	3.923264577	11.74015497	0.000611659	0.00713467	2.455179325	3.327938933	3.403098856	4.990090995	4.143448621
ENSG00000097046	CDC7	cell division cycle 7 [Source:HGNC Symbol;Acc:1745]	-1.208826748	4.49291546	11.7207674	0.000618065	0.007204598	4.975210034	5.141540505	3.948363866	3.090594325	4.431855191
ENSG00000111319	SCNN1A	sodium channel, non-voltage-gated 1 alpha subunit [Source:HGNC Symbol;Acc:10599]	1.900367242	0.142747355	11.70394413	0.000623678	0.007265203	-0.625332215	0.016063025	0.805492218	-0.406691783	0.139468177
ENSG00000166002	SMCO4	single-pass membrane protein with coiled-coil domains 4 [Source:HGNC Symbol;Acc:24810]	1.577684283	4.056883454	11.69427691	0.000626926	0.007298204	2.863368893	3.352442362	3.701339772	4.903497265	4.492587718
ENSG00000007038	PRSS21	protease, serine, 21 (testisin) [Source:HGNC Symbol;Acc:9485]	-1.70208642	2.131996255	11.69118488	0.000627969	0.007302837	3.124027351	2.652185163	1.163613388	-0.146146183	1.802296017
ENSG00000266853	ITM2BP1	integral membrane protein 2B pseudogene 1 [Source:HGNC Symbol;Acc:20919]	1.630634375	1.62689803	11.69062989	0.000628156	0.007302837	1.210071084	0.904001603	2.150470541	1.914722448	1.481174441
ENSG00000230450	NEK2P4	NEK2 pseudogene 4 [Source:HGNC Symbol;Acc:37818]	-2.436821221	-0.066932869	11.6839185	0.000630426	0.007324376	0.848890306	0.715367718	-3.766908349	-2.97427061	-0.257803757
ENSG00000164344	KLKB1	kallikrein B, plasma (Fletcher factor) 1 [Source:HGNC Symbol;Acc:6371]	1.828989092	-0.068405991	11.67274847	0.000634222	0.007363608	-0.250832646	-0.204475541	0.365283811	0.03296005	-1.083770229
ENSG00000199133	MIRLET7D	microRNA let-7d [Source:HGNC Symbol;Acc:31481]	1.886608669	-0.107937043	11.66375473	0.000637296	0.007394401	-0.625332215	-1.289265519	0.328039018	0.798919003	-1.70800759
ENSG00000104626	ERI1	exoribonuclease 1 [Source:HGNC Symbol;Acc:23994]	-1.134788756	4.692461114	11.65561696	0.00064009	0.007421912	5.148029922	5.328128009	4.177648248	3.683523464	4.486201609
ENSG00000197329	PELI1	pellino E3 ubiquitin protein ligase 1 [Source:HGNC Symbol;Acc:8827]	1.66165435	6.179403435	11.64341709	0.000644301	0.007463239	5.237695108	5.205707923	5.64119881	7.341260267	6.235985773
ENSG00000196787	HIST1H2AG	histone cluster 1, H2ag [Source:HGNC Symbol;Acc:4737]	1.696020579	2.443350659	11.64283163	0.000644504	0.007463239	1.752861822	1.464652754	2.36792042	3.314964122	2.504656975
ENSG00000152042	NBPF11	neuroblastoma breakpoint family, member 11 [Source:HGNC Symbol;Acc:31993]	1.822362399	0.370475473	11.64128223	0.000645041	0.007464535	-0.196880952	0.277241192	0.28980717	0.823471408	0.139468177
ENSG00000114019	AMOTL2	angiomotin like 2 [Source:HGNC Symbol;Acc:17812]	-3.172307694	-0.34379676	11.62435769	0.000650937	0.007527801	0.796654307	0.764888368	-3.766908349	-7.002070102	-3.221241555
ENSG00000171729	TMEM51	transmembrane protein 51 [Source:HGNC Symbol;Acc:25488]	1.805322502	0.281110424	11.62222596	0.000651684	0.007531472	-0.094677427	-0.112176905	0.505333341	0.301169826	0.378903451
ENSG00000205269	TMEM170B	transmembrane protein 170B [Source:HGNC Symbol;Acc:34244]	1.601314774	6.449190567	11.59646545	0.000660773	0.007631492	4.933486777	5.941205894	6.16535218	7.465655792	6.585778961
ENSG00000105835	NAMPT	nicotinamide phosphoribosyltransferase [Source:HGNC Symbol;Acc:30092]	2.515950866	7.208195278	11.59226451	0.000662267	0.007643722	5.266786509	5.557084059	5.264935772	8.649049925	7.738613381

ENSG00000238197	PAXBP1-AS1	PAXBP1 antisense RNA 1 [Source:HGNC Symbol;Acc:39603]	1.549324953	2.112555648	11.58041456	0.000666501	0.007687531	1.361587606	1.551875699	2.5886897	2.475638093	2.098677261
ENSG00000129277	CCL4	chemokine (C-C motif) ligand 4 [Source:HGNC Symbol;Acc:10630]	-1.626244165	4.336704384	11.57264235	0.000669292	0.007708758	5.519334152	4.180008374	3.061151464	3.382617132	4.170253557
ENSG00000149798	CDC42EP2	CDC42 effector protein (Rho GTPase binding) 2 [Source:HGNC Symbol;Acc:16263]	1.881581138	2.812697793	11.57162548	0.000669659	0.007708758	1.608823392	1.809558268	2.1289755	3.883584355	3.194574245
ENSG00000232928	DDX3YP1	DEAD (Asp-Glu-Ala-Asp) box polypeptide 3, Y-linked pseudogene 1 [Source:HGNC Symbol;Acc:42171]	1.934011716	4.168275543	11.57235834	0.000669395	0.007708758	2.824764954	2.825562359	3.089249033	5.198030214	4.918082231
ENSG00000224086	L22NC03-86G7.1		1.92695116	1.071673456	11.56789614	0.000671003	0.007719172	-0.046168491	0.207316148	0.538319988	1.770646721	1.702990823
ENSG00000124098	FAM210B	family with sequence similarity 210, member B [Source:HGNC Symbol;Acc:16102]	-1.114344535	6.170682623	11.5601911	0.000673789	0.00774615	6.424497523	6.854056826	5.706144324	5.482564629	5.939871642
ENSG00000198019	FCGR1B	Fc fragment of IgG, high affinity Ib, receptor (CD64) [Source:HGNC Symbol;Acc:3614]	1.979107621	2.302972455	11.55879943	0.000674294	0.007746877	0.502670228	1.128690501	1.360908506	2.890169632	3.439004866
ENSG00000232801	SDCBPP3	syndecan binding protein (syntenin) pseudogene 3 [Source:HGNC Symbol;Acc:44687]	1.282706747	5.865995958	11.55260112	0.000676545	0.007763791	4.736135045	5.434287602	5.782128645	6.362951298	6.394430056
ENSG00000130489	SCO2	SCO2 cytochrome c oxidase assembly protein [Source:HGNC Symbol;Acc:10604]	1.610339708	5.517662388	11.55231234	0.00067665	0.007763791	4.230629451	4.525351572	4.92479781	6.679398028	5.757453315
ENSG00000214787	MS4A4E	membrane-spanning 4-domains, subfamily A, member 4E [Source:HGNC Symbol;Acc:14284]	1.901045978	2.186255549	11.54909413	0.000677823	0.007772161	0.686141241	1.785765038	2.107155349	2.974805722	2.390748373
ENSG00000101213	PTK6	protein tyrosine kinase 6 [Source:HGNC Symbol;Acc:9617]	1.995743978	0.663764256	11.54179977	0.000680487	0.007797621	-0.489407866	0.438524285	0.602113338	1.55300499	0.139468177
ENSG00000258315	C17orf49	chromosome 17 open reading frame 49 [Source:HGNC Symbol;Acc:28737]	1.706492834	1.594839253	11.54015879	0.000681088	0.007799415	1.040754067	0.740340508	1.855590689	2.436381066	1.044483142
ENSG00000237683	AL627309.1	Uncharacterized protein [Source:UniProtKB/TrEMBL;Acc:R4GN28]	1.799843419	5.975345659	11.52662717	0.000686064	0.007851271	4.70458965	4.30453643	4.932573168	7.115066374	6.633755157
ENSG00000158062	UBXN11	UBX domain protein 11 [Source:HGNC Symbol;Acc:30600]	1.384231936	7.260149725	11.51564559	0.000690129	0.007892643	5.923440551	6.808290873	7.453535717	8.137536399	7.092980759
ENSG00000140287	HDC	histidine decarboxylase [Source:HGNC Symbol;Acc:4855]	-1.813550725	2.906424359	11.51293005	0.000691138	0.007899036	4.230629451	3.100920657	2.150470541	0.497040107	2.006486965
ENSG00000142512	SIGLEC10	sialic acid binding Ig-like lectin 10 [Source:HGNC Symbol;Acc:15620]	1.322972786	6.372407	11.51014156	0.000692175	0.007905747	5.26918452	5.774413599	6.283758522	7.359738805	6.294416672
ENSG00000233387	RP11-342D11.3		1.940259546	0.416776591	11.50833083	0.00069285	0.007908307	-0.14487434	0.016063025	1.566775005	-0.296766077	-0.440394449
ENSG00000185222	WBP5	WW domain binding protein 5 [Source:HGNC Symbol;Acc:30084]	-1.752846537	1.821212806	11.50462968	0.000694231	0.00791892	2.614540713	2.393450112	-0.329705368	0.402426505	1.958084208
ENSG00000129450	SIGLEC9	sialic acid binding Ig-like lectin 9 [Source:HGNC Symbol;Acc:10878]	1.324726802	5.433301422	11.48871448	0.0007002	0.007981831	4.183097611	4.681789027	5.136180506	6.005421395	6.181031271
ENSG00000247570	SDCBPP2	syndecan binding protein (syntenin) pseudogene 2 [Source:HGNC Symbol;Acc:44686]	1.25629111	4.916636888	11.47723298	0.000704539	0.008026081	3.918660435	4.401659971	4.8396951	5.490252556	5.354221754
ENSG00000164821	DEFA4	defensin, alpha 4, corticostatin [Source:HGNC Symbol;Acc:2763]	-2.609575363	1.702475625	11.47333478	0.000706019	0.008037717	2.937609558	1.03087254	-7.002070102	-0.525689211	2.496207062
ENSG00000091651	ORC6	origin recognition complex, subunit 6 [Source:HGNC Symbol;Acc:17151]	-1.707235967	3.699755299	11.47107624	0.000706877	0.008042276	4.580707812	4.30453643	1.534439871	3.03386134	3.305127547
ENSG00000014914	MTMR11	myotubularin related protein 11 [Source:HGNC Symbol;Acc:24307]	1.373425765	5.098287944	11.46926108	0.000707568	0.008044921	4.001759993	4.61753212	5.025659538	6.034631298	5.012946482
ENSG00000263391	AL512428.1		1.738515853	1.973847425	11.46801195	0.000708044	0.008045119	0.874315765	1.255368251	1.881530361	3.216436525	1.090116303
ENSG00000248099	INSL3	insulin-like 3 (Leydig cell) [Source:HGNC Symbol;Acc:6086]	1.877447634	0.617205833	11.45987832	0.00071115	0.008075181	-0.775407551	0.016063025	0.632982468	0.74852395	1.374569317
ENSG00000255240	RP11-142C4.6		2.076247234	0.803570413	11.45413836	0.00071335	0.008094927	0.365960855	-0.408932621	0.250534523	1.55300499	1.198257616
ENSG00000248905	FMN1	formin 1 [Source:HGNC Symbol;Acc:3768]	1.726596872	3.06796527	11.43593383	0.000720373	0.008169342	1.711139862	2.244306154	2.612118495	4.05666439	3.443406881
ENSG00000213928	IRF9	interferon regulatory factor 9 [Source:HGNC Symbol;Acc:6131]	1.347652503	3.238033839	11.42603065	0.000724222	0.008202403	2.560743753	2.69113155	3.622778389	3.854096116	2.956635145
ENSG00000101493	ZNF516	zinc finger protein 516 [Source:HGNC Symbol;Acc:28990]	1.348424935	5.85516196	11.42674758	0.000723943	0.008202403	4.782196375	5.033297242	5.504950982	6.739900509	6.259268388
ENSG00000172889	EGFL7	EGF-like-domain, multiple 7 [Source:HGNC Symbol;Acc:20594]	-1.816037552	3.466528511	11.4207323	0.000726291	0.008220524	4.267078814	3.909778597	0.805492218	2.565713262	3.704069251
ENSG00000183763	TRAIP	TRAF interacting protein [Source:HGNC Symbol;Acc:30764]	-1.480752333	2.395519179	11.41916233	0.000726905	0.008222172	3.053437594	3.247479563	1.304500245	0.74852395	2.018338105
ENSG00000113749	HRH2	histamine receptor H2 [Source:HGNC Symbol;Acc:5183]	1.523853295	4.686695723	11.4177613	0.000727453	0.008223076	3.250774959	4.16394696	4.326707234	5.492168154	5.168612187
ENSG00000116120	FARSB	phenylalanyl-tRNA synthetase, beta subunit [Source:HGNC Symbol;Acc:17800]	-1.183484347	4.31126439	11.40439004	0.000732708	0.008277143	4.944031259	4.920372416	3.803243143	3.178871708	3.896147672

ENSG00000170458	CD14	CD14 molecule [Source:HGNC Symbol;Acc:1628]	1.787302131	8.486211114	11.3877916	0.000739284	0.008346061	6.469506905	7.482074742	7.984612549	9.354563373	9.270023302
ENSG00000165283	STOML2	stomatin (EPB72)-like 2 [Source:HGNC Symbol;Acc:14559]	-1.107483811	5.586969909	11.38407497	0.000740764	0.008357402	6.203470965	5.967221872	5.183866841	4.80213659	5.305482859
ENSG00000046653	GPM6B	glycoprotein M6B [Source:HGNC Symbol;Acc:4461]	-3.11636756	0.134724916	11.37744648	0.000743412	0.008381892	1.084982398	0.925936776	-7.002070102	-3.888428289	0.139468177
ENSG00000175538	KCNE3	potassium voltage-gated channel, Isk-related family, member 3 [Source:HGNC Symbol;Acc:6243]	1.279464186	5.95926261	11.37448975	0.000744597	0.008389856	4.68316955	5.235654878	5.686046143	6.534023815	6.690871289
ENSG00000123989	CHPF	chondroitin polymerizing factor [Source:HGNC Symbol;Acc:24291]	-1.546143698	3.366052632	11.3724966	0.000745396	0.008392379	4.362249789	3.651289677	2.766259878	1.596392694	3.057715783
ENSG00000173597	SULT1B1	sulfotransferase family, cytosolic, 1B, member 1 [Source:HGNC Symbol;Acc:17845]	1.356267369	5.388861944	11.37154851	0.000745777	0.008392379	3.701474281	4.884416301	5.389928924	5.642901371	6.225161062
ENSG00000164649	CDCA7L	cell division cycle associated 7-like [Source:HGNC Symbol;Acc:30777]	-1.212128068	5.132799245	11.36961557	0.000746553	0.008395735	5.564941291	5.379702666	4.497880644	3.490045265	5.710743664
ENSG00000142089	IFITM3	interferon induced transmembrane protein 3 [Source:HGNC Symbol;Acc:5414]	1.737596547	5.321421595	11.36788342	0.00074725	0.008398189	3.740019116	3.821846517	4.011394951	6.057451373	6.483154982
ENSG00000272345	RP1-30M3.5		1.871457217	0.892400204	11.36472272	0.000748523	0.00840711	-0.555769858	0.610915408	1.379231566	1.206127397	0.923655414
ENSG00000197927	C2orf27A	chromosome 2 open reading frame 27A [Source:HGNC Symbol;Acc:25077]	1.762209629	0.47321989	11.35887535	0.000750883	0.008428227	-0.196880952	0.310972527	0.884581662	0.466186565	0.415212929
ENSG00000253422	CTB-47B8.4		-1.906763698	0.630979232	11.35215118	0.000753606	0.008453392	1.806678074	1.434365866	-1.438615935	-1.231517591	-1.18885156
ENSG00000164403	SHROOM1	shroom family member 1 [Source:HGNC Symbol;Acc:24084]	1.765394261	2.97917084	11.34885347	0.000754946	0.008463008	1.668175302	2.813846027	3.186096972	4.268193945	-0.9858258
ENSG00000107282	APBA1	amyloid beta (A4) precursor protein-binding, family A, member 1 [Source:HGNC Symbol;Acc:578]	1.860914503	0.357617944	11.33920526	0.000758878	0.008501661	-0.698419327	0.133828548	0.632982468	0.527247606	0.518960621
ENSG00000267283	AC005306.3		1.39570587	2.847099778	11.33518589	0.000760522	0.008514648	2.42108026	2.287305009	3.575887544	2.924622147	2.578558393
ENSG00000136478	TEX2	testis expressed 2 [Source:HGNC Symbol;Acc:30884]	-1.069733789	5.503599121	11.33199239	0.000761831	0.008523867	5.842018808	6.054440781	4.780611023	4.877351856	5.518650873
ENSG00000168734	PKIG	protein kinase (cAMP-dependent, catalytic) inhibitor gamma [Source:HGNC Symbol;Acc:9019]	-1.436442562	4.149982524	11.32674852	0.000763986	0.008542526	5.082729028	4.409466019	2.886421762	3.336448462	3.986396982
ENSG00000115183	TANC1	tetratricopeptide repeat, ankyrin repeat and coiled-coil containing 1 [Source:HGNC Symbol;Acc:29364]	-1.784137939	1.177498458	11.32163221	0.000766093	0.008560643	2.060341768	1.912023327	-0.740035668	-1.042179808	0.997359302
ENSG00000120889	TNFRSF10B	tumor necrosis factor receptor superfamily, member 10b [Source:HGNC Symbol;Acc:11905]	1.230009473	6.733139045	11.32025923	0.00076666	0.008561525	5.706144815	6.293075228	6.82401577	7.464192677	6.796157949
ENSG00000070785	EIF2B3	eukaryotic translation initiation factor 2B, subunit 3 gamma, 58kDa [Source:HGNC Symbol;Acc:3259]	-1.243565572	3.99099676	11.31734658	0.000767864	0.008569514	4.716939397	4.537869269	3.324906709	3.044343756	3.512071509
ENSG00000139631	CSAD	cysteine sulfinic acid decarboxylase [Source:HGNC Symbol;Acc:18966]	1.092106368	4.972789428	11.30618818	0.000772492	0.00861569	4.373437975	4.576518077	5.461542069	5.269019574	4.884482612
ENSG00000135604	STX11	syntaxin 11 [Source:HGNC Symbol;Acc:11429]	1.602514599	6.435083782	11.30263858	0.00077397	0.008626697	5.129672167	5.808022229	6.00203063	7.533963088	6.52165395
ENSG00000137965	IFI44	interferon-induced protein 44 [Source:HGNC Symbol;Acc:16938]	1.297246477	5.172724419	11.29834862	0.000775761	0.008641166	4.203302211	4.472303462	5.011045961	5.715208396	5.773276013
ENSG00000259493	RP11-621H8.2		-1.443601785	2.706122003	11.28870431	0.000779801	0.008680662	3.317173381	3.50595424	1.360908506	1.493047208	2.487707366
ENSG00000134291	TMEM106C	transmembrane protein 106C [Source:HGNC Symbol;Acc:28775]	-1.25563484	4.813239431	11.28238268	0.000782461	0.008704751	5.389790814	5.282100858	4.4767647	3.49387447	4.696278898
ENSG00000184368	MAP7D2	MAP7 domain containing 2 [Source:HGNC Symbol;Acc:25899]	-1.973383031	1.558980496	11.25840725	0.000792632	0.00881232	2.737401394	2.48494876	0.505333341	-3.360122781	-0.649489103
ENSG00000186818	LILRB4	leukocyte immunoglobulin-like receptor, subfamily B (with TM and ITIM domains), member 4 [Source:HGNC Symbol;Acc:6608]	1.548286453	5.409810205	11.25643692	0.000793474	0.008816096	4.074859969	4.61584628	4.77196939	6.217693964	6.112197499
ENSG00000186074	CD300LF	CD300 molecule-like family member f [Source:HGNC Symbol;Acc:29883]	1.482945578	6.126232557	11.24401481	0.000798802	0.008869681	4.797229045	5.488725746	5.746402261	7.057366875	6.48208562
ENSG00000250384	UBE2CP3	ubiquitin-conjugating enzyme E2C pseudogene 3 [Source:HGNC Symbol;Acc:43552]	-2.610539397	-0.159810486	11.23461196	0.000802859	0.008897846	0.742455703	0.527293245	-3.766908349	-3.888428289	-0.095747566
ENSG00000183207	RUVBL2	RuvB-like AAA ATPase 2 [Source:HGNC Symbol;Acc:10475]	-1.085986696	5.258851502	11.23643789	0.000802069	0.008897846	5.833942089	5.758457548	4.581362878	4.532993113	5.039358506
ENSG00000102032	RENBP	renin binding protein [Source:HGNC Symbol;Acc:9959]	1.308974626	4.357805493	11.23507317	0.000802659	0.008897846	3.458362025	3.873554127	4.336117616	5.130762949	4.416264004
ENSG00000247763	RP11-447H19.1		1.674322924	2.093789811	11.23293266	0.000803586	0.008900282	1.148882041	1.202433699	1.907011864	2.643615717	2.71589062
ENSG00000256751	RP11-695J4.2		1.840877526	0.867768995	11.22096223	0.000808785	0.008952225	-0.196880952	0.583576386	0.570569237	1.206127397	1.428856327

ENSG00000182481	KPNA2	karyopherin alpha 2 (RAG cohort 1, importin alpha 1) [Source:HGNC Symbol;Acc:6395]	-1.557242551	4.492731132	11.21841065	0.000809898	0.008958894	5.311680775	4.994900652	2.604351095	3.886500298	4.306975745
ENSG00000137474	MYO7A	myosin VIIA [Source:HGNC Symbol;Acc:7606]	1.727327348	2.667701928	11.19265714	0.000821217	0.009078374	1.040754067	2.08771013	2.386155201	3.314964122	3.324348684
ENSG00000158186	MRAS	muscle RAS oncogene homolog [Source:HGNC Symbol;Acc:7227]	1.728710043	3.090611889	11.18605378	0.000824144	0.009105006	1.431768352	2.652185163	2.892812798	3.918192614	3.430160334
ENSG00000129195	FAM64A	family with sequence similarity 64, member A [Source:HGNC Symbol;Acc:25483]	-2.548445022	0.500261705	11.18306123	0.000825475	0.009113967	1.229903997	0.904001603	-7.002070102	-2.670141381	1.279288931
ENSG00000166033	HTRA1	HtrA serine peptidase 1 [Source:HGNC Symbol;Acc:9476]	1.843603336	1.034349731	11.17761143	0.000827903	0.009135031	-0.196880952	0.242702272	-0.329705368	0.984547783	2.372397358
ENSG00000101307	SIRPB1	signal-regulatory protein beta 1 [Source:HGNC Symbol;Acc:15928]	1.399486981	6.758402983	11.17338078	0.000829793	0.009146543	5.470155113	6.2077954	6.554045411	7.701865288	6.914239558
ENSG00000179921	GPBAR1	G protein-coupled bile acid receptor 1 [Source:HGNC Symbol;Acc:19680]	1.414674371	4.725886192	11.17294397	0.000829988	0.009146543	3.424338996	4.379972967	4.789201202	5.625553375	4.550866558
ENSG00000261662	RP5-1042I8.7		2.210802973	2.173414056	11.17067251	0.000831005	0.009152003	0.435934254	1.699255595	1.689321579	3.427354029	1.869263524
ENSG00000145428	RNF175	ring finger protein 175 [Source:HGNC Symbol;Acc:27735]	2.104939498	-0.09065371	11.15924557	0.000836139	0.009202773	-1.47369337	0.016063025	0.934997507	-0.874832399	-0.726511138
ENSG00000171643	S100Z	S100 calcium binding protein Z [Source:HGNC Symbol;Acc:30367]	1.573075848	3.216185767	11.15436288	0.000838343	0.009221245	2.082327188	2.825562359	3.186096972	4.094984473	3.114026435
ENSG00000178035	IMPDH2	IMP (inosine 5'-monophosphate) dehydrogenase 2 [Source:HGNC Symbol;Acc:6053]	-1.14942917	6.512641266	11.13722288	0.000846125	0.009295317	7.026881118	6.95151089	6.182408321	5.670231437	6.295025729
ENSG00000197312	DDI2	DNA-damage inducible 1 homolog 2 (S. cerevisiae) [Source:HGNC Symbol;Acc:24578]	-1.075480733	6.275328191	11.13719822	0.000846136	0.009295317	6.675175993	6.876406641	5.847746149	5.520601021	5.995639036
ENSG00000119969	HELLS	helicase, lymphoid-specific [Source:HGNC Symbol;Acc:4861]	-1.28780421	3.391903617	11.13078257	0.000849068	0.00932169	3.948891281	4.028581215	2.456873398	2.045707388	3.434589378
ENSG00000147119	CHST7	carbohydrate (N-acetylglucosamine 6-O) sulfotransferase 7 [Source:HGNC Symbol;Acc:13817]	1.412347171	2.795648774	11.12851876	0.000850105	0.00932724	2.015339542	2.295753104	3.055465682	3.221064093	2.974995322
ENSG00000187796	CARD9	caspase recruitment domain family, member 9 [Source:HGNC Symbol;Acc:16391]	1.521822155	5.422564406	11.12477644	0.000851821	0.009340241	4.118070306	4.779898163	5.072860299	6.452252305	5.58788573
ENSG00000151458	ANKRD50	ankyrin repeat domain 50 [Source:HGNC Symbol;Acc:29223]	1.440811516	4.43211813	11.12144861	0.000853351	0.009351173	3.274841002	3.807148173	4.13245547	5.056326182	5.052385377
ENSG00000133069	TMCC2	transmembrane and coiled-coil domain family 2 [Source:HGNC Symbol;Acc:24239]	-1.479191602	4.067808692	11.11930154	0.00085434	0.009356164	4.047181178	5.176270433	2.627528952	3.130496995	3.989410538
ENSG00000158473	CD1D	CD1d molecule [Source:HGNC Symbol;Acc:1637]	1.30371588	6.011383722	11.10772456	0.000859689	0.009403016	4.749936823	5.419867954	5.876246965	6.890199169	6.232174554
ENSG00000111052	LIN7A	lin-7 homolog A (C. elegans) [Source:HGNC Symbol;Acc:17787]	1.699236096	4.17532201	11.10878829	0.000859196	0.009403016	2.824764954	3.120075649	3.362231416	5.02755662	4.972399074
ENSG00000143184	XCL1	chemokine (C motif) ligand 1 [Source:HGNC Symbol;Acc:10645]	-1.978055671	2.015162898	11.10258579	0.000862074	0.009423235	3.567603998	2.068117593	1.120696904	-0.350682213	-0.095747566
ENSG00000095917	TPSD1	tryptase delta 1 [Source:HGNC Symbol;Acc:14118]	-2.564653212	0.495456168	11.10141296	0.00086262	0.009423329	2.276003363	0.207316148	-2.539967122	-1.854339469	-1.55968627
ENSG00000258645	HSPE1P2	heat shock 10kDa protein 1 pseudogene 2 [Source:HGNC Symbol;Acc:20052]	-1.284526969	3.792839009	11.0962695	0.000865015	0.009443624	4.616932295	4.33149293	3.049757403	2.436381066	3.412306925
ENSG00000135643	KCNMB4	potassium large conductance calcium-activated channel, subfamily M, beta member 4 [Source:HGNC Symbol;Acc:6289]	1.617526963	1.459635246	11.09476057	0.00086572	0.009445437	0.686141241	0.98980953	1.659646938	1.596392694	1.895203721
ENSG00000104972	LILRB1	leukocyte immunoglobulin-like receptor, subfamily B (with TM and ITIM domains), member 1 [Source:HGNC Symbol;Acc:6605]	1.295154699	6.745337261	11.09294758	0.000866566	0.009448803	5.665761812	6.036909884	6.52348126	7.578594849	7.093681056
ENSG00000197249	SERPINA1	serpin peptidase inhibitor, clade A (alpha-1 antiproteinase, antitrypsin), member 1 [Source:HGNC Symbol;Acc:8941]	1.871550032	9.270281566	11.08997192	0.000867958	0.009458102	7.268066693	8.251497483	8.978124508	10.32993031	9.708886256
ENSG00000111445	RFC5	replication factor C (activator 1) 5, 36.5kDa [Source:HGNC Symbol;Acc:9973]	-1.143973595	4.353136733	11.0866593	0.00086951	0.009469135	4.868548297	4.84466512	3.70497617	3.20713662	4.438485891
ENSG00000248592	MEM110-MUSTI	TMEM110-MUSTN1 readthrough [Source:HGNC Symbol;Acc:38834]	1.620333486	1.2244895	11.08326285	0.000871104	0.009480613	0.686141241	0.98980953	1.718398126	1.610570062	0.551948963
ENSG00000116514	RNF19B	ring finger protein 19B [Source:HGNC Symbol;Acc:26886]	1.361731418	5.959513536	11.07592373	0.000874558	0.009512313	5.020038328	5.362715152	5.734887125	6.865657723	6.074381435
ENSG00000151503	NCAPD3	non-SMC condensin II complex, subunit D3 [Source:HGNC Symbol;Acc:28952]	-1.109647464	5.805107392	11.07402493	0.000875454	0.009516163	6.167929236	6.4360694	5.489220774	4.753453877	5.611549358
ENSG00000164181	ELOVL7	ELOVL fatty acid elongase 7 [Source:HGNC Symbol;Acc:26292]	1.69812092	3.07550266	11.0717893	0.000876511	0.009521747	1.711139862	2.784132029	3.055465682	4.064410267	2.730373058

ENSG00000010292	NCAPD2	non-SMC condensin I complex, subunit D2 [Source:HGNC Symbol;Acc:24305]	-1.087445571	6.396979076	11.06928973	0.000877693	0.009528695	6.767543695	6.926908018	5.962322384	5.571315722	6.331700246
ENSG00000269271	CTB-83J4.2		2.196766273	1.737825063	11.06464538	0.000879894	0.009546689	-0.14487434	1.306429105	1.360908506	2.704366551	1.97033799
ENSG00000243708	PLA2G4B	phospholipase A2, group IVB (cytosolic) [Source:HGNC Symbol;Acc:9036]	1.735336571	0.466105312	11.05650982	0.000883763	0.009582747	0.292419846	0.171040209	1.030832979	0.527247606	-0.316136847
ENSG00000163386	NBPF10	neuroblastoma breakpoint family, member 10 [Source:HGNC Symbol;Acc:31992]	1.340524582	6.391283951	11.05342354	0.000885236	0.009592787	5.252314135	5.872272214	6.249930415	7.325499553	6.429801145
ENSG00000149948	HMGA2	high mobility group AT-hook 2 [Source:HGNC Symbol;Acc:5009]	-1.949314952	0.904324314	11.05074448	0.000886516	0.009594813	1.696960037	1.900991143	-2.845623393	-1.336401299	0.615745481
ENSG00000123342	MMP19	matrix metallopeptidase 19 [Source:HGNC Symbol;Acc:7165]	1.927138649	0.143457004	11.0509001	0.000886442	0.009594813	-0.942926472	-0.464918547	-0.66302287	0.335717742	1.090116303
ENSG00000144959	NCEH1	neutral cholesterol ester hydrolase 1 [Source:HGNC Symbol;Acc:29260]	-1.168508737	4.936281678	11.04882299	0.000887435	0.009598845	5.208004997	5.666176731	3.907918014	4.250195492	4.954005614
ENSG00000180596	HIST1H2BC	histone cluster 1, H2bc [Source:HGNC Symbol;Acc:4757]	2.347859433	0.744311765	11.04570103	0.000888931	0.009609102	0.254194054	-1.191564836	0.210162819	1.732261721	0.997359302
ENSG00000183723	CMTM4	CKLF-like MARVEL transmembrane domain containing 4 [Source:HGNC Symbol;Acc:19175]	1.489809226	3.538259775	11.04257035	0.000890433	0.009619419	2.446729819	3.03180153	3.447189363	4.352857805	3.681886894
ENSG00000140749	IGSF6	immunoglobulin superfamily, member 6 [Source:HGNC Symbol;Acc:5953]	1.358861754	5.604767633	11.0398012	0.000891764	0.009627873	4.141819186	5.038340436	5.388798938	6.154385947	6.311373824
ENSG00000171262	FAM98B	family with sequence similarity 98, member B [Source:HGNC Symbol;Acc:26773]	-1.162915081	4.510488931	11.03839253	0.000892442	0.009629269	4.876434106	5.067001583	3.876015453	3.080443796	4.789130606
ENSG00000170866	LILRA3	leukocyte immunoglobulin-like receptor, subfamily A (without TM domain), member 3 [Source:HGNC Symbol;Acc:6604]	1.159637605	5.783740691	11.03398291	0.000894567	0.009646272	4.763607814	5.460824645	6.103327111	6.37234231	5.710743664
ENSG00000091262	ABCC6	ATP-binding cassette, sub-family C (CFTR/MRP), member 6 [Source:HGNC Symbol;Acc:57]	1.765976354	2.38742371	11.03188537	0.00089558	0.009651265	1.361587606	1.773719635	1.980862385	3.439318024	2.3444256
ENSG00000258546	RP11-713N11.3		-1.925871988	0.693660222	11.02954127	0.000896713	0.009657548	1.752861822	1.494316873	-2.073364206	-1.042179808	-0.201737898
ENSG00000113494	PRLR	prolactin receptor [Source:HGNC Symbol;Acc:9446]	2.476969388	1.672027921	11.02084412	0.00090093	0.009697016	-0.250832646	0.968831255	-0.010608983	3.065082826	1.788521245
ENSG00000140932	CMTM2	CKLF-like MARVEL transmembrane domain containing 2 [Source:HGNC Symbol;Acc:19173]	2.01232673	1.377038458	11.01511515	0.000903719	0.009721072	-0.250832646	0.947543428	1.415193986	2.320413773	1.177268129
ENSG00000211904	IGHJ2	immunoglobulin heavy joining 2 [Source:HGNC Symbol;Acc:5534]	-1.844281609	1.810956252	11.01053232	0.000905956	0.009735449	2.973344712	1.955327308	-1.097244829	1.770646721	0.706443614
ENSG00000230373	GOLGA6L5P	golgin A6 family-like 5, pseudogene [Source:HGNC Symbol;Acc:30472]	1.915585007	0.067172929	11.01010559	0.000906165	0.009735449	-0.306880589	-0.932821174	-0.10933503	0.402426505	0.485200289
ENSG00000270878	CTD-2302E22.5		-1.67352629	1.751195636	11.00524845	0.000908543	0.009750106	2.730456787	2.361613919	0.6928072	-0.46496394	1.279288931
ENSG00000111452	GPR133	G protein-coupled receptor 133 [Source:HGNC Symbol;Acc:19893]	1.439988364	2.840481269	11.00504977	0.00090864	0.009750106	1.79341104	2.492318033	3.170400428	3.579252501	2.453198788
ENSG00000103381	CPPED1	calcineurin-like phosphoesterase domain containing 1 [Source:HGNC Symbol;Acc:25632]	1.392126724	7.013560362	10.99969504	0.000911269	0.009772342	5.660290985	6.314020098	6.782026379	7.994518863	7.234006661
ENSG00000105352	CEACAM4	carcinoembryonic antigen-related cell adhesion molecule 4 [Source:HGNC Symbol;Acc:1816]	1.545137215	4.412210158	10.9880443	0.000917015	0.009827963	3.042264405	3.419690528	3.66819303	5.143007294	5.293341295
ENSG00000180509	KCNE1	potassium voltage-gated channel, Isk-related family, member 1 [Source:HGNC Symbol;Acc:6240]	1.882991385	2.302816129	10.98424826	0.000918895	0.009842105	1.37945544	1.255368251	1.613958159	3.239427344	2.786891356
ENSG00000267190	AC002314.4		-2.308960593	0.021373604	10.9793672	0.000921319	0.009862044	0.899300886	0.859104642	-4.621297774	-2.419096061	-0.201737898
ENSG00000020577	SAMD4A	sterile alpha motif domain containing 4A [Source:HGNC Symbol;Acc:23023]	1.49690189	2.608275495	10.97572532	0.000923131	0.009875422	1.945089405	1.878670417	2.752269159	3.419322554	2.390748373
ENSG00000204394	VARS	valyl-tRNA synthetase [Source:HGNC Symbol;Acc:12651]	-1.092214125	6.489549719	10.96280131	0.000929592	0.009932431	7.025108654	6.976686723	6.085303912	5.794968159	6.116338743
ENSG00000119922	IFIT2	interferon-induced protein with tetratricopeptide repeats 2 [Source:HGNC Symbol;Acc:5409]	1.326258894	4.449622877	10.96342714	0.000929278	0.009932431	3.55586655	3.396312155	4.077409057	4.887575893	5.330652314
ENSG00000183527	PSMG1	proteasome (prosome, macropain) assembly chaperone 1 [Source:HGNC Symbol;Acc:3043]	-1.188839572	4.425551239	10.95910279	0.000931449	0.009946222	5.149332295	4.899738006	3.626618026	3.683523464	4.054164368
ENSG00000103184	SEC14L5	SEC14-like 5 (S. cerevisiae) [Source:HGNC Symbol;Acc:29032]	2.391813413	1.700438113	10.95321336	0.000934414	0.009965761	-0.698419327	1.418980604	1.225685365	3.023302203	1.11240278
ENSG00000237892	KLF7-IT1	KLF7 intronic transcript 1 (non-protein coding) [Source:HGNC Symbol;Acc:41355]	2.757629651	0.462717819	10.9540269	0.000934004	0.009965761	-0.306880589	-1.191564836	-0.99931534	1.914722448	0.095393329
ENSG00000187997	C17orf99	chromosome 17 open reading frame 99 [Source:HGNC Symbol;Acc:34490]	-2.057387787	0.826485302	10.93484205	0.000943725	0.010058954	1.448792784	2.116608898	-1.721358966	-2.97427061	0.04992943

ENSG00000126458	RRAS	related RAS viral (r-ras) oncogene homolog [Source:HGNC Symbol;Acc:10447]	1.260952799	4.083731339	10.92894635	0.000946733	0.010084892	3.118718315	3.621355247	4.268918289	4.640538067	4.270278622
ENSG00000132535	DLG4	discs, large homolog 4 (Drosophila) [Source:HGNC Symbol;Acc:2903]	1.344618175	4.803298708	10.92008387	0.000951273	0.010114838	3.672780374	4.37599461	4.618470392	5.519662232	5.144482599
ENSG00000124104	SNX21	sorting nexin family member 21 [Source:HGNC Symbol;Acc:16154]	1.674537121	3.01801849	10.92020255	0.000951212	0.010114838	1.79341104	2.226741239	2.548775847	3.889410359	3.512071509
ENSG00000262180	OCLM	oculomedin [Source:HGNC Symbol;Acc:8103]	1.831056214	0.464240248	10.9216005	0.000950495	0.010114838	-0.306880589	0.133828548	0.805492218	1.12943269	-0.376928291
ENSG00000204315	FKBPL	FK506 binding protein like [Source:HGNC Symbol;Acc:13949]	-1.608095356	2.14105388	10.90834389	0.000957321	0.010172981	3.02534068	2.865836059	0.328039018	1.048430793	1.580441325
ENSG00000252759	Y_RNA	Y RNA [Source:RFAM;Acc:RF00019]	1.74784377	0.897458356	10.90407225	0.000959531	0.010190299	0.254194054	0.016063025	0.778135316	2.056104393	-0.095747566
ENSG00000233280	CRYBG3	Beta/gamma crystallin domain-containing protein 3; cDNA FLJ60082, weakly similar to Uro-adherence factor A [Source:UniProtKB/TrEMBL;Acc:B4DLE8]	1.50016771	2.685394288	10.89272869	0.000965425	0.010246696	1.46561866	2.353543875	2.918097532	3.059925941	3.02284327
ENSG00000197459	HIST1H2BH	histone cluster 1, H2bh [Source:HGNC Symbol;Acc:4755]	1.842491473	0.195369543	10.88185293	0.00097111	0.010300812	-0.306880589	-0.355038393	0.250534523	0.894716898	-0.316136847
ENSG00000159335	PTMS	parathymosin [Source:HGNC Symbol;Acc:9629]	-1.319060254	3.595953351	10.87844489	0.000972899	0.010313552	4.104705577	4.251237133	2.37706662	2.848906753	3.524590078
ENSG00000120756	PLS1	plastin 1 [Source:HGNC Symbol;Acc:9090]	-1.64244404	2.049445746	10.87129685	0.000976661	0.010347186	2.778381342	2.916017525	1.120696904	-0.406691783	1.612068369
ENSG00000166831	RBPMS2	RNA binding protein with multiple splicing 2 [Source:HGNC Symbol;Acc:19098]	-1.678261226	1.600668659	10.85873264	0.00098331	0.01041134	2.438230535	2.632310473	-0.99931534	0.114810932	0.706443614
ENSG00000161996	WDR90	WD repeat domain 90 [Source:HGNC Symbol;Acc:26960]	-1.092571921	4.673913608	10.85645204	0.000984521	0.010417886	5.163581333	5.183117208	3.996635421	3.871861288	4.618607854
ENSG00000164402	Sep-08	septin 8 [Source:HGNC Symbol;Acc:16511]	-1.151726426	4.646295662	10.85426262	0.000985686	0.010423927	5.283489476	5.364724096	4.340799889	3.579252501	3.647960442
ENSG00000233527	AC092295.7		1.641393933	1.145150819	10.84396497	0.000991183	0.010475745	0.435934254	0.859104642	1.517996407	1.43048915	1.044483142
ENSG00000153904	DDAH1	dimethylarginine dimethylaminohydrolase 1 [Source:HGNC Symbol;Acc:2715]	-2.80635955	0.07471912	10.83478545	0.000996109	0.010521472	1.37945544	0.836121102	-3.766908349	-4.730697034	-1.083770229
ENSG00000155380	SLC16A1	solute carrier family 16 (monocarboxylate transporter), member 1 [Source:HGNC Symbol;Acc:10922]	-1.209816316	4.205879147	10.82299323	0.001002473	0.010575969	4.686761725	4.746390666	3.611197783	2.697741416	4.37076624
ENSG00000182272	B4GALNT4	beta-1,4-N-acetyl-galactosaminyl transferase 4 [Source:HGNC Symbol;Acc:26315]	1.850898613	0.149464621	10.82347048	0.001002215	0.010575969	-0.046168491	-0.713501401	0.570569237	0.434658742	-0.201737898
ENSG00000135077	HAVCR2	hepatitis A virus cellular receptor 2 [Source:HGNC Symbol;Acc:18437]	1.326029577	4.858546794	10.81669044	0.001005891	0.01060566	3.995980462	4.073491918	4.526933176	5.486413713	5.463369879
ENSG00000232229	LINC00865	long intergenic non-protein coding RNA 865 [Source:HGNC Symbol;Acc:45170]	1.783506882	0.941369488	10.8082026	0.001010514	0.010647999	0.000762307	-0.523165112	0.832340008	0.229488395	2.247064062
ENSG00000069869	NEDD4	neural precursor cell expressed, developmentally down-regulated 4, E3 ubiquitin protein ligase [Source:HGNC Symbol;Acc:7727]	-1.215184929	3.909590892	10.80690003	0.001011225	0.010649102	4.44729995	4.4852879	3.232187228	2.768996501	3.850471957
ENSG00000105711	SCN1B	sodium channel, voltage-gated, type I, beta subunit [Source:HGNC Symbol;Acc:10586]	1.896652801	1.833414597	10.80091596	0.001014499	0.010677175	0.686141241	1.109650305	1.304500245	2.842914522	2.041752268
ENSG00000237886	RP11-611D20.2		1.981931485	0.22348403	10.79828404	0.001015942	0.010685959	-0.365194284	-1.394066322	0.210162819	0.402426505	0.973207307
ENSG00000108272	DHRS11	dehydrogenase/reductase (SDR family) member 11 [Source:HGNC Symbol;Acc:28639]	-1.347694049	2.838231888	10.78869614	0.001021218	0.010735018	3.520070133	3.621355247	2.004662384	1.665926088	2.164148811
ENSG00000137124	ALDH1B1	aldehyde dehydrogenase 1 family, member B1 [Source:HGNC Symbol;Acc:407]	-1.322282673	3.508291512	10.77833759	0.001026949	0.0107888	4.225699209	4.031113272	2.949092746	2.203686226	3.204982016
ENSG00000100003	SEC14L2	SEC14-like 2 (S. cerevisiae) [Source:HGNC Symbol;Acc:10699]	-1.659371903	2.55745106	10.77506002	0.001028769	0.010801457	3.420029142	3.494990685	2.016416749	-0.099242291	1.11240278
ENSG00000149636	DSN1	DSN1, MIS12 kinetochore complex component [Source:HGNC Symbol;Acc:16165]	-1.178609711	4.212537693	10.77282299	0.001030013	0.010801599	4.802205327	4.702587364	3.599523466	2.985724478	4.204371626
ENSG00000131042	LILRB2	leukocyte immunoglobulin-like receptor, subfamily B (with TM and ITIM domains), member 2 [Source:HGNC Symbol;Acc:6606]	1.543979777	7.823739621	10.77373447	0.001029506	0.010801599	6.031212219	7.021943625	7.62432399	8.741130988	8.28514159
ENSG00000213397	HAUS7	HAUS augmin-like complex, subunit 7 [Source:HGNC Symbol;Acc:32979]	1.673473257	0.578941977	10.76619963	0.001033705	0.01083385	0.174562846	0.583576386	0.721814282	0.773941511	0.264147325
ENSG00000215784	FAM72D	family with sequence similarity 72, member D [Source:HGNC Symbol;Acc:33593]	-1.749421966	1.501037318	10.75397482	0.001040556	0.01089914	2.446729819	2.244306154	-1.202308962	-0.053815412	1.177268129
ENSG00000203760	CENPW	centromere protein W [Source:HGNC Symbol;Acc:21488]	-1.696417548	2.651478706	10.75273127	0.001041255	0.010899963	3.449931193	3.286151722	0.328039018	1.679439471	2.578558393
ENSG00000175155	YPEL2	yippee-like 2 (Drosophila) [Source:HGNC Symbol;Acc:18326]	1.07294899	6.000356277	10.75043139	0.00104255	0.010907013	5.10168997	5.60290773	6.237429934	6.480027647	6.173101579
ENSG00000112339	HBS1L	HBS1-like (S. cerevisiae) [Source:HGNC Symbol;Acc:4834]	-1.092087004	5.517285558	10.7346802	0.001051462	0.010980613	6.139376066	5.892604324	4.878855169	4.94044551	5.273699717

ENSG00000136689	IL1RN	interleukin 1 receptor antagonist [Source:HGNC Symbol;Acc:6000]	1.419283082	5.286900606	10.73508049	0.001051234	0.010980613	4.03877292	4.688220509	4.869164352	6.049667943	5.839068896
ENSG00000146054	TRIM7	tripartite motif containing 7 [Source:HGNC Symbol;Acc:16278]	1.65737074	2.427539459	10.73533659	0.001051089	0.010980613	1.018120824	1.711936244	2.096120255	3.007317163	3.173530708
ENSG00000183066	WBP2NL	WBP2 N-terminal like [Source:HGNC Symbol;Acc:28389]	1.721788053	0.353005245	10.72801896	0.001055254	0.011013661	0.046214424	0.056395802	0.9100098	0.265774258	-0.045536284
ENSG00000163823	CCR1	chemokine (C-C motif) receptor 1 [Source:HGNC Symbol;Acc:1602]	1.455412193	6.273292966	10.7226205	0.001058337	0.011033042	4.276645101	5.249855047	5.557186035	6.759516999	7.43996047
ENSG00000120708	TGFBI	transforming growth factor, beta-induced, 68kDa [Source:HGNC Symbol;Acc:11771]	1.483615092	8.28432839	10.72256668	0.001058368	0.011033042	6.614703752	7.500464188	8.217246795	9.071306245	8.794252217
ENSG00000162873	KLHDC8A	kelch domain containing 8A [Source:HGNC Symbol;Acc:25573]	-3.053577844	-0.058731146	10.71493316	0.001062743	0.011058953	0.995126841	0.764888368	-7.002070102	-3.888428289	-0.440394449
ENSG00000115414	FN1	fibronectin 1 [Source:HGNC Symbol;Acc:3778]	-1.976650548	1.24160452	10.7158935	0.001062192	0.011058953	2.512979527	1.94462256	-3.233942493	-0.350682213	0.095393329
ENSG00000169247	SH3TC2	SH3 domain and tetratricopeptide repeats 2 [Source:HGNC Symbol;Acc:29427]	-1.514835893	2.565684227	10.71564466	0.001062334	0.011058953	3.250774959	3.538353702	0.884581662	1.719236363	1.580441325
ENSG00000238975	snoU13	Small nucleolar RNA U13 [Source:RFAM;Acc:RF01210]	1.702392337	0.665338211	10.71174677	0.001064575	0.011068143	-0.046168491	-0.068151468	0.9100098	1.523337554	-0.045536284
ENSG00000114923	SLC4A3	solute carrier family 4 (anion exchanger), member 3 [Source:HGNC Symbol;Acc:11029]	1.765975619	1.247343401	10.71120395	0.001064887	0.011068143	-0.094677427	0.836121102	1.053829154	1.39816257	2.064792492
ENSG00000251429	RP11-597D13.7		2.017657096	0.535514838	10.70868342	0.001066339	0.011076673	-0.306880589	0.016063025	0.081791624	1.538247531	0.223773082
ENSG00000125089	SH3TC1	SH3 domain and tetratricopeptide repeats 1 [Source:HGNC Symbol;Acc:26009]	1.17576481	6.286964897	10.70319286	0.001069508	0.011103024	5.323266482	5.82563175	6.292244141	6.991180562	6.456180224
ENSG00000161981	SNRNP25	small nuclear ribonucleoprotein 25kDa (U11/U12) [Source:HGNC Symbol;Acc:14161]	-1.220174868	4.251546877	10.69942679	0.001071687	0.011112505	4.898287778	4.713663503	3.149202463	3.696873774	4.091104988
ENSG00000234719	RP11-166B2.1	Putative NPIP-like protein LOC729978 [Source:UniProtKB/Swiss-Prot;Acc:A6NJ64]	1.834975226	0.209731215	10.70046892	0.001071084	0.011112505	0.000762307	-0.355038393	-0.10933503	0.556835562	0.415212929
ENSG00000181982	CCDC149	coiled-coil domain containing 149 [Source:HGNC Symbol;Acc:25405]	1.59932427	3.923866516	10.6909553	0.001076606	0.011156917	2.237334199	3.340242669	3.579853854	4.726042594	4.481928444
ENSG00000123685	BATF3	basic leucine zipper transcription factor, ATF-like 3 [Source:HGNC Symbol;Acc:28915]	1.553209517	1.903153812	10.67814675	0.001084086	0.011227807	1.084982398	1.387708937	2.263288494	2.460063232	1.760570749
ENSG00000163625	WDFY3	WD repeat and FYVE domain containing 3 [Source:HGNC Symbol;Acc:20751]	1.459252401	5.733176228	10.67568928	0.001085527	0.011236104	4.175447334	4.994900652	5.190367014	6.614928733	6.343524541
ENSG00000182351	CRIP1P4	cysteine-rich protein 1 (intestinal) pseudogene 4 [Source:HGNC Symbol;Acc:44519]	1.041748439	5.830580998	10.66970658	0.001089044	0.011252599	5.277546306	5.458945245	6.102638058	6.37078138	5.644422022
ENSG00000229124	VIM-AS1	VIM antisense RNA 1 [Source:HGNC Symbol;Acc:44879]	1.175118804	4.400818272	10.66990488	0.001088927	0.011252599	3.936874852	3.907024163	4.677037216	4.950233534	4.21213175
ENSG00000167850	CD300C	CD300c molecule [Source:HGNC Symbol;Acc:19320]	1.30558431	4.849871622	10.66988295	0.00108894	0.011252599	3.814150459	4.543200925	4.943388747	5.790304926	4.349814582
ENSG00000225119	LINC00999	long intergenic non-protein coding RNA 999 [Source:HGNC Symbol;Acc:38537]	1.746356002	0.133473369	10.66805165	0.001090019	0.011256046	-0.094677427	-0.157588231	0.28980717	0.466186565	-0.376928291
ENSG00000178789	CD300LB	CD300 molecule-like family member b [Source:HGNC Symbol;Acc:30811]	1.48060749	5.625944714	10.66480155	0.001091936	0.011269213	4.041581121	4.912154033	5.096586211	6.452744229	6.290146058
ENSG00000107551	RASSF4	Ras association (RalGDS/AF-6) domain family member 4 [Source:HGNC Symbol;Acc:20793]	1.392071265	7.244213442	10.65633557	0.001096945	0.01131426	6.030505619	6.612968907	7.050627766	8.239413288	7.319408458
ENSG00000070081	NUCB2	nucleobindin 2 [Source:HGNC Symbol;Acc:8044]	-1.296039389	6.461040691	10.65457658	0.001097989	0.011318375	6.854751	7.010761799	6.21847375	4.944648512	6.495925879
ENSG00000169116	PARM1	prostate androgen-regulated mucin-like protein 1 [Source:HGNC Symbol;Acc:24536]	-1.395648978	3.557213739	10.65121779	0.001099985	0.011330623	4.438804034	4.152364049	3.138485523	1.856429106	2.737560122
ENSG00000137700	SLC37A4	solute carrier family 37 (glucose-6-phosphate transporter), member 4 [Source:HGNC Symbol;Acc:4061]	-1.319318941	4.931569185	10.65040551	0.001100468	0.011330623	5.499046223	5.622349393	4.709983676	3.399046051	4.398236712
ENSG00000169026	MFSD7	major facilitator superfamily domain containing 7 [Source:HGNC Symbol;Acc:26177]	1.62064323	3.550140033	10.64787976	0.001101972	0.011339456	2.629549878	2.66528446	3.211884232	4.643988414	3.52042928
ENSG00000132694	ARHGEF11	Rho guanine nucleotide exchange factor (GEF) 11 [Source:HGNC Symbol;Acc:14580]	1.284026615	6.387132757	10.63436925	0.001110051	0.011409222	4.954499232	5.959931408	6.3971188	7.099129185	6.694569045
ENSG00000248971	KRT8P46	keratin 8 pseudogene 46 [Source:HGNC Symbol;Acc:39880]	1.866820459	0.836169471	10.63445111	0.001110002	0.011409222	0.000762307	0.016063025	0.505333341	1.582074621	1.155968757
ENSG00000164134	NAA15	N(alpha)-acetyltransferase 15, NatA auxiliary subunit [Source:HGNC Symbol;Acc:30782]	-1.050747041	5.790595242	10.62434434	0.001116085	0.011464528	6.322548783	6.25891781	5.31099974	5.139344889	5.489147641
ENSG00000130449	ZSWIM6	zinc finger, SWIM-type containing 6 [Source:HGNC Symbol;Acc:29316]	1.320060987	5.403093129	10.61678179	0.001120659	0.011504779	4.288514442	4.831651785	5.174717113	6.253709458	5.668124032

ENSG00000185339	TCN2	transcobalamin II [Source:HGNC Symbol;Acc:11653]	1.504428169	3.884944158	10.60211234	0.001129585	0.011589638	3.250774959	3.221109942	3.733742023	4.910676573	3.503665038
ENSG00000196411	EPHB4	EPH receptor B4 [Source:HGNC Symbol;Acc:3395]	-1.227596863	4.358535145	10.60087577	0.001130341	0.011590618	4.97374051	4.970043401	3.975715067	2.848906753	4.051283214
ENSG00000090376	IRAK3	interleukin-1 receptor-associated kinase 3 [Source:HGNC Symbol;Acc:17020]	1.351819376	7.03534619	10.59800244	0.001132099	0.011601867	5.643752917	6.33926996	6.761655956	7.733586467	7.667487717
ENSG00000272658	LTB4R2	Leukotriene B4 receptor 2 [Source:UniProtKB/Swiss-Prot;Acc:Q9NPC1]	1.902875662	0.399418162	10.59395867	0.001134578	0.011620487	-0.555769858	0.095631612	0.328039018	1.296595379	-0.257803757
ENSG00000164713	BRI3	brain protein I3 [Source:HGNC Symbol;Acc:1109]	1.271290373	5.766619815	10.58906178	0.001137587	0.011644512	4.748218804	5.257443791	5.57418573	6.599906329	5.95767589
ENSG00000259065	RP5-1021I20.1		1.748463369	0.070312138	10.58415874	0.001140608	0.011668631	-0.046168491	-0.252938016	0.832340008	0.074465887	-1.55968627
ENSG00000104081	BMF	Bcl2 modifying factor [Source:HGNC Symbol;Acc:24132]	1.148465523	5.838126015	10.57649739	0.001145345	0.011703448	5.074526	5.240039066	5.79327425	6.467911261	6.135508911
ENSG00000258082	RP11-443B7.3		2.381847997	-0.349673857	10.57676067	0.001145181	0.011703448	-1.132483902	-1.394066322	-2.287883461	0.58582888	0.139468177
ENSG00000242068	RP11-259P15.4		1.160278881	4.642718441	10.57224575	0.001147982	0.011723572	3.966730322	4.235953559	4.652765747	5.02755662	5.017382202
ENSG00000114270	COL7A1	collagen, type VII, alpha 1 [Source:HGNC Symbol;Acc:2214]	2.000903297	1.112492688	10.56427872	0.00115294	0.011767363	0.848890306	0.133828548	0.505333341	2.106994346	0.923655414
ENSG00000186704	DTX2P1	deltex homolog 2 pseudogene 1 [Source:HGNC Symbol;Acc:42352]	1.613931829	0.889139041	10.55778499	0.001156998	0.011801913	0.435934254	0.789025519	1.225685365	1.314027603	0.095393329
ENSG00000188610	FAM72B	family with sequence similarity 72, member B [Source:HGNC Symbol;Acc:24805]	-1.632790253	2.086938879	10.55116895	0.001161146	0.011837354	2.811663962	2.684713061	-0.390491059	0.61425099	2.381602043
ENSG00000113790	EHHADH	enoyl-CoA, hydratase/3-hydroxyacyl CoA dehydrogenase [Source:HGNC Symbol;Acc:3247]	-1.495172563	2.368007807	10.53994015	0.001168222	0.011902576	3.206417789	3.026735387	1.517996407	0.369457657	1.994537666
ENSG00000163191	S100A11	S100 calcium binding protein A11 [Source:HGNC Symbol;Acc:10488]	1.302568413	6.940479616	10.52933628	0.001174944	0.01196412	5.605359776	6.386010826	6.930744714	7.691482051	7.255757502
ENSG00000011105	TSPAN9	tetraspanin 9 [Source:HGNC Symbol;Acc:21640]	1.700454024	2.755164063	10.52449213	0.001178028	0.011988567	1.106598279	2.278807152	2.56487414	3.686872641	2.938038295
ENSG00000079785	DDX1	DEAD (Asp-Glu-Ala-Asp) box helicase 1 [Source:HGNC Symbol;Acc:2734]	-1.067850109	6.192287567	10.52237224	0.00117938	0.011995373	6.694485599	6.697887453	5.837843371	5.265663847	5.960749975
ENSG00000170006	TMEM154	transmembrane protein 154 [Source:HGNC Symbol;Acc:26489]	1.128106793	6.624922879	10.51138278	0.001186414	0.012059933	5.7480094	6.169234625	6.753777709	7.251940832	6.743582732
ENSG00000146410	MTFR2	mitochondrial fission regulator 2 [Source:HGNC Symbol;Acc:21115]	-1.633137218	1.770123553	10.50239991	0.001192196	0.01211169	2.716466474	2.278807152	-0.010608983	-0.099242291	1.774613685
ENSG00000134243	SORT1	sortilin 1 [Source:HGNC Symbol;Acc:11186]	1.280199386	6.372238523	10.50028801	0.001193559	0.012118528	5.028556163	5.864484452	6.226718694	7.0515329	6.851446282
ENSG00000259689	ABCB10P1	ATP-binding cassette, sub-family B (MDR/TAP), member 10 pseudogene 1 [Source:HGNC Symbol;Acc:14114]	-1.439901792	2.359344867	10.49807201	0.001194992	0.012119052	3.031004009	3.203258561	1.120696904	1.27894994	1.746389777
ENSG00000146232	NFKBIE	nuclear factor of kappa light polypeptide gene enhancer in B-cells inhibitor, epsilon [Source:HGNC Symbol;Acc:7799]	1.731373281	5.151562359	10.49849769	0.001194716	0.012119052	4.391160537	3.955816848	4.409269357	6.552500844	4.688882678
ENSG00000268913	AC026806.2		1.622325077	2.27894296	10.48885965	0.001200965	0.012172596	1.32517403	1.965953212	2.243421497	2.860817063	2.479157296
ENSG00000104951	IL4I1	interleukin 4 induced 1 [Source:HGNC Symbol;Acc:19094]	1.590191874	1.192588398	10.4845978	0.001203738	0.012193666	0.566455146	1.050973916	1.566775005	1.348272594	1.06748014
ENSG00000171595	DNAI2	dynein, axonemal, intermediate chain 2 [Source:HGNC Symbol;Acc:18744]	1.970217397	0.140985211	10.47311462	0.001211243	0.012262617	0.174562846	-0.713501401	1.304500245	-0.406691783	-1.70800759
ENSG00000072134	EPN2	epsin 2 [Source:HGNC Symbol;Acc:18639]	-1.276210949	3.47715588	10.46702339	0.001215244	0.012296025	4.021807945	4.314964006	2.905510557	2.240618578	2.807527639
ENSG00000246731	CTD-2514K5.2		1.784949468	1.495579255	10.46173437	0.001218728	0.012324176	-0.046168491	0.947543428	1.534439871	2.076676283	1.933259916
ENSG00000206341	HLA-H	major histocompatibility complex, class I, H (pseudogene) [Source:HGNC Symbol;Acc:4965]	1.600926329	6.104585835	10.45752312	0.00122151	0.012345193	4.782196375	4.734010556	5.016909167	7.036843908	6.977072555
ENSG00000231412	CTC-490G23.2		2.110433443	0.677635661	10.45555556	0.001222811	0.012351238	-1.034594152	0.555709266	1.076464519	1.79568083	-2.832686105
ENSG00000161955	TNFSF13	tumor necrosis factor (ligand) superfamily, member 13 [Source:HGNC Symbol;Acc:11928]	1.405717844	4.320081784	10.4512524	0.001225663	0.012372926	3.058991905	3.690259922	3.939130343	5.079445529	4.868203525
ENSG00000072952	MRVI1	murine retrovirus integration site 1 homolog [Source:HGNC Symbol;Acc:7237]	1.834479205	3.600597615	10.4422311	0.001231664	0.012426358	1.32517403	2.995959146	3.094803471	4.436811325	4.299710617
ENSG00000161929	SCIMP	SLP adaptor and CSK interacting membrane protein [Source:HGNC Symbol;Acc:33504]	1.228378558	6.554607275	10.43478854	0.001236637	0.01246303	5.631689742	5.960595701	6.436476474	7.352902946	6.752035869
ENSG00000153208	MERTK	c-mer proto-oncogene tyrosine kinase [Source:HGNC Symbol;Acc:7027]	1.462964856	3.946610331	10.43466642	0.001236719	0.01246303	3.013946887	3.376536553	3.754946158	4.94044551	3.82032647
ENSG00000255031	RP11-802E16.3		1.335981291	4.376726963	10.43297587	0.001237851	0.012467286	3.424338996	3.845056765	4.198435311	5.231670152	4.494710155
ENSG00000215022	RP1-257A7.4		1.804241269	1.078564829	10.43083508	0.001239287	0.012474589	0.401371743	0.789025519	0.75024962	1.926103707	0.791775511
ENSG00000188643	S100A16	S100 calcium binding protein A16 [Source:HGNC Symbol;Acc:20441]	-2.977363959	-0.369502603	10.4293473	0.001240286	0.012477487	0.742455703	0.812765477	-4.621297774	-4.730697034	-4.610216385
ENSG00000173457	PPP1R14B	protein phosphatase 1, regulatory (inhibitor) subunit 14B [Source:HGNC Symbol;Acc:9057]	-1.278020005	3.240885476	10.42260026	0.001244825	0.012515981	3.875243923	3.789310181	2.556847448	2.126858114	3.04038489

ENSG00000105281	SLC1A5	solute carrier family 1 (neutral amino acid transporter), member 5 [Source:HGNC Symbol;Acc:10943]	-1.064751481	6.234806552	10.42060621	0.00124617	0.01252233	6.520218483	6.737574389	5.438791925	5.742824339	6.337033175
ENSG00000257432	RP11-244H18.4		-2.779364017	-0.419919237	10.40523895	0.001256583	0.012612684	0.823008709	0.056395802	-3.766908349	-4.730697034	-0.894110176
ENSG00000203805	PPAPDC1A	phosphatidic acid phosphatase type 2 domain containing 1A [Source:HGNC Symbol;Acc:23531]	-2.735050911	-0.010591472	10.40521638	0.001256598	0.012612684	1.268770164	0.947543428	-4.621297774	-3.888428289	-2.274700861
ENSG00000184602	SNN	stannin [Source:HGNC Symbol;Acc:11149]	1.253245839	7.166066554	10.3846882	0.001270646	0.012746399	6.037556089	6.580060189	7.242368802	8.00930852	7.203188517
ENSG00000204301	NOTCH4	notch 4 [Source:HGNC Symbol;Acc:7884]	1.765183997	1.633428885	10.37101584	0.001280091	0.012833807	0.597320328	0.881727766	0.778135316	2.086853261	2.5705316
ENSG00000153132	CLGN	calmegin [Source:HGNC Symbol;Acc:2060]	-1.79963121	1.30748339	10.36336513	0.001285407	0.012879747	2.217603867	1.878670417	-1.886652327	-0.655391447	1.515028015
ENSG00000196576	PLXNB2	plexin B2 [Source:HGNC Symbol;Acc:9104]	1.550892903	8.214362074	10.33641108	0.001304314	0.013061738	6.493291736	7.530303488	8.162757618	9.21589627	8.360105702
ENSG00000215873	FEN1P1	flap structure-specific endonuclease 1 pseudogene 1 [Source:HGNC Symbol;Acc:3651]	-1.850112222	1.005139184	10.33448317	0.001305677	0.013067934	1.89628059	1.889873947	-2.287883461	-0.406691783	0.415212929
ENSG00000178623	GPR35	G protein-coupled receptor 35 [Source:HGNC Symbol;Acc:4492]	1.378770765	3.600313901	10.33147275	0.001307808	0.013081807	2.695223288	3.303012124	3.72302193	4.363361466	3.352708597
ENSG00000167600	CYP2S1	cytochrome P450, family 2, subfamily S, polypeptide 1 [Source:HGNC Symbol;Acc:15654]	1.348715214	3.357911756	10.33000019	0.001308852	0.013084792	2.359395999	2.802033765	3.352990378	3.863006047	3.82032647
ENSG00000175544	CABP4	calcium binding protein 4 [Source:HGNC Symbol;Acc:1386]	1.704650594	0.03434053	10.32280393	0.001313965	0.013128434	-0.425964666	-0.157588231	0.632982468	0.074465887	-0.726511138
ENSG00000182487	NCF1B	neutrophil cytosolic factor 1B pseudogene [Source:HGNC Symbol;Acc:32522]	1.445775312	4.929454574	10.32133651	0.00131501	0.013131402	4.004641099	4.028581215	4.326707234	5.784063743	5.477389978
ENSG00000242612	DECR2	2,4-dienoyl CoA reductase 2, peroxisomal [Source:HGNC Symbol;Acc:2754]	-1.314343641	3.134009547	10.31765706	0.001317635	0.013150128	3.856229278	3.926195668	2.532495892	1.820287937	2.19580308
ENSG00000198223	CSF2RA	colony stimulating factor 2 receptor, alpha, low-affinity (granulocyte-macrophage) [Source:HGNC Symbol;Acc:2435]	1.303624158	5.574103551	10.31518562	0.0013194	0.013160268	4.453639214	5.006527308	5.219896278	6.349277663	6.023837348
ENSG00000213926	MSRB1P1	methionine sulfoxide reductase B1 pseudogene 1 [Source:HGNC Symbol;Acc:43985]	1.734985727	1.676286627	10.31354987	0.00132057	0.013164458	0.92386066	0.925936776	1.342349735	2.412303653	2.018338105
ENSG00000232160	RAP2C-AS1	RAP2C antisense RNA 1 [Source:HGNC Symbol;Acc:40957]	1.633900436	0.722266668	10.31178189	0.001321836	0.013169597	0.174562846	0.376157587	1.379231566	0.61425099	0.518960621
ENSG00000188906	LRRK2	leucine-rich repeat kinase 2 [Source:HGNC Symbol;Acc:18618]	1.399730361	7.530324735	10.30886635	0.001323926	0.013176497	5.983087844	6.829538218	7.424330022	8.427967276	7.850983411
ENSG00000125775	SDCBP2	syndecan binding protein (syntenin) 2 [Source:HGNC Symbol;Acc:15756]	1.787047832	-0.007254824	10.30872167	0.00132403	0.013176497	-0.625332215	-0.025429826	-0.390491059	0.335717742	0.04992943
ENSG00000167874	TMEM88	transmembrane protein 88 [Source:HGNC Symbol;Acc:32371]	2.146490908	1.276737368	10.30713414	0.001325169	0.013180365	0.174562846	0.407677918	0.401591237	2.428399826	1.410986673
ENSG00000197580	BCO2	beta-carotene oxygenase 2 [Source:HGNC Symbol;Acc:18503]	1.800497554	0.091023507	10.30267472	0.001328375	0.013204773	-0.775407551	0.095631612	-0.271377577	0.301169826	0.415212929
ENSG00000135972	MRPS9	mitochondrial ribosomal protein S9 [Source:HGNC Symbol;Acc:14501]	-1.062983732	4.751177457	10.27637759	0.001347442	0.013386729	5.314005372	5.32193738	4.256581955	3.808701883	4.422966584
ENSG00000214176	PLEKHM1P	pleckstrin homology domain containing, family M (with RUN domain) member 1 pseudogene [Source:HGNC Symbol;Acc:35411]	1.103640228	6.446377479	10.26600397	0.00135504	0.013454595	5.731582968	5.926320565	6.573570424	7.179490414	6.350572878
ENSG00000246263	KB-431C1.4		1.686574829	3.166223712	10.2614546	0.001358385	0.013480189	2.455179325	2.199986264	2.752269159	4.105033564	3.403296679
ENSG00000249943	RP11-213L8.1		1.575606734	1.321503172	10.25072425	0.001366309	0.013551165	0.714573228	0.836121102	1.644577544	1.770646721	1.177268129
ENSG00000165949	IFI27	interferon, alpha-inducible protein 27 [Source:HGNC Symbol;Acc:5397]	-1.852187454	0.924859586	10.24192215	0.001372845	0.013608293	2.187493018	1.699255595	-0.390491059	-2.419096061	-1.18885156
ENSG00000116701	NCF2	neutrophil cytosolic factor 2 [Source:HGNC Symbol;Acc:7661]	1.662434356	8.605655871	10.23780796	0.00137591	0.013630982	6.942255082	7.801767658	8.33676753	9.543475008	9.05476715
ENSG00000169704	GP9	glycoprotein IX (platelet) [Source:HGNC Symbol;Acc:4444]	1.680956024	3.775224652	10.22933669	0.001382244	0.013686005	2.304339895	3.352442362	3.407569039	4.785049495	3.889710441
ENSG00000196295	AC005154.6		1.025862395	5.992041724	10.22704119	0.001383965	0.013695324	5.326724129	5.761510433	6.46945395	6.486768637	5.500810255
ENSG00000143185	XCL2	chemokine (C motif) ligand 2 [Source:HGNC Symbol;Acc:10646]	-1.869350845	2.109799934	10.21400432	0.001393782	0.01378078	3.15547772	1.607225949	0.570569237	-0.350682213	2.83459139
ENSG00000148411	NACC2	NACC family member 2, BEN and BTB (POZ) domain containing [Source:HGNC Symbol;Acc:23846]	1.236616387	5.933418768	10.21349043	0.001394171	0.01378078	4.720448566	5.375723561	5.67681722	6.666308482	6.404060387
ENSG00000213630	RP11-772E11.1		-1.468754371	2.57813417	10.20311548	0.001402036	0.013850726	3.53210123	3.134276817	1.746900214	0.722650538	2.076176073
ENSG00000096063	SRPK1	SRSF protein kinase 1 [Source:HGNC Symbol;Acc:11305]	-1.026416247	6.0040254	10.1825169	0.001417785	0.013998436	6.329459238	6.462104936	5.57418573	5.088337272	6.136868511
ENSG00000119535	CSF3R	colony stimulating factor 3 receptor (granulocyte) [Source:HGNC Symbol;Acc:2439]	2.014942004	9.634869994	10.18003507	0.001419695	0.014009411	7.859285692	8.117779151	8.795230468	10.84627376	10.20413536
ENSG00000224796	RPL32P1	ribosomal protein L32 pseudogene 1 [Source:HGNC Symbol;Acc:10339]	2.413523262	0.959440962	10.17763964	0.00142154	0.014019743	0.46968809	-0.583862591	-0.10933503	2.387817579	0.264147325

ENSG00000127337	YEATS4	YEATS domain containing 4 [Source:HGNC Symbol;Acc:24859]	-1.15471239	4.360301093	10.17457219	0.001423907	0.014035201	5.017187838	4.830198586	3.892054915	2.952717131	4.247803769
ENSG00000167768	KRT1	keratin 1 [Source:HGNC Symbol;Acc:6412]	1.447658135	2.134714867	10.17271931	0.001425339	0.014041429	1.871242652	1.523383318	3.032495787	2.684399171	-0.894110176
ENSG00000073910	FRY	furry homolog (Drosophila) [Source:HGNC Symbol;Acc:20367]	1.326482548	5.306013433	10.17153592	0.001426254	0.014042564	4.061086952	4.796364522	5.075671919	6.168230563	5.566856794
ENSG00000162645	GBP2	guanylate binding protein 2, interferon-inducible [Source:HGNC Symbol;Acc:4183]	1.106885567	7.11661029	10.16530301	0.001431084	0.014082219	6.349994191	6.55344605	7.550452741	7.541613879	7.173354884
ENSG00000226328	CTA-217C2.1		1.267335528	3.468709344	10.15166516	0.00144171	0.01417883	2.979215452	3.02165139	3.796441777	3.963082217	3.260920169
ENSG00000184014	DENND5A	DENN/MADD domain containing 5A [Source:HGNC Symbol;Acc:19344]	1.187832487	7.208103653	10.14594018	0.001446194	0.014214968	6.010579004	6.649270053	7.386051389	7.898120176	7.395499534
ENSG00000237766	GGTA2P	glycoprotein, alpha-galactosyltransferase 2, pseudogene [Source:HGNC Symbol;Acc:4254]	2.12158892	0.447671369	10.14441064	0.001447394	0.014218806	-1.350769713	-0.204475541	-0.215316613	1.0274492	1.259453775
ENSG00000205565	RP11-497H16.6		1.78896122	1.73837758	10.13650972	0.001453611	0.014271895	-0.196880952	0.881727766	1.360908506	2.056104393	2.807527639
ENSG00000175866	BAIAP2	BAI1-associated protein 2 [Source:HGNC Symbol;Acc:947]	1.340021305	3.036572647	10.13196721	0.001457198	0.014299112	2.060341768	2.369639072	2.985428851	3.439318024	3.663137444
ENSG00000166025	AMOTL1	angiomotin like 1 [Source:HGNC Symbol;Acc:17811]	-1.554775272	2.708856643	10.12606475	0.001461872	0.014336961	3.43292027	3.247479563	0.365283811	1.75796486	2.893690069
ENSG00000092051	JPH4	junctophilin 4 [Source:HGNC Symbol;Acc:20156]	1.732454123	0.73280171	10.10891576	0.001475537	0.014462903	-0.425964666	0.438524285	0.721814282	0.773941511	1.374569317
ENSG00000079616	KIF22	kinesin family member 22 [Source:HGNC Symbol;Acc:6391]	-1.128127354	6.2966324	10.09923719	0.001483307	0.014530947	6.775578084	6.783725989	6.047135854	5.22592584	6.118404916
ENSG00000101977	MCF2	MCF.2 cell line derived transforming sequence [Source:HGNC Symbol;Acc:6940]	1.68004322	0.417255154	10.09809315	0.001484228	0.014531861	0.214927798	0.056395802	0.6928072	0.722650538	-0.095747566
ENSG00000186767	SPIN4	spindlin family, member 4 [Source:HGNC Symbol;Acc:27040]	-1.233214769	3.412635423	10.09680587	0.001485265	0.01453391	4.120728458	3.912527782	2.548775847	2.483362921	3.178820438
ENSG00000232742	RHOQP2	ras homolog family member Q pseudogene 2 [Source:HGNC Symbol;Acc:37836]	1.543081744	2.534594045	10.08227876	0.001497021	0.01464078	1.725181674	2.077947121	2.340128686	3.211794066	2.772967998
ENSG00000170390	DCLK2	doublecortin-like kinase 2 [Source:HGNC Symbol;Acc:19002]	2.109096066	0.21156423	10.08104105	0.001498027	0.014642461	-0.425964666	-0.252938016	-2.073364206	1.224680533	0.415212929
ENSG00000142552	RCN3	reticulocalbin 3, EF-hand calcium binding domain [Source:HGNC Symbol;Acc:21145]	1.490687257	4.322207016	10.07239617	0.001505072	0.014703135	3.583106845	3.53121644	3.885660513	5.335612556	4.39369447
ENSG00000100373	UPK3A	uroplakin 3A [Source:HGNC Symbol;Acc:12580]	1.872233299	1.138228514	10.07002475	0.00150701	0.014713883	0.000762307	0.789025519	0.6928072	2.014059105	1.218946104
ENSG00000138796	HADH	hydroxyacyl-CoA dehydrogenase [Source:HGNC Symbol;Acc:4799]	-1.091498378	4.488589782	10.0666712	0.001509756	0.014732495	5.03843049	5.039598485	3.945292586	3.45118362	4.311798918
ENSG00000229161	TCP1P1	t-complex 1 pseudogene 1 [Source:HGNC Symbol;Acc:11659]	-1.002425736	5.712848703	10.05504459	0.001519313	0.014817526	6.218439122	6.167509445	5.31814041	5.023588668	5.432701873
ENSG00000230453	ANKRD18B	ankyrin repeat domain 18B [Source:HGNC Symbol;Acc:23644]	-7.245673581	-0.928199885	10.05267824	0.001521266	0.014828333	0.292419846	0.016063025	-7.002070102	-7.002070102	-3.754655114
ENSG00000204764	RANBP17	RAN binding protein 17 [Source:HGNC Symbol;Acc:14428]	-1.927593139	0.369027755	10.0434927	0.00152887	0.014894185	1.361587606	1.184348258	-2.845623393	-1.572251189	-0.257803757
ENSG00000167992	VWCE	von Willebrand factor C and EGF domains [Source:HGNC Symbol;Acc:26487]	-1.976723072	0.841094085	10.04243654	0.001529747	0.014894461	1.752861822	1.634124584	-2.539967122	-2.419096061	0.872340852
ENSG00000189269	C22orf43	chromosome 22 open reading frame 43 [Source:HGNC Symbol;Acc:28031]	1.890348337	-0.024265894	10.0321667	0.0015383	0.014969431	-1.132483902	0.207316148	0.721814282	-0.146146183	-1.083770229
ENSG00000054967	RELT	RELT tumor necrosis factor receptor [Source:HGNC Symbol;Acc:13764]	1.506321729	6.870407001	10.0239954	0.001545139	0.015019336	5.887992541	5.926320565	6.295262663	7.99586966	7.032177549
ENSG00000181016	LSMEM1	leucine-rich single-pass membrane protein 1 [Source:HGNC Symbol;Acc:22036]	1.791084263	1.257202852	10.0240345	0.001545106	0.015019336	0.435934254	0.376157587	0.832340008	1.926103707	1.788521245
ENSG00000232368	FTLP2	ferritin, light polypeptide pseudogene 2 [Source:HGNC Symbol;Acc:4001]	1.373469529	3.439044445	10.01759059	0.001550522	0.015063314	2.680885373	3.066774989	3.30587588	4.006617121	3.72229752
ENSG00000126467	TSKS	testis-specific serine kinase substrate [Source:HGNC Symbol;Acc:30719]	1.593674809	0.934384472	10.01525	0.001552493	0.015074127	0.292419846	0.881727766	1.285196495	1.224680533	0.518960621
ENSG00000214391	RP11-121L10.3		-1.2240155	7.476248798	10.01063386	0.001556389	0.015103603	7.985144095	7.892114059	6.355500878	7.03717196	7.538151092
ENSG00000168062	BATF2	basic leucine zipper transcription factor, ATF-like 2 [Source:HGNC Symbol;Acc:25163]	1.813722956	0.476020279	10.00930459	0.001557513	0.015106157	-0.14487434	-0.112176905	-0.161352853	1.109604583	0.84598249
ENSG00000198585	NUDT16	nudix (nucleoside diphosphate linked moiety X)-type motif 16 [Source:HGNC Symbol;Acc:26442]	1.0404393	5.937953065	10.00803792	0.001558585	0.015108203	5.064896475	5.378708918	6.020388228	6.478095851	6.269221328
ENSG00000253193	FCGR1C	Fc fragment of IgG, high affinity Ic, receptor (CD64), pseudogene [Source:HGNC Symbol;Acc:3615]	1.874669527	0.45839973	10.00609604	0.001560229	0.015115795	-0.489407866	-1.191564836	-0.390491059	0.03296005	1.895203721
ENSG00000198734	F5	coagulation factor V (proaccelerin, labile factor) [Source:HGNC Symbol;Acc:3542]	1.218706054	5.635604585	9.999541675	0.001565792	0.015161322	4.466234785	5.340430139	5.624975047	6.261030623	5.897988131
ENSG00000272888	AC013394.2		1.037556089	6.33490741	9.991178852	0.001572919	0.015221934	5.853250786	5.899549479	6.755093741	6.937712694	5.787196413
ENSG00000185829	ARL17A	ADP-ribosylation factor-like 17A [Source:HGNC Symbol;Acc:24096]	1.59867346	1.517224788	9.989300302	0.001574524	0.015229076	0.174562846	0.881727766	1.205290011	1.224680533	2.693890668

ENSG00000224411	RP11-1033A18.1		-1.246445112	8.200677633	9.979986285	0.001582509	0.015297874	8.712689107	8.779231646	7.543107205	7.286017903	8.07283623
ENSG00000227502	RP1-249H1.4		-2.525976171	0.270164805	9.975032038	0.001586772	0.015330649	1.16956809	0.016063025	-3.766908349	-3.360122781	1.239342106
ENSG00000119729	RHOQ	ras homolog family member Q [Source:HGNC Symbol;Acc:17736]	1.135881218	6.168330729	9.960989436	0.001598921	0.015431038	5.361976146	5.635721147	6.140055546	6.835419742	6.388164233
ENSG00000273151	RP11-449P15.2		1.863757318	2.919949379	9.96167032	0.00159833	0.015431038	1.819824215	2.08771013	2.085000104	4.225075169	2.765955603
ENSG00000125319	C17orf53	chromosome 17 open reading frame 53 [Source:HGNC Symbol;Acc:28460]	-1.623614268	1.582502781	9.95413945	0.001604881	0.01548005	2.42108026	2.304152017	-0.907612816	0.03296005	1.498200525
ENSG00000261485	PAN3-AS1	PAN3 antisense RNA 1 [Source:HGNC Symbol;Acc:39932]	1.652824343	0.591679429	9.951060996	0.001607567	0.015497442	-0.306880589	-0.025429826	0.858697288	0.74852395	0.997359302
ENSG00000263244	RP11-473I1.10		1.173497559	6.888353506	9.947394412	0.001610772	0.015519817	5.974296451	6.363080927	7.031567709	7.698965506	6.758232955
ENSG00000184674	GSTT1	glutathione S-transferase theta 1 [Source:HGNC Symbol;Acc:4641]	-2.116231151	2.858810716	9.944758106	0.001613081	0.015533533	3.683607578	3.502308971	2.039642018	-7.002070102	2.944263918
ENSG00000153064	BANK1	B-cell scaffold protein with ankyrin repeats 1 [Source:HGNC Symbol;Acc:18233]	-1.151881996	6.237527559	9.93980541	0.001617426	0.015566843	6.894902327	6.463980227	5.860029792	5.483527863	6.063698636
ENSG00000139890	REM2	RAS (RAD and GEM)-like GTP binding 2 [Source:HGNC Symbol;Acc:20248]	1.552063818	2.538835506	9.937587659	0.001619376	0.015577069	1.920891399	1.355744383	2.358715867	3.314964122	2.880764656
ENSG00000100284	TOM1	target of myb1 (chicken) [Source:HGNC Symbol;Acc:11982]	1.18328533	6.136289442	9.926901284	0.001628805	0.015659186	5.296478906	5.667804805	6.064224218	6.978596154	6.092712941
ENSG00000238057	ZEB2-AS1	ZEB2 antisense RNA 1 [Source:HGNC Symbol;Acc:37149]	1.972909741	1.535488432	9.925415381	0.001630121	0.015663255	-0.250832646	1.010487126	1.205290011	2.491046607	1.774613685
ENSG00000114126	TFDP2	transcription factor Dp-2 (E2F dimerization partner 2) [Source:HGNC Symbol;Acc:11751]	-1.018712466	5.481784248	9.910733966	0.001643176	0.01578006	5.907365734	5.970523579	5.125366032	4.674675734	5.321115655
ENSG00000145491	ROPN1L	rhophilin associated tail protein 1-like [Source:HGNC Symbol;Acc:24060]	1.698879797	0.481759867	9.896119402	0.001656277	0.015897177	-0.094677427	0.133828548	-0.10933503	0.61425099	1.155968757
ENSG00000188026	RILPL1	Rab interacting lysosomal protein-like 1 [Source:HGNC Symbol;Acc:26814]	1.533131112	1.31055277	9.883931593	0.001667283	0.015994074	0.657137645	0.947543428	1.689321579	1.27894994	1.580441325
ENSG00000204472	AIF1	allograft inflammatory factor 1 [Source:HGNC Symbol;Acc:352]	1.399476847	7.449663653	9.878849679	0.001671894	0.016023466	6.122897312	6.903261088	7.297300326	8.298351105	7.729855779
ENSG00000219200	RNASEK	ribonuclease, RNase K [Source:HGNC Symbol;Acc:33911]	1.53327601	2.750029204	9.878543445	0.001672173	0.016023466	2.049222129	2.107040151	2.56487414	3.690214061	2.602374247
ENSG00000271605	MILR1	mast cell immunoglobulin-like receptor 1 [Source:HGNC Symbol;Acc:27570]	1.368281444	3.028585642	9.875744527	0.001674718	0.016039104	2.217603867	2.144940142	2.860570679	3.516638688	3.674416329
ENSG00000093134	VNN3	vanin 3 [Source:HGNC Symbol;Acc:16431]	1.911858201	3.665348973	9.863045715	0.001686317	0.016141383	2.614540713	2.492318033	2.56487414	4.78035404	4.170253557
ENSG00000163485	ADORA1	adenosine A1 receptor [Source:HGNC Symbol;Acc:262]	1.626832634	0.26245469	9.824907196	0.001721644	0.016470549	-0.046168491	0.343933181	0.602113338	0.434658742	-0.726511138
ENSG00000040275	SPDL1	spindle apparatus coiled-coil protein 1 [Source:HGNC Symbol;Acc:26010]	-1.158985504	4.070771533	9.811880155	0.001733882	0.016571495	4.7274414	4.6359475	3.579853854	2.749909693	3.7756399
ENSG00000138744	NAAA	N-acylethanolamine acid amidase [Source:HGNC Symbol;Acc:736]	1.110789389	6.819837406	9.811665933	0.001734084	0.016571495	5.872304469	6.429366465	7.04274659	7.414300622	6.875264274
ENSG00000131759	RARA	retinoic acid receptor, alpha [Source:HGNC Symbol;Acc:9864]	1.167386184	6.749863845	9.810100759	0.00173556	0.016576581	5.709680306	6.269679133	6.674390547	7.485744774	6.988413856
ENSG00000239961	LILRA4	leukocyte immunoglobulin-like receptor, subfamily A (with TM domain), member 4 [Source:HGNC Symbol;Acc:15503]	2.089040099	2.127234329	9.805552649	0.001739857	0.01660859	0.133035942	0.310972527	-0.453950945	2.475638093	3.700395796
ENSG00000113368	LMNB1	lamin B1 [Source:HGNC Symbol;Acc:6637]	-1.045688206	6.413004705	9.803886981	0.001741434	0.016614605	6.539219722	6.76597427	5.483939025	5.717666472	6.983888028
ENSG00000088826	SMOX	spermine oxidase [Source:HGNC Symbol;Acc:15862]	1.416505572	4.036971006	9.790234701	0.001754411	0.016729321	2.894768068	3.573519788	3.782742091	4.894834669	4.24276145
ENSG00000146859	TMEM140	transmembrane protein 140 [Source:HGNC Symbol;Acc:21870]	1.163991037	4.266711825	9.78270119	0.001761613	0.016788882	3.690780833	3.795280718	4.36857882	4.786611257	4.386854181
ENSG00000125900	SIRPD	signal-regulatory protein delta [Source:HGNC Symbol;Acc:16248]	1.795927229	0.666236319	9.780128657	0.00176408	0.016803266	-0.365194284	0.583576386	0.505333341	1.089500161	0.872340852
ENSG00000140961	OSGIN1	oxidative stress induced growth inhibitor 1 [Source:HGNC Symbol;Acc:30093]	1.776734035	0.677829424	9.775313516	0.001768706	0.016838192	-0.365194284	0.637745977	0.401591237	1.048430793	0.997359302
ENSG00000255422	AP002954.4		1.690027937	0.778538396	9.771818505	0.001772071	0.016861087	-0.14487434	0.968831255	1.285196495	1.187332556	-0.506781561
ENSG00000242324	RP4-576H24.2		1.78987045	0.16041496	9.769140529	0.001774654	0.016876517	-0.555769858	0.207316148	-0.329705368	0.58582888	0.223773082
ENSG00000005102	MEOX1	mesenchyme homeobox 1 [Source:HGNC Symbol;Acc:7013]	1.950891545	0.03145325	9.762200363	0.001781366	0.016931173	-1.350769713	0.133828548	0.934997507	-0.956081001	-0.095747566
ENSG00000182578	CSF1R	colony stimulating factor 1 receptor [Source:HGNC Symbol;Acc:2433]	1.492816047	8.275479236	9.75702105	0.001786391	0.016969752	6.530250323	7.680800692	8.254169536	9.246646814	8.403537164
ENSG00000108924	HLF	hepatic leukemia factor [Source:HGNC Symbol;Acc:4977]	1.799140037	0.592283175	9.753251604	0.001790058	0.016995387	-0.094677427	0.277241192	1.550698027	-0.406691783	0.551948963
ENSG00000242338	BMS1P4	BMS1 pseudogene 4 [Source:HGNC Symbol;Acc:23652]	1.686650659	0.328430132	9.734218314	0.001808689	0.017162994	-0.046168491	0.133828548	0.437007322	0.823471408	-0.316136847
ENSG00000166278	C2	complement component 2 [Source:HGNC Symbol;Acc:1248]	1.90027203	-0.130681202	9.728983371	0.001813848	0.017202647	-0.365194284	-0.932821174	-1.438615935	0.301169826	0.485200289
ENSG00000138346	DNA2	DNA replication helicase/nuclease 2 [Source:HGNC Symbol;Acc:2939]	-1.207266081	3.515380915	9.723809134	0.001818961	0.017241619	4.126030116	4.038682887	2.665349954	2.27662903	3.561506932

ENSG00000163162	RNF149	ring finger protein 149 [Source:HGNC Symbol;Acc:23137]	1.28628814	7.074530849	9.721847783	0.001820904	0.017241619	6.066797471	6.4375017	6.921397365	7.912496459	7.301940144
ENSG00000264290	RP11-68I3.4		1.799635429	-0.176435245	9.722073676	0.00182068	0.017241619	-1.132483902	-0.30308518	0.250534523	0.074465887	-0.649489103
ENSG00000134824	FADS2	fatty acid desaturase 2 [Source:HGNC Symbol;Acc:3575]	-1.777373611	6.292283084	9.719527309	0.001823204	0.017247692	6.889457078	6.796060714	3.510897178	5.886657785	6.512779767
ENSG00000197582	GPX1P1	glutathione peroxidase pseudogene 1 [Source:HGNC Symbol;Acc:4560]	1.24062682	5.911858633	9.719219006	0.00182351	0.017247692	5.082729028	5.463639162	5.660054909	6.750342465	6.011290397
ENSG00000225873	LINC00694	long intergenic non-protein coding RNA 694 [Source:HGNC Symbol;Acc:44570]	1.814005743	1.013213016	9.708741152	0.001833936	0.017336966	0.133035942	-0.025429826	0.471574783	1.820287937	1.498200525
ENSG00000139926	FRMD6	FERM domain containing 6 [Source:HGNC Symbol;Acc:19839]	-1.671635061	1.152136987	9.705594416	0.001837079	0.017357331	2.167064212	1.785765038	-0.453950945	-1.133744865	0.763888603
ENSG00000164850	GPER1	G protein-coupled estrogen receptor 1 [Source:HGNC Symbol;Acc:4485]	1.83529677	0.679621404	9.703655416	0.001839018	0.017366308	0.329658892	-0.252938016	0.125863448	1.27894994	1.090116303
ENSG00000047634	SCML1	sex comb on midleg-like 1 (Drosophila) [Source:HGNC Symbol;Acc:10580]	1.371790132	3.49178513	9.701935126	0.001840741	0.017373227	2.42108026	3.552523163	4.203585614	3.965841977	2.267069113
ENSG00000258011	HMGA1P3	high mobility group AT-hook 1 pseudogene 3 [Source:HGNC Symbol;Acc:13323]	-1.185105701	3.641724675	9.686975954	0.001855787	0.017505826	4.316608668	4.296139761	2.793840748	2.498689587	3.314770126
ENSG00000215218	UBE2QL1	ubiquitin-conjugating enzyme E2Q family-like 1 [Source:HGNC Symbol;Acc:37269]	-3.069559847	-0.414598402	9.684943682	0.001857841	0.017515787	0.94800933	0.310972527	-3.766908349	-7.002070102	-2.274700861
ENSG00000116649	SRM	spermidine synthase [Source:HGNC Symbol;Acc:11296]	-1.038766249	5.021262351	9.681298929	0.00186153	0.017541146	5.593926721	5.474842644	4.68073531	4.222769665	4.643690936
ENSG00000059377	TBXAS1	thromboxane A synthase 1 (platelet) [Source:HGNC Symbol;Acc:11609]	1.288678403	6.916073761	9.672132349	0.00187084	0.017619423	5.564941291	6.183531466	6.578534562	7.868930563	7.268528284
ENSG00000261026	CTD-3247F14.2		2.81543799	1.942720728	9.669082225	0.001873949	0.017639236	0.174562846	0.207316148	-2.073364206	3.47849629	2.390748373
ENSG00000141076	CIRH1A	cirrhosis, autosomal recessive 1A (cirhin) [Source:HGNC Symbol;Acc:1983]	-1.063784766	5.009434663	9.667394482	0.001875671	0.017645986	5.576605438	5.564968749	4.711792159	4.025258184	4.536516067
ENSG00000151287	TEX30	testis expressed 30 [Source:HGNC Symbol;Acc:25188]	-1.295560746	3.117763155	9.666167949	0.001876924	0.017646056	3.975567801	3.545455828	2.171650019	2.126858114	2.772967998
ENSG00000121858	TNFSF10	tumor necrosis factor (ligand) superfamily, member 10 [Source:HGNC Symbol;Acc:11925]	1.075269703	6.802936199	9.665419237	0.001877689	0.017646056	5.720235091	6.2094731	6.897760548	7.06607384	7.47639523
ENSG00000112964	GHR	growth hormone receptor [Source:HGNC Symbol;Acc:4263]	-3.59964507	-0.511735334	9.659320996	0.001883932	0.017695256	1.018120824	0.095631612	-4.621297774	-7.002070102	-3.754655114
ENSG00000106484	MEST	mesoderm specific transcript [Source:HGNC Symbol;Acc:7028]	-1.2624576	3.646431965	9.645567402	0.001898091	0.017809182	4.525609656	4.121805905	2.642776537	2.787834084	3.125029543
ENSG00000210196	MT-TP	mitochondrially encoded tRNA proline [Source:HGNC Symbol;Acc:7494]	1.350494228	2.69810156	9.644161283	0.001899544	0.0178133	2.304339895	1.184348258	2.665349954	3.535338275	2.814341322
ENSG00000245532	NEAT1	nuclear paraspeckle assembly transcript 1 (non-protein coding) [Source:HGNC Symbol;Acc:30815]	1.589585118	9.566327742	9.637728223	0.001906209	0.017866253	8.636027243	8.567379764	9.684287714	10.5932589	9.334617452
ENSG00000132341	RAN	RAN, member RAS oncogene family [Source:HGNC Symbol;Acc:9846]	-1.013428784	6.037899557	9.635004781	0.001909037	0.017883216	6.530250323	6.434157451	5.698868248	5.347300717	5.824799093
ENSG00000088053	GP6	glycoprotein VI (platelet) [Source:HGNC Symbol;Acc:14388]	1.913485462	2.342679462	9.631452167	0.001912733	0.017908283	0.502670228	2.077947121	2.107155349	3.340707202	2.31590077
ENSG00000120256	LRP11	low density lipoprotein receptor-related protein 11 [Source:HGNC Symbol;Acc:16936]	-1.36317991	2.402386878	9.625652906	0.001918782	0.017955342	2.913284701	3.212211862	1.432844522	0.696304622	2.32547185
ENSG00000257078	RP3-461F17.2		1.461329407	1.832776811	9.62342962	0.001921106	0.017957947	1.448792784	1.434365866	2.5886897	1.891686981	1.259453775
ENSG00000224025	RP11-274B18.3		1.678681016	1.383890806	9.624103907	0.001920401	0.017957947	0.133035942	0.310972527	0.9100098	1.770646721	2.372397358
ENSG00000253818	IGLV1-41	immunoglobulin lambda variable 1-41 (pseudogene) [Source:HGNC Symbol;Acc:5878]	-2.587054779	-0.149686433	9.612529684	0.001932542	0.018055233	1.127895066	0.016063025	-3.233942493	-4.730697034	-0.147769603
ENSG00000132361	CLUH	clustered mitochondria (cluA/CLU1) homolog [Source:HGNC Symbol;Acc:29094]	-1.007272572	5.968389204	9.606385151	0.00193902	0.018106111	6.505038703	6.478431669	5.698868248	5.207388815	5.455763778
ENSG00000160339	FCN2	ficolin (collagen/fibrinogen domain containing lectin) 2 [Source:HGNC Symbol;Acc:3624]	1.878892665	1.484638435	9.598241822	0.001947638	0.018176916	-0.14487434	1.09035546	1.32354911	2.371259556	1.580441325
ENSG00000204466	DGKK	diacylglycerol kinase, kappa [Source:HGNC Symbol;Acc:32395]	2.411706312	1.657797346	9.596979628	0.001948977	0.01817975	0.92386066	0.016063025	-0.329705368	3.044343756	1.81594051
ENSG00000174791	RIN1	Ras and Rab interactor 1 [Source:HGNC Symbol;Acc:18749]	1.291094187	3.956317859	9.590838716	0.001955506	0.018230965	2.723478589	3.667655396	4.190675219	4.532993113	4.054164368
ENSG00000080824	HSP90AA1	heat shock protein 90kDa alpha (cytosolic), class A member 1 [Source:HGNC Symbol;Acc:5253]	-1.300868195	8.629783152	9.580603395	0.001966438	0.018323149	9.144878678	9.118205518	8.030981562	7.989442081	8.421219819
ENSG00000088367	EPB41L1	erythrocyte membrane protein band 4.1-like 1 [Source:HGNC Symbol;Acc:3378]	-2.857293265	-0.18088912	9.578028062	0.001969198	0.018339135	1.18996172	0.376157587	-7.002070102	-3.360122781	-1.18885156
ENSG00000100116	GCAT	glycine C-acetyltransferase [Source:HGNC Symbol;Acc:4188]	-1.794200275	1.208694595	9.573988712	0.001973535	0.018360049	2.359395999	1.724506406	-0.059127673	-2.205328685	0.735452007

ENSG00000165682	CLEC1B	C-type lectin domain family 1, member B [Source:HGNC Symbol;Acc:24356]	1.875066322	2.142804633	9.574412381	0.00197308	0.018360049	0.657137645	1.773719635	1.674560554	3.03386134	2.435629945
ENSG00000165209	STRBP	spermatid perinuclear RNA binding protein [Source:HGNC Symbol;Acc:16462]	-1.025575496	5.420807736	9.571546386	0.001976163	0.018374753	5.910441444	5.809497926	5.006632866	4.54226781	5.400235998
ENSG00000160447	PKN3	protein kinase N3 [Source:HGNC Symbol;Acc:17999]	-1.30747153	2.909853652	9.568520138	0.001979423	0.018395324	3.733087128	3.590786525	2.311791045	1.314027603	2.164148811
ENSG00000214212	C19orf38	chromosome 19 open reading frame 38 [Source:HGNC Symbol;Acc:34073]	1.284895785	6.268427446	9.561818686	0.001986662	0.01845283	5.034206909	5.839421768	6.037073363	7.091567975	6.540268057
ENSG00000101336	HCK	hemopoietic cell kinase [Source:HGNC Symbol;Acc:4840]	1.396585104	7.96680817	9.54443727	0.002005563	0.01861854	6.521727713	7.163685965	7.687721106	8.858363173	8.430673552
ENSG00000273356	RP11-804H8.6		1.739528704	1.328628628	9.541652476	0.002008608	0.018636954	0.686141241	0.376157587	1.076464519	2.086853261	1.596341512
ENSG00000120262	CCDC170	coiled-coil domain containing 170 [Source:HGNC Symbol;Acc:21177]	1.435713201	2.248505372	9.53498824	0.002015915	0.018694866	1.287817526	1.647388141	2.233384453	2.697741416	2.751827722
ENSG00000172824	CES4A	carboxylesterase 4A [Source:HGNC Symbol;Acc:26741]	1.229663577	3.403489803	9.530895218	0.002020415	0.018726712	2.552892059	3.216667762	3.945292586	3.71994361	3.152175675
ENSG00000198053	SIRPA	signal-regulatory protein alpha [Source:HGNC Symbol;Acc:9662]	1.342071835	7.295719559	9.527887779	0.002023729	0.018727761	5.663940507	6.581788344	6.918268075	7.969985717	8.061605139
ENSG00000114853	ZBTB47	zinc finger and BTB domain containing 47 [Source:HGNC Symbol;Acc:26955]	1.426282111	3.785520447	9.528493276	0.002023061	0.018727761	2.730456787	3.189723558	3.4515252	4.558813477	4.201775615
ENSG00000234290	AC116366.6		2.48470555	3.093669963	9.523234577	0.002028867	0.018765413	2.103982592	0.637745977	1.285196495	4.649148511	3.168221511
ENSG00000066336	SPI1	spleen focus forming virus (SFFV) proviral integration oncogene [Source:HGNC Symbol;Acc:11241]	1.453634108	8.020786307	9.521421864	0.002030872	0.018774067	6.608088569	7.241729892	7.721998354	9.015043863	8.325944358
ENSG00000213204	C6orf165	chromosome 6 open reading frame 165 [Source:HGNC Symbol;Acc:21405]	1.605831251	0.499638412	9.517455167	0.002035266	0.018804789	0.000762307	0.133828548	0.602113338	0.229488395	1.021113618
ENSG00000270382	RP1-152L7.9		1.038145507	4.999017307	9.515968087	0.002036916	0.018810134	4.371207268	4.586881416	4.969322447	5.409439159	5.360054437
ENSG00000161714	PLCD3	phospholipase C, delta 3 [Source:HGNC Symbol;Acc:9061]	1.472994566	1.761984918	9.501833004	0.002052667	0.018945623	1.148882041	1.371815184	1.881530361	2.076676283	1.994537666
ENSG00000227051	C14orf132	chromosome 14 open reading frame 132 [Source:HGNC Symbol;Acc:20346]	1.666928036	1.252091532	9.499610563	0.002055155	0.018958617	0.329658892	1.010487126	2.1289755	0.642123966	1.298855074
ENSG00000198794	SCAMP5	secretory carrier membrane protein 5 [Source:HGNC Symbol;Acc:30386]	-1.412874049	2.544244836	9.495671976	0.002059571	0.018989378	3.216393469	2.802033765	0.858697288	1.069111613	3.125029543
ENSG00000203710	CR1	complement component (3b/4b) receptor 1 (Knops blood group) [Source:HGNC Symbol;Acc:2334]	1.147270575	6.355019974	9.479079713	0.002078282	0.019151832	5.187866611	5.707920758	6.111570172	6.912900738	7.02961811
ENSG00000197785	ATAD3A	ATPase family, AAA domain containing 3A [Source:HGNC Symbol;Acc:25567]	-1.046743014	4.507716784	9.475321408	0.002082544	0.019171176	5.127030457	5.160167117	4.026005012	3.683523464	3.765128041
ENSG00000126709	IFI6	interferon, alpha-inducible protein 6 [Source:HGNC Symbol;Acc:4054]	1.188687098	4.612260085	9.475302713	0.002082565	0.019171176	3.683607578	3.777294464	4.254101979	5.170781053	5.335397107
ENSG00000182574	RP3-341D10.1		1.279038995	3.601094939	9.470937884	0.002087526	0.019206773	2.955587776	3.294606553	3.653213102	4.100017768	3.711388219
ENSG00000260837	RP11-434B12.1		1.558514828	0.967804555	9.457975455	0.00210233	0.019332845	0.090278201	0.664086661	1.432844522	1.261086007	0.819133578
ENSG00000253210	RP11-809O17.1		1.961564129	0.978943192	9.429501006	0.002135224	0.019625054	0.046214424	-0.068151468	-0.329705368	1.90325069	1.612068369
ENSG00000108405	P2RX1	purinergic receptor P2X, ligand-gated ion channel, 1 [Source:HGNC Symbol;Acc:8533]	1.424433692	6.067822978	9.417900076	0.002148775	0.019739266	5.060749778	5.296905755	5.430029956	7.079195854	6.393861558
ENSG00000236530	AC097711.1		-1.342298703	3.434317127	9.413830796	0.002153548	0.019766415	4.29323497	3.856523346	2.06249931	2.59453048	3.240829074
ENSG00000137575	SDCBP	syndecan binding protein (syntenin) [Source:HGNC Symbol;Acc:10662]	1.257615626	6.978876885	9.413461755	0.002153982	0.019766415	5.713207155	6.416834926	6.825686891	7.637765624	7.491353223
ENSG00000273033	RP11-67L2.2		1.323817314	3.901584686	9.411292334	0.002156532	0.019779475	2.688072143	3.590786525	4.068967903	4.551483264	3.974279416
ENSG00000157601	MX1	myxovirus (influenza virus) resistance 1, interferon-inducible protein p78 (mouse) [Source:HGNC Symbol;Acc:7532]	1.093490352	5.604616876	9.409091264	0.002159122	0.019792892	4.820306233	4.923101506	5.397814138	6.125674787	6.179052931
ENSG00000077942	FBLN1	fibulin 1 [Source:HGNC Symbol;Acc:3600]	-2.538810607	0.285342404	9.395609384	0.002175057	0.019918166	1.752861822	1.050973916	-3.233942493	-4.730697034	-2.274700861
ENSG00000075188	NUP37	nucleoporin 37kDa [Source:HGNC Symbol;Acc:29929]	-1.142990519	4.251484829	9.395645178	0.002175014	0.019918166	4.985454998	4.685008352	3.366829833	3.439318024	4.033874407
ENSG00000151779	NBAS	neuroblastoma amplified sequence [Source:HGNC Symbol;Acc:15625]	-1.045272452	5.660553606	9.381957525	0.002191314	0.020056581	6.085287286	6.121914966	5.439883439	4.791286425	5.459028447
ENSG00000125347	IRF1	interferon regulatory factor 1 [Source:HGNC Symbol;Acc:6116]	1.28932743	7.695004582	9.379693863	0.002194022	0.020070899	6.921443401	6.868641225	7.752188616	8.768358552	7.190478141
ENSG00000009694	TENM1	teneurin transmembrane protein 1 [Source:HGNC Symbol;Acc:8117]	1.532546317	2.52971177	9.373030087	0.002202012	0.020133502	1.018120824	2.377619831	3.049757403	3.221064093	1.908000984
ENSG00000270610	RP11-486O13.3		1.218408457	4.035345519	9.372000633	0.002203249	0.020134326	3.428636013	3.777294464	4.894863265	3.874800997	3.681886894
ENSG00000187699	C2orf88	chromosome 2 open reading frame 88 [Source:HGNC Symbol;Acc:28191]	1.262718133	5.001637445	9.369590321	0.002206148	0.020150329	4.131312362	4.459201101	4.745729503	5.994651253	4.895769655
ENSG00000131979	GCH1	GTP cyclohydrolase 1 [Source:HGNC Symbol;Acc:4193]	1.081180522	5.690499198	9.364101569	0.002212764	0.020200248	4.918288272	5.342470334	5.727754931	6.32905707	5.747697761
ENSG00000111291	GPRC5D	G protein-coupled receptor, family C, group 5, member D [Source:HGNC Symbol;Acc:13310]	-2.006458824	0.041695596	9.361954373	0.002215358	0.020213415	1.268770164	0.98980953	-2.539967122	-2.97427061	-2.832686105

ENSG00000167711	SERPINF2	serpin peptidase inhibitor, clade F (alpha-2 antiplasmin, pigment epithelium derived factor), member 2 [Source:HGNC Symbol;Acc:9075]	1.63270884	0.962755143	9.358764236	0.002219217	0.020238108	0.292419846	0.242702272	0.28980717	1.187332556	1.84284836
ENSG00000258759	RP11-1012A1.7		-1.495300894	1.67558119	9.356476667	0.002221989	0.020242352	2.488491009	2.353543875	0.570569237	-0.296766077	1.410986673
ENSG00000198517	MAFK	v-maf avian musculoaponeurotic fibrosarcoma oncogene homolog K [Source:HGNC Symbol;Acc:6782]	1.094938062	4.668502982	9.356647426	0.002221782	0.020242352	4.245319886	3.974354883	4.812985108	5.255549576	4.694433395
ENSG00000188603	CLN3	ceroid-lipofuscinosis, neuronal 3 [Source:HGNC Symbol;Acc:2074]	1.249304313	3.432705302	9.346016311	0.002234707	0.020347656	2.75119112	3.152996839	3.664462604	4.084864894	3.074840951
ENSG00000167880	EVPL	envoplakin [Source:HGNC Symbol;Acc:3503]	-1.783892948	2.261538656	9.341571649	0.002240133	0.02038649	3.176070082	3.303012124	1.718398126	-2.670141381	0.378903451
ENSG00000138185	ENTPD1	ectonucleoside triphosphate diphosphohydrolase 1 [Source:HGNC Symbol;Acc:3363]	1.201753921	6.266369526	9.326021474	0.002259223	0.020538927	5.451271999	5.55883994	6.049998008	7.15521396	6.408007209
ENSG00000205959	RP11-689P11.2		1.65759258	0.105173619	9.326645307	0.002258454	0.020538927	-0.555769858	0.343933181	0.365283811	0.074465887	-0.316136847
ENSG00000166881	TMEM194A	transmembrane protein 194A [Source:HGNC Symbol;Acc:29001]	-1.04683935	5.407157591	9.308846342	0.0022805	0.020699847	5.805312087	5.992777004	5.119928242	4.231969659	5.274935181
ENSG00000178127	NDUFV2	NADH dehydrogenase (ubiquinone) flavoprotein 2, 24kDa [Source:HGNC Symbol;Acc:7717]	1.488246919	1.526697381	9.309810033	0.002279301	0.020699847	1.210071084	1.16603322	1.956663165	2.076676283	0.551948963
ENSG00000233622	CYP2T2P	cytochrome P450, family 2, subfamily T, polypeptide 2, pseudogene [Source:HGNC Symbol;Acc:18852]	1.538082821	0.865546715	9.310095812	0.002278946	0.020699847	0.435934254	0.277241192	1.245796404	1.414416401	0.303422373
ENSG00000243789	JMJD7	jumonji domain containing 7 [Source:HGNC Symbol;Acc:34397]	1.645583063	0.15028167	9.307930338	0.002281641	0.020699847	-0.489407866	0.133828548	0.632982468	0.192266291	-0.376928291
ENSG00000233325	MIPEPP3	mitochondrial intermediate peptidase pseudogene 3 [Source:HGNC Symbol;Acc:39458]	1.71255562	0.078668066	9.296078694	0.002296449	0.020823429	-0.698419327	-0.252938016	-0.10933503	0.335717742	0.450630967
ENSG00000271722	RP11-446E9.2		1.917277527	0.049936679	9.29451825	0.002298406	0.020830415	-0.365194284	-0.855949929	-0.390491059	0.798919003	0.095393329
ENSG00000162711	NLRP3	NLR family, pyrin domain containing 3 [Source:HGNC Symbol;Acc:16400]	1.384412795	5.98422043	9.289335272	0.002304918	0.020878655	4.574904087	5.489646553	5.521542954	6.835796941	6.447988456
ENSG00000224837	GCSHP5	glycine cleavage system protein H pseudogene 5 [Source:HGNC Symbol;Acc:44195]	-1.425759607	1.990980708	9.285958162	0.002309171	0.020906394	2.882290283	2.612158157	0.884581662	0.369457657	1.564363944
ENSG00000173369	C1QB	complement component 1, q subcomponent, B chain [Source:HGNC Symbol;Acc:1242]	-1.482030449	2.934324075	9.283791403	0.002311904	0.020911176	4.052759582	2.84296036	1.829176068	1.65228493	2.906500707
ENSG00000103528	SYT17	synaptotagmin XVII [Source:HGNC Symbol;Acc:24119]	1.682023743	1.188850621	9.28365074	0.002312082	0.020911176	0.627538984	0.904001603	0.858697288	2.13668837	0.551948963
ENSG00000137877	SPTBN5	spectrin, beta, non-erythrocytic 5 [Source:HGNC Symbol;Acc:15680]	1.269480205	2.681314122	9.28260674	0.0023134	0.020912324	2.386155691	2.353543875	3.242232575	3.266541267	1.239342106
ENSG00000249476	CTD-2587M2.1		1.524047656	0.964546101	9.276294385	0.002321386	0.020973715	0.534915169	0.689955027	1.120696904	1.348272594	0.763888603
ENSG00000122121	XPNPEP2	X-prolyl aminopeptidase (aminopeptidase P) 2, membrane-bound [Source:HGNC Symbol;Acc:12823]	1.704046079	0.225020218	9.274052208	0.002324229	0.020988603	0.133035942	-0.523165112	0.125863448	0.192266291	0.646616095
ENSG00000181481	RNF135	ring finger protein 135 [Source:HGNC Symbol;Acc:21158]	1.154835574	5.114088624	9.270331329	0.002328956	0.021020472	4.377889068	4.595461025	4.947999288	5.770710897	5.40813974
ENSG00000254750	CASP1P2	caspase 1, apoptosis-related cysteine peptidase pseudogene 2 [Source:HGNC Symbol;Acc:43776]	2.085591772	0.385152244	9.263478042	0.002337687	0.021088432	-1.608074432	0.376157587	1.629349082	0.265774258	-1.55968627
ENSG00000230756	RHOQP3	ras homolog family member Q pseudogene 3 [Source:HGNC Symbol;Acc:37837]	1.799545697	0.835888712	9.255514474	0.002347874	0.021169452	0.046214424	0.095631612	0.365283811	1.55300499	1.218946104
ENSG00000236603	RANP1	RAN, member RAS oncogene family pseudogene 1 [Source:HGNC Symbol;Acc:21631]	-1.051329365	6.97917624	9.2485908	0.002356767	0.021235367	7.535428582	7.476047346	6.481694967	6.048366612	6.800024919
ENSG00000127586	CHTF18	CTF18, chromosome transmission fidelity factor 18 homolog (S. cerevisiae) [Source:HGNC Symbol;Acc:18435]	-1.01456989	4.934189845	9.233490542	0.002376282	0.021392632	5.408403051	5.40822756	4.658403207	4.038427289	4.698122044
ENSG00000128309	MPST	mercaptopyruvate sulfurtransferase [Source:HGNC Symbol;Acc:7223]	-1.108570373	5.075299236	9.229822605	0.002381047	0.021424543	5.62327883	5.458945245	3.939130343	4.631875924	5.124061664
ENSG00000229644	NAMPTL	nicotinamide phosphoribosyltransferase-like [Source:HGNC Symbol;Acc:17633]	1.99594391	6.849288132	9.22205154	0.002391175	0.021504646	5.532369944	5.886324968	5.642147491	8.333767185	6.617290475
ENSG00000065534	MYLK	myosin light chain kinase [Source:HGNC Symbol;Acc:7590]	1.598909147	3.83588348	9.217431802	0.002397216	0.021547938	2.103982592	3.332051848	3.371413639	4.871476853	4.096705135
ENSG00000176853	FAM91A1	family with sequence similarity 91, member A1 [Source:HGNC Symbol;Acc:26306]	1.082566689	5.38913238	9.211743907	0.002404675	0.021603927	4.760202192	5.007813394	5.444241255	5.997351335	5.411513848

ENSG00000107833	NPM3	nucleophosmin/nucleoplasmin 3 [Source:HGNC Symbol;Acc:7931]	-1.282007192	3.623936617	9.207794028	0.002409869	0.021639515	4.453639214	4.043707322	3.133097051	2.116960417	3.300281982
ENSG00000164068	RNF123	ring finger protein 123 [Source:HGNC Symbol;Acc:21148]	-1.010625221	6.137943218	9.203324014	0.002415761	0.021675115	6.503511903	6.755294011	5.386536308	5.739602708	5.852371578
ENSG00000135540	NHSL1	NHS-like 1 [Source:HGNC Symbol;Acc:21021]	1.705479558	1.245028707	9.202913045	0.002416303	0.021675115	0.292419846	0.881727766	1.007464306	2.194303497	0.923655414
ENSG00000131981	LGALS3	lectin, galactoside-binding, soluble, 3 [Source:HGNC Symbol;Acc:6563]	1.160349445	6.399426741	9.199067581	0.002421384	0.021698521	5.48053991	5.915397584	6.270935771	7.138493962	6.624577478
ENSG00000196730	DAPK1	death-associated protein kinase 1 [Source:HGNC Symbol;Acc:2674]	1.299220428	6.19921079	9.19777703	0.002423092	0.021702745	4.884277045	5.448564552	5.652542018	7.102268031	6.730584323
ENSG00000169245	CXCL10	chemokine (C-X-C motif) ligand 10 [Source:HGNC Symbol;Acc:10637]	1.896089357	0.396658557	9.195897832	0.002425581	0.021713958	-0.625332215	-0.112176905	-0.010608983	1.27894994	0.378903451
ENSG00000138780	GSTCD	glutathione S-transferase, C-terminal domain containing [Source:HGNC Symbol;Acc:25806]	-1.23258855	3.053604686	9.19447037	0.002427473	0.021714709	3.76402209	3.647994137	2.28288562	1.948600359	2.671550033
ENSG00000160201	U2AF1	U2 small nuclear RNA auxiliary factor 1 [Source:HGNC Symbol;Acc:12453]	1.314902334	6.401249363	9.193968595	0.002428139	0.021714709	5.624215801	5.805066297	6.132649283	7.539994371	5.925782981
ENSG00000254006	RP11-1D12.2		-1.988123885	1.274745976	9.192585616	0.002429974	0.021720057	1.819824215	0.98980953	-2.845623393	-2.205328685	2.586540774
ENSG00000188167	TMPPE	transmembrane protein with metallophosphoesterase domain [Source:HGNC Symbol;Acc:33865]	1.385198619	2.864598455	9.190927493	0.002432176	0.021728678	2.071376358	2.345428436	2.840873769	3.723209392	2.693890668
ENSG00000270055	CTD-3092A11.2		1.28281661	3.869473904	9.169998899	0.002460147	0.021967387	3.18625708	3.269091943	3.708603425	4.512376441	4.183471549
ENSG00000261716	RP11-196G18.22		1.244327412	3.038501108	9.166777722	0.002464481	0.021994899	2.217603867	2.632310473	3.523307974	3.174106579	3.240829074
ENSG00000240710	RP11-430C7.4		1.659794247	0.626190727	9.164067496	0.002468134	0.022016304	-0.250832646	0.610915408	1.379231566	0.497040107	0.095393329
ENSG00000223773	CD99P1	CD99 molecule pseudogene 1 [Source:HGNC Symbol;Acc:7083]	1.392487488	2.623409692	9.158646064	0.002475457	0.022059208	2.114689606	1.986973075	2.657864658	3.26205752	2.664025609
ENSG00000234506	RP11-274B18.2		2.068137545	0.726687829	9.158758939	0.002475304	0.022059208	0.000762307	-0.647226338	-0.329705368	1.665926088	1.259453775
ENSG00000261644	RP11-327F22.2		1.488747912	2.181586796	9.154380008	0.002481234	0.02208827	1.268770164	1.673554946	2.171650019	3.007317163	2.087470534
ENSG00000164845	FAM86FP	family with sequence similarity 86, member F, pseudogene [Source:HGNC Symbol;Acc:42357]	1.644600453	0.226894171	9.154424865	0.002481173	0.02208827	-0.489407866	0.207316148	0.471574783	0.466186565	-0.095747566
ENSG00000143179	UCK2	uridine-cytidine kinase 2 [Source:HGNC Symbol;Acc:12562]	-1.135311312	4.251445314	9.153416847	0.002482541	0.022088698	4.736135045	4.738665556	3.083673126	3.568398624	4.40953014
ENSG00000007237	GAS7	growth arrest-specific 7 [Source:HGNC Symbol;Acc:4169]	1.282955569	7.451086894	9.142811803	0.002496971	0.022205837	6.077782981	6.45599335	7.0188414	8.386986655	7.993631947
ENSG00000196502	SULT1A1	sulfotransferase family, cytosolic, 1A, phenol-preferring, member 1 [Source:HGNC Symbol;Acc:11453]	1.075098157	5.409474626	9.140319894	0.002500374	0.022217238	4.646747126	5.309475856	5.763940308	6.093859817	4.636019409
ENSG00000124357	NAGK	N-acetylglucosamine kinase [Source:HGNC Symbol;Acc:17174]	1.176462191	7.27994503	9.140019821	0.002500784	0.022217238	6.264238088	6.749924085	7.355142903	8.138454021	7.197336102
ENSG00000151151	IPMK	inositol polyphosphate multikinase [Source:HGNC Symbol;Acc:20739]	1.303491346	4.531499149	9.132064555	0.002511681	0.022288225	3.499792625	4.056192383	4.172404344	5.238533272	4.983021284
ENSG00000198853	RUSC2	RUN and SH3 domain containing 2 [Source:HGNC Symbol;Acc:23625]	1.54982215	3.939015663	9.133195505	0.002510129	0.022288225	2.80506858	3.171477064	3.186096972	4.846237689	4.494710155
ENSG00000090339	ICAM1	intercellular adhesion molecule 1 [Source:HGNC Symbol;Acc:5344]	1.595200045	5.830362037	9.131407757	0.002512583	0.022288225	4.633778887	4.932612871	4.849585219	6.966934515	6.227077216
ENSG00000132763	MMACHC	methylmalonic aciduria (cobalamin deficiency) cblC type, with homocystinuria [Source:HGNC Symbol;Acc:24525]	-1.478536188	2.333899527	9.124894273	0.002521545	0.022354213	3.080997749	3.011429331	1.732719555	0.074465887	2.006486965
ENSG00000228327	RP11-206L10.2		1.680404601	0.2818553	9.124151289	0.002522569	0.022354213	-0.094677427	-0.112176905	-0.10933503	0.74852395	0.415212929
ENSG00000149564	ESAM	endothelial cell adhesion molecule [Source:HGNC Symbol;Acc:17474]	1.487349241	3.49335888	9.102193808	0.002553031	0.022612744	2.003866175	3.180629157	3.434103115	4.292580805	3.647960442
ENSG00000165097	KDM1B	lysine (K)-specific demethylase 1B [Source:HGNC Symbol;Acc:21577]	1.098666045	6.168504713	9.099155191	0.002557276	0.022638919	5.282302799	5.739229248	6.125883162	6.831264034	6.380723913
ENSG00000197353	LYPD2	LY6/PLAUR domain containing 2 [Source:HGNC Symbol;Acc:25215]	1.826636812	1.260230892	9.093102418	0.002565753	0.022702515	-0.306880589	1.184348258	1.613958159	2.267710332	-0.506781561
ENSG00000067177	PHKA1	phosphorylase kinase, alpha 1 (muscle) [Source:HGNC Symbol;Acc:8925]	-1.912446419	0.672409077	9.088230301	0.002572597	0.022718378	1.531025871	1.434365866	-3.233942493	-2.0191864	0.791775511
ENSG00000146072	TNFRSF21	tumor necrosis factor receptor superfamily, member 21 [Source:HGNC Symbol;Acc:13469]	-1.445573115	2.412642454	9.087497806	0.002573627	0.022718378	2.764850286	2.492318033	-0.215316613	0.669468625	3.524590078
ENSG00000213513	RP13-39P12.2		1.303412354	3.338443766	9.087221559	0.002574016	0.022718378	2.702339162	2.871498754	3.222071445	4.017298632	3.45217082
ENSG00000229980	TOB1-AS1	TOB1 antisense RNA 1 [Source:HGNC Symbol;Acc:44340]	1.66706249	0.153854652	9.090869576	0.002568887	0.022718378	0.000762307	-0.30308518	0.538319988	-0.296766077	0.303422373
ENSG00000272993	RP11-196G18.24		1.773426084	0.430857773	9.08740352	0.00257376	0.022718378	0.174562846	-0.932821174	0.934997507	0.61425099	0.485200289
ENSG00000173599	PC	pyruvate carboxylase [Source:HGNC Symbol;Acc:8636]	-1.190654456	3.577197495	9.079418355	0.002585023	0.022792602	4.069366531	4.327378419	3.066814926	2.27662903	3.215315242
ENSG00000101751	POLI	polymerase (DNA directed) iota [Source:HGNC Symbol;Acc:9182]	1.003392728	4.864836606	9.079845757	0.002584418	0.022792602	4.281404562	4.627606002	5.205848971	5.22592584	4.750573979

ENSG00000179593	ALOX15B	arachidonate 15-lipoxygenase, type B [Source:HGNC Symbol;Acc:434]	1.843387948	0.217545525	9.074901966	0.002591415	0.022837492	-0.775407551	0.207316148	-0.453950945	1.006157955	0.04992943
ENSG00000214193	SH3D21	SH3 domain containing 21 [Source:HGNC Symbol;Acc:26236]	1.618888273	1.124373141	9.069349909	0.002599295	0.02288396	0.566455146	0.715367718	0.75024962	1.732261721	1.318159404
ENSG00000260136	CTD-2270L9.4		1.770597151	-0.032192441	9.041848461	0.002638687	0.023207491	-0.555769858	-0.932821174	-0.66302287	0.335717742	0.615745481
ENSG00000198924	DCLRE1A	DNA cross-link repair 1A [Source:HGNC Symbol;Acc:17660]	-1.059384532	4.223212762	9.030125178	0.002655663	0.023333415	4.775464577	4.723090122	3.523307974	3.253048002	4.191344602
ENSG00000133246	PRAM1	PML-RARA regulated adaptor molecule 1 [Source:HGNC Symbol;Acc:30091]	1.245465611	7.01126196	9.030378336	0.002655295	0.023333415	5.819289062	6.390947726	6.75158165	7.876833484	7.335780113
ENSG00000186642	PDE2A	phosphodiesterase 2A, cGMP-stimulated [Source:HGNC Symbol;Acc:8777]	1.609617428	0.238142965	9.025442389	0.002662475	0.023381563	-0.250832646	0.376157587	-0.10933503	0.527247606	0.139468177
ENSG00000162881	OXER1	oxoeicosanoid (OXE) receptor 1 [Source:HGNC Symbol;Acc:24884]	1.669821813	2.007231441	9.023441746	0.00266539	0.023395465	0.742455703	1.565913615	1.703933103	2.842914522	2.247064062
ENSG00000244480	AC005154.7		1.48466496	1.3209449	9.022468316	0.00266681	0.023396229	0.597320328	1.09035546	1.829176068	1.745170531	0.735452007
ENSG00000225241	RP11-640M9.2		1.030122225	5.259730361	9.018082945	0.002673216	0.023429011	4.59607123	5.313641668	5.988667006	5.655776828	3.743871718
ENSG00000173166	RAPH1	Ras association (RalGDS/AF-6) and pleckstrin homology domains 1 [Source:HGNC Symbol;Acc:14436]	1.408462799	2.324057669	9.018172604	0.002673085	0.023429011	1.431768352	2.048255313	2.490973667	3.001949214	2.098677261
ENSG00000176463	SLCO3A1	solute carrier organic anion transporter family, member 3A1 [Source:HGNC Symbol;Acc:10952]	1.040758052	5.766051025	9.013128578	0.002680472	0.023480881	4.641203577	5.34348935	5.814469071	6.362951298	6.08075335
ENSG00000262370	RP11-473M20.9		1.297466999	3.469414404	9.010817965	0.002683863	0.023498859	2.863368893	2.899483554	3.447189363	4.268193945	3.348020491
ENSG00000199804	RNA5SP383	RNA, 5S ribosomal pseudogene 383 [Source:HGNC Symbol;Acc:43283]	1.609604165	0.34516138	9.006055777	0.002690865	0.023536689	-0.306880589	-0.355038393	0.832340008	0.74852395	0.04992943
ENSG00000188312	CENPP	centromere protein P [Source:HGNC Symbol;Acc:32933]	-1.318154362	2.816965598	8.999117667	0.0027011	0.023614446	3.462559037	3.480241792	2.28288562	1.148991974	2.504656975
ENSG00000051128	HOMER3	homer homolog 3 (Drosophila) [Source:HGNC Symbol;Acc:17514]	1.449334915	3.423475603	8.993186002	0.002709881	0.023679426	2.285510849	2.772072928	2.905510557	4.161495642	4.025090588
ENSG00000175489	LRRC25	leucine rich repeat containing 25 [Source:HGNC Symbol;Acc:29806]	1.263758314	7.179222535	8.992049322	0.002711567	0.023682371	5.657547772	6.654206185	7.211703874	7.976158226	7.435824091
ENSG00000259448	RP11-16E12.1		1.488021952	0.921657489	8.982794702	0.002725335	0.023790779	0.254194054	0.468724906	1.304500245	1.0274492	1.090116303
ENSG00000112578	BYSL	bystin-like [Source:HGNC Symbol;Acc:1157]	-1.228576107	3.45675592	8.976485635	0.002734761	0.0238612	4.185638714	3.984842068	2.28288562	2.775303064	3.146787158
ENSG00000126814	TRMT5	tRNA methyltransferase 5 [Source:HGNC Symbol;Acc:23141]	-1.214255405	3.462889559	8.969439424	0.002745328	0.023918623	4.104705577	4.051211317	3.009154251	1.959718487	3.189342067
ENSG00000197872	FAM49A	family with sequence similarity 49, member A [Source:HGNC Symbol;Acc:25373]	1.155894818	6.136921328	8.969371611	0.00274543	0.023918623	5.311680775	5.375723561	5.871400925	6.899250526	6.53047388
ENSG00000239653	PSMD6-AS2	PSMD6 antisense RNA 2 [Source:HGNC Symbol;Acc:44125]	1.295835129	2.587144256	8.970585444	0.002743607	0.023918623	1.871242652	2.416874535	3.227138203	2.996561217	1.774613685
ENSG00000180304	OAZ2	ornithine decarboxylase antizyme 2 [Source:HGNC Symbol;Acc:8096]	1.174264041	6.370831791	8.965010317	0.002751991	0.023952013	5.45338237	5.805066297	6.144079358	7.198093666	6.588265736
ENSG00000154608	CEP170P1	centrosomal protein 170kDa pseudogene 1 [Source:HGNC Symbol;Acc:28364]	1.79416759	0.257174145	8.965914379	0.00275063	0.023952013	0.254194054	-0.583862591	0.471574783	0.642123966	-0.147769603
ENSG00000188897	CTD-3088G3.8	Protein LOC388210 [Source:UniProtKB/TrEMBL;Acc:I3L099]	1.583539554	4.329515705	8.962971318	0.002755065	0.023966877	2.907138859	2.217877931	2.827591492	4.688107788	5.788927056
ENSG00000272825	L21NC02-1C16.2		1.694179975	1.39607848	8.96025128	0.002759169	0.023990698	-0.14487434	1.010487126	1.265630948	1.719236363	2.098677261
ENSG00000270640	RP11-373D23.2		2.123292874	0.289714207	8.959138122	0.002760851	0.023993436	0.046214424	-1.289265519	-0.390491059	1.261086007	0.303422373
ENSG00000271288	IGHV1OR15-3	immunoglobulin heavy variable 1/OR15-3 (pseudogene) [Source:HGNC Symbol;Acc:5565]	-1.916356516	0.395751655	8.950742751	0.002773568	0.024092026	1.546924617	0.740340508	-2.845623393	-1.572251189	0.303422373
ENSG00000076356	PLXNA2	plexin A2 [Source:HGNC Symbol;Acc:9100]	1.363250294	2.772284264	8.946488126	0.002780035	0.024126601	1.992300831	2.048255313	2.604351095	3.535338275	3.016948344
ENSG00000144802	NFKBIZ	nuclear factor of kappa light polypeptide gene enhancer in B-cells inhibitor, zeta [Source:HGNC Symbol;Acc:29805]	1.493267424	7.79111781	8.943746266	0.002784211	0.02414863	7.070843133	6.756059515	7.535979298	8.901073491	7.650929943
ENSG00000253729	PRKDC	protein kinase, DNA-activated, catalytic polypeptide [Source:HGNC Symbol;Acc:9413]	-1.157875194	7.475180134	8.940095166	0.002789782	0.024184998	7.630820357	8.154531063	7.207544162	6.544677003	7.366818121
ENSG00000160111	CPAMD8	C3 and PZP-like, alpha-2-macroglobulin domain containing 8 [Source:HGNC Symbol;Acc:23228]	1.351443714	3.219645943	8.93331443	0.002800158	0.024262964	1.682639457	2.882757898	3.712221583	3.243981866	3.718670245
ENSG00000175048	ZDHHC14	zinc finger, DHHC-type containing 14 [Source:HGNC Symbol;Acc:20341]	-1.13112288	3.632355805	8.926757238	0.002810228	0.024328475	4.167756273	4.161637805	2.873554121	2.913229148	3.456532906
ENSG00000143224	PPOX	protoporphyrinogen oxidase [Source:HGNC Symbol;Acc:9280]	-1.049962636	5.516347002	8.926587486	0.00281049	0.024328475	5.994726982	6.155955215	5.406773353	4.45852833	4.861640106
ENSG00000092964	DPYSL2	dihydropyrimidinase-like 2 [Source:HGNC Symbol;Acc:3014]	1.11507959	5.843032792	8.9254171	0.002812291	0.024332071	4.543550295	5.375723561	5.705236819	6.380638865	6.433125028
ENSG00000244617	ASPRV1	aspartic peptidase, retroviral-like 1 [Source:HGNC Symbol;Acc:26321]	1.624951983	1.204494603	8.924107012	0.002814309	0.024337537	0.502670228	0.498306264	1.030832979	2.003353492	1.239342106

ENSG00000082781	ITGB5	integrin, beta 5 [Source:HGNC Symbol;Acc:6160]	1.408412359	4.071160304	8.918946268	0.002822273	0.024394389	2.863368893	3.534789485	3.63044747	5.00358354	4.352157627
ENSG00000133812	SBF2	SET binding factor 2 [Source:HGNC Symbol;Acc:2135]	1.048768013	4.954318698	8.915682221	0.002827322	0.024413986	3.903304094	4.559078681	5.044438545	5.439532733	5.315123071
ENSG00000131781	FMO5	flavin containing monooxygenase 5 [Source:HGNC Symbol;Acc:3773]	1.323320524	3.418748665	8.915875224	0.002827023	0.024413986	2.606977165	2.813846027	3.196467253	4.019956698	3.876749164
ENSG00000152926	ZNF117	zinc finger protein 117 [Source:HGNC Symbol;Acc:12897]	1.196001365	4.701606078	8.900751533	0.002850533	0.024602315	4.044383867	4.16855421	4.437959236	5.309777588	5.087965869
ENSG00000248930	CTC-250P20.1		1.205320467	3.836460425	8.892494442	0.002863453	0.024696293	3.196372651	3.494990685	3.761945509	4.261470811	4.167595364
ENSG00000267519	MIR24-2	microRNA 24-2 [Source:HGNC Symbol;Acc:31608]	1.576660604	4.643368995	8.891995384	0.002864235	0.024696293	4.136575339	3.180629157	3.966655493	5.719302866	4.892553786
ENSG00000254294	IMPDH1P6	IMP (inosine monophosphate) dehydrogenase 1 pseudogene 6 [Source:HGNC Symbol;Acc:33961]	1.50704261	2.299736125	8.889983625	0.002867393	0.024699257	1.696960037	1.537699846	2.039642018	3.04955654	2.537970241
ENSG00000182389	CACNB4	calcium channel, voltage-dependent, beta 4 subunit [Source:HGNC Symbol;Acc:1404]	1.533279846	1.618493931	8.890030101	0.00286732	0.024699257	0.502670228	1.371815184	1.932051102	2.258736156	1.298855074
ENSG00000132334	PTPRE	protein tyrosine phosphatase, receptor type, E [Source:HGNC Symbol;Acc:9669]	1.264724696	7.317037746	8.888854132	0.002869167	0.02470242	6.01558652	6.511448068	6.935782687	8.28680977	7.688964309
ENSG00000224080	UBE2FP1	ubiquitin-conjugating enzyme E2F (putative) pseudogene 1 [Source:HGNC Symbol;Acc:44535]	1.868294691	0.872143358	8.882461419	0.002879231	0.024776912	0.254194054	0.277241192	0.28980717	1.808036846	0.791775511
ENSG00000234025	RP11-257K9.3		-1.880963596	-0.028127266	8.874669727	0.002891545	0.024858512	0.823008709	0.947543428	-2.539967122	-2.419096061	-0.894110176
ENSG00000062716	VMP1	vacuole membrane protein 1 [Source:HGNC Symbol;Acc:29559]	1.279629855	7.203109623	8.874943613	0.002891112	0.024858512	6.143303116	6.406633226	6.854213162	8.137689377	7.520551463
ENSG00000204622	HLA-J	major histocompatibility complex, class I, J (pseudogene) [Source:HGNC Symbol;Acc:4967]	1.658312868	1.473073179	8.872091525	0.002895632	0.024881458	0.534915169	0.664086661	1.007464306	2.285492931	1.882291923
ENSG00000108001	EBF3	early B-cell factor 3 [Source:HGNC Symbol;Acc:19087]	-2.859278057	-0.612720413	8.866709193	0.002904182	0.024942718	0.566455146	0.376157587	-7.002070102	-3.888428289	-3.221241555
ENSG00000226950	DANCR	differentiation antagonizing non-protein coding RNA [Source:HGNC Symbol;Acc:28964]	-1.078902767	4.951431711	8.863627498	0.002909089	0.024972644	5.612931785	5.415029199	4.792623003	3.871861288	4.354496872
ENSG00000185883	ATP6V0C	ATPase, H+ transporting, lysosomal 16kDa, V0 subunit c [Source:HGNC Symbol;Acc:855]	2.26545957	1.198873668	8.857286123	0.002919213	0.025047302	0.714573228	-0.355038393	-0.271377577	2.724061344	0.378903451
ENSG00000124507	PACSIN1	protein kinase C and casein kinase substrate in neurons 1 [Source:HGNC Symbol;Acc:8570]	1.449654489	1.767287174	8.853991144	0.002924487	0.025080299	0.534915169	0.689955027	1.030832979	1.089500161	3.260920169
ENSG00000213081	AC012362.3		1.331867613	2.689460253	8.851310415	0.002928785	0.025104897	1.968888231	2.409108576	2.709464044	3.145180481	2.880764656
ENSG00000069399	BCL3	B-cell CLL/lymphoma 3 [Source:HGNC Symbol;Acc:998]	1.474623777	5.452260594	8.849125167	0.002932294	0.025122705	4.391160537	4.48344012	4.535127562	6.402193005	6.077216872
ENSG00000039560	RAI14	retinoic acid induced 14 [Source:HGNC Symbol;Acc:14873]	-1.733855957	0.750969352	8.847881228	0.002934293	0.025127569	2.093195522	1.418980604	-1.315629595	-1.449512048	-1.3021922
ENSG00000123352	SPATS2	spermatogenesis associated, serine-rich 2 [Source:HGNC Symbol;Acc:18650]	-1.165998363	3.829822053	8.84406686	0.002940432	0.025167861	4.626315788	4.1936344	3.196467253	2.895969157	3.486703703
ENSG00000100106	TRIOBP	TRIO and F-actin binding protein [Source:HGNC Symbol;Acc:17009]	1.266212267	4.130773534	8.842252085	0.002943357	0.025180622	3.331012571	3.731322633	3.996635421	5.020937291	3.955910067
ENSG00000259366	CTD-2647L4.4		1.377441832	2.036588052	8.840294753	0.002946516	0.025184402	1.448792784	1.773719635	2.37706662	2.258736156	2.041752268
ENSG00000255398	HCAR3	hydroxycarboxylic acid receptor 3 [Source:HGNC Symbol;Acc:16824]	1.915843904	3.533865531	8.840200144	0.002946668	0.025184402	3.070036784	1.773719635	2.672796614	4.889030573	3.290541751
ENSG00000268758	EMR4P	egf-like module containing, mucin-like, hormone receptor-like 4 pseudogene [Source:HGNC Symbol;Acc:19240]	1.347158935	2.881909937	8.835144647	0.002954842	0.025241973	2.026722384	2.871498754	3.936039322	2.866735547	1.374569317
ENSG00000164124	TMEM144	transmembrane protein 144 [Source:HGNC Symbol;Acc:25633]	1.780531474	3.066405833	8.819466281	0.002980338	0.025447392	2.156740297	2.244306154	2.085000104	4.183001653	3.319567355
ENSG00000006740	ARHGAP44	Rho GTPase activating protein 44 [Source:HGNC Symbol;Acc:29096]	2.230193086	0.350610315	8.817502724	0.002983547	0.025462406	0.133035942	-0.583862591	-1.886652327	1.914722448	-2.060109799
ENSG00000180879	SSR4	signal sequence receptor, delta [Source:HGNC Symbol;Acc:11326]	-1.079936051	6.976525552	8.815405706	0.002986978	0.025479299	7.628954373	7.163685965	6.555053148	6.414366601	6.764843282
ENSG00000167210	LOXHD1	lipoxygenase homology domains 1 [Source:HGNC Symbol;Acc:26521]	1.575910931	2.306818982	8.802395914	0.003008352	0.025649161	1.57820595	1.912023327	2.1289755	3.579252501	0.415212929
ENSG00000131480	AOC2	amine oxidase, copper containing 2 (retina-specific) [Source:HGNC Symbol;Acc:549]	1.671229379	0.96439663	8.798432563	0.003014895	0.025692463	0.329658892	0.056395802	0.471574783	1.638513559	1.428856327
ENSG00000164932	CTHRC1	collagen triple helix repeat containing 1 [Source:HGNC Symbol;Acc:18831]	-3.344563522	-0.55589229	8.791111435	0.003027018	0.025770755	0.566455146	0.376157587	-4.621297774	-7.002070102	-1.87334355
ENSG00000120254	MTHFD1L	methylenetetrahydrofolate dehydrogenase (NADP+ dependent) 1-like [Source:HGNC Symbol;Acc:21055]	-1.233260978	2.886402604	8.781117005	0.003043648	0.025899774	3.598444871	3.364539756	2.171650019	1.692827451	2.708594515

ENSG00000198910	L1CAM	L1 cell adhesion molecule [Source:HGNC Symbol;Acc:6470]	1.606705342	0.887485552	8.770396798	0.003061588	0.026039818	0.823008709	0.527293245	0.632982468	1.567613022	0.303422373
ENSG00000105514	RAB3D	RAB3D, member RAS oncogene family [Source:HGNC Symbol;Acc:9779]	1.122829586	5.714495805	8.767198818	0.003066961	0.026065693	4.497250847	5.242226174	5.489220774	6.174208769	6.40462488
ENSG00000119900	OGFRL1	opioid growth factor receptor-like 1 [Source:HGNC Symbol;Acc:21378]	1.210142894	7.631382632	8.766280404	0.003068506	0.026065693	6.217818539	7.197329557	7.861107208	8.108172302	8.03067253
ENSG00000105499	PLA2G4C	phospholipase A2, group IVC (cytosolic, calcium-independent) [Source:HGNC Symbol;Acc:9037]	1.698488735	1.081129042	8.765936202	0.003069085	0.026065693	0.92386066	0.343933181	0.9100098	1.86827773	0.676839958
ENSG00000102837	OLFM4	olfactomedin 4 [Source:HGNC Symbol;Acc:17190]	-3.242999553	1.910945517	8.760752992	0.003077819	0.026127235	3.33559629	-1.191564836	-3.766908349	-2.419096061	2.968901188
ENSG00000175556	LONRF3	LON peptidase N-terminal domain and ring finger 3 [Source:HGNC Symbol;Acc:21152]	1.30932808	2.363933198	8.759198706	0.003080444	0.026136872	1.79341104	1.965953212	2.635172888	2.860817063	2.206202022
ENSG00000120694	HSPH1	heat shock 105kDa/110kDa protein 1 [Source:HGNC Symbol;Acc:16969]	-1.044403137	6.240698698	8.756703448	0.003084661	0.026160013	6.579389111	6.906712288	6.072693092	5.073060563	5.935971906
ENSG00000142207	URB1	URB1 ribosome biogenesis 1 homolog (S. cerevisiae) [Source:HGNC Symbol;Acc:17344]	-1.070048976	5.155850522	8.74748445	0.003100294	0.026279897	5.710562827	5.746951348	4.973850979	4.041046746	4.585134766
ENSG00000136295	TTYH3	tweety family member 3 [Source:HGNC Symbol;Acc:22222]	1.189037393	7.314239862	8.74522676	0.003104135	0.026299754	6.161489323	6.809397582	7.244556723	8.08249574	7.562384767
ENSG00000206344	HCG27	HLA complex group 27 (non-protein coding) [Source:HGNC Symbol;Acc:27366]	1.204018083	2.817410756	8.742094327	0.003109472	0.026332261	2.256798335	2.345428436	3.329625449	3.174106579	2.594479232
ENSG00000213731	RAB5CP1	RAB5C, member RAS oncogene family pseudogene 1 [Source:HGNC Symbol;Acc:45104]	1.509449717	0.73005783	8.733410117	0.003124316	0.026432468	0.292419846	0.171040209	0.9100098	1.048430793	0.791775511
ENSG00000239264	TXNDC5	thioredoxin domain containing 5 (endoplasmic reticulum) [Source:HGNC Symbol;Acc:21073]	-1.446001883	1.922290978	8.732005737	0.003126724	0.026440092	2.900966723	2.46261236	0.602113338	0.58582888	1.410986673
ENSG00000080200	CRYBG3	beta-gamma crystallin domain containing 3 [Source:HGNC Symbol;Acc:34427]	1.220990807	3.72358438	8.729544307	0.003130947	0.026463063	2.659107731	3.538353702	3.948363866	4.122452783	3.905749982
ENSG00000175792	RUVBL1	RuvB-like AAA ATPase 1 [Source:HGNC Symbol;Acc:10474]	-1.00560695	5.109033061	8.722603199	0.00314289	0.026551216	5.612931785	5.54029573	4.761530635	4.142106283	5.001050786
ENSG00000272086	CTD-2186M15.3		1.561331509	1.081208128	8.716653244	0.003153163	0.026625197	0.796654307	0.095631612	1.205290011	1.477660161	1.279288931
ENSG00000272098	AC092299.8		1.963871718	-0.170251123	8.712042804	0.003161148	0.026679783	-0.625332215	-1.507080648	-0.82139248	0.497040107	0.264147325
ENSG00000154874	CCDC144B	coiled-coil domain containing 144B (pseudogene) [Source:HGNC Symbol;Acc:26704]	1.357917603	2.804672224	8.695620992	0.003189754	0.026908283	1.992300831	2.584842899	2.840873769	3.539049283	2.554342782
ENSG00000188483	IER5L	immediate early response 5-like [Source:HGNC Symbol;Acc:23679]	1.386379963	2.308230324	8.693244151	0.003193916	0.026930453	1.361587606	1.508923295	2.985428851	1.891686981	2.906500707
ENSG00000128298	BAIAP2L2	BAI1-associated protein 2-like 2 [Source:HGNC Symbol;Acc:26203]	1.66796886	0.231588412	8.689816588	0.003199928	0.02696819	-0.094677427	0.171040209	1.030832979	-0.797916568	-0.095747566
ENSG00000172594	SMPDL3A	sphingomyelin phosphodiesterase, acid-like 3A [Source:HGNC Symbol;Acc:17389]	1.398363664	3.290776736	8.688708626	0.003201874	0.026971638	2.114689606	2.766005365	2.924350076	3.758653379	4.036790502
ENSG00000182782	HCAR2	hydroxycarboxylic acid receptor 2 [Source:HGNC Symbol;Acc:24827]	2.138918883	2.911418455	8.687834375	0.00320341	0.026971638	2.247099092	1.050973916	1.501363361	4.333756326	2.906500707
ENSG00000184220	CMSS1	cms1 ribosomal small subunit homolog (yeast) [Source:HGNC Symbol;Acc:28666]	-1.214601118	3.272078753	8.682107278	0.003213492	0.027032456	3.998873122	3.765177832	2.780116222	2.035234912	2.900109607
ENSG00000167173	C15orf39	chromosome 15 open reading frame 39 [Source:HGNC Symbol;Acc:24497]	1.200429969	7.298503149	8.681981855	0.003213713	0.027032456	6.332328853	6.727071469	7.206582523	8.222781393	7.265423867
ENSG00000177045	SIX5	SIX homeobox 5 [Source:HGNC Symbol;Acc:10891]	-1.541137235	1.235392165	8.676652763	0.003223124	0.027098633	2.217603867	1.660530868	0.401591237	-0.525689211	0.735452007
ENSG00000168614	NBPF9	neuroblastoma breakpoint family, member 9 [Source:HGNC Symbol;Acc:31991]	1.046577584	4.657170527	8.675608199	0.003224972	0.02710119	3.918660435	4.562583466	5.030014902	5.11219935	4.311798918
ENSG00000049768	FOXP3	forkhead box P3 [Source:HGNC Symbol;Acc:6106]	1.454329365	1.800606237	8.674716577	0.00322655	0.027101479	0.848890306	1.508923295	2.456873398	1.79568083	1.829457167
ENSG00000171954	CYP4F22	cytochrome P450, family 4, subfamily F, polypeptide 22 [Source:HGNC Symbol;Acc:26820]	1.496803725	1.707450806	8.672045158	0.003231284	0.027128256	1.084982398	1.508923295	1.774850102	2.521378548	0.923655414
ENSG00000176812	RP11-629O4.1		-1.50041013	1.229084553	8.670942119	0.00323324	0.027131705	2.146341971	1.867379203	-0.10933503	-0.525689211	0.791775511
ENSG00000159588	CCDC17	coiled-coil domain containing 17 [Source:HGNC Symbol;Acc:26574]	1.508688616	1.734395507	8.666784854	0.003240624	0.027180679	0.97176043	0.881727766	1.534439871	2.420374188	2.109797605
ENSG00000104856	RELB	v-rel avian reticuloendotheliosis viral oncogene homolog B [Source:HGNC Symbol;Acc:9956]	1.082288698	4.800643193	8.657869048	0.003256519	0.027300952	4.27425947	4.317040508	4.752773603	5.45139657	4.861640106
ENSG00000229988	HBBP1	hemoglobin, beta pseudogene 1 [Source:HGNC Symbol;Acc:4828]	-2.797349375	-0.92808049	8.652141176	0.003266772	0.027360777	0.254194054	0.016063025	-3.766908349	-7.002070102	-4.610216385
ENSG00000138061	CYP1B1	cytochrome P450, family 1, subfamily B, polypeptide 1 [Source:HGNC Symbol;Acc:2597]	1.18097505	5.910952403	8.639424192	0.003289654	0.027539282	4.748218804	5.06453179	5.263703476	6.354552101	6.907086147

ENSG00000263142	LRRC37A17P	leucine rich repeat containing 37, member A17, pseudogene [Source:HGNC Symbol;Acc:48365]	1.493313057	0.953353912	8.630980271	0.003304937	0.027654035	0.292419846	1.010487126	1.205290011	1.348272594	0.378903451
ENSG00000243650	RN7SL834P	RNA, 7SL, cytoplasmic 834, pseudogene [Source:HGNC Symbol;Acc:46850]	1.442676796	1.200761644	8.61979605	0.003325291	0.027811091	0.686141241	0.947543428	1.644577544	1.624609464	0.551948963
ENSG00000134548	C12orf39	chromosome 12 open reading frame 39 [Source:HGNC Symbol;Acc:28139]	1.686258418	1.914697271	8.611380442	0.00334069	0.027926578	0.686141241	1.449588786	1.501363361	2.704366551	2.286800555
ENSG00000181885	CLDN7	claudin 7 [Source:HGNC Symbol;Acc:2049]	1.56312512	1.071011971	8.602409327	0.003357186	0.028037772	0.090278201	0.764888368	1.265630948	1.745170531	0.763888603
ENSG00000270069	RP6-99M1.2		2.087991941	1.308938546	8.599053512	0.003363378	0.028076126	0.92386066	0.277241192	-0.059127673	2.572971709	1.044483142
ENSG00000196371	FUT4	fucosyltransferase 4 (alpha (1,3) fucosyltransferase, myeloid-specific) [Source:HGNC Symbol;Acc:4015]	1.146221598	5.344182595	8.59706	0.003367061	0.028093517	4.419503295	4.620897903	4.963262207	6.082461368	5.854852233
ENSG00000215267	AKR1C7P	aldo-keto reductase family 1, member C7, pseudogene [Source:HGNC Symbol;Acc:44681]	1.639039013	0.34174474	8.594723973	0.003371383	0.028116214	0.435934254	-0.157588231	0.6928072	1.006157955	-2.526883755
ENSG00000261559	RP11-1H8.3		-1.440776368	1.794648357	8.588504694	0.003382917	0.0281945	2.480234919	2.320804564	0.250534523	0.497040107	1.958084208
ENSG00000166963	MAP1A	microtubule-associated protein 1A [Source:HGNC Symbol;Acc:6835]	1.565318469	2.827855901	8.587931146	0.003383982	0.0281945	0.94800933	2.126114598	2.107155349	3.100673935	4.007360863
ENSG00000271462	RP11-841C19.4		1.62902441	0.458966949	8.584694883	0.003390002	0.028231254	0.000762307	-0.355038393	0.250534523	1.069111613	0.615745481
ENSG00000065833	ME1	malic enzyme 1, NADP(+)-dependent, cytosolic [Source:HGNC Symbol;Acc:6983]	1.385625381	1.765636497	8.580684789	0.003397476	0.028275068	1.063037713	1.050973916	1.980862385	1.926103707	2.276968566
ENSG00000242349	NPPA-AS1	NPPA antisense RNA 1 [Source:HGNC Symbol;Acc:37635]	1.691940305	1.368576485	8.580144315	0.003398484	0.028275068	0.329658892	0.968831255	1.142314722	2.420374188	0.872340852
ENSG00000004660	CAMKK1	calcium/calmodulin-dependent protein kinase kinase 1, alpha [Source:HGNC Symbol;Acc:1469]	1.249225147	3.67560561	8.577094932	0.003404181	0.028282248	2.659107731	3.538353702	3.840088097	4.263715338	3.601447293
ENSG00000112769	LAMA4	laminin, alpha 4 [Source:HGNC Symbol;Acc:6484]	-2.414106526	-0.073041694	8.552915659	0.003449693	0.028633264	1.268770164	0.789025519	-7.002070102	-2.97427061	-2.526883755
ENSG00000100714	MTHFD1	methylenetetrahydrofolate dehydrogenase (NADP+ dependent) 1, methenyltetrahydrofolate cyclohydrolase, formyltetrahydrofolate synthetase [Source:HGNC Symbol;Acc:7432]	-1.138779097	3.458969445	8.54899088	0.003457138	0.028681503	4.175447334	4.02350373	2.74522259	2.491046607	3.005085735
ENSG00000196189	SEMA4A	sema domain, immunoglobulin domain (Ig), transmembrane domain (TM) and short cytoplasmic domain, (semaphorin) 4A [Source:HGNC Symbol;Acc:10729]	1.125174077	6.661423378	8.546462788	0.003461943	0.028707797	5.631689742	6.126658197	6.440844579	7.350529121	7.090877824
ENSG00000215912	TTC34	tetratricopeptide repeat domain 34 [Source:HGNC Symbol;Acc:34297]	1.487347227	1.077039372	8.543725456	0.003467153	0.028737426	0.566455146	1.010487126	1.802268781	0.823471408	0.615745481
ENSG00000225036	RP11-414B7.1		-1.322797137	2.192121948	8.537751357	0.003478551	0.028806407	3.036645193	2.753793145	1.205290011	0.722650538	1.869263524
ENSG00000112299	VNN1	vanin 1 [Source:HGNC Symbol;Acc:12705]	1.221436808	3.994382938	8.537017998	0.003479953	0.028806407	2.47193131	3.396312155	3.683019014	3.929546638	5.141776451
ENSG00000121933	ADORA3	adenosine A3 receptor [Source:HGNC Symbol;Acc:268]	1.612377088	0.270634841	8.536785151	0.003480398	0.028806407	-0.365194284	-0.112176905	-0.589913612	0.074465887	1.239342106
ENSG00000189184	PCDH18	protocadherin 18 [Source:HGNC Symbol;Acc:14268]	-3.315069839	-0.63929276	8.533641626	0.003486414	0.028842603	0.627538984	0.343933181	-4.621297774	-7.002070102	-4.610216385
ENSG00000181511	RP4-655L22.4		-1.472575807	1.286285087	8.529970591	0.003493453	0.028887223	2.32292636	1.922971788	-0.10933503	-0.194626373	0.378903451
ENSG00000134827	TCN1	transcobalamin I (vitamin B12 binding protein, R binder family) [Source:HGNC Symbol;Acc:11652]	-2.642575946	1.322336417	8.528581593	0.003496121	0.028888611	3.124027351	-0.068151468	-2.287883461	-1.70641174	1.06748014
ENSG00000267009	RP11-120M18.2		1.476362661	0.685939459	8.52816938	0.003496913	0.028888611	0.254194054	0.715367718	1.030832979	1.006157955	-0.201737898
ENSG00000266786	RP1-66C13.3		1.639027483	0.303247723	8.527179721	0.003498815	0.028890729	-0.425964666	-0.157588231	-0.453950945	0.527247606	1.06748014
ENSG00000236017	ASMTL-AS1	ASMTL antisense RNA 1 [Source:HGNC Symbol;Acc:25811]	1.290694489	2.78156798	8.503342902	0.003544946	0.029216679	1.766505254	2.44752696	2.942946747	3.20246409	3.063446788
ENSG00000162148	PPP1R32	protein phosphatase 1, regulatory subunit 32 [Source:HGNC Symbol;Acc:28869]	1.370000104	1.623623715	8.504147183	0.003543379	0.029216679	1.084982398	1.128690501	2.004662384	1.926103707	1.596341512
ENSG00000254726	MEX3A	mex-3 RNA binding family member A [Source:HGNC Symbol;Acc:33482]	-1.655447032	0.817861415	8.49819454	0.003554991	0.029285716	1.871242652	1.387708937	-2.073364206	-0.874832399	0.551948963
ENSG00000171051	FPR1	formyl peptide receptor 1 [Source:HGNC Symbol;Acc:3826]	1.29868533	7.39424073	8.49163339	0.003567834	0.029377729	6.032624381	6.805705246	7.088356766	8.266667368	7.777704609
ENSG00000101856	PGRMC1	progesterone receptor membrane component 1 [Source:HGNC Symbol;Acc:16090]	1.059591021	5.653158205	8.489181545	0.003572645	0.029403554	4.979609644	5.054610117	5.503907621	6.357182107	5.887526399
ENSG00000140548	ZNF710	zinc finger protein 710 [Source:HGNC Symbol;Acc:25352]	1.108949893	5.537334492	8.483320086	0.003584174	0.029470807	4.639350983	5.057096939	5.280860563	6.305828777	5.800125918
ENSG00000100196	KDELR3	KDEL (Lys-Asp-Glu-Leu) endoplasmic reticulum protein retention receptor 3 [Source:HGNC Symbol;Acc:6306]	-2.107027388	0.243563449	8.479149656	0.0035924	0.029516401	1.608823392	0.925936776	-2.845623393	-3.360122781	-1.18885156
ENSG00000159259	CHAF1B	chromatin assembly factor 1, subunit B (p60) [Source:HGNC Symbol;Acc:1911]	-1.338806379	2.096219554	8.478804402	0.003593082	0.029516401	2.744312733	2.759912177	0.983710868	0.402426505	2.109797605

ENSG00000120800	UTP20	UTP20, small subunit (SSU) processome component, homolog (yeast) [Source:HGNC Symbol;Acc:17897]	-1.026937702	4.837572536	8.477059794	0.003596529	0.029530903	5.343889142	5.460824645	4.610737206	3.706806052	4.359164014
ENSG00000111817	DSE	dermatan sulfate epimerase [Source:HGNC Symbol;Acc:21144]	1.089341705	5.220896307	8.475694461	0.00359923	0.029539259	4.198277527	4.592035301	4.883676276	5.715208396	5.946864712
ENSG00000198753	PLXNB3	plexin B3 [Source:HGNC Symbol;Acc:9105]	2.114501764	0.591585338	8.470396183	0.003609728	0.029611577	-1.921450438	0.376157587	-0.453950945	1.224680533	1.3928929
ENSG00000171298	GAA	glucosidase, alpha; acid [Source:HGNC Symbol;Acc:4065]	1.176256091	7.323480286	8.466641112	0.003617188	0.02965891	6.016300463	6.807552595	7.283382293	7.97957602	7.752648931
ENSG00000182287	AP1S2	adaptor-related protein complex 1, sigma 2 subunit [Source:HGNC Symbol;Acc:560]	1.208856402	7.123130811	8.458207799	0.003633998	0.029782833	5.836369856	6.747231608	7.12885143	7.781171224	7.428348591
ENSG00000180263	FGD6	FYVE, RhoGEF and PH domain containing 6 [Source:HGNC Symbol;Acc:21740]	1.476781362	3.722964852	8.452560559	0.003645299	0.02986151	2.412428059	3.194249349	3.175651608	4.582382673	4.191344602
ENSG00000141384	TAF4B	TAF4b RNA polymerase II, TATA box binding protein (TBP)-associated factor, 105kDa [Source:HGNC Symbol;Acc:11538]	-1.176378819	3.727465422	8.448912025	0.003652619	0.02990752	4.464143145	4.30453643	3.403098856	2.387817579	3.05196192
ENSG00000183401	CCDC159	coiled-coil domain containing 159 [Source:HGNC Symbol;Acc:26996]	1.009075419	5.039127975	8.443370523	0.003663766	0.029981554	4.395557368	4.729340488	5.192958906	5.687889473	4.828367502
ENSG00000100024	UPB1	ureidopropionase, beta [Source:HGNC Symbol;Acc:16297]	1.720703717	-0.020037996	8.442720115	0.003665076	0.029981554	-0.942926472	0.016063025	0.28980717	-1.231517591	0.584199832
ENSG00000244041	LINC01011	long intergenic non-protein coding RNA 1011 [Source:HGNC Symbol;Acc:33812]	1.35983638	1.748635437	8.432791681	0.003685141	0.030117625	1.127895066	1.010487126	2.150470541	1.937395883	2.030092686
ENSG00000078814	MYH7B	myosin, heavy chain 7B, cardiac muscle, beta [Source:HGNC Symbol;Acc:15906]	1.597634141	-0.287279617	8.433544428	0.003683616	0.030117625	-0.489407866	0.056395802	0.081791624	-0.46496394	-1.87334355
ENSG00000100077	ADRBK2	adrenergic, beta, receptor kinase 2 [Source:HGNC Symbol;Acc:290]	1.111501101	6.671762444	8.420240951	0.003710666	0.030312119	5.609150748	6.111185334	6.460876785	7.403399412	7.067897368
ENSG00000181751	C5orf30	chromosome 5 open reading frame 30 [Source:HGNC Symbol;Acc:25052]	-1.141212579	3.323053017	8.41709024	0.003717102	0.030341139	4.035959242	3.807148173	2.548775847	2.086853261	3.210157881
ENSG00000166321	NUDT13	nudix (nucleoside diphosphate linked moiety X)-type motif 13 [Source:HGNC Symbol;Acc:18827]	1.487431999	1.857625906	8.416809835	0.003717675	0.030341139	0.92386066	1.593586194	1.932051102	2.412303653	1.920685729
ENSG00000124019	FAM124B	family with sequence similarity 124B [Source:HGNC Symbol;Acc:26224]	-1.379633689	2.291305397	8.412413555	0.003726676	0.030400462	3.231228835	2.678265888	0.832340008	1.006157955	2.206202022
ENSG00000105663	KMT2B	lysine (K)-specific methyltransferase 2B [Source:HGNC Symbol;Acc:15840]	1.539822867	1.692687509	8.399780205	0.003752664	0.030584036	1.287817526	1.184348258	1.450281721	2.452212292	1.564363944
ENSG00000197320	AC060834.2		1.861195958	0.735931563	8.398753952	0.003754783	0.030586077	-1.237502295	0.376157587	1.397324829	0.229488395	1.35601001
ENSG00000081148	IMPG2	interphotoreceptor matrix proteoglycan 2 [Source:HGNC Symbol;Acc:18362]	1.526961758	0.308009692	8.394091021	0.003764427	0.030637236	-0.046168491	0.277241192	0.858697288	-0.009775356	-0.095747566
ENSG00000107104	KANK1	KN motif and ankyrin repeat domains 1 [Source:HGNC Symbol;Acc:19309]	-1.399736175	3.679056263	8.383800452	0.0037858	0.030768387	4.318925344	4.372005251	3.447189363	1.582074621	3.199787516
ENSG00000141068	KSR1	kinase suppressor of ras 1 [Source:HGNC Symbol;Acc:6465]	1.099487696	6.161682184	8.384365215	0.003784624	0.030768387	5.312843542	5.63987453	6.002769437	7.112578036	6.034088822
ENSG00000138316	ADAMTS14	ADAM metallopeptidase with thrombospondin type 1 motif, 14 [Source:HGNC Symbol;Acc:14899]	-1.675218819	1.303479036	8.380584061	0.003792505	0.030808621	2.6444045	1.724506406	-0.99931534	-0.589083246	0.415212929
ENSG00000109684	CLNK	cytokine-dependent hematopoietic cell linker [Source:HGNC Symbol;Acc:17438]	2.110853924	0.352597716	8.378225552	0.00379743	0.030834358	0.174562846	-0.647226338	-1.57307192	1.692827451	-0.440394449
ENSG00000248334	WHAMMP2	WAS protein homolog associated with actin, golgi membranes and microtubules pseudogene 2 [Source:HGNC Symbol;Acc:32360]	1.138370133	3.434442486	8.367947479	0.003818967	0.030994899	2.798442909	3.229953478	3.793029032	3.761832794	3.324348684
ENSG00000187990	HIST1H2BG	histone cluster 1, H2bg [Source:HGNC Symbol;Acc:4746]	1.927157968	0.41810547	8.360386753	0.003834889	0.031109743	0.214927798	-1.191564836	0.168628767	1.048430793	0.706443614
ENSG00000074276	CDHR2	cadherin-related family member 2 [Source:HGNC Symbol;Acc:18231]	1.749583585	0.046642724	8.357257182	0.0038415	0.031148974	-0.942926472	0.310972527	0.663204908	-1.133744865	0.139468177
ENSG00000072422	RHOBTB1	Rho-related BTB domain containing 1 [Source:HGNC Symbol;Acc:18738]	1.337416523	3.068233866	8.336374485	0.003885907	0.031494502	2.313663059	2.393450112	2.759281478	3.732962613	3.507874397
ENSG00000270491	RP4-743O11.1		1.540699507	1.236136093	8.334607057	0.003889689	0.031510609	0.534915169	0.583576386	0.934997507	1.679439471	1.788521245
ENSG00000087253	LPCAT2	lysophosphatidylcholine acyltransferase 2 [Source:HGNC Symbol;Acc:26032]	1.092662283	5.641786985	8.331598912	0.003896135	0.031548271	4.551452856	5.375723561	5.585079744	6.214796707	5.956906344
ENSG00000109743	BST1	bone marrow stromal cell antigen 1 [Source:HGNC Symbol;Acc:1118]	1.209993402	5.952422321	8.328274842	0.003903271	0.031591479	4.765307615	5.45329231	5.504950982	6.612289061	6.571270519
ENSG00000179271	GADD45GIP1	growth arrest and DNA-damage-inducible, gamma interacting protein 1 [Source:HGNC Symbol;Acc:29996]	-1.028269861	4.237187603	8.326526376	0.00390703	0.031607329	4.8795764	4.649194385	3.740844752	3.427354029	3.940421563
ENSG00000035862	TIMP2	TIMP metallopeptidase inhibitor 2 [Source:HGNC Symbol;Acc:11821]	1.172086952	6.97354347	8.323006812	0.003914607	0.03161036	5.682950487	6.386010826	6.661348042	7.688143569	7.536349247
ENSG00000259388	CTD-2647E9.1		1.470638203	0.917465798	8.325196004	0.003909892	0.03161036	0.686141241	0.310972527	1.304500245	1.508271876	0.002986071
ENSG00000144214	LYG1	lysozyme G-like 1 [Source:HGNC Symbol;Acc:27014]	1.490228767	0.422349276	8.324125824	0.003912196	0.03161036	0.133035942	0.310972527	0.6928072	0.58582888	-0.045536284

ENSG00000113555	PCDH12	protocadherin 12 [Source:HGNC Symbol;Acc:8657]	1.681715355	0.60126037	8.323623749	0.003913278	0.03161036	-0.425964666	0.812765477	0.602113338	1.048430793	0.264147325
ENSG00000244687	UBE2V1	ubiquitin-conjugating enzyme E2 variant 1 [Source:HGNC Symbol;Acc:12494]	1.334009824	2.214565791	8.318807509	0.003923667	0.031668954	1.682639457	1.997369264	2.474024282	2.924622147	1.35601001
ENSG00000155974	GRIP1	glutamate receptor interacting protein 1 [Source:HGNC Symbol;Acc:18708]	1.61095993	0.331514402	8.312362121	0.003937615	0.031766925	-0.698419327	-0.112176905	-0.059127673	0.61425099	0.997359302
ENSG00000122547	EEPD1	endonuclease/exonuclease/phosphatase family domain containing 1 [Source:HGNC Symbol;Acc:22223]	1.128017875	3.877943256	8.304078557	0.003955615	0.03189748	3.02534068	3.516835107	3.987706675	4.472179951	3.962059208
ENSG00000152661	GJA1	gap junction protein, alpha 1, 43kDa [Source:HGNC Symbol;Acc:4274]	-1.977556806	0.023536406	8.300873174	0.003962602	0.031939057	0.97176043	1.237938318	-4.621297774	-2.205328685	-2.526883755
ENSG00000226137	BAIAP2-AS1	BAIAP2 antisense RNA 1 (head to head) [Source:HGNC Symbol;Acc:44342]	1.218115293	4.742298378	8.297885024	0.003969128	0.031947746	3.317173381	4.329437141	4.571437007	5.341999652	5.290900672
ENSG00000211698	TRGV4	T cell receptor gamma variable 4 [Source:HGNC Symbol;Acc:12289]	1.364731884	1.727179793	8.298545324	0.003967685	0.031947746	0.995126841	1.272590116	1.944409617	1.832435869	2.164148811
ENSG00000259623	RP11-156E6.1		1.136381691	3.718559523	8.295093083	0.003975234	0.031982235	3.326414242	3.364539756	4.08580111	4.400553776	2.8278729
ENSG00000213250	RBMS2P1	RNA binding motif, single stranded interacting protein 2 pseudogene 1 [Source:HGNC Symbol;Acc:30994]	1.407220337	1.94680574	8.284228665	0.003999089	0.032159416	0.97176043	1.403429499	1.829176068	2.249705807	2.610226292
ENSG00000253636	RP11-531A24.5		1.574458692	0.367938189	8.28334304	0.00400104	0.032160373	-0.489407866	0.343933181	0.75024962	0.497040107	0.139468177
ENSG00000236484	RRM2P2	ribonucleotide reductase M2 polypeptide pseudogene 2 [Source:HGNC Symbol;Acc:10454]	-2.29672293	-0.508780137	8.273292438	0.004023248	0.032294523	0.502670228	0.095631612	-3.766908349	-3.888428289	-0.726511138
ENSG00000203602	AL137059.1		1.536242872	0.124437833	8.274054779	0.004021559	0.032294523	-0.489407866	0.056395802	0.75024962	0.301169826	-0.9858258
ENSG00000109927	TECTA	tectorin alpha [Source:HGNC Symbol;Acc:11720]	1.5942378	0.050523186	8.274269585	0.004021084	0.032294523	-0.046168491	-0.464918547	0.036330944	-0.146146183	0.378903451
ENSG00000137752	CASP1	caspase 1, apoptosis-related cysteine peptidase [Source:HGNC Symbol;Acc:1499]	1.158150589	7.426166119	8.266001268	0.004039438	0.032409657	6.238158112	6.926568062	7.389445144	8.122929057	7.755528238
ENSG00000049130	KITLG	KIT ligand [Source:HGNC Symbol;Acc:6343]	-1.86531439	0.314645451	8.264838142	0.004042026	0.032415612	1.482250559	1.289608822	-2.845623393	-2.419096061	-1.55968627
ENSG00000267702	RP11-53B2.2		1.576357854	0.417296858	8.261579946	0.004049287	0.032459012	-0.555769858	0.095631612	0.538319988	0.823471408	0.518960621
ENSG00000162437	RAVER2	ribonucleoprotein, PTB-binding 2 [Source:HGNC Symbol;Acc:25577]	-1.106178508	3.493538914	8.25809348	0.004057071	0.032491737	4.041581121	3.997844747	2.680205034	2.371259556	3.585603588
ENSG00000240505	TNFRSF13B	tumor necrosis factor receptor superfamily, member 13B [Source:HGNC Symbol;Acc:18153]	-1.271119111	3.047099909	8.255843862	0.004062102	0.032517192	4.1728882	3.457831828	2.413082378	1.948600359	1.498200525
ENSG00000211584	SLC48A1	solute carrier family 48 (heme transporter), member 1 [Source:HGNC Symbol;Acc:26035]	-1.021766856	4.466941908	8.245250729	0.004085876	0.032692599	5.048237693	4.977939425	3.626618026	3.857072211	4.235164774
ENSG00000189223	PAX8-AS1	PAX8 antisense RNA 1 [Source:HGNC Symbol;Acc:49271]	1.19693686	4.722013181	8.239208463	0.004099501	0.032779026	3.912537499	4.143030294	4.293278938	5.523413729	5.059572022
ENSG00000211900	IGHJ6	immunoglobulin heavy joining 6 [Source:HGNC Symbol;Acc:5540]	-1.21072839	2.925445905	8.234059565	0.004111147	0.032834934	3.878388807	2.772072928	1.432844522	2.924622147	2.554342782
ENSG00000114491	UMPS	uridine monophosphate synthetase [Source:HGNC Symbol;Acc:12563]	-1.096722766	4.364101272	8.226539394	0.004128217	0.032941298	4.992728541	4.883015316	4.13515342	2.87850017	4.036790502
ENSG00000166548	TK2	thymidine kinase 2, mitochondrial [Source:HGNC Symbol;Acc:11831]	1.136653938	5.258681634	8.22670374	0.004127843	0.032941298	4.457849968	4.683399588	4.88688142	5.958389745	5.69236789
ENSG00000075826	SEC31B	SEC31 homolog B (S. cerevisiae) [Source:HGNC Symbol;Acc:23197]	1.003827557	5.708265849	8.223463129	0.004135221	0.032982192	5.002850287	5.518806299	6.489095485	5.696222699	5.377412319
ENSG00000166562	SEC11C	SEC11 homolog C (S. cerevisiae) [Source:HGNC Symbol;Acc:23400]	-1.049791546	5.21981548	8.222571017	0.004137254	0.032983424	6.012727211	5.431415179	4.864294423	4.283760478	4.894162617
ENSG00000229391	HLA-DRB6	major histocompatibility complex, class II, DR beta 6 (pseudogene) [Source:HGNC Symbol;Acc:4954]	1.326801498	5.375211272	8.2189223	0.004145581	0.033019818	1.819824215	1.306429105	1.689321579	3.065082826	7.568443088
ENSG00000271975	RP11-383J24.6		1.197712325	2.993807935	8.210204301	0.004165546	0.033133739	2.207636582	2.729054134	3.232187228	3.257559794	3.225574983
ENSG00000156414	TDRD9	tudor domain containing 9 [Source:HGNC Symbol;Acc:20122]	1.403446127	4.000509691	8.211020211	0.004163673	0.033133739	2.702339162	3.277647049	3.267044131	4.701415938	4.802899588
ENSG00000082996	RNF13	ring finger protein 13 [Source:HGNC Symbol;Acc:10057]	1.011776312	5.447470519	8.207519919	0.004171713	0.03315275	4.590329104	5.012946303	5.428930958	5.966680081	5.805265449
ENSG00000251002	AE000661.37		1.597713462	0.715632715	8.208025123	0.004170552	0.03315275	0.292419846	0.527293245	0.081791624	1.446384807	0.551948963
ENSG00000173548	SNX33	sorting nexin 33 [Source:HGNC Symbol;Acc:28468]	1.176858248	3.637140257	8.206383322	0.004174327	0.033158513	3.036645193	3.100920657	3.622778389	4.22046047	3.827080113
ENSG00000129007	CALML4	calmodulin-like 4 [Source:HGNC Symbol;Acc:18445]	1.31401549	3.713116691	8.203796206	0.004180283	0.033190808	2.680885373	3.368549783	3.527421294	4.47411968	3.886481022
ENSG00000125505	MBOAT7	membrane bound O-acyltransferase domain containing 7 [Source:HGNC Symbol;Acc:15505]	1.139767564	6.79332009	8.195769764	0.004198818	0.033322894	5.812728526	6.25621489	6.52605315	7.594954242	7.089123035
ENSG00000163737	PF4	platelet factor 4 [Source:HGNC Symbol;Acc:8861]	1.467892238	5.793708941	8.182606809	0.004229394	0.033550388	4.063852094	5.547388326	5.451835903	6.739094142	5.97678233

ENSG00000117281	CD160	CD160 molecule [Source:HGNC Symbol;Acc:17013]	-1.300765503	4.052456121	8.177677797	0.004240902	0.033610008	4.916759578	3.847931966	2.800654358	2.895969157	4.567094213
ENSG00000109458	GAB1	GRB2-associated binding protein 1 [Source:HGNC Symbol;Acc:4066]	-1.29328306	4.333691289	8.1761104	0.004244568	0.033610008	4.8795764	4.932612871	2.532495892	3.886500298	4.319003578
ENSG00000178502	KLHL11	kelch-like family member 11 [Source:HGNC Symbol;Acc:19008]	1.163897649	3.362910631	8.178017283	0.004240108	0.033610008	3.124027351	2.860151049	3.664462604	4.072114777	2.504656975
ENSG00000230715	RP11-274B21.4		1.040939696	4.053123735	8.168334951	0.004262802	0.033739175	3.827228993	3.683837545	4.518691979	4.462441992	3.421261246
ENSG00000142046	TMEM91	transmembrane protein 91 [Source:HGNC Symbol;Acc:32393]	1.216882098	3.581465871	8.157686948	0.004287902	0.033907264	2.723478589	3.234355	3.5025636	4.180627867	3.816937756
ENSG00000038427	VCAN	versican [Source:HGNC Symbol;Acc:2464]	1.737116325	10.22720198	8.147950446	0.004310986	0.03407445	7.955671156	9.10452085	9.708888066	11.01302763	11.16166129
ENSG00000134256	CD101	CD101 molecule [Source:HGNC Symbol;Acc:5949]	1.034864666	5.014668989	8.145359898	0.004317149	0.034092462	3.951879815	4.854706069	5.37174208	5.136898111	5.335397107
ENSG00000241839	PLEKHO2	pleckstrin homology domain containing, family O member 2 [Source:HGNC Symbol;Acc:30026]	1.06938475	6.484337426	8.14385988	0.004320721	0.034105334	5.767646993	5.979072822	6.367022969	7.246560553	6.576289928
ENSG00000115392	FANCL	Fanconi anemia, complementation group L [Source:HGNC Symbol;Acc:20748]	-1.239543064	2.803696167	8.142085691	0.004324951	0.034123376	3.445697229	3.198760988	1.582674799	1.679439471	3.063446788
ENSG00000163803	PLB1	phospholipase B1 [Source:HGNC Symbol;Acc:30041]	1.239067796	4.630689803	8.138584327	0.00433331	0.034163776	3.274841002	3.583904609	3.71943079	5.085802361	5.776768725
ENSG00000163736	PPBP	pro-platelet basic protein (chemokine (C-X-C motif) ligand 7) [Source:HGNC Symbol;Acc:9240]	1.633875211	7.156510639	8.138311001	0.004333963	0.034163776	4.964891797	6.86651611	6.82735608	8.005122308	7.604759649
ENSG00000151726	ACSL1	acyl-CoA synthetase long-chain family member 1 [Source:HGNC Symbol;Acc:3569]	1.321927645	7.318095567	8.132216474	0.004348555	0.034263416	6.219059437	6.206115746	6.634427235	8.181382164	8.020396666
ENSG00000165178	NCF1C	neutrophil cytosolic factor 1C pseudogene [Source:HGNC Symbol;Acc:32523]	1.169952143	4.482038407	8.128328841	0.00435789	0.034321558	3.915602215	3.856523346	4.218927114	5.233961488	4.679583759
ENSG00000259340	CTD-2027G2.1		-1.550909367	0.44814655	8.123474765	0.004369573	0.03439814	1.431768352	1.010487126	-1.202308962	-0.874832399	-0.257803757
ENSG00000214954	LRRC69	leucine rich repeat containing 69 [Source:HGNC Symbol;Acc:34303]	1.55365244	0.248678634	8.111575767	0.004398348	0.034593635	-0.14487434	0.277241192	1.053829154	-0.244792505	-0.649489103
ENSG00000110777	POU2AF1	POU class 2 associating factor 1 [Source:HGNC Symbol;Acc:9211]	-1.050406318	5.979655561	8.109795571	0.004402669	0.034596624	6.684189364	6.099772366	5.54810444	5.384904003	5.788061994
ENSG00000105339	DENND3	DENN/MADD domain containing 3 [Source:HGNC Symbol;Acc:29134]	1.278327821	7.093394091	8.11012141	0.004401878	0.034596624	6.113586324	6.14664474	6.541897341	8.072447923	7.492415741
ENSG00000161960	EIF4A1	eukaryotic translation initiation factor 4A1 [Source:HGNC Symbol;Acc:3282]	1.162607435	5.154279529	8.108814965	0.004405051	0.034599856	4.715181607	4.419164558	4.981367087	6.191406778	4.668345358
ENSG00000141526	SLC16A3	solute carrier family 16 (monocarboxylate transporter), member 3 [Source:HGNC Symbol;Acc:10924]	1.14062405	6.58885029	8.10555608	0.004412978	0.034631127	5.7333209	5.90024216	6.220380616	7.330861481	7.050328387
ENSG00000151117	TMEM86A	transmembrane protein 86A [Source:HGNC Symbol;Acc:26890]	1.400979391	1.895445125	8.10583764	0.004412293	0.034631127	1.040754067	1.371815184	1.868618824	2.412303653	2.236956541
ENSG00000172159	FRMD3	FERM domain containing 3 [Source:HGNC Symbol;Acc:24125]	1.240856005	3.071004615	8.098231773	0.004430846	0.034735418	1.920891399	2.557000471	2.979436032	3.531617698	3.659358136
ENSG00000124713	GNMT	glycine N-methyltransferase [Source:HGNC Symbol;Acc:4415]	1.522374521	0.796185185	8.099064305	0.004428812	0.034735418	0.133035942	0.310972527	0.663204908	1.493047208	0.735452007
ENSG00000185482	STAC3	SH3 and cysteine rich domain 3 [Source:HGNC Symbol;Acc:28423]	1.189348888	4.001798112	8.095946039	0.004436438	0.034737583	3.075527676	3.494990685	3.768911065	4.659413665	4.407278515
ENSG00000270060	RP11-390K5.6		1.378448589	1.20623569	8.094293955	0.004440483	0.03475376	0.742455703	0.859104642	1.774850102	1.43048915	0.735452007
ENSG00000162685	LSP1P3	lymphocyte-specific protein 1 pseudogene 3 [Source:HGNC Symbol;Acc:39718]	1.150084141	3.649011159	8.086053727	0.004460719	0.034882727	2.943627224	3.446494941	3.879237643	4.241111357	3.352708597
ENSG00000148926	ADM	adrenomedullin [Source:HGNC Symbol;Acc:259]	1.805173877	3.870870798	8.085965691	0.004460935	0.034882727	2.996685683	2.278807152	2.465474326	5.007606819	4.481928444
ENSG00000073331	ALPK1	alpha-kinase 1 [Source:HGNC Symbol;Acc:20917]	1.175419847	5.011843186	8.084836361	0.004463716	0.03488893	4.131312362	4.279198377	4.499975339	5.683704741	5.646332579
ENSG00000139668	WDFY2	WD repeat and FYVE domain containing 2 [Source:HGNC Symbol;Acc:20482]	1.029713412	5.170476163	8.080386376	0.00447469	0.034912529	4.513524074	4.696219752	5.122649699	5.835518293	5.288455913
ENSG00000142733	MAP3K6	mitogen-activated protein kinase kinase kinase 6 [Source:HGNC Symbol;Acc:6858]	1.158209069	3.876091039	8.08156476	0.004471782	0.034912529	3.075527676	3.53121644	3.888861257	4.499101663	3.974279416
ENSG00000228486	LINC01125	long intergenic non-protein coding RNA 1125 [Source:HGNC Symbol;Acc:49272]	1.321838056	2.041321402	8.082452382	0.004469592	0.034912529	1.306616686	1.844528154	2.74522259	2.045707388	1.774613685
ENSG00000236871	LINC00106	long intergenic non-protein coding RNA 106 [Source:HGNC Symbol;Acc:31843]	1.420390686	2.014875049	8.081015196	0.004473138	0.034912529	0.823008709	1.634124584	2.039642018	2.521378548	2.381602043
ENSG00000261471	RP11-61F12.1		1.949165514	0.244253453	8.074182606	0.004490036	0.035016693	-0.094677427	-1.100062965	-0.520331194	1.187332556	0.341656497
ENSG00000109534	GAR1	GAR1 ribonucleoprotein [Source:HGNC Symbol;Acc:14264]	-1.145110883	3.038195269	8.073256661	0.004492331	0.035019034	3.694354129	3.651289677	2.223277091	2.056104393	2.686482162
ENSG00000111321	LTBR	lymphotoxin beta receptor (TNFR superfamily, member 3) [Source:HGNC Symbol;Acc:6718]	1.2857299	6.033472217	8.062105145	0.004520064	0.035219581	4.720448566	5.490566773	5.451835903	6.858621547	6.586276659
ENSG00000109099	PMP22	peripheral myelin protein 22 [Source:HGNC Symbol;Acc:9118]	-1.51100809	1.71147904	8.060802243	0.004523315	0.03522928	2.636996308	2.144940142	-0.82139248	0.301169826	1.869263524

ENSG00000204010	IFIT1B	interferon-induced protein with tetratricopeptide repeats 1B [Source:HGNC Symbol;Acc:23442]	-1.762613278	1.375001244	8.056449626	0.004534195	0.035296085	1.623891846	2.345428436	0.437007322	-3.360122781	1.612068369
ENSG00000239969	RP11-163E9.2		1.485096527	0.133395328	8.055762885	0.004535914	0.035296085	-0.046168491	0.016063025	0.401591237	0.265774258	-0.506781561
ENSG00000267904	CTC-429P9.5		1.361074441	1.603328301	8.054857403	0.004538181	0.035298084	0.94800933	1.464652754	2.051115931	1.844482366	1.318159404
ENSG00000106348	IMPDH1	IMP (inosine 5'-monophosphate) dehydrogenase 1 [Source:HGNC Symbol;Acc:6052]	1.034927777	5.966683451	8.050811252	0.004548328	0.035361338	5.249887894	5.51157143	5.841977792	6.638903288	6.164461608
ENSG00000123384	LRP1	low density lipoprotein receptor-related protein 1 [Source:HGNC Symbol;Acc:6692]	1.619063863	8.92003214	8.048217779	0.004554844	0.035396321	7.265666822	8.062536685	8.328098466	10.06644237	9.245103828
ENSG00000161643	SIGLEC16	sialic acid binding Ig-like lectin 16 (gene/pseudogene) [Source:HGNC Symbol;Acc:24851]	1.488048435	1.776674899	8.045054367	0.004562805	0.035442496	0.657137645	1.523383318	1.881530361	2.50629229	1.596341512
ENSG00000103642	LACTB	lactamase, beta [Source:HGNC Symbol;Acc:16468]	1.226840146	5.070892105	8.040772329	0.004573603	0.035510661	4.33045325	4.574783594	4.677037216	5.834763651	5.363542761
ENSG00000181418	DDN	dendrin [Source:HGNC Symbol;Acc:24458]	-1.751953858	0.297732797	8.036162316	0.004585258	0.035585411	1.514949964	0.812765477	-2.539967122	-1.449512048	-0.506781561
ENSG00000226124	FTCDNL1	formiminotransferase cyclodeaminase N-terminal like [Source:HGNC Symbol;Acc:48661]	1.529809882	0.528629328	8.03279507	0.00459379	0.035635871	0.401371743	0.133828548	0.437007322	0.823471408	0.485200289
ENSG00000196421	LINC00176	long intergenic non-protein coding RNA 176 [Source:HGNC Symbol;Acc:27655]	1.745234523	-0.22903102	8.030842113	0.004598745	0.035642814	-0.555769858	-1.289265519	0.28980717	-1.042179808	0.303422373
ENSG00000230204	FTH1P5	ferritin, heavy polypeptide 1 pseudogene 5 [Source:HGNC Symbol;Acc:3996]	1.622350426	1.191739769	8.024088157	0.004615926	0.035760185	0.627538984	0.637745977	0.778135316	1.937395883	1.318159404
ENSG00000255524	NPIPB8	nuclear pore complex interacting protein family, member B8 [Source:HGNC Symbol;Acc:37490]	1.453731189	0.339892032	8.00911922	0.004654237	0.036025189	0.090278201	0.310972527	0.778135316	0.335717742	-0.376928291
ENSG00000168010	ATG16L2	autophagy related 16-like 2 (S. cerevisiae) [Source:HGNC Symbol;Acc:25464]	1.253325118	8.318219267	8.002558402	0.00467113	0.036140015	7.274944167	7.463916478	8.17984428	9.165845175	8.643268767
ENSG00000128512	DOCK4	dedicator of cytokinesis 4 [Source:HGNC Symbol;Acc:19192]	1.452177648	1.884930829	7.994192816	0.004692762	0.036291379	0.214927798	1.050973916	1.432844522	2.003353492	3.05196192
ENSG00000165046	LETM2	leucine zipper-EF-hand containing transmembrane protein 2 [Source:HGNC Symbol;Acc:14648]	1.486783675	1.539922223	7.991625351	0.004699421	0.036314286	1.148882041	0.789025519	1.432844522	1.992567844	1.869263524
ENSG00000269657	AC079210.1	Uncharacterized protein; cDNA FLJ45097 fis, clone BRAWH3031054 [Source:UniProtKB/TrEMBL;Acc:Q6ZSZ7]	1.621680905	0.053645616	7.990931394	0.004701222	0.036314286	-0.856736938	-0.408932621	-0.329705368	0.402426505	0.584199832
ENSG00000226148	SLC25A39P1	solute carrier family 25, member 39 pseudogene 1 [Source:HGNC Symbol;Acc:43859]	-1.116446743	3.760112819	7.987724781	0.004709556	0.036337345	4.316608668	4.530729604	3.329625449	2.412303653	3.173530708
ENSG00000256238	RP11-473N11.2		-1.029029768	6.395719416	7.98687412	0.00471177	0.036337345	6.739714253	6.884480586	6.204412517	5.270136417	6.38129761
ENSG00000164885	CDK5	cyclin-dependent kinase 5 [Source:HGNC Symbol;Acc:1774]	1.502936716	0.785423975	7.987108258	0.00471116	0.036337345	0.174562846	0.242702272	0.505333341	1.314027603	1.090116303
ENSG00000060982	BCAT1	branched chain amino-acid transaminase 1, cytosolic [Source:HGNC Symbol;Acc:976]	-1.007914109	4.168919089	7.980479926	0.004728441	0.036422893	4.557351498	4.605689658	3.506736406	3.188355007	4.411778255
ENSG00000138670	RASGEF1B	RasGEF domain family, member 1B [Source:HGNC Symbol;Acc:24881]	1.752221902	5.417441542	7.97344597	0.004746849	0.036537842	4.360001702	4.349864383	4.0084551	7.071849427	4.445086256
ENSG00000234456	MAGI2-AS3	MAGI2 antisense RNA 3 [Source:HGNC Symbol;Acc:40862]	1.760434832	1.376582556	7.973187816	0.004747526	0.036537842	0.435934254	1.237938318	0.934997507	2.444318394	0.676839958
ENSG00000197498	RPF2	ribosome production factor 2 homolog (S. cerevisiae) [Source:HGNC Symbol;Acc:20870]	-1.226774033	2.471293968	7.971104388	0.004752993	0.036547873	3.118718315	3.129558609	1.644577544	0.894716898	2.32547185
ENSG00000246985	SOCS2-AS1	SOCS2 antisense RNA 1 [Source:HGNC Symbol;Acc:27054]	1.419758467	0.804697986	7.971818317	0.004751119	0.036547873	0.365960855	0.527293245	1.285196495	0.434658742	0.973207307
ENSG00000100979	PLTP	phospholipid transfer protein [Source:HGNC Symbol;Acc:9093]	-1.315349271	2.421758282	7.966585023	0.004764875	0.036607167	3.312530659	2.905015959	0.9100098	1.508271876	2.120832889
ENSG00000248166	AC008984.2		1.635681938	1.536040682	7.96671316	0.004764538	0.036607167	0.401371743	1.109650305	0.505333341	2.066427005	2.363133567
ENSG00000230191	RP4-725G10.3		1.700508333	-0.107316299	7.949798637	0.004809273	0.036932101	-1.034594152	-0.355038393	-0.390491059	0.335717742	0.095393329
ENSG00000232549	SRD5A1P1	steroid-5-alpha-reductase, alpha polypeptide 1 pseudogene 1 (3-oxo-5 alpha-steroid delta 4-dehydrogenase alpha pseudogene) [Source:HGNC Symbol;Acc:11286]	1.358703693	2.029556993	7.94810887	0.004813765	0.036950437	1.361587606	1.620737955	1.992811462	2.444318394	2.3444256
ENSG00000224066	RP4-622L5.7		1.626615112	0.686392001	7.946811362	0.004817218	0.036960777	-0.489407866	0.555709266	1.550698027	0.556835562	0.264147325
ENSG00000167566	NCKAP5L	NCK-associated protein 5-like [Source:HGNC Symbol;Acc:29321]	1.110749826	5.216280293	7.945156857	0.004821624	0.036978422	4.307304582	4.864677616	4.957176402	5.895369469	5.525933363
ENSG00000183307	CECR6	cat eye syndrome chromosome region, candidate 6 [Source:HGNC Symbol;Acc:1844]	1.300359025	2.816222091	7.944283766	0.004823951	0.036980111	1.148882041	2.208959833	2.80743594	2.762662248	3.843827185

ENSG00000253676	TAGLN2P1	transgelin 2 pseudogene 1 [Source:HGNC Symbol;Acc:21739]	1.129818386	7.376817317	7.940532268	0.004833961	0.037040678	6.793597635	6.985526048	7.30510805	8.016314125	7.459651659
ENSG00000111199	TRPV4	transient receptor potential cation channel, subfamily V, member 4 [Source:HGNC Symbol;Acc:18083]	1.463851495	1.036211682	7.931385927	0.004858457	0.037212086	0.292419846	1.16603322	1.598401271	0.962608982	0.615745481
ENSG00000182048	TRPC2	transient receptor potential cation channel, subfamily C, member 2, pseudogene [Source:HGNC Symbol;Acc:12334]	1.561723449	0.108126774	7.930598994	0.00486057	0.037212086	-0.250832646	0.056395802	-0.740035668	0.301169826	0.485200289
ENSG00000104763	ASAH1	N-acylsphingosine amidohydrolase (acid ceramidase) 1 [Source:HGNC Symbol;Acc:735]	1.229926946	8.064206839	7.916462208	0.004898697	0.037454977	6.921443401	7.486694125	8.003191697	8.75834514	8.44212934
ENSG00000137449	CPEB2	cytoplasmic polyadenylation element binding protein 2 [Source:HGNC Symbol;Acc:21745]	1.069115371	4.592637933	7.911227999	0.004912891	0.03754299	4.099324826	4.109878245	4.633813507	5.189791339	4.622495289
ENSG00000233261	LINC00264	long intergenic non-protein coding RNA 264 [Source:HGNC Symbol;Acc:17776]	1.47741347	0.718738732	7.910640946	0.004914485	0.03754299	0.214927798	0.133828548	0.805492218	1.348272594	0.485200289
ENSG00000236409	NRADDP	neurotrophin receptor associated death domain, pseudogene [Source:HGNC Symbol;Acc:19337]	1.694123971	0.274490961	7.902278581	0.004937256	0.037700538	-0.306880589	-0.355038393	-0.390491059	0.940331404	0.584199832
ENSG00000247121	CTD-2260A17.2	Uncharacterized protein [Source:UniProtKB/TrEMBL;Acc:D6RER1]	1.353574586	1.407303487	7.895465088	0.004955889	0.037826364	0.92386066	1.03087254	1.718398126	1.493047208	1.564363944
ENSG00000089169	RPH3A	rabphilin 3A homolog (mouse) [Source:HGNC Symbol;Acc:17056]	1.197940245	3.722741211	7.894510455	0.004958506	0.037829886	2.082327188	3.189723558	3.730177502	3.805624207	4.653223332
ENSG00000187068	C3orf70	chromosome 3 open reading frame 70 [Source:HGNC Symbol;Acc:33731]	-2.183073327	-0.021104023	7.874395158	0.005013964	0.038236375	1.210071084	0.947543428	-7.002070102	-2.670141381	-2.274700861
ENSG00000255847	RP11-167N4.2		1.291248553	2.986059845	7.872960401	0.005017944	0.038250109	2.217603867	2.796091168	4.060477069	2.935925879	1.774613685
ENSG00000107954	NEURL1	neuralized E3 ubiquitin protein ligase 1 [Source:HGNC Symbol;Acc:7761]	1.218964437	3.495497481	7.868780444	0.005029556	0.038321988	2.863368893	3.281905652	3.559912356	4.468292652	2.363133567
ENSG00000236279	CLEC2L	C-type lectin domain family 2, member L [Source:HGNC Symbol;Acc:21969]	1.923764135	-0.169918767	7.864305324	0.005042019	0.038400282	-1.921450438	0.277241192	-0.329705368	0.335717742	-0.726511138
ENSG00000114859	CLCN2	chloride channel, voltage-sensitive 2 [Source:HGNC Symbol;Acc:2020]	-1.156025925	2.811049202	7.863458246	0.005044382	0.038401616	3.628640226	3.457831828	2.051115931	1.820287937	1.908000984
ENSG00000111863	ADTRP	androgen-dependent TFPI-regulating protein [Source:HGNC Symbol;Acc:21214]	1.16556131	3.33314078	7.860919863	0.005051469	0.038405601	2.6444045	3.336153071	4.283583984	3.459040145	1.71760268
ENSG00000258539	RP11-12J10.3		1.692969776	0.270830507	7.861098953	0.005050968	0.038405601	0.292419846	-0.782968247	-0.161352853	0.871357159	0.303422373
ENSG00000230633	RP11-37E23.5		1.989296349	0.519374006	7.861680966	0.005049343	0.038405601	-0.625332215	0.056395802	-0.589913612	1.446384807	0.84598249
ENSG00000107736	CDH23	cadherin-related 23 [Source:HGNC Symbol;Acc:13733]	1.085278063	5.629898052	7.858649195	0.005057817	0.038437216	4.438804034	5.536736313	6.289824767	6.137350316	4.93381112
ENSG00000203644	AC083799.1		1.179309438	3.297910008	7.857458059	0.00506115	0.038445904	2.70942011	3.081507914	3.455848046	3.72971886	3.27086176
ENSG00000234271	RP1-138B7.4		-1.316437031	1.872625311	7.852168196	0.00507598	0.038541877	2.730456787	2.671789775	1.007464306	0.466186565	0.676839958
ENSG00000218976	RP11-528A10.1		1.199287828	2.8225333	7.846006116	0.00509331	0.038640045	2.227502762	2.409108576	2.873554121	3.323596289	2.956635145
ENSG00000241399	CD302	CD302 molecule [Source:HGNC Symbol;Acc:30843]	1.168357637	7.078176121	7.841387547	0.00510634	0.038722157	5.73852217	6.694297439	7.011518501	7.666677194	7.54072126
ENSG00000110077	MS4A6A	membrane-spanning 4-domains, subfamily A, member 6A [Source:HGNC Symbol;Acc:13375]	1.36675347	8.181933029	7.839983573	0.005110307	0.03873551	6.894514061	7.551182629	7.907412197	9.005771024	8.611347923
ENSG00000163171	CDC42EP3	CDC42 effector protein (Rho GTPase binding) 3 [Source:HGNC Symbol;Acc:16943]	1.109952447	7.624632271	7.833248342	0.005129384	0.038863327	6.812628406	7.007872409	7.724463834	8.483066949	7.459923264
ENSG00000174130	TLR6	toll-like receptor 6 [Source:HGNC Symbol;Acc:16711]	1.044947035	5.163122463	7.832310177	0.005132047	0.038866729	4.620693019	4.510911363	4.98884424	5.592899069	5.685880671
ENSG00000230551	CTB-89H12.4		1.189233325	4.367910816	7.830154265	0.005138171	0.038896334	3.563702116	4.229353564	4.361684071	5.199203364	3.912116199
ENSG00000147416	ATP6V1B2	ATPase, H+ transporting, lysosomal 56/58kDa, V1 subunit B2 [Source:HGNC Symbol;Acc:854]	1.13240628	7.511957526	7.827645284	0.005145309	0.038921417	6.460091999	7.016842685	7.480306413	8.186862768	7.812790416
ENSG00000189423	USP32P3	ubiquitin specific peptidase 32 pseudogene 3 [Source:HGNC Symbol;Acc:43576]	1.80581802	0.439374854	7.82743088	0.005145919	0.038921417	-0.425964666	0.438524285	-0.589913612	1.39816257	0.095393329
ENSG00000095383	TBC1D2	TBC1 domain family, member 2 [Source:HGNC Symbol;Acc:18026]	1.089516435	5.084181377	7.81445409	0.005183	0.039151277	4.136575339	4.612468678	4.740423765	5.691228542	5.611549358
ENSG00000167618	LAIR2	leukocyte-associated immunoglobulin-like receptor 2 [Source:HGNC Symbol;Acc:6478]	1.484255734	0.401434303	7.814644727	0.005182453	0.039151277	-0.250832646	0.016063025	0.28980717	0.074465887	1.155968757
ENSG00000099338	CATSPERG	catsper channel auxiliary subunit gamma [Source:HGNC Symbol;Acc:25243]	1.334455053	1.484353353	7.81104615	0.005192783	0.039208307	1.249467958	1.255368251	1.919585805	1.820287937	0.706443614
ENSG00000143622	RIT1	Ras-like without CAAX 1 [Source:HGNC Symbol;Acc:10023]	1.029026118	5.710623492	7.809011811	0.005198632	0.039235594	4.985454998	5.155532961	5.500773002	6.318839493	6.111506134
ENSG00000173918	C1QTNF1	C1q and tumor necrosis factor related protein 1 [Source:HGNC Symbol;Acc:14324]	-2.323983033	-0.065174572	7.806287969	0.005206474	0.039256179	1.268770164	0.610915408	-2.539967122	-7.002070102	-1.42520211
ENSG00000154654	NCAM2	neural cell adhesion molecule 2 [Source:HGNC Symbol;Acc:7657]	-1.902896378	-0.474848891	7.805734804	0.005208068	0.039256179	0.597320328	0.207316148	-2.539967122	-2.97427061	-1.55968627

ENSG00000186868	MAPT	microtubule-associated protein tau [Source:HGNC Symbol;Acc:6893]	1.607296077	-0.071970921	7.807190467	0.005203875	0.039256179	-0.489407866	0.056395802	0.570569237	-1.042179808	-0.376928291
ENSG00000215244	RP11-563J2.2		1.506043271	0.611819528	7.802971171	0.00521604	0.039299389	0.46968809	0.095631612	0.6928072	0.773941511	0.676839958
ENSG00000119227	PIGZ	phosphatidylinositol glycan anchor biosynthesis, class Z [Source:HGNC Symbol;Acc:30596]	1.545651509	1.377359065	7.801327195	0.005220787	0.039318285	0.848890306	1.050973916	0.959559782	2.175352965	1.239342106
ENSG00000119121	TRPM6	transient receptor potential cation channel, subfamily M, member 6 [Source:HGNC Symbol;Acc:17995]	2.048479963	1.043530256	7.796942955	0.00523347	0.039380014	0.46968809	-2.076136033	-0.271377577	1.477660161	2.276968566
ENSG00000213073	RP11-288H12.3		1.491305204	2.767448121	7.791463887	0.005249365	0.03946578	2.146341971	2.01793956	2.273120332	3.854096116	2.578558393
ENSG00000211593	IGKJ5	immunoglobulin kappa joining 5 [Source:HGNC Symbol;Acc:5723]	-1.477327069	1.620409624	7.788486059	0.005258024	0.039507354	2.629549878	1.634124584	0.602113338	0.847612951	1.318159404
ENSG00000165029	ABCA1	ATP-binding cassette, sub-family A (ABC1), member 1 [Source:HGNC Symbol;Acc:29]	1.151888392	4.044620277	7.788014806	0.005259395	0.039507354	3.284356125	3.216667762	3.733742023	4.544115616	4.732701701
ENSG00000145780	FEM1C	fem-1 homolog c (C. elegans) [Source:HGNC Symbol;Acc:16933]	1.057522256	5.042732626	7.785865782	0.005265655	0.039537457	4.692133273	4.576518077	5.103490805	5.726643757	4.780457779
ENSG00000206573	SETD5-AS1	SETD5 antisense RNA 1 [Source:HGNC Symbol;Acc:44478]	1.01822561	4.08866944	7.782852797	0.005274444	0.039569601	3.487487892	3.93973528	4.435772465	4.408690123	3.940421563
ENSG00000163694	RBM47	RNA binding motif protein 47 [Source:HGNC Symbol;Acc:30358]	1.114553387	5.635249203	7.775668899	0.00529546	0.039697167	4.582637211	4.983179535	5.150919272	6.356656489	6.232174554
ENSG00000259330	LINC00984	long intergenic non-protein coding RNA 984 [Source:HGNC Symbol;Acc:35165]	1.305219221	3.875779478	7.775494841	0.005295971	0.039697167	3.080997749	3.502308971	3.515045984	4.640538067	4.082663797
ENSG00000254331	CKS1BP7	CDC28 protein kinase regulatory subunit 1B pseudogene 7 [Source:HGNC Symbol;Acc:1999]	-1.438101532	0.959423432	7.76998508	0.005312149	0.039801439	1.957037891	1.449588786	-0.520331194	-0.46496394	0.485200289
ENSG00000131379	C3orf20	chromosome 3 open reading frame 20 [Source:HGNC Symbol;Acc:25320]	1.461791035	0.259750232	7.768161038	0.005317516	0.039824654	-0.14487434	0.498306264	0.602113338	0.402426505	-0.894110176
ENSG00000105371	ICAM4	intercellular adhesion molecule 4 (Landsteiner-Wiener blood group) [Source:HGNC Symbol;Acc:5347]	-1.118070855	3.480748308	7.762226105	0.005335017	0.039937134	4.213299382	4.227146839	2.548775847	2.935925879	2.236956541
ENSG00000182625	RP11-123C21.1		1.48763856	0.642383838	7.761526167	0.005337084	0.039937134	-0.094677427	-0.068151468	0.328039018	1.0274492	1.239342106
ENSG00000105963	ADAP1	ArfGAP with dual PH domains 1 [Source:HGNC Symbol;Acc:16486]	1.170746332	4.56538561	7.755088318	0.005356142	0.040028556	3.743472659	4.102674026	4.218927114	5.320598436	4.855046691
ENSG00000006534	ALDH3B1	aldehyde dehydrogenase 3 family, member B1 [Source:HGNC Symbol;Acc:410]	1.09570381	6.081121015	7.748325852	0.005376236	0.040161625	5.231559854	5.715811808	5.863287843	6.879993772	6.171775716
ENSG00000104067	TJP1	tight junction protein 1 [Source:HGNC Symbol;Acc:11827]	-3.19826732	-0.693484675	7.745786988	0.005383799	0.04016685	0.502670228	0.207316148	-7.002070102	-4.730697034	-2.526883755
ENSG00000131470	PSMC3IP	PSMC3 interacting protein [Source:HGNC Symbol;Acc:17928]	-1.290229393	2.214270661	7.746269198	0.005382362	0.04016685	2.798442909	2.759912177	0.721814282	1.314027603	2.334979851
ENSG00000205181	LINC00654	long intergenic non-protein coding RNA 654 [Source:HGNC Symbol;Acc:27154]	1.53550354	1.407324469	7.746433809	0.005381871	0.04016685	0.686141241	0.925936776	1.098750223	2.33756225	1.177268129
ENSG00000050555	LAMC3	laminin, gamma 3 [Source:HGNC Symbol;Acc:6494]	1.881793876	-0.222945037	7.728655815	0.00543512	0.040532509	-1.608074432	0.407677918	0.721814282	-1.042179808	-2.274700861
ENSG00000108861	DUSP3	dual specificity phosphatase 3 [Source:HGNC Symbol;Acc:3069]	1.072131628	6.118753606	7.726793933	0.005440727	0.0405571	5.066276063	5.423727308	5.666596748	6.677294827	6.878112447
ENSG00000227295	ELL2P1	elongation factor, RNA polymerase II, 2 pseudogene 1 [Source:HGNC Symbol;Acc:39343]	-1.117505598	3.313393007	7.725777733	0.00544379	0.040562709	3.909466259	3.881994713	2.292585253	2.580193821	3.130499786
ENSG00000166900	STX3	syntaxin 3 [Source:HGNC Symbol;Acc:11438]	1.073381583	5.860244558	7.715886138	0.005473699	0.040768255	5.008602417	5.302156613	5.534884824	6.563929961	6.277257837
ENSG00000245213	RP11-10K16.1		1.449655988	0.777302379	7.714070637	0.005479206	0.040791968	0.292419846	0.664086661	0.602113338	1.048430793	0.923655414
ENSG00000121211	MND1	meiotic nuclear divisions 1 homolog (S. cerevisiae) [Source:HGNC Symbol;Acc:24839]	-1.66779886	0.817021319	7.710494618	0.005490071	0.040855528	1.858558824	1.403429499	-1.202308962	-2.670141381	0.735452007
ENSG00000185155	MIXL1	Mix paired-like homeobox [Source:HGNC Symbol;Acc:13363]	-2.10471217	-0.084861063	7.703554497	0.005511219	0.040983158	1.397104685	0.056395802	-3.233942493	-2.670141381	-0.9858258
ENSG00000163739	CXCL1	chemokine (C-X-C motif) ligand 1 (melanoma growth stimulating activity, alpha) [Source:HGNC Symbol;Acc:4602]	2.887807383	2.217509802	7.703334587	0.00551189	0.040983158	1.127895066	-1.763731145	-0.589913612	3.597162784	2.950462792
ENSG00000248281	RP11-174E22.2		1.393562436	2.357910413	7.700316452	0.005521114	0.041034363	1.593595892	1.699255595	1.956663165	2.930285084	2.925505868
ENSG00000054598	FOXC1	forkhead box C1 [Source:HGNC Symbol;Acc:3800]	-1.797621437	-0.028355138	7.696730513	0.005532094	0.041098568	1.343495699	0.498306264	-2.287883461	-2.205328685	-2.060109799
ENSG00000117151	CTBS	chitobiase, di-N-acetyl- [Source:HGNC Symbol;Acc:2496]	1.007997746	5.215371218	7.690764911	0.00555041	0.041217193	4.574904087	4.828743922	5.145577126	5.700371331	5.518650873
ENSG00000031081	ARHGAP31	Rho GTPase activating protein 31 [Source:HGNC Symbol;Acc:29216]	1.01350518	5.417063168	7.689125345	0.005555454	0.041235081	4.679568409	5.005240075	5.329963542	6.123204694	5.513426495
ENSG00000228624	RP3-399L15.3		1.680253311	-0.324593365	7.688455609	0.005557516	0.041235081	-1.132483902	-0.713501401	-1.315629595	0.114810932	0.223773082
ENSG00000146828	SLC12A9	solute carrier family 12, member 9 [Source:HGNC Symbol;Acc:17435]	1.028008309	7.263794152	7.684314659	0.005570283	0.041294897	6.282751708	6.707021551	7.249856471	7.832349215	7.684322605

ENSG00000087589	CASS4	Cas scaffolding protein family member 4 [Source:HGNC Symbol;Acc:15878]	1.079078082	3.456743638	7.681144828	0.005580076	0.041332586	2.651774847	3.251828018	3.833458626	3.635799812	3.601447293
ENSG00000168615	ADAM9	ADAM metallopeptidase domain 9 [Source:HGNC Symbol;Acc:216]	1.128208982	4.933470056	7.681196704	0.005579915	0.041332586	3.924757494	4.347834625	4.424788612	5.460230859	5.685880671
ENSG00000160183	TMPRSS3	transmembrane protease, serine 3 [Source:HGNC Symbol;Acc:11877]	1.617362614	0.461628894	7.677701865	0.005590732	0.041394054	-0.196880952	0.583576386	1.415193986	-0.296766077	-0.376928291
ENSG00000267478	RP11-815J4.5		1.097585751	3.858507606	7.663319298	0.005635473	0.041707728	3.36279692	3.516835107	3.895241519	4.448697471	3.78607572
ENSG00000238142	RP11-108M9.4		1.503801352	2.214269103	7.662175527	0.005639047	0.041716589	1.57820595	1.673554946	1.732719555	3.065082826	2.334979851
ENSG00000102554	KLF5	Kruppel-like factor 5 (intestinal) [Source:HGNC Symbol;Acc:6349]	1.59515714	2.435298075	7.658420248	0.005650796	0.041768306	1.871242652	1.912023327	1.842443831	3.516638688	2.098677261
ENSG00000273018	CTD-2303H24.2		1.551384079	2.028940448	7.657482824	0.005653733	0.041772426	0.714573228	2.01793956	2.085000104	2.697741416	1.882291923
ENSG00000225358	MIPEPP1	mitochondrial intermediate peptidase pseudogene 1 [Source:HGNC Symbol;Acc:39456]	-1.326104003	1.822925461	7.653937135	0.005664856	0.041828827	2.504862786	2.632310473	1.163613388	0.229488395	1.06748014
ENSG00000172123	SLFN12	schlafen family member 12 [Source:HGNC Symbol;Acc:25500]	1.142701248	3.783811837	7.653530629	0.005666132	0.041828827	3.064524914	3.372548695	3.679326777	4.178250168	4.245284812
ENSG00000130066	SAT1	spermidine/spermine N1-acetyltransferase 1 [Source:HGNC Symbol;Acc:10540]	1.887591653	9.342876785	7.647741667	0.005684344	0.041928006	7.99911617	7.824935792	8.041086484	10.66607203	9.774284812
ENSG00000119042	SATB2	SATB homeobox 2 [Source:HGNC Symbol;Acc:21637]	1.522067649	0.766894264	7.64079052	0.00570629	0.042054546	0.254194054	0.376157587	0.036330944	1.314027603	1.155968757
ENSG00000272040	CTC-366B18.4		1.523350013	-0.068334766	7.638866974	0.005712379	0.04208175	-0.625332215	-0.713501401	0.328039018	-0.053815412	0.04992943
ENSG00000099139	PCSK5	proprotein convertase subtilisin/kexin type 5 [Source:HGNC Symbol;Acc:8747]	1.013713279	4.278961546	7.634937166	0.005724838	0.042155843	3.437191842	4.031113272	4.802839979	4.519907545	4.230078009
ENSG00000108352	RAPGEFL1	Rap guanine nucleotide exchange factor (GEF)-like 1 [Source:HGNC Symbol;Acc:17428]	1.305840081	2.581125667	7.633614117	0.005729039	0.042169089	2.103982592	1.986973075	2.430758609	3.275467173	2.656461736
ENSG00000107815	C10orf2	chromosome 10 open reading frame 2 [Source:HGNC Symbol;Acc:1160]	-1.126042874	4.151004819	7.626915143	0.005750357	0.042290542	4.825203682	4.670464478	3.963622948	2.818693563	3.59354719
ENSG00000169087	HSPBAP1	HSPB (heat shock 27kDa) associated protein 1 [Source:HGNC Symbol;Acc:16389]	1.080978165	4.409098803	7.622683307	0.005763866	0.04236872	3.948891281	4.018408312	4.352439515	5.053734345	4.368453247
ENSG00000205560	CPT1B	carnitine palmitoyltransferase 1B (muscle) [Source:HGNC Symbol;Acc:2329]	1.388387542	0.668396048	7.601904907	0.005830667	0.042809432	0.292419846	0.468724906	1.053829154	0.61425099	0.551948963
ENSG00000106268	NUDT1	nudix (nucleoside diphosphate linked moiety X)-type motif 1 [Source:HGNC Symbol;Acc:8048]	-1.059365974	3.69527062	7.599145359	0.005839597	0.042849668	4.074859969	4.312884512	2.800654358	2.952717131	3.704069251
ENSG00000167112	TRUB2	TruB pseudouridine (psi) synthase family member 2 [Source:HGNC Symbol;Acc:17170]	-1.053962334	4.085307615	7.598703575	0.005841029	0.042849668	4.755078638	4.476025242	3.71943079	2.895969157	3.880000416
ENSG00000261269	RP11-389C8.2		1.709784556	0.322210597	7.595002464	0.005853032	0.042919786	-0.856736938	-0.252938016	-0.390491059	0.847612951	1.021113618
ENSG00000138119	MYOF	myoferlin [Source:HGNC Symbol;Acc:3656]	1.080237239	5.226286207	7.579184795	0.005904614	0.043225811	4.154846016	4.917638154	5.086863926	5.975607843	5.390010002
ENSG00000229140	CCDC26	coiled-coil domain containing 26 [Source:HGNC Symbol;Acc:28416]	-1.867357893	-0.053495967	7.576825492	0.005912347	0.043264383	1.063037713	0.836121102	-2.845623393	-2.97427061	-1.42520211
ENSG00000166510	CCDC68	coiled-coil domain containing 68 [Source:HGNC Symbol;Acc:24350]	1.543431711	-0.112763678	7.570550835	0.005932965	0.043397167	-0.306880589	0.242702272	-0.453950945	0.434658742	-2.060109799
ENSG00000166741	NNMT	nicotinamide N-methyltransferase [Source:HGNC Symbol;Acc:7861]	-2.697344812	0.03326299	7.569491973	0.005936451	0.043404584	1.766505254	0.207316148	-3.766908349	-4.730697034	-2.526883755
ENSG00000226535	RP11-417L14.1		-1.198092848	2.723701584	7.565626529	0.005949197	0.043479664	3.571495355	3.225538486	1.674560554	1.86827773	2.216526545
ENSG00000234961	RP11-124N14.3		1.580443006	0.019778585	7.559717809	0.005968734	0.043604295	-1.132483902	-0.157588231	0.401591237	-0.053815412	0.223773082
ENSG00000102471	NDFIP2	Nedd4 family interacting protein 2 [Source:HGNC Symbol;Acc:18537]	-1.177051243	2.544979775	7.557092812	0.005977434	0.04364969	3.191323731	3.216667762	1.868618824	1.12943269	2.206202022
ENSG00000114948	ADAM23	ADAM metallopeptidase domain 23 [Source:HGNC Symbol;Acc:202]	1.610781448	1.414146983	7.549956095	0.006001154	0.043804679	0.174562846	1.237938318	2.448220886	0.773941511	1.279288931
ENSG00000221821	C6orf226	chromosome 6 open reading frame 226 [Source:HGNC Symbol;Acc:34431]	1.204486455	2.329662303	7.547817236	0.006008281	0.043838476	2.093195522	1.955327308	2.548775847	2.629760944	2.226777706
ENSG00000253239	IGLVI-70	immunoglobulin lambda variable (I)-70 (pseudogene) [Source:HGNC Symbol;Acc:5940]	-1.681331022	0.228967283	7.546667288	0.006012117	0.043848238	1.32517403	0.637745977	-1.57307192	-2.205328685	-0.045536284
ENSG00000136630	HLX	H2.0-like homeobox [Source:HGNC Symbol;Acc:4978]	1.082645766	4.31379131	7.543102503	0.006024023	0.043916826	3.687198663	3.392378661	3.939130343	4.826634759	5.004033918
ENSG00000132205	EMILIN2	elastin microfibril interfacer 2 [Source:HGNC Symbol;Acc:19881]	1.107516973	7.311635358	7.53935786	0.006036555	0.043953458	6.003395125	6.716491526	7.056332609	7.956694083	7.926037372
ENSG00000175294	CATSPER1	cation channel, sperm associated 1 [Source:HGNC Symbol;Acc:17116]	1.389077817	2.260798737	7.539356846	0.006036559	0.043953458	1.57820595	1.647388141	1.932051102	3.065082826	2.426764636
ENSG00000212743	DKFZP667F0711		1.586638858	-0.121618055	7.540564764	0.006032513	0.043953458	-0.942926472	-0.523165112	-0.329705368	0.114810932	0.303422373
ENSG00000181649	PHLDA2	pleckstrin homology-like domain, family A, member 2 [Source:HGNC Symbol;Acc:12385]	2.326515384	0.571178613	7.538358358	0.006039905	0.04395959	-0.094677427	-0.647226338	-2.073364206	2.02468586	0.303422373

ENSG00000138095	LRPPRC	leucine-rich pentatricopeptide repeat containing [Source:HGNC Symbol;Acc:15714]	-1.030068866	7.192725713	7.537015826	0.006044407	0.043974127	7.600433037	7.643226642	7.0188414	6.248052403	7.039101795
ENSG00000100804	PSMB5	proteasome (prosome, macropain) subunit, beta type, 5 [Source:HGNC Symbol;Acc:9542]	-1.009470149	4.062709449	7.535315753	0.006050113	0.043982884	4.730925147	4.48158997	3.531522919	3.140302572	3.806723591
ENSG00000181826	RELL1	RELT-like 1 [Source:HGNC Symbol;Acc:27379]	1.303690489	2.46227608	7.535164374	0.006050622	0.043982884	1.89628059	2.028115757	2.386155201	3.221064093	2.296565991
ENSG00000068793	CYFIP1	cytoplasmic FMR1 interacting protein 1 [Source:HGNC Symbol;Acc:13759]	1.013048974	6.05517865	7.531572439	0.006062697	0.044052419	5.042641743	5.601204704	5.839498562	6.705638951	6.476726889
ENSG00000140259	MFAP1	microfibrillar-associated protein 1 [Source:HGNC Symbol;Acc:7032]	-1.026106613	4.049152701	7.517276576	0.006111	0.044348329	4.486986071	4.525351572	3.595610939	2.697741416	4.206962975
ENSG00000115318	LOXL3	lysyl oxidase-like 3 [Source:HGNC Symbol;Acc:13869]	1.293454129	4.025999543	7.516167749	0.006114763	0.0443573	3.019655031	3.55604389	3.519182893	4.791286425	4.46903248
ENSG00000086548	CEACAM6	carcinoembryonic antigen-related cell adhesion molecule 6 (non-specific cross reacting antigen) [Source:HGNC Symbol;Acc:1818]	-1.94923022	1.101171704	7.515189591	0.006118085	0.044360002	2.125317742	1.07079906	-7.002070102	-1.042179808	1.81594051
ENSG00000135722	FBXL8	F-box and leucine-rich repeat protein 8 [Source:HGNC Symbol;Acc:17875]	1.248465063	2.542018027	7.514569977	0.006120189	0.044360002	1.766505254	2.270258944	3.380537789	2.521378548	2.164148811
ENSG00000270681	RP11-372K14.2		1.590204997	0.431441462	7.511133877	0.006131876	0.044426364	0.329658892	-0.204475541	0.505333341	1.206127397	-0.726511138
ENSG00000272520	CTD-2044J15.2		1.307337912	1.625520374	7.505823162	0.006149983	0.044520802	0.848890306	1.202433699	1.932051102	1.820287937	1.895203721
ENSG00000158887	MPZ	myelin protein zero [Source:HGNC Symbol;Acc:7225]	1.315771341	1.814655607	7.502602464	0.00616099	0.044582103	1.084982398	1.289608822	1.919585805	2.346060651	1.958084208
ENSG00000250067	YJEFN3	YjeF N-terminal domain containing 3 [Source:HGNC Symbol;Acc:24785]	1.359191547	1.538712562	7.498709332	0.006174323	0.044660171	1.018120824	1.306429105	2.311791045	1.12943269	1.3928929
ENSG00000272941	RP11-134L10.1		1.325766341	1.951373836	7.497854614	0.006177254	0.044662969	1.16956809	1.620737955	1.968813513	2.354509285	2.236956541
ENSG00000099998	GGT5	gamma-glutamyltransferase 5 [Source:HGNC Symbol;Acc:4260]	-1.604180592	0.787167352	7.496883939	0.006180584	0.044668651	2.177314774	1.434365866	-1.57307192	-1.231517591	-1.55968627
ENSG00000252620	RNU6ATAC24P	RNA, U6atac small nuclear 24, pseudogene [Source:HGNC Symbol;Acc:46923]	1.472775951	0.10630818	7.494701555	0.006188079	0.044704411	-0.489407866	-0.252938016	0.505333341	0.669468625	-0.894110176
ENSG00000228393	LINC01004	long intergenic non-protein coding RNA 1004 [Source:HGNC Symbol;Acc:48961]	1.586911245	0.281789141	7.489056846	0.006207506	0.044826313	0.254194054	-0.523165112	0.505333341	-0.009775356	0.615745481
ENSG00000110092	CCND1	cyclin D1 [Source:HGNC Symbol;Acc:1582]	-1.454572319	1.674223278	7.47829583	0.006244715	0.045057941	2.931566685	2.116608898	-0.161352853	0.58582888	0.378903451
ENSG00000203836	NBPF24	neuroblastoma breakpoint family, member 24 [Source:HGNC Symbol;Acc:27299]	1.523990329	0.388415634	7.478931507	0.006242511	0.045057941	0.534915169	-0.068151468	0.721814282	0.773941511	-0.9858258
ENSG00000100504	PYGL	phosphorylase, glycogen, liver [Source:HGNC Symbol;Acc:9725]	1.069925274	6.745941033	7.473670923	0.006260777	0.045155272	5.610096937	6.068067153	6.308469644	7.299481203	7.521072175
ENSG00000166145	SPINT1	serine peptidase inhibitor, Kunitz type 1 [Source:HGNC Symbol;Acc:11246]	1.008818155	4.519758636	7.471181131	0.006269441	0.045163434	3.628640226	4.159324949	4.463945173	4.853706917	5.053825573
ENSG00000130598	TNNI2	troponin I type 2 (skeletal, fast) [Source:HGNC Symbol;Acc:11946]	1.466729433	2.214374997	7.469650946	0.006274772	0.045163434	1.32517403	1.272590116	1.582674799	2.924622147	2.893690069
ENSG00000205038	PKHD1L1	polycystic kidney and hepatic disease 1 (autosomal recessive)-like 1 [Source:HGNC Symbol;Acc:20313]	1.899408815	2.196894829	7.468754998	0.006277895	0.045167397	0.796654307	1.479561055	0.570569237	3.415289988	2.5705316
ENSG00000198150	AC135178.1	HCG1985372; Uncharacterized protein; cDNA FLJ37541 fis, clone BRCAN2026340 [Source:UniProtKB/TrEMBL;Acc:Q8N1U9]	1.365589291	1.031939691	7.46546339	0.006289384	0.045212996	0.435934254	0.836121102	1.855590689	1.069111613	0.139468177
ENSG00000241370	RPP21	ribonuclease P/MRP 21kDa subunit [Source:HGNC Symbol;Acc:21300]	1.339446762	1.104822168	7.462802185	0.006298688	0.045260071	0.796654307	0.689955027	1.467510679	1.582074621	0.450630967
ENSG00000163221	S100A12	S100 calcium binding protein A12 [Source:HGNC Symbol;Acc:10489]	1.347237856	6.798791255	7.452597695	0.006334496	0.045444251	5.694594263	5.939859011	5.911295764	7.758868473	7.378933837
ENSG00000203709	C1orf132	chromosome 1 open reading frame 132 [Source:HGNC Symbol;Acc:32018]	1.258107485	4.156688407	7.451552068	0.006338177	0.045452084	3.843412177	3.415820346	3.975715067	5.173171083	3.589580856
ENSG00000204420	C6orf25	chromosome 6 open reading frame 25 [Source:HGNC Symbol;Acc:13937]	1.238120195	5.194267403	7.449922923	0.006343917	0.045474665	4.115407246	4.72152329	4.689939282	6.145893276	5.407013282
ENSG00000273295	AP000350.5		1.601100068	0.849885967	7.448182464	0.006350054	0.04550008	-0.698419327	0.637745977	1.225685365	1.006157955	1.155968757
ENSG00000198502	HLA-DRB5	major histocompatibility complex, class II, DR beta 5 [Source:HGNC Symbol;Acc:4953]	1.276392317	6.869055241	7.447264283	0.006353294	0.045504724	5.365342359	5.571941368	5.685125903	7.275767829	8.175136701
ENSG00000254859	RP11-661A12.5		1.524121589	1.057778137	7.443608789	0.006366211	0.045578643	0.566455146	0.836121102	0.959559782	2.22227058	-2.274700861
ENSG00000196782	MAML3	mastermind-like 3 (Drosophila) [Source:HGNC Symbol;Acc:16272]	1.063821465	4.713465058	7.43892219	0.00638281	0.045678857	3.55586655	4.264209695	4.455335096	5.350472031	5.216979501
ENSG00000177301	KCNA2	potassium voltage-gated channel, shaker-related subfamily, member 2 [Source:HGNC Symbol;Acc:6220]	1.531408839	-0.054642369	7.431553041	0.006409001	0.045791618	-0.250832646	0.095631612	0.570569237	-0.956081001	-0.649489103
ENSG00000167378	IRGQ	immunity-related GTPase family, Q [Source:HGNC Symbol;Acc:24868]	1.084321331	4.349950261	7.425768086	0.006429637	0.045903858	3.918660435	3.942428021	4.259057675	5.188610507	3.958987913

ENSG00000272916	RP11-574K11.31	Bifunctional heparan sulfate N-deacetylase/N-sulfotransferase 2 [Source:UniProtKB/TrEMBL;Acc:S4R438]	1.895169426	-0.080107053	7.425683559	0.006429939	0.045903858	-1.921450438	0.376157587	0.28980717	0.229488395	-1.083770229
ENSG00000102362	SYTL4	synaptotagmin-like 4 [Source:HGNC Symbol;Acc:15588]	-1.508716051	2.529138462	7.42029063	0.00644924	0.046008476	3.279606408	3.624712133	-0.271377577	1.926103707	0.872340852
ENSG00000166682	TMPRSS5	transmembrane protease, serine 5 [Source:HGNC Symbol;Acc:14908]	1.453346791	0.026648253	7.420124482	0.006449835	0.046008476	-0.306880589	0.207316148	-0.059127673	0.154058352	-0.376928291
ENSG00000198682	PAPSS2	3'-phosphoadenosine 5'-phosphosulfate synthase 2 [Source:HGNC Symbol;Acc:8604]	1.187849402	2.942744312	7.418345016	0.006456217	0.046016603	2.093195522	2.865836059	3.186096972	3.462952422	2.693890668
ENSG00000095015	MAP3K1	mitogen-activated protein kinase kinase kinase 1, E3 ubiquitin protein ligase [Source:HGNC Symbol;Acc:6848]	1.006237645	7.413284549	7.416767579	0.00646188	0.046019279	6.645948858	7.029244774	7.678502925	8.132325486	7.085254968
ENSG00000180871	CXCR2	chemokine (C-X-C motif) receptor 2 [Source:HGNC Symbol;Acc:6027]	1.153748193	4.447115437	7.417026106	0.006460952	0.046019279	3.774188075	3.594215211	3.963622948	5.031513688	5.118566908
ENSG00000165731	RET	ret proto-oncogene [Source:HGNC Symbol;Acc:9967]	1.429635349	0.540780314	7.416050236	0.006464457	0.046019279	0.254194054	0.583576386	0.036330944	0.798919003	0.615745481
ENSG00000156042	TTC18	tetratricopeptide repeat domain 18 [Source:HGNC Symbol;Acc:30726]	1.303891817	1.387166748	7.404556889	0.006505887	0.046276678	1.063037713	1.202433699	2.06249931	1.538247531	0.415212929
ENSG00000027001	MIPEP	mitochondrial intermediate peptidase [Source:HGNC Symbol;Acc:7104]	-1.20366588	2.461408994	7.402380781	0.006513761	0.046313924	3.265262705	3.100920657	1.907011864	1.006157955	1.71760268
ENSG00000254275	RP11-89M16.1		1.824245891	0.64189445	7.397948715	0.00652983	0.046409376	-0.094677427	0.637745977	2.171650019	-0.956081001	-4.610216385
ENSG00000101986	ABCD1	ATP-binding cassette, sub-family D (ALD), member 1 [Source:HGNC Symbol;Acc:61]	1.007791581	5.118248111	7.392562491	0.006549412	0.046529713	4.323547566	4.848976944	5.034357156	5.666843359	5.356557658
ENSG00000088280	ASAP3	ArfGAP with SH3 domain, ankyrin repeat and PH domain 3 [Source:HGNC Symbol;Acc:14987]	1.362288127	0.764587205	7.390456507	0.006557084	0.046565379	0.627538984	0.637745977	0.959559782	1.069111613	0.095393329
ENSG00000168939	SPRY3	sprouty homolog 3 (Drosophila) [Source:HGNC Symbol;Acc:11271]	1.327178976	1.509170667	7.383842246	0.006581242	0.046718037	1.148882041	0.789025519	1.842443831	1.55300499	1.81594051
ENSG00000115271	GCA	grancalcin, EF-hand calcium binding protein [Source:HGNC Symbol;Acc:15990]	1.056674214	6.854864594	7.381563071	0.006589587	0.04675837	5.762549857	6.144892317	6.431546497	7.293451993	7.716050489
ENSG00000224177	LINC00570	long intergenic non-protein coding RNA 570 [Source:HGNC Symbol;Acc:43717]	-2.195408906	-0.221097885	7.380303926	0.006594203	0.046772212	0.597320328	0.836121102	-2.845623393	-7.002070102	-0.894110176
ENSG00000224997	AL049840.1	Uncharacterized protein; cDNA FLJ53535 [Source:UniProtKB/TrEMBL;Acc:B4DK98]	1.791161532	0.079465802	7.379475133	0.006597242	0.046774873	-0.196880952	-0.713501401	-0.99931534	0.984547783	0.04992943
ENSG00000269430	LRRC3DN	LRRC3 downstream neighbor (non-protein coding) [Source:HGNC Symbol;Acc:1270]	1.530725369	-0.012518843	7.373415318	0.00661951	0.046913803	-0.856736938	-0.112176905	-0.010608983	0.192266291	0.095393329
ENSG00000120278	PLEKHG1	pleckstrin homology domain containing, family G (with RhoGef domain) member 1 [Source:HGNC Symbol;Acc:20884]	-1.045837648	3.990736015	7.371890885	0.006625123	0.046934641	4.659599832	4.343766522	3.159840379	3.47074529	3.765128041
ENSG00000152213	ARL11	ADP-ribosylation factor-like 11 [Source:HGNC Symbol;Acc:24046]	1.178833221	4.211010055	7.365252226	0.006649627	0.047088648	3.270059802	3.408048696	3.599523466	4.81596796	4.990560977
ENSG00000273066	RP11-216L13.19		1.337198964	1.199656576	7.364549306	0.006652227	0.047088648	0.848890306	0.925936776	1.432844522	1.679439471	0.676839958
ENSG00000164509	IL31RA	interleukin 31 receptor A [Source:HGNC Symbol;Acc:18969]	1.637830248	1.177203543	7.362174388	0.006661019	0.047131879	0.796654307	0.689955027	0.210162819	2.106994346	1.134350211
ENSG00000179397	C1orf101	chromosome 1 open reading frame 101 [Source:HGNC Symbol;Acc:28491]	1.422560513	0.185249923	7.359130146	0.006672306	0.047192723	-0.046168491	0.056395802	0.28980717	0.114810932	0.139468177
ENSG00000132470	ITGB4	integrin, beta 4 [Source:HGNC Symbol;Acc:6158]	-1.434396202	1.345867818	7.355262591	0.006686674	0.047256267	2.304339895	2.068117593	-0.161352853	-1.231517591	0.973207307
ENSG00000175061	FAM211A-AS1	FAM211A antisense RNA 1 [Source:HGNC Symbol;Acc:28619]	-1.163828968	8.186586087	7.355523034	0.006685706	0.047256267	8.824219999	8.564105195	8.014408596	7.232878216	7.75287062
ENSG00000257702	LBX2-AS1	LBX2 antisense RNA 1 [Source:HGNC Symbol;Acc:25136]	1.358330496	1.919436653	7.35042267	0.006704699	0.047364585	1.531025871	1.050973916	1.89432737	2.491046607	2.120832889
ENSG00000261996	CTC-281F24.1		1.495877221	0.41714269	7.347814364	0.006714434	0.047395208	-0.489407866	0.498306264	1.120696904	0.722650538	-1.18885156
ENSG00000143061	IGSF3	immunoglobulin superfamily, member 3 [Source:HGNC Symbol;Acc:5950]	-1.401462209	1.004725071	7.344976241	0.006725042	0.04745101	1.883815937	1.997369264	-0.99931534	-0.406691783	-0.376928291
ENSG00000204632	HLA-G	major histocompatibility complex, class I, G [Source:HGNC Symbol;Acc:4964]	1.051900528	4.502240137	7.328995724	0.006785095	0.047836285	3.998873122	3.783314832	4.305306726	5.190971206	4.754122018
ENSG00000105948	TTC26	tetratricopeptide repeat domain 26 [Source:HGNC Symbol;Acc:21882]	-1.330606475	1.839178966	7.32651943	0.00679445	0.047883007	2.798442909	2.369639072	0.6928072	0.265774258	1.410986673
ENSG00000117425	PTCH2	patched 2 [Source:HGNC Symbol;Acc:9586]	1.383549148	0.448712955	7.302763092	0.006884865	0.048500727	0.254194054	0.468724906	0.6928072	0.434658742	0.002986071
ENSG00000169258	GPRIN1	G protein regulated inducer of neurite outgrowth 1 [Source:HGNC Symbol;Acc:24835]	1.400363076	0.551438443	7.301219368	0.006890783	0.048522943	0.090278201	0.498306264	0.505333341	0.669468625	0.646616095
ENSG00000177096	FAM109B	family with sequence similarity 109, member B [Source:HGNC Symbol;Acc:27161]	-1.124077846	3.066512988	7.297885336	0.006903581	0.048592364	3.701474281	3.541909135	1.956663165	2.404187718	2.956635145
ENSG00000157657	ZNF618	zinc finger protein 618 [Source:HGNC Symbol;Acc:29416]	-1.094603082	3.373908118	7.297209782	0.006906178	0.048592364	3.878388807	3.847931966	2.213098418	2.756300061	3.465217709

ENSG00000214651	RP11-477J21.2		1.012560304	5.086389828	7.295696026	0.006911999	0.048594368	4.154846016	4.796364522	4.976862123	5.604456613	5.454673912
ENSG00000153291	SLC25A27	solute carrier family 25, member 27 [Source:HGNC Symbol;Acc:21065]	1.482088027	-0.151892453	7.296393361	0.006909317	0.048594368	-0.425964666	0.171040209	0.125863448	-0.244792505	-1.3021922
ENSG00000136541	ERMN	ermin, ERM-like protein [Source:HGNC Symbol;Acc:29208]	1.247885933	2.1950465	7.293006748	0.006922353	0.048647679	1.414540624	2.135558076	2.867077005	2.460063232	1.463945021
ENSG00000168769	TET2	tet methylcytosine dioxygenase 2 [Source:HGNC Symbol;Acc:25941]	1.028380859	6.849144786	7.278392716	0.006978895	0.048986208	5.904283452	6.306201362	6.673926768	7.570458388	7.179635047
ENSG00000258461	RP11-164J13.1		1.324417261	1.173071866	7.279096357	0.006976162	0.048986208	0.534915169	1.050973916	1.501363361	1.048430793	1.374569317
ENSG00000164611	PTTG1	pituitary tumor-transforming 1 [Source:HGNC Symbol;Acc:9690]	-1.359789916	1.986153267	7.263492115	0.007037033	0.049335107	2.811663962	2.747648049	0.328039018	1.224680533	1.11240278
ENSG00000198478	SH3BGRL2	SH3 domain binding glutamic acid-rich protein like 2 [Source:HGNC Symbol;Acc:15567]	1.087905207	4.901064046	7.259639153	0.007052146	0.049421326	4.104705577	4.573047022	4.610737206	5.554913634	5.196262588
ENSG00000162949	CAPN13	calpain 13 [Source:HGNC Symbol;Acc:16663]	1.416328086	0.201575975	7.252661454	0.007079601	0.049574148	-0.14487434	0.171040209	0.125863448	-0.009775356	0.450630967
ENSG00000174137	FAM53A	family with sequence similarity 53, member A [Source:HGNC Symbol;Acc:31860]	1.440356881	0.22552322	7.243546788	0.007115628	0.049786708	0.214927798	0.171040209	-0.329705368	0.497040107	0.095393329
ENSG00000092621	PHGDH	phosphoglycerate dehydrogenase [Source:HGNC Symbol;Acc:8923]	-1.141456615	2.969086885	7.239957492	0.007129867	0.049866459	3.255620391	3.446494941	2.243421497	1.109604583	3.528738911
ENSG00000232533	AC093673.5		1.419195322	3.348472576	7.238827901	0.007134354	0.04987797	2.438230535	2.854443549	2.532495892	4.119977163	3.889710441
